# Characterisation of Methicillin-Resistant *Staphylococcus aureus* from Alexandria, Egypt

**DOI:** 10.3390/antibiotics12010078

**Published:** 2023-01-01

**Authors:** Stefan Monecke, Amira K. Bedewy, Elke Müller, Sascha D. Braun, Celia Diezel, Amel Elsheredy, Ola Kader, Martin Reinicke, Abeer Ghazal, Shahinda Rezk, Ralf Ehricht

**Affiliations:** 1 Leibniz Institute of Photonic Technology (IPHT), 07745 Jena, Germany; 2 InfectoGnostics Research Campus, 07743 Jena, Germany; 3 Institute for Medical Microbiology and Virology, Dresden University Hospital, 01307 Dresden, Germany; 4 Department of Microbiology, Medical Research Institute, Alexandria University, Alexandria 5424041, Egypt; 5 Institute of Physical Chemistry, Friedrich-Schiller University, 07743 Jena, Germany

**Keywords:** *Staphylococcus aureus*, DNA microarrays, MRSA, hospital-acquired MRSA, community-acquired MRSA, Panton-Valentine-leukocidin, *mecA*, *fusC*, fusidic acid resistance

## Abstract

The present study aims to characterise clinical MRSA isolates from a tertiary care centre in Egypt’s second-largest city, Alexandria. Thirty isolates collected in 2020 were genotypically characterised by microarray to detect their resistance and virulence genes and assign them to clonal complexes (CC) and strains. Isolates belonged to 11 different CCs and 14 different strains. CC15-MRSA-[V+*fus*] (n = 6), CC1-MRSA-[V+*fus+tir+ccrA/B-1*] (PVL+) (n = 5) as well as CC1-MRSA-[V+*fus+tir+ccrA/B-1*] and CC1153-MRSA-[V+*fus*] (PVL+) (both with n = 3) were the most common strains. Most isolates (83%) harboured variant or composite SCC*mec* V or VI elements that included the fusidic acid resistance gene *fusC*. The SCC*mec* [V+*fus+tir+ccrA/B*-1] element of one of the CC1 isolates was sequenced, revealing a presence not only of *fusC* but also of *blaZ*, *aacA-aphD* and other resistance genes. PVL genes were also common (40%). The hospital-acquired MRSA CC239-III strain was only found twice. A comparison to data from a study on strains collected in 2015 (Montelongo et al., 2022) showed an increase in *fusC* and PVL carriage and a decreasing prevalence of the CC239 strain. These observations indicate a diffusion of community-acquired strains into hospital settings. The beta-lactam use in hospitals and the widespread fusidic acid consumption in the community might pose a selective pressure that favours MRSA strains with composite SCC*mec* elements comprising *mecA* and *fusC*. This is an unsettling trend, but more MRSA typing data from Egypt are required.

## 1. Introduction

*Staphylococcus aureus* (*S. aureus*) is a human and animal pathogen that is a global cause of morbidity and mortality. Antimicrobial resistance in *S. aureus* is frequently associated with mobile genetic elements, including plasmids, transposons, and staphylococcal cassette chromosome (SCC) elements that act as carrier to exchange genetic information between *Staphylococcus* strains. Methicillin resistance in staphylococci is based on the production of altered penicillin-binding proteins with a low affinity for beta-lactam antibiotics. These proteins are encoded by different *mec* genes (*mecA* or *mecC*), out of which *mecA* is the most common and widespread one [1]. SCC*mec* elements carry *mec* genes along with the genes that control their expression. There are three basic genetic units within SCC*mec*: the *ccr* recombinase gene complex, the *mec* gene complex and the joining region (J region) [2,3,4,5,6]. Other SCC elements might carry fusidic acid resistance (mediated by the *fusC* gene), heavy metal resistance, or certain virulence factors such as *tirS* or a phenol-soluble modulin (PSM-*mec*) [7,8].

MRSA has been recognized for decades as a common cause of nosocomial infections, resulting in increased mortality, longer hospitalisations, and higher costs to healthcare systems. Healthcare-associated MRSA (HA-MRSA) are *S. aureus* isolates obtained from patients two or more days after hospitalisation or from patients with a history of recent hospitalisation, surgery, dialysis, or residence in a long-term care facility with an indwelling medical device at the time of culture [9]. However, some MRSA strains can disseminate among otherwise healthy individuals, leading to community-acquired infections [10,11,12,13,14,15,16,17,18,19]. Community-associated MRSA (CA-MRSA) differ from typical HA-MRSA strains in not only epidemiological background, but also in their antibiotic susceptibility profile and in genotypic features. These include the presence of the Panton–Valentine leukocidin (PVL, [17,18,19,20,21]) and the carriage of SCC*mec* IV or V elements. However, strains or lineages traditionally considered “community-associated” can also cause nosocomial outbreaks, and there are strains associated with medical care outside hospitals. In addition, parts of the world—including India, the Middle East, and North Africa—are currently plagued by an emergence of PVL-positive, multi-resistant strains harbouring SCC*mec* IV and V elements, which can be found both in hospitals as well as in the community. Thus, distinctions between HA-MRSA and CA-MRSA become increasingly blurry, and epidemiological considerations are only sometimes compatible with a genotype-based definition of CA-MRSA [22].

Although molecular typing data for *S. aureus* and MRSA are abundantly available for Western Europe, North America and Australia, comparatively few studies were conducted to describe the *S. aureus*/MRSA epidemiology in the rest of the world [23,24,25,26,27]. For the Middle East, previous work showed a high rate of MRSA, a high diversity of different clonal complexes and strains, and a high rate of PVL carriage and of resistance to fusidic acid, either mediated by plasmids or by SCC*mec* elements harbouring *fusC* [28,29,30,31,32,33,34,35]. A detailed molecular characterisation of clinical *S. aureus* isolates from Africa has been largely neglected in the past [36]. Some localised studies and analysis of cases of *S. aureus*-related infections in returning travellers have suggested that African *S. aureus* might have a different genetic background and might be more virulent than isolates from Europe with Africa being an endemic region for PVL-positive *S. aureus* [37,38,39].

At the crossroads of Africa and the Middle East, Egypt is among the countries where only anecdotal typing data are available. Some work focused on livestock animals and contact persons [40,41,42], but in general, the epidemiology of human MRSA infection, regardless of whether HA or CA, needs to be better understood [42]. This is regrettable, as MRSA appears to be abundant. A recent study revealed an extremely high MRSA rate, with about 80% of total *S. aureus* isolates being MRSA [43]. A recently published sequencing study included clinical methicillin-susceptible *S. aureus* (MSSA) and MRSA isolates from Alexandria [44], collected in 2015. In this study, the most common MRSA strains were the pandemic hospital-associated ST239-MRSA-III, CC80-MRSA-IV (a PVL-positive strain frequently found in Mediterranean and Middle Eastern countries as well as in Western European tourists returning from there) and a variety of CC1 strains.

Various molecular techniques have been developed to study the genetic diversity of *S. aureus* strains and, in particular, MRSA strains [45,46]. DNA microarray technology allows the simultaneous detection of many molecular targets, including resistance genes and virulence factors. The overall hybridization profile could also be used as a fingerprint, or a dataset, that allows elucidating relatedness between different isolates and allocating them to strains [23,47] based on a framework of clonal complexes (CC), as initially defined by multilocus sequence typing (MLST; [48,49,50]), and on their SCC*mec* types. The present study aimed to characterise clinical MRSA isolates from a tertiary care centre in Egypt’s second-largest city, Alexandria.

## 2. Results

Thirty-four clinical isolates of *S. aureus* were obtained from routine diagnostic procedures at the microbiology laboratory of the Medical Research Institute, Alexandria University over five months in 2020. Most MRSA isolates were obtained from aspirated pus followed by wound swabs (Table 1). Four isolates were excluded from further analysis based on PCR and microarray experiments. The *mecA* gene was absent in one isolate that phenotypically was tested as cefoxitin-resistant; three isolates were PCR-negative for *femA* and were by microarray categorised as coagulase-negative, albeit *mecA*-positive, staphylococci.

### 2.1. Resistance Genes and Antibiotic Resistance, SCCmec Elements

MRSA isolates were tested against 19 antimicrobial discs (Table 2). Apart from cefoxitin, the isolates exhibited the highest rates of resistance to gentamicin (90%), tobramycin (90%), and fusidic acid (86.7%).

All isolates harboured *mecA* as part of various SCC*mec* elements. SCC*mec* elements I, II, VT, VII, VIII, IX, X and XI (including *mecC*) were not found. Two isolates harboured composite SCC*mec* III elements. Another three isolates carried “plain” SCC*mec* IVa elements. All others had composite elements that included SCC*mec* IV, V, or VI and SCC*fus*. Accordingly, the SCC-associated *fusC* gene was found in 25 isolates, i.e., in 83%.

In contrast, the plasmid-borne fusidic acid resistance gene *far-*1 was not detected. This gene is usually associated with the PVL-positive CC80-MRSA-IV, which was conspicuously absent. The mupirocin resistance gene *mupA* was detected once in a CC1-MRSA-[V+*fus+tir+ccrAB1*] isolate that also harboured *cfr* and *aadD*. The multidrug resistance gene *cfr* was found once, although no linezolid resistance was observed phenotypically. Vancomycin resistance genes were not detected which was in accordance to the phenotypic glycopeptide susceptibility of all isolates. The gene *fexA* was detected in two CC5 isolates, and both were phenotypically resistant to chloramphenicol. SCC*mec* markers and resistance genes, as detected by array hybridisation, are listed in Table 3.

### 2.2. Virulence Factors

Regarding virulence factors (Table 4), 12 isolates (40%) were Panton–Valentine leucocidin (PVL)-positive, of which five belonged to CC1, three to CC 1153, two to CC 152, one to CC 121 and one to CC 30. The enterotoxin gene cluster (*egc*, consisting of *seg*, *sei*, *selm*, *seln*, *selo* and *selu*) was found in all CC5, CC22, CC30, and CC121 isolates, representing 20% of all the tested isolates. The toxic shock toxin gene (*tst1*) was found in all CC22 isolates (6.7%). Enterotoxin genes *sec* and *sel* as well as the exfoliative toxins genes *etB* and *etD* were not detected. However, *etD2=etE* yielded signals in two isolates belonging to CC152 (6.7%). The epidermal cell differentiation inhibitor gene *edinB* was also found in these two isolates. The *edinA* gene was not identified in any of the isolates, and *edinC* was present in only one isolate belonging to CC5. Various combinations of the immune evasion complex (IEC) genes (*sea*, *sak*, *chp* and *scn*) were also detected in most isolates. Amongst the IEC-positive isolates, IEC type D (*sea*, *sak*, and *scn*) predominated (36.6%). Amongst the individual IEC genes, the most predominant gene was *scn* (in all but one CC1 isolate). The gene encoding surface-anchored protein X, *sasX=sesI*, was not found, neither in the two CC239 isolates (which represent the lineage from which it was initially described) nor elsewhere.

### 2.3. Strain Affiliations

This study identified 11 different CCs and 14 strains (as defined by CC affiliation, toxin gene carriage, and SCC*mec* subtype; Table 5). A recently published sequencing described MRSA from Alexandria [44] collected in 2015. Forty-six genomes from this study were analysed (see Section 4) and assigned to strains allowing a direct comparison of strain prevalence in 2015 and 2020.

### 2.4. The SCCmec Element in CC1-MRSA-[V+fus+tir+ccrA/B-1]

Eight isolates harboured SCC*mec* elements described according to the array profiles as SCC*mec* [V+*fus+tir+ccrA/B*-1]. All these isolates belonged to CC1. To the best of our knowledge, no contiguous sequence of such an element was yet available, as related MUM475 (GenBank AZSG01000015.1 plus AZSG01000034) and MRSA1_ST20130096 (FSRY01000032.1) carry SCC*mec* [VT+*fus+tir+ccrA/B*-1] elements. Thus, one representative isolate (the PVL-negative Alexandria_2020-19) was fully sequenced using nanopore technology. This confirmed affiliation to CC1, Sequence Type 1 and the absence of PVL from that particular isolate (although another prophage inhabited the integration site usually occupied by PVL phages).

**Figure 1:**
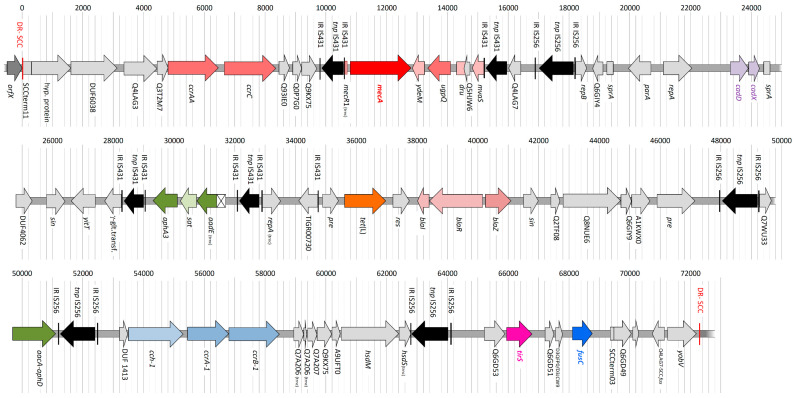
Schematic representation of the SCC*mec* [V+*fus+tir+ccrA/B*-1] element from a CC1 isolate (GenBank CP113244.1).

Its SCC*mec* element spanned 72,298 bp. Its sequence and a detailed list of all identified genes are provided as supplemental files S2/S3, and a schematic representation is provided in Figure 1.

It comprised the markers of a typical SCC*mec* V element (*mecA*, *ugpQ*, *ccrAA*, *ccrC*, *mvaS*-SCC), but it lacked the additional *ccrAA/ccrC* and D1GU38 genes that define SCC*mec* VT as they are present in the previously released CC1 sequence MUM475 (GenBank AZSG01000015.1 plus AZSG01000034).

The SCC*mec* V genes were combined with several transposase genes and integrated mobile genetic elements that, among various “putative proteins”, also included heavy metal resistance genes *cadD+cadX*. The aminoglycoside and streptothricin resistance genes *aphA3* and *sat* were also present. The gene *aadE* (that typically accompanies these two genes) was found to be truncated from 909 bp to 624 bp. This truncation was also present in other Egyptian CC1 sequences (JAEOUR010000048.1 and JAEOWJ010000028.1 [44]), but it can also be found in entirely unrelated strains such as TCH1516 (CP000731.1, pos. 15,611 to 16,252). Furthermore, there was the tetracycline resistance gene *tet*(L), the penicillinase operon *blaI+blaR+blaZ* and the gentamicin/tobramycin resistance gene *aacA-aphD*.

Then, *ccrA/B-1* recombinase genes and a truncated gene cluster encoding an incomplete type I restriction–modification system followed. The *hsdM* gene was still present there, *hsdS* was truncated and *hsdR* has been replaced by yet another transposase copy which also removed the first gene (for a “putative protein” Q6GD54) from the actual *fusC*-associated complex. This complex included, among other genes, *tirS*, *fusC* and *yobV*; it was with regard to gene content as well as to allelic variants most closely related to the one in other CC1 strains (MSSA476, BX571857 and KT/314250, AOCP01000013) and thus it could be assigned to the previously defined [35] *fusC-*complex class “A”. The association of a *fusC*-complex class “A” with *ccrA/B-1* recombinase genes is typical for, but not restricted to, CC1 strains [35].

## 3. Discussion

With regard to antimicrobial resistance, the most remarkable observation of the present study was a presence of *fusC* in diverse lineages of *S. aureus* and its extremely high prevalence. It was as high as 83%%, while in 2015 [44], the rate of genotypically fusidic acid (FA)-resistant strains (e.g., positive either for *fusC* or *far1*) was 30,4%. This could indicate an alarming trend, although several caveats (low numbers, absence of data from other hospitals, other towns and provinces, and predominance of skin/soft tissue infections among study samples) apply. As many genotypically different strains were involved, a local outbreak situation as a cause for this observation can likely be ruled out. A high and possibly increasing rate of genotypic FA resistance is likely to be related to a high rate of consumption of that drug, as observed in other countries such as New Zealand, where an increase in FA consumption led to a parallel increase in MRSA with composite SCC[*mec*+*fus*] elements [54]. Indeed, FA is over-the-counter available in Egyptian pharmacies, without prescription, as ointment, cream, or eye-drops. It is extensively misused and/or overused as monotherapy, even for non-infectious skin conditions or for prophylaxis. It is inexpensive, with prices as low as 15 to 25 Egyptian Pounds (*ca.* 0.75 to 1.30 Euro) for 15 g crème with 2% FA content. MRSA with composite SCC[*mec*+*fus*] elements can be expected to have a clear evolutionary advantage under such conditions in both ecological niches, in hospitals and in the community. In hospitals, they thrive because of their beta-lactam resistance (with about half of all antibiotics used being beta-lactams; see [55]). The high consumption of FA in the community poses a selective pressure favouring *fusC-*positives. When *mecA* and *fusC* are located on the same mobile genetic element, outpatient use of FA promotes MRSA in the same way as an in-hospital use of beta-lactams favours FA resistance. This might be a reason for a blurring of the distinction between CA- and HA-MRSA. It also means that the excessive use of FA in the community eventually endangers the lives of Egyptian hospital patients. Therefore, the use of FA should be curtailed, e.g., by requiring a prescription by a physician, as has been recently done in the U.A.E.

Another interesting observation was the high rate of carriage of PVL genes. Similar observations were made at other study sites in Egypt [56] and the Middle East [57,58]. A previous study [56] from another Egyptian city (Cairo) observed, in 20202, an even higher PVL rate of 75% (29% in hospital- and 92% in community-acquired infection) clearly indicating that the rate observed here was no outlier. In general, Middle Eastern and Northern African studies indicate that PVL-MRSA are no longer restricted to the “community” (if they ever were in this part of the world) but also thoroughly infiltrated hospital settings. The high PVL prevalence in our study, as well as in others from the region, cannot be attributed to a single outbreak strain, simply because of a high genotypic diversity of PVL-positive strains. However, the high proportion of wound and pus samples could in our case have caused a bias towards PVL-positive strains. The PVL rate for MRSA collected in Alexandria in 2015 was much lower 17% [44], and it cannot be ruled out that the lower rate observed in this study could be related to an outbreak of a PVL-negative strain (CC239).

The recent publication of genomes of MRSA isolates also from Alexandria [44] allowed to look at temporal changes affecting population structure, as defined by affiliations to CCs.

CC1 strains with SCC*mec* V or VT (as in MUM475, GenBank AZGS) elements that additionally harbour *fusC* and the virulence factor *tirS* have frequently been observed in various countries. However, in most cases, whether they originated from SCC*mec* V or VT elements was not determined. Some previously described isolates [52,59] with Middle Eastern or Eastern African provenance indeed harboured composite elements based on SCC*mec* V rather than on SCC*mec* VT, as all Egyptian isolates described herein or in the earlier study [44] did. As discussed above, one isolate was sequenced to characterise its SCC*mec* element. It was found to harbour *mecA*, *fusC*, and several other resistance genes (including *aacA-aphD*), fitting into a broader trend of increasing “multi-resistance” in supposedly community-associated SCC*mec* IV/V strains. Another aminoglycoside resistance gene in this strain’s SCC*mec* element, *aadE*, appeared to be truncated in our sequence. This was not a sequencing artifact, as corresponding contigs of previously sequenced CC1 strains [44] and strains from unrelated lineages (e.g., TCH1516, CC8 or TW20, CC239) showed the same. This likely indicated that this truncation predated the acquisition of the mobile genetic element carrying *aphA3/sat/aadE* by diverse MRSA strains.

Egyptian CC5-MRSA-[VI+*fus+tir*] can be discerned from a similar strain from Portugal (HDE288; AF411935,3) based on differences in its SCC*mec* element, including an absence of *dcs*, but isolates match a strain that was repeatedly found in Middle Eastern countries (Kuwait: [60]; K.S.A.: [61]; U.A.E.: [62]).

Not much is known about CC6-MRSA-[V+*fus*], although a similar or related strain was observed in Kuwait [33]. In general, CC6 MRSA are common and widespread in the Middle East, but previously described strains usually differ in having SCC*mec* type IV [61,62,63,64].

CC15-MRSA-[V+*fus*] is a remarkable strain given that it is nearly the only MRSA strain that emerged from the globally spread and common lineage CC15 [65,66,67]. It has been found in humans in Saudi Arabia and other Gulf countries and in livestock and camel meat [68,69,70,71]. It was also observed in chickens from Egypt (unpubl. communication with Dr. Hotzel, Jena, Germany), and it was detected in a farmer from the Nile Delta region in Egypt [42]. In 2015, this MRSA strain was not found in Alexandria [44].

CC22-MRSA-IVa carrying the *tst1* gene has been frequently observed around the Mediterranean Sea [72], in Middle Eastern counties [69,73,74,75,76], and among refugees from the Middle East after it was first from Gaza [77,78,79]. It was also found in Egypt, in livestock, and in farm personnel [42], as well as in an Alexandrian hospital [44]. Thus, its detection is not surprising, but it was remarkable for being one of three lineages that did not harbour SCC-encoded fusidic acid resistance.

PVL-positive CC30-MRSA-IV (PVL+) has been dubbed the “WSPP/Southwest Pacific Clone” after an initial outbreak among New Zealanders and Samoans [80,81]. Meanwhile, such strains can be found globally, but different SCC*mec* subtypes might indicate a polyphyletic emergence. SCC*mec* IVa, as in the present isolate, has been observed in WSPP-like isolates from Europe and the Middle East [52,62] and the U.S. (GenBank CP026066).

CC97-MRSA-[V+*fus*] has been found in Europe and the Middle East [69], and it was detected some years ago in chicken meat brought from Egypt to Germany [82], indicating both a prolonged presence in Egypt as well as a possible livestock connection. Other, *fusC*-negative, CC97-MRSA have also been observed in Egypt [44].

CC121-MRSA-[V+*fus*] (PVL+) is another rare MRSA strain emerging from a globally spread and common MSSA lineage [65,83,84,85,86]. The authors have observed related or similar strains in the Middle East [33,42,62].

CC152-MRSA-[V+*fus*] belongs to a lineage known to be common in Africa, but these isolates are usually MSSA. The particular MRSA strain was observed in Egyptian livestock and contact persons [42], and again, the presence of PVL could be seen as an indication of transmission from humans to animals. Other observations came from the Arabian Gulf [62] and Egypt [44]. CC152 was recently shown to carry a novel *etD/E* homologue, *etE2* [53]. The observation of weak and/or irregular signals for *etD2* likely can be attributed to a presence of this gene.

CC239-MRSA is a comparatively ancient, truly pandemic lineage of hospital-acquired MRSA with a core genome that can be described as a chimera of CC8 and CC30 [87] harbouring the large and distinct SCC*mec* III element [3]. Many variants of that strain largely correlate with geographic regions of origin [88,89,90,91,92]. The two isolates found in our study were not identical. One isolate belonged to a clade previously identified in various Middle Eastern countries or people from there [88]. The other isolate matched a group of strains and sequences from Western Europe (Portugal), the U.S. (ATCC33592), and Russia [88], as well as from Egypt [44]. Since it differed only in the absence of *ccrAA/C* recombinase genes from the abovementioned clade, this entire group, or single specimens out of it, might be a mere deletion variant of the Middle Eastern clade. CC239-MRSA was previously found in Alexandria [44], and then (in 2015) it was the most common strain, comprising nearly half of the MRSA isolates characterised, i.e., 23 out of 47 isolates (49%) that belonged to various variants of the ST239-MRSA-III strain.

The issue of the receding CC239 clone might, although the numbers of typed strains are low, suggest a profound change in the MRSA population structure, blurring the distinction between HA- and CA-MRSA. As mentioned in the Introduction, there was an idea of distinguishing CA- and HA-MRSA by molecular means, e.g., based on PVL status, type of SCC*mec* elements, and affiliation to “unusual” clonal complexes. This concept might still apply in countries such as China (where CC239-MRSA-III at least until recently predominated in hospitals and CC59-MRSA-IV or -V in the community [93,94]) or the USA (where CC5-MRSA-II used to be common in hospitals, while PVL-positive CC8-MRSA-IV prevail in the community). However, among the Egyptian sample analysed herein, only two out of 30 hospital isolates could be assigned to the CC239 strain traditionally associated with a hospital-acquired infection. In contrast, all others belonged to various strains with features associated with CA-MRSA (PVL, SCC*mec* IV/V/VI elements). Similar observations were also made in Middle Eastern countries [37]. No molecular marker can be used in these settings anymore as a surrogate for the assignment to hospital- versus community-acquired infections. In order to discern these, one must thoroughly interview the patient and assess the case history.

CC1153-MRSA-[V+*fus*] (PVL+) is a strain that we previously found in a patient of Egyptian origin in Germany [35] and Dubai [58]. Furthermore, isolates for which it was not determined whether they harboured SCC[*mec* V+*fus*] or SCC[*mec* VT+*fus*] elements have been observed in the United Arab Emirates, Saudi Arabia, and Kuwait [35]. PVL-positive MSSA from this lineage has also been found in Egypt’s livestock [40]. Given the pathogenetic role of PVL in humans, this might be attributed to an anthropozoonotic transition. However, this observation raises the question whether ancestral, susceptible CC1153 strains might already have circulated in Egypt prior to the emergence of MRSA from this lineage. In 2015, no CC153 MRSA was found [44], possibly indicating a recent emergence.

Finally, the PVL-positive CC80-MRSA-IV strain, widespread in the Mediterranean and the Middle East [30,73,95,96,97,98,99] and previously found in Alexandria [44], was not observed. Whether this is due to the small sample or a recent decline still needs to be established.

A limitation of the study is of course the small sample size, resulting from opportunistic sampling at a single location. The predominance of isolates from swab/pus specimens might have caused a bias towards *fusC*- and/or PVL-positive isolates that might be less common in other types of samples.

More comprehensive surveys into *S. aureus*/MRSA populations in Egypt and elsewhere in Africa and the Greater Middle East are urgently needed as well as studies on a possible impact of new antibiotics such as daptomycin and fifth generation cephalosporins. A two-pronged approach of array-based typing followed by genome sequencing of “interesting” or conspicuous strains, as described herein, might help to gather more typing data for these parts of the world.

## 4. Materials and Methods

### 4.1. Isolates

Clinical samples (see Table 1) were cultured routinely on blood agar (Oxoid Ltd., Basingstoke, Hampshire, UK) and MacConkey agar and were incubated at 37 °C under aerobic conditions for 16–24 h. Subculturing was aided by microscopy; Gram-positive cocci were further identified by biochemical tests, including Catalase test and Coagulase test (Remel-Oxoid, Basingstoke, Hampshire, UK) and they were subjected to antibiotic susceptibility testing by disc diffusion (Oxoid Ltd., Basingstoke, Hampshire, UK) using CLSI methodology and breakpoints (CLSI; https://clsi.org/media/3481/m100ed30_sample.pdf; accessed on 1 December 2022). In addition, identification as *S. aureus*/MRSA was confirmed by PCR (see below). For long-term storage at the laboratory in Egypt, one ml of fresh saturated bacterial culture grown on Luria Bertani (LB) broth was added to one ml of sterile glycerol solution in screw capped glass tubes. The tubes were stored at −20 °C. Strains at the German laboratory were stored at −80 °C using microbank tubes (Fisher Scientific GmbH, Schwerte, Germany/Pro-Lab Diagnostics, Richmond Hill, ON, Canada) according to the manufacturer’s instructions. For re-culturing, one loop of bacterial material was streaked over blood agar and incubated overnight at 37 °C.

### 4.2. PCR for S. aureus/MRSA

Multiplex PCR was used for genotypically identifying *S. aureus* and methicillin resistance by amplification of *femA* and *mecA* genes, respectively. Nucleotide sequences of primers (Biosearch Technologies, Inc., Petaluma, CA, USA) used in this investigation are shown in Table 6.

DNA was extracted from MRSA isolates by boiling method [103]. Multiplex PCR was performed using 12 μL as total volume, consisting of 6 μL of mastermix (MyTaq HS Red Mix 2X; BioLine, London, UK), 0.5 μL of each of the diluted DNA extract, *femA*, and *mecA* primers, and 3.5 μL of PCR grade water. A negative control was prepared by adding the same contents to the tubes with water placed instead of the DNA extract. PCR cycling conditions were as follows: 4 min of initial denaturation at 95 °C, followed by 35 cycles of denaturation at 95 °C for 15 s, annealing at 52 °C for 15 s, and extension at 72 °C for 1 min, followed by a final extension at 72 °C for 5 min. PCR was performed using a Veriti Thermal Cycler (Applied Biosystems, Foster, CA, USA). The PCR products were loaded on 1.5% agarose gel and analysed by gel electrophoresis (Mupid-exU, ADVANCE Co., Ltd., Tokyo, Japan).

### 4.3. Array Procedures

For this study, a new experimental microarray was used. It was based on a previously described system [23,47]. However, it has been modified by adding probes for detecting some recently described markers and for a more detailed typing of SCC elements. These probes have already been used in earlier work when they were, among others, localised on another second array [52]. Experimental procedures were performed as described for earlier versions [23,47]; primer and probe sequences have been disclosed thence ([23,47,51,52]; see also Table 1, Table 2 and Table 3 and Supplemental Table S1 for individual target genes).

Isolates were cultured overnight at 37 °C on Columbia blood agar. Harvested cells were digested enzymatically [104]. DNA was purified using Qiagen columns (Qiagen, Hilden, Germany) according to the manufacturer’s instructions. The assay relied on a linear multiplex primer elongation using one primer per target. During amplification, biotin-16-dUTP was incorporated into the amplicons which were then hybridised to the array. After washing and blocking, horseradish–peroxidase–streptavidin was added binding to the biotin and causing local precipitation of a dye in case of a positive reaction. Finally, an array image was recorded and analysed using a designated reader and software (Arraymate, Iconoclust, both by Alere Technologies/Abbott, Jena, Germany).

### 4.4. Nanopore Sequencing

The Oxford Nanopore MinION platform was used to sequence the genome of one MRSA isolate (Alexandria_2020-19). Library preparation was done using the 1D genomic DNA by ligation kit (SQK-LSK109, version GDE_9063_v109_revX_14Aug2019; ONT) following the manufacturer’s instruction for flongles (FLO-FLG001 containing an R9.4.1 pore). Before library preparation, size selection was performed using AMPure-beads (Beckman Coulter) in a ratio of 1:1 (*v*/*v*) with the isolated DNA sample. The flongle flow cell was loaded with ca. 200 ng DNA (measured by Qubit4 Fluorometer; Thermo Fisher Scientific, Waltham, WA, USA). The sequencing ran for 48 h using the MinKNOW software version 20.10.3 starting with a total of 65 active pores.

The Guppy basecaller (version 4.4.2+9623c1626, Oxford Nanopore Technologies, Oxford, UK) translated the MinION raw reads (FAST5) into quality tagged sequence reads (4000 reads per FASTQ-file) using the barcode trimming option. Flye (version 2.8.3-b1695) was used to assemble each strain’s quality tagged sequence reads into one big circular contig. The polishing of assemblies was divided into two steps. At first, racon (v1.4.17) was iteratively used four times with the following parameter: match 8; mismatch 6; gap 8, and windows-lengths 500. Afterwards, medaka (version 1.4.3) ran on the last racon (version 1.4.21) polished assembly using the model r941_min_high_g360. This corrected assembly was used for further analysis.

### 4.5. Analysis of Previously Published Genome Sequences

Published genome sequences from the previously published paper on MRSA from Alexandria [44] were considered (GenBank JAEOUR, JAEOUS, JAEOUU, JAEOUV, JAEOUW, JAEOUX, JAEOUY, JAEOUZ, JAEOVA, JAEOVB, JAEOVC, JAEOVD, JAEOVE, JAEOVF, JAEOVG, JAEOVH, JAEOVI, JAEOVJ, JAEOVK, JAEOVL, JAEOVM, JAEOVO, JAEOVP, JAEOVQ, JAEOVR, JAEOVS, JAEOVT, JAEOVU, JAEOVV, JAEOVX, JAEOVY, JAEOVZ, JAEOWA, JAEOWB, JAEOWC, JAEOWE, JAEOWG, JAEOWH, JAEOWI, JAEOWJ, JAEOWK, JAEOWL, JAEOWM, JAEOWN, JAEOWP, JAEOWQ, JAEOWU). Sequences were analysed for the presence of the known probe sequences and their reverse complement sequences. A perfect match was assigned a score of 0.9. In case of one or two mismatches, a score of 0.4 was assigned. Probes with no hits, or with more than two mismatches, got a score of 0.0. Thus, a list of probes with corresponding scores was generated for each sequence and this list was analysed in the same way as the measurements from array experiments. This allowed us to assign previously sequenced isolates to strains using the same nomenclature and criteria for array experiments and directly compare results from both approaches. Two isolates were described as SCC*mec* un-typable (JAEOUU and JAEOWG) but were identified as CC97 and CC1 MRSA, respectively. This contradiction cannot be resolved here, but results for these sequences were included in Table 4. Another sequence (JAEOWU) included *mecA* (as also mentioned in the supplemental file to [44]) but lacked any other SCC-associated genes; it was excluded.

## 5. Conclusions

In conclusion, the population structure of MRSA from Alexandria, 2020, was characterised by the presence of many genotypically diverse strains. Most (83%) harboured SCC*mec* elements that included a fusidic acid resistance gene, and PVL was also common (40%). A comparison to an earlier study [44] from the same city in 2015 suggested a dramatic increase in the prevalence of fusidic acid resistance and of PVL carriage while a long-known strictly hospital-associated strain (CC239-MRSA-III) was receding. These observations indicate a diffusion of community-acquired strains into hospital settings and a selective pressure by beta-lactam use in hospitals and fusidic acid consumption in the community that favours MRSA strains with composite SCC*mec* elements comprising *mecA* and *fusC*. This could indicate an unsettling trend, but more data are needed to assess the current situation.

## Figures and Tables

**Table 1:** Sample types and isolates.

Sample Type	Number of MRSA Isolates	Percent
Aspirated pus	18	60.0
Wound swab	4	13.3
Blood culture	2	6.7
Bone marrow aspirate	1	3.3
Catheter tip	1	3.3
Peritoneal fluid	1	3.3
Sputum	1	3.3
Throat swab	1	3.3
Urine	1	3.3

**Table 2:** Susceptibility of MRSA isolates to antibiotics using the Kirby–Bauer disk diffusion method according to CLSI guidelines or if no interpretive criteria are recommended by the CLSI, according to EUCAST (https://www.eucast.org/fileadmin/src/media/PDFs/EUCAST_files/Breakpoint_tables/v_10.0_Breakpoint_Tables.pdf; as accessed 1 December 2022).

Antibiotic Compound	N (Suscept.)	% (Suscept.)	N (Intermed.)	% (Intermed.)	N (Resistant)	% (Resistant)
Cefoxitin	0	0.0	0	0.0	30	100.0
Vancomycin	30	100.0	0	0.0	0	0.0
Gentamicin	1	3.3	2	6.7	27	90.0
Amikacin *	1	3.3	13	43.3	16	53.3
Tobramycin *	0	0.0	3	10.0	27	90.0
Erythromycin	1	3.3	17	56.7	12	40.0
Doxycycline	0	0.0	16	53.3	14	46.7
Tigecycline *	14	46.7	12	40.0	4	13.3
Ciprofloxacin	3	10.0	14	46.7	13	43.3
Levofloxacin	2	6.7	20	66.7	8	26.7
Ofloxacin	0	0.0	14	46.7	16	53.3
Norfloxacin	0	0.0	1	3.3	0	0.0
Clindamycin	9	30.0	11	36.7	10	33.3
Trimethoprim+ sulfamethoxazole	4	13.3	9	30.0	17	56.7
Chloramphenicol	0	0.0	23	76.7	7	23.3
Rifampin	21	70.0	5	16.7	4	13.3
Linezolid	28	93.3	0	0.0	2	6.7
Fusidic acid *	0	0.0	4	13.3	26	86.7
Nitrofurantoin	0	0.0	1	3.3	0	0.0

* EUCAST criteria used.

**Table 3:** Markers used for SCC*mec* subtyping and resistance genes.

Marker	Description/Gene Product	Ref.	n	%
*mecA*	Gene encoding a modified penicillin-binding protein (PBP2a)	[23,47]	30	100.0
*mecC*	Alternate gene encoding a modified penicillin-binding protein, SCC*mec* XI	[51,52]	0	0.0
Delta *mecR1*	Truncated methicillin resistance operon repressor 1. Truncated *mec*R1 is present in SCC*mec* I, IV, V, VI, VII	[23,47]	7	23.3
*mecR1*	Methicillin resistance operon repressor 1. Un-truncated sequence in SCC*mec* II, III, VIII	[23,47]	2	6.7
*mecI*	Gene encoding a methicillin-resistance regulatory protein. Present in SCC*mec* II, III, VIII	[23,47]	2	6.7
*xylR=mecR2*	Methicillin resistance operon repressor 2, homolog of xylose repressor. Present in SCC*mec* II, III, VIII	[23,47]	2	6.7
*ugpQ*	Gene encoding glycerophosphoryl diester phosphodiesterase. Accompanies *mecA* in nearly all SCC*mec* sequences	[23,47]	30	100.0
*pls*-SCC	Gene encoding Plasmin-sensitive surface protein	[23,47]	0	0.0
*cstB-*SCC	CsoR-like sulfur transferase-regulated gene B. Used to distinguish SCC*mec* IVa from other SCC*mec* IV subtypes	[52]	3	10.0
*kdpA+B+D-*SCC	SCC-borne ATP-driven potassium transport (KDP) system, SCC*mec* II	[23,47]	0	0.0
D1GU38	Putative protein. Used for identification of SCC*mec* VT, SCC*mec* ZH47, SCC*mec* VII because of an association with (additional/second) *ccrC* copies	[52]	0	0.0
B2Y834	Abortive phage resistance protein. Used for identification of SCC*mec* IV A, G, c and SCC*mec* MRSAZH47	[52]	0	0.0
B6VQU0	Putative protein. Used for identification of SCC*mec* IVh/j	[52]	0	0.0
Q3YK51	*Putative* protein. Subtyping SCC*mec* IV, i.e., identification of SCC*mec* IV g	[52]	0	0.0
*tirS*	Staphylococcal TIR-protein binding protein	[52]	11	36.7
*arcA+B+D-*SCC	Genes encoding the arginine metabolic operon from ACME-1/-2 elements	[23,47]	0	0.0
*opp3B* and *speG*	Genes encoding oligopeptide permease and spermidine N-acetyltransferase. Associated with ACME or composite SCC*mec*/ACME elements	[52]	0	0.0
*ccrA-1+ccrB-1*	Cassette chromosome recombinase genes, type 1	[23,47]	8	26.7
*ccrA-2+ccrB-2*	Cassette chromosome recombinase genes, type 2	[23,47]	3	10.0
*ccrA-3+ccrB-3*	Cassette chromosome recombinase genes, type 3	[23,47]	2	6.7
*ccrAA+ccrC*	Cassette chromosome recombinase gene C and associated *ccr* homologue	[23,47]	24	80.0
*ccrA-4+ccrB-4*	Cassette chromosome recombinase genes, type 4	[23,47]	2	6.7
Q9XB68-*dcs*	Located at the terminus of SCC*mec* directly next to *orfX*	[23,47]	0	0.0
*merA+merB*	Genes from the mercury resistance operon	[23,47]	0	0.0
*czrC*	Cadmium and zinc resistance gene C, heavy metal translocating P-type ATPase. Frequently associated with livestock MRSA	[52]	0	0.0
*cadD* (R35)	SCC-borne cadmium resistance gene, used for subtyping CC239-MRSA-III clades	[52]	2	6.7
*blaZ+blaI+blaR*	Penicillinase operon (excluding the SCC*mec* XI-associated allele)	[23,47]	28	93.3
*blaZ*_(SCC*mec* XI)_	Gene encoding beta-lactamase, from SCC*mec* XI	[51,52]	0	0.0
*erm*(A)	rRNA adenine N-6-methyl-transferase conferring erythro-/clindamycin resistance	[23,47]	1	3.3
*erm*(B)	rRNA adenine N-6-methyl-transferase, erythro-/clindamycin resistance	[23,47]	1	3.3
*erm*(C)	rRNA adenine N-6-methyl-transferase, erythro-/clindamycin resistance	[23,47]	4	13.3
*lnu*(A)	Lincosamide-nucleotidyltransferase (=*linA*)	[23,47]	5	16.7
*lsa-E*	Lincosamide ABC transporter	[52]	0	0.0
*msrA*	Macrolide resistance ABC transporter, ATP-binding protein	[23,47]	5	16.7
*mefA*	Macrolide efflux protein A	[23,47]	0	0.0
*mph*(C)	Macrolide 2′-phosphotransferase II (=*mpbBM*)	[23,47]	0	0.0
*vat*(A), *vat*(B)	Acetyltransferase inactivating streptogramin A, virginiamycin	[23,47]	0	0.0
*vga*(A), *vgb*	Streptogramin A resistance genes	[23,47]	0	0.0
*aacA-aphD*	Bifunctional enzyme Aac/Aph (6′-aminoglycoside N-acetyltransferase and 2′′-aminoglycoside phosphotransferase), gentamicin/tobramycin resistance	[23,47]	25	83.3
*aadD*	Aminoglycoside adenyltransferase, tobramycin resistance	[23,47]	6	20.0
*aphA3*	3′5′-aminoglycoside phosphotransferase, neo-/kanamycin resistance	[23,47]	12	40.0
*sat*	Streptothricine acetyltransferase	[23,47]	9	30.0
*dfrA*	Dihydrofolate reductase type 1	[23,47]	4	13.3
*fusC*	SCC-associated fusidic acid resistance gene (=Q6GD50)	[23,47]	25	83.3
*far1*	Plasmid borne fusidic acid resistance gene (=*fusB*)	[23,47]	0	0.0
*mupA*	(High level) mupirocin resistance protein	[23,47]	1	3.3
*tet*(K)	Tetracycline resistance gene	[23,47]	9	30.0
*tet*(L)	Tetracycline resistance gene	[52]	9	30.0
*tet*(M)	Tetracycline resistance gene	[23,47]	4	13.3
*cat*	Chloramphenicol acetyltransferase	[23,47]	1	3.3
*cfr*	23S rRNA methyltransferase encoding resistance towards Lincosamides, Oxazolidinones, Pleuromutilins, Streptogramin A etc.	[23,47]	1	3.3
*fexA*	Chloramphenicol/florfenicol exporter	[23,47]	2	6.7
*qacA*, *qacC*	Quaternary ammonium compound resistance proteins A and C	[23,47]	0	0.0
*vanA*, *vanB*, *vanZ*	Glycopeptide resistance genes	[23,47]	0	0.0

**Table 4:** Virulence factors.

Marker	Description/Gene Product	Ref.	n	%
*lukF-PV+lukS-PV*	Phage-borne Panton-Valentine leukocidin	[23,47]	12	40.0
*lukM+lukF-P83*	Phage-borne LukM/F-P83 leukocidin, associated with disease in ungulates	[23,47]	0	0.0
*lukD+lukE*	Genomic-Island-borne leukocidin	[23,47]	21	70.0
*tst1*	Toxic shock syndrome toxin	[23,47]	2	6.7
*sea*	Gene encoding enterotoxin A	[23,47]	13	43.3
*sea*_(N315)_=*sep*	Allele of the enterotoxin A gene, frequently found in CC5 and CC7	[23,47]	0	0.0
*seb*	Gene encoding enterotoxin B	[23,47]	2	6,7
*sec*, *see*, *sel*	Genes encoding enterotoxins C, E and L	[23,47]	0	0.0
*sed*	Gene encoding enterotoxin D	[23,47]	1	3.3
*seh*	Gene encoding enterotoxin H, associated, e.g., with CC1, CC10 and CC34	[23,47]	9	30.0
*sej*	Gene encoding enterotoxin J	[23,47]	1	3.3
*sek*	Gene encoding enterotoxin K	[23,47]	12	40.0
*seq*	Gene encoding enterotoxin Q	[23,47]	12	40.0
*ser*	Gene encoding enterotoxin R	[23,47]	1	3.3
*egc*	Enterotoxin gene cluster consisting of *seg*, *sei*, *selm*, *seln*, *selo* and *selu*	[23,47]	6	20.0
ORF CM14	Enterotoxin gene homologue, associated, e.g., with CCs 93, 121 and 705	[23,47]	1	3.3
*sak*	Staphylokinase	[23,47]	24	80.0
*chp*	Chemotaxis-inhibiting protein (CHIPS)	[23,47]	9	30.0
*scn*	Staphylococcal complement inhibitor	[23,47]	29	96.7
*etA*, *etB*, *etD*	Genes encoding Exfoliative Toxins A, B. D	[23,47]	0	0.0
*etD2/etE/etE2*	Exfoliative Toxin homologue *	[51,52]	2	6.7
*edinA*	Epidermal cell differentiation inhibitor	[23,47]	1	3.3
*edinB*	Epidermal cell differentiation inhibitor B	[23,47]	2	6.7
*edinC*	Epidermal cell differentiation inhibitor C	[23,47]	0	0.0
*cap* 5	Capsule type 5 (summary of probes for *capH*5, *capJ*5, *capK*5)	[23,47]	10	33.3
*cap* 8	Capsule type 8 (summary of probes for *capH*8, *capI*8, *capJ*8, *capK*8)	[23,47]	20	66.7
*cna*	Gene encoding collagen adhesion factor	[23,47]	18	60.0
*sasG*	*Staphylococcus aureus surface* protein G	[23,47]	26	86.7
*sasX=sesI*	Surface-anchored protein X, used for subtyping CC239-MRSA-III clades	[52]	0	0.0
*agr I*	Accessory gene regulator, group (variant/allele) 1	[23,47]	8	26.7
*agr II*	Accessory gene regulator, group (variant/allele) 2	[23,47]	11	36.7
*agr III*	Accessory gene regulator, group (variant/allele) 3	[23,47]	10	33.3
*agr IV*	Accessory gene regulator, group (variant/allele) 4	[23,47]	1	3.3
*hld*	Haemolysin Delta, small peptide whose gene is located next to *agr*	[23,47]	30	100.0
*tirS*	Staphylococcal TIR-protein binding protein	[52]	11	36.7
*arcA+B+D-*SCC	Genes encoding the arginine metabolic operon from ACME-1/-2 elements	[23,47]	0	0.0

* Weak and/or irregular signals (one out of two probes) attributable to the presence of a novel *etD/E* homologue in CC152 strains, as recently recognised in [53].

**Table 5:** Affiliations to CCs and strains, comparison to the data from the 2015 study [44].

Clonal Complex	Strain	n in 2020	% in 2020	n in 2015	% in 2015
CC1	CC1-MRSA-[V+*fus*+*tir*+ccrA/B-1]	3	10.0	2	4.3
	CC1-MRSA-[V+*fus*+*tir*+ccrA/B-1] (PVL+)	5	16.7	4	8.7
	CC1-MRSA-[V+*fus*+*tir*]	1	3.3	0	0
CC5	CC5-MRSA-[V+cas], “WA MRSA-123”	0	0.0	2	4.3
	CC5-MRSA-[VI+*fus*+*tir*]	2	6.7	1	2.2
CC6	CC6-MRSA-IVa, “WA MRSA-51”	0	0.0	1	2.2
	CC6-MRSA-[V+*fus*]	1	3.3	0	0
CC15	CC15-MRSA-[V+*fus*]	6	20.0	0	0
CC22	CC22-MRSA-IVa (tst1+), “Gaza Epidemic Strain”	2	6.7	3	6.5
CC30	CC30-MRSA-IVa (PVL+), “WSPP/Southwest Pacific Clone”	1	3.3	0	0
CC80	CC80-MRSA-IVc	0	0.0	1	2.2
	CC80-MRSA-IVc (PVL+)	0	0.0	3	6.5
CC88	CC88-MRSA-IV	0	0.0	1	2.2
CC97	CC97-MRSA-IVc, “WA MRSA-54/63”	0	0.0	3	6.5
	CC97-MRSA-V	0	0.0	1	2.2
	CC97-MRSA-[V+*fus*]	1	3.3	2	4.3
CC121	CC121-MRSA-[V+*fus*] (PVL+)	1	3.3	0	0
CC152	CC152-MRSA-[V+*fus*] (PVL+)	2	6.7	1	2.2
CC239	CC239-MRSA-[III+Cd/Hg+*ccrC*] (*sasX*-positive), “Southeast Asian Clade”	0	0.0	1	2.2
	CC239-MRSA-[III+Cd+*ccrC*] (*sasX*-negative), “Middle Eastern Cluster”	1	3.3	18	39.1
	CC239-MRSA-[III+Cd] (*sasX*-negative)	1	3.3	2	4.3
CC1153	CC1153-MRSA-[V+*fus*] (PVL+)	3	10.0	0	0

**Table 6:** PCR primers used.

Primer	Sequence	Tm (°C)	Amplicon Size (bp)	Reference
*femA*_FWR_	CTTACTTACTGCTGTACCTG	58	686	[100,101]
*femA*_REV_	ATCTCGCTTGTTATGTGC	56		
*mecA*_FWR_	TGGCTATCGTGTCACAATCG	58.08	304	[100,102]
*mecA*_REV_	CTGGAACTTGTTGAGCAGAG	56.3		

## Data Availability

All relevant data are provided as supplementary files. The genome sequence, including the SCC*mec* element discussed, can be accessed under BioSample accession number SAMN31868372 and GenBank accession number CP113244.1.

## Supplementary Materials

The following supporting information can be downloaded at: https://www.mdpi.com/article/10.3390/antibiotics12010078/s1, Table S1: Array hybridisation profiles (pdf); File S2a: Genome sequence of strain Alexandria_2020-19 (fasta); File S2b: Genes identified in the SCCmec element of strain Alexandria_2020-19 (fasta).
