# Supplementary material for: Characterisation of Methicillin-Resistant Staphylococcus aureus from Alexandria, Egypt
Source: Antibiotics (Basel). 2023 Jan 1;12(1):78. doi: 10.3390/antibiotics12010078 (PMC9855118; doi:10.3390/antibiotics12010078)
Supplement: Supplementary file 1 [file antibiotics-12-00078-s001.zip › Supplement S1_Hybridisation patterns.pdf]

Isolate	Strain assignment	SPECIES MARKER								REGULATORY GENES		
		rrnD1	gapA	katA	CoA	nuc1	spa	sbi	crtN	sarA	saeS	vraS
		Domain 1 of 23S-rRNA	glyceraldehyde 3-phosphate dehydro-genase, locus 1	katalase A	coagulase	thermostable extracellular nuclease	staphylococcal protein A	IgG-binding protein	dehydrosqualene desaturase	staphylococcal accessory regulator A	histidine protein kinase, sae locus	sensor protein
**CC1**												
Alexandria University_2020-17	CC1-MRSA-[V+fus+tir]	POS	POS	POS	POS	POS	POS	POS	POS	POS	POS	POS
Alexandria University_2020-03	CC1-MRSA-[V+fus+tir+ccrAB1]	POS	POS	POS	POS	POS	POS	POS	POS	POS	POS	POS
Alexandria University_2020-19	CC1-MRSA-[V+fus+tir+ccrAB1]	POS	POS	POS	POS	POS	POS	POS	POS	POS	POS	POS
Alexandria University_2020-21	CC1-MRSA-[V+fus+tir+ccrAB1]	POS	POS	POS	POS	POS	POS	POS	POS	POS	POS	POS
Alexandria University_2020-09	CC1-MRSA-[V+fus+tir+ccrAB1] (PVL+)	POS	POS	POS	POS	POS	POS	POS	POS	POS	POS	POS
Alexandria University_2020-14	CC1-MRSA-[V+fus+tir+ccrAB1] (PVL+)	POS	POS	POS	POS	POS	POS	POS	POS	POS	POS	POS
Alexandria University_2020-24	CC1-MRSA-[V+fus+tir+ccrAB1] (PVL+)	POS	POS	POS	POS	POS	POS	POS	POS	POS	POS	POS
Alexandria University_2020-27	CC1-MRSA-[V+fus+tir+ccrAB1] (PVL+)	POS	POS	POS	POS	POS	POS	POS	POS	POS	POS	POS
Alexandria University_2020-28	CC1-MRSA-[V+fus+tir+ccrAB1] (PVL+)	POS	POS	POS	POS	POS	POS	POS	POS	POS	POS	POS
**CC5**												
Alexandria University_2020-12	CC5-MRSA-[VI+fus+tir]	POS	POS	POS	POS	POS	POS	POS	POS	POS	POS	POS
Alexandria University_2020-20	CC5-MRSA-[VI+fus+tir]	POS	POS	POS	POS	POS	POS	POS	POS	POS	POS	POS
**CC6**												
Alexandria University_2020-29	CC6-MRSA-[V+fus]	POS	POS	POS	POS	POS	POS	POS	POS	POS	POS	POS
**CC15**												
Alexandria University_2020-01	CC15-MRSA-[V+fus]	POS	POS	POS	POS	POS	POS	POS	POS	POS	POS	POS
Alexandria University_2020-02	CC15-MRSA-[V+fus]	POS	POS	POS	POS	POS	POS	POS	POS	POS	POS	POS
Alexandria University_2020-05	CC15-MRSA-[V+fus]	POS	POS	POS	POS	POS	POS	POS	POS	POS	POS	POS
Alexandria University_2020-15	CC15-MRSA-[V+fus]	POS	POS	POS	POS	POS	POS	POS	POS	POS	POS	POS
Alexandria University_2020-22	CC15-MRSA-[V+fus]	POS	POS	POS	POS	POS	POS	POS	POS	POS	POS	POS
Alexandria University_2020-32	CC15-MRSA-[V+fus]	POS	POS	POS	POS	POS	POS	POS	POS	POS	POS	POS
**CC22**												
Alexandria University_2020-04	CC22-MRSA-IVa (dcs negatives) (tst1+), "Gaza Epidemic Strain"	POS	POS	POS	POS	POS	POS	POS	POS	POS	POS	POS
Alexandria University_2020-11	CC22-MRSA-IVa (dcs negatives) (tst1+), "Gaza Epidemic Strain"	POS	POS	POS	POS	POS	POS	POS	POS	POS	POS	POS
**CC30**												
Alexandria University_2020-26	CC30-MRSA-IVa (PVL+), "WSPP/Southwest Pacific Clone"	POS	POS	POS	POS	POS	POS	POS	POS	POS	POS	POS
**CC97**												
Alexandria University_2020-30	CC97-MRSA-[V+fus]	POS	POS	POS	POS	POS	POS	POS	POS	POS	POS	POS
**CC121**												
Alexandria University_2020-06	CC121-MRSA-[V+fus] (PVL+)	POS	POS	POS	POS	POS	POS	POS	POS	POS	POS	POS
**CC152**												
Alexandria University_2020-18	CC152-MRSA-[V+fus]	POS	POS	POS	POS	POS	POS	POS	NEG	POS	POS	POS
Alexandria University_2020-31	CC152-MRSA-[V+fus]	POS	POS	POS	POS	POS	POS	AMB	NEG	POS	POS	POS
**CC239**												
Alexandria University_2020-13	CC239-MRSA-[III+Cd+ccrC] (sasX-negat.), "Middle Eastern Cluster"	POS	POS	POS	POS	POS	POS	POS	POS	POS	POS	POS
Alexandria University_2020-16	CC239-MRSA-[III+Cd], sasX-negative	POS	POS	POS	POS	POS	POS	POS	POS	POS	POS	POS
**CC1153**												
Alexandria University_2020-07	CC1153-MRSA-[V+fus] (PVL+)	POS	AMB	POS	AMB	POS	POS	POS	POS	POS	POS	POS
Alexandria University_2020-08	CC1153-MRSA-[V+fus] (PVL+)	POS	POS	POS	POS	POS	POS	POS	POS	POS	POS	POS
Alexandria University_2020-23	CC1153-MRSA-[V+fus] (PVL+)	POS	POS	POS	POS	POS	POS	POS	POS	POS	POS	POS
**Simulated hybridisations for sequences from Montelongo et al., 2022**												
**CC1**												
JAEOUR Staphylococcus aureus strain AA1	CC1-MRSA-[V+fus+tir+ccrAB1]	POS	POS	POS	POS	POS	POS	POS	POS	POS	POS	POS
JAEOWK Staphylococcus aureus strain AA78	CC1-MRSA-[V+fus+tir+ccrAB1]	POS	POS	POS	POS	POS	POS	POS	POS	POS	POS	POS
JAEOVR Staphylococcus aureus strain AA51	CC1-MRSA-[V+fus+tir+ccrAB1] (PVL+)	POS	POS	POS	POS	POS	POS	POS	POS	POS	POS	POS
JAEOWE Staphylococcus aureus strain AA67	CC1-MRSA-[V+fus+tir+ccrAB1] (PVL+)	POS	POS	POS	POS	POS	POS	POS	POS	POS	POS	POS
JAEOWG Staphylococcus aureus strain AA69	CC1-MRSA-[V+fus+tir+ccrAB1] (PVL+)	POS	POS	POS	POS	POS	POS	POS	POS	POS	POS	POS
JAEOWJ Staphylococcus aureus strain AA77	CC1-MRSA-[V+fus+tir+ccrAB1] (PVL+)	POS	POS	POS	POS	POS	POS	POS	POS	POS	POS	POS
**CC5**												
JAEOWI Staphylococcus aureus strain AA76	CC5-MRSA-[V+cas], WA MRSA-123	POS	POS	POS	POS	POS	POS	POS	POS	POS	POS	POS
JAEOWN Staphylococcus aureus strain AA80	CC5-MRSA-[V+cas], WA MRSA-123	POS	POS	POS	POS	POS	POS	POS	POS	POS	POS	POS
JAEOWH Staphylococcus aureus strain AA70	CC5-MRSA-[VI+fus+tir+, dcs-]	POS	POS	POS	POS	POS	POS	POS	POS	POS	POS	POS
**CC6**												
JAEOVF Staphylococcus aureus strain AA30	CC6-MRSA-IVa, WA MRSA-51	POS	POS	POS	POS	POS	POS	POS	AMB	POS	POS	POS
**CC80**												
JAEOVM Staphylococcus aureus strain AA4	CC80-MRSA-IVc (PVL-) [aphA3/sat+]:[far1+], contaminated	POS	POS	POS	POS	POS	POS	POS	POS	POS	POS	POS
JAEOUZ Staphylococcus aureus strain AA2	CC80-MRSA-IVc (PVL+) [aphA3/sat+]:[far1+]	POS	POS	POS	POS	POS	POS	POS	POS	POS	POS	POS
JAEOVE Staphylococcus aureus strain AA3	CC80-MRSA-IVc (PVL+) [aphA3/sat+]:[far1+]	POS	POS	POS	POS	POS	POS	POS	POS	POS	POS	POS
JAEOVO Staphylococcus aureus strain AA45	CC80-MRSA-IVc (PVL+) [aphA3/sat+]:[far1+]	POS	POS	POS	POS	POS	POS	POS	POS	POS	POS	POS
**CC88**												
JAEOVT Staphylococcus aureus strain AA53	CC88-MRSA-IV, contaminated	POS	POS	POS	POS	POS	POS	POS	AMB	POS	POS	POS
**CC97**												
JAEOVL Staphylococcus aureus strain AA39	CC97-MRSA-IVc, WA MRSA-54/63	POS	POS	POS	POS	POS	POS	POS	POS	POS	POS	POS
JAEOVX Staphylococcus aureus strain AA6	CC97-MRSA-IVc, WA MRSA-54/63	POS	POS	POS	POS	POS	POS	POS	POS	POS	POS	POS
JAEOWM Staphylococcus aureus strain AA8	CC97-MRSA-IVc, WA MRSA-54/63	POS	POS	POS	POS	POS	POS	POS	POS	POS	POS	POS
JAEOUU Staphylococcus aureus strain AA104	CC97-MRSA-V	POS	POS	POS	POS	POS	POS	POS	POS	POS	POS	POS
JAEOVJ Staphylococcus aureus strain AA35	CC97-MRSA-[V+fus]	POS	POS	POS	POS	POS	POS	POS	POS	POS	POS	POS
JAEOVK Staphylococcus aureus strain AA36	CC97-MRSA-[V+fus]	POS	POS	POS	POS	POS	POS	POS	POS	POS	POS	POS
**CC22**												
JAEOUY Staphylococcus aureus strain AA18	CC22-MRSA-IVa (dcs negatives) (tst1+), "Gaza Epidemic Strain"	POS	POS	POS	POS	POS	POS	POS	POS	POS	POS	POS
JAEOVH Staphylococcus aureus strain AA32	CC22-MRSA-IVa (dcs negatives) (tst1+), "Gaza Epidemic Strain"	POS	POS	POS	POS	POS	POS	POS	POS	POS	POS	POS
JAEOVQ Staphylococcus aureus strain AA5	CC22-MRSA-IVa (dcs negatives) (tst1+), "Gaza Epidemic Strain"	POS	POS	POS	POS	POS	POS	POS	POS	POS	POS	POS
**CC152**												
JAEOUX Staphylococcus aureus strain AA17	CC152-MRSA-[V+fus]	POS	POS	POS	POS	POS	AMB	POS	NEG	POS	POS	POS
**CC239**												
JAEOVI Staphylococcus aureus strain AA33	CC239-MRSA-[III+Cd/Hg+ccrC] (sasX-positive), "Southeast Asian Clade"	POS	POS	POS	POS	POS	POS	POS	POS	POS	POS	POS
JAEOUS Staphylococcus aureus strain AA101	CC239-MRSA-[III+Cd+ccrC] (sasX-negat.), "Middle Eastern Cluster"	POS	POS	POS	POS	POS	POS	POS	POS	POS	POS	POS
JAEOUV Staphylococcus aureus strain AA13	CC239-MRSA-[III+Cd+ccrC] (sasX-negat.), "Middle Eastern Cluster"	POS	POS	POS	POS	POS	POS	POS	POS	POS	POS	POS
JAEOUW Staphylococcus aureus strain AA14	CC239-MRSA-[III+Cd+ccrC] (sasX-negat.), "Middle Eastern Cluster"	POS	POS	POS	POS	POS	POS	POS	POS	POS	POS	POS
JAEOVA Staphylococcus aureus strain AA22	CC239-MRSA-[III+Cd+ccrC] (sasX-negat.), "Middle Eastern Cluster"	POS	POS	POS	POS	POS	POS	POS	POS	POS	POS	POS
JAEOVB Staphylococcus aureus strain AA23	CC239-MRSA-[III+Cd+ccrC] (sasX-negat.), "Middle Eastern Cluster"	POS	POS	POS	POS	POS	POS	POS	POS	POS	POS	POS
JAEOVC Staphylococcus aureus strain AA27	CC239-MRSA-[III+Cd+ccrC] (sasX-negat.), "Middle Eastern Cluster"	POS	POS	POS	POS	POS	POS	POS	POS	POS	POS	POS
JAEOVD Staphylococcus aureus strain AA29	CC239-MRSA-[III+Cd+ccrC] (sasX-negat.), "Middle Eastern Cluster"	POS	POS	POS	POS	POS	POS	POS	POS	POS	POS	POS
JAEOVG Staphylococcus aureus strain AA31	CC239-MRSA-[III+Cd+ccrC] (sasX-negat.), "Middle Eastern Cluster"	POS	POS	POS	POS	POS	POS	POS	POS	POS	POS	POS
JAEOVP Staphylococcus aureus strain AA46	CC239-MRSA-[III+Cd+ccrC] (sasX-negat.), "Middle Eastern Cluster"	POS	POS	POS	POS	POS	POS	POS	POS	POS	POS	POS
JAEOVS Staphylococcus aureus strain AA52	CC239-MRSA-[III+Cd+ccrC] (sasX-negat.), "Middle Eastern Cluster"	POS	POS	POS	POS	POS	POS	POS	POS	POS	POS	POS
JAEOVU Staphylococcus aureus strain AA55	CC239-MRSA-[III+Cd+ccrC] (sasX-negat.), "Middle Eastern Cluster"	POS	POS	POS	POS	POS	POS	POS	POS	POS	POS	POS
JAEOVV Staphylococcus aureus strain AA57	CC239-MRSA-[III+Cd+ccrC] (sasX-negat.), "Middle Eastern Cluster"	POS	POS	POS	POS	POS	POS	POS	POS	POS	POS	POS
JAEOVY Staphylococcus aureus strain AA60	CC239-MRSA-[III+Cd+ccrC] (sasX-negat.), "Middle Eastern Cluster"	POS	POS	POS	POS	POS	POS	POS	POS	POS	POS	POS
JAEOVZ Staphylococcus aureus strain AA61	CC239-MRSA-[III+Cd+ccrC] (sasX-negat.), "Middle Eastern Cluster"	POS	POS	POS	POS	POS	POS	POS	POS	POS	POS	POS
JAEOWA Staphylococcus aureus strain AA62	CC239-MRSA-[III+Cd+ccrC] (sasX-negat.), "Middle Eastern Cluster"	POS	POS	POS	POS	POS	POS	POS	POS	POS	POS	POS
JAEOWB Staphylococcus aureus strain AA63	CC239-MRSA-[III+Cd+ccrC] (sasX-negat.), "Middle Eastern Cluster"	POS	POS	POS	POS	POS	POS	POS	POS	POS	POS	POS
JAEOWL Staphylococcus aureus strain AA79	CC239-MRSA-[III+Cd+ccrC] (sasX-negat.), "Middle Eastern Cluster"	POS	POS	POS	POS	POS	POS	POS	POS	POS	POS	POS
JAEOWQ Staphylococcus aureus strain AA92	CC239-MRSA-[III+Cd+ccrC] (sasX-negat.), "Middle Eastern Cluster"	POS	POS	POS	POS	POS	POS	POS	POS	POS	POS	POS
JAEOWC Staphylococcus aureus strain AA64	CC239-MRSA-[mec III+Cd], sasX-negative	POS	POS	POS	POS	POS	POS	POS	POS	POS	POS	POS
JAEOWP Staphylococcus aureus strain AA91	CC239-MRSA-[mec III+Cd], sasX-negative	POS	POS	POS	POS	POS	POS	POS	POS	POS	POS	POS

Isolate	Strain assignment	REGULATORY GENES													
		agrI			agrII				agrIII				agrIV		hld
		agrI (total)	agrC-I	agrD-I	agrII (total)	agrB-II	agrC-II	agrD-II	agrIII (total)	agrB-III	agrC-III	agrD-III	agrIV (total)	agrC-IV	
		accessory gene regulator allele I			accessory gene regulator allele II				accessory gene regulator allele III				accessory gene regulator allele IV		haemolysin delta
**CC1**															
Alexandria University_2020-17	CC1-MRSA-[V+fus+tir]	NEG	NEG	NEG	NEG	NEG	NEG	NEG	POS	POS	AMB	POS	NEG	NEG	POS
Alexandria University_2020-03	CC1-MRSA-[V+fus+tir+ccrAB1]	NEG	NEG	NEG	NEG	NEG	NEG	NEG	POS	POS	POS	POS	NEG	NEG	POS
Alexandria University_2020-19	CC1-MRSA-[V+fus+tir+ccrAB1]	NEG	NEG	NEG	NEG	NEG	NEG	NEG	POS	POS	AMB	POS	NEG	NEG	POS
Alexandria University_2020-21	CC1-MRSA-[V+fus+tir+ccrAB1]	NEG	NEG	NEG	NEG	NEG	NEG	NEG	POS	POS	POS	POS	NEG	NEG	POS
Alexandria University_2020-09	CC1-MRSA-[V+fus+tir+ccrAB1] (PVL+)	NEG	NEG	NEG	NEG	NEG	NEG	NEG	POS	POS	AMB	POS	NEG	NEG	POS
Alexandria University_2020-14	CC1-MRSA-[V+fus+tir+ccrAB1] (PVL+)	NEG	NEG	NEG	NEG	NEG	NEG	NEG	POS	POS	POS	POS	NEG	NEG	POS
Alexandria University_2020-24	CC1-MRSA-[V+fus+tir+ccrAB1] (PVL+)	NEG	NEG	NEG	NEG	NEG	NEG	NEG	POS	POS	POS	POS	NEG	NEG	POS
Alexandria University_2020-27	CC1-MRSA-[V+fus+tir+ccrAB1] (PVL+)	NEG	NEG	NEG	NEG	NEG	NEG	NEG	POS	POS	POS	POS	NEG	NEG	POS
Alexandria University_2020-28	CC1-MRSA-[V+fus+tir+ccrAB1] (PVL+)	NEG	NEG	NEG	NEG	NEG	NEG	NEG	POS	POS	NEG	POS	NEG	NEG	POS
**CC5**															
Alexandria University_2020-12	CC5-MRSA-[VI+fus+tir]	NEG	NEG	NEG	POS	POS	POS	POS	NEG	NEG	NEG	NEG	NEG	NEG	POS
Alexandria University_2020-20	CC5-MRSA-[VI+fus+tir]	NEG	NEG	NEG	POS	POS	POS	POS	NEG	NEG	NEG	NEG	NEG	NEG	POS
**CC6**															
Alexandria University_2020-29	CC6-MRSA-[V+fus]	POS	POS	POS	NEG	NEG	NEG	NEG	NEG	NEG	NEG	NEG	NEG	NEG	POS
**CC15**															
Alexandria University_2020-01	CC15-MRSA-[V+fus]	NEG	NEG	NEG	POS	POS	POS	POS	NEG	NEG	NEG	NEG	NEG	NEG	POS
Alexandria University_2020-02	CC15-MRSA-[V+fus]	NEG	NEG	NEG	POS	POS	POS	POS	NEG	NEG	NEG	NEG	NEG	NEG	POS
Alexandria University_2020-05	CC15-MRSA-[V+fus]	NEG	NEG	NEG	POS	POS	POS	POS	NEG	NEG	NEG	NEG	NEG	NEG	POS
Alexandria University_2020-15	CC15-MRSA-[V+fus]	NEG	NEG	NEG	POS	POS	POS	POS	NEG	NEG	NEG	NEG	NEG	NEG	POS
Alexandria University_2020-22	CC15-MRSA-[V+fus]	NEG	NEG	NEG	POS	POS	POS	POS	NEG	NEG	NEG	NEG	NEG	NEG	POS
Alexandria University_2020-32	CC15-MRSA-[V+fus]	NEG	NEG	NEG	POS	POS	POS	POS	NEG	NEG	NEG	NEG	NEG	NEG	POS
**CC22**															
Alexandria University_2020-04	CC22-MRSA-IVa (dcs negatives) (tst1+), "Gaza Epidemic Strain"	POS	AMB	POS	NEG	NEG	NEG	NEG	NEG	NEG	NEG	NEG	NEG	NEG	POS
Alexandria University_2020-11	CC22-MRSA-IVa (dcs negatives) (tst1+), "Gaza Epidemic Strain"	POS	POS	POS	NEG	NEG	NEG	NEG	NEG	NEG	NEG	NEG	NEG	NEG	POS
**CC30**															
Alexandria University_2020-26	CC30-MRSA-IVa (PVL+), "WSPP/Southwest Pacific Clone"	NEG	NEG	NEG	NEG	NEG	NEG	NEG	POS	POS	POS	POS	NEG	NEG	POS
**CC97**															
Alexandria University_2020-30	CC97-MRSA-[V+fus]	POS	POS	POS	NEG	NEG	NEG	NEG	NEG	NEG	NEG	NEG	NEG	NEG	POS
**CC121**															
Alexandria University_2020-06	CC121-MRSA-[V+fus] (PVL+)	NEG	POS	NEG	NEG	NEG	NEG	NEG	NEG	NEG	NEG	NEG	POS	POS	POS
**CC152**															
Alexandria University_2020-18	CC152-MRSA-[V+fus]	POS	POS	POS	NEG	NEG	NEG	NEG	NEG	NEG	NEG	NEG	NEG	NEG	POS
Alexandria University_2020-31	CC152-MRSA-[V+fus]	POS	POS	POS	NEG	NEG	NEG	NEG	NEG	NEG	NEG	NEG	NEG	NEG	POS
**CC239**															
Alexandria University_2020-13	CC239-MRSA-[III+Cd+ccrC] (sasX-negat.), "Middle Eastern Cluster"	POS	POS	POS	NEG	NEG	NEG	NEG	NEG	NEG	NEG	NEG	NEG	NEG	POS
Alexandria University_2020-16	CC239-MRSA-[III+Cd], sasX-negative	POS	POS	POS	NEG	NEG	NEG	NEG	NEG	NEG	NEG	NEG	NEG	NEG	POS
**CC1153**															
Alexandria University_2020-07	CC1153-MRSA-[V+fus] (PVL+)	NEG	NEG	NEG	POS	POS	AMB	POS	NEG	NEG	NEG	NEG	NEG	NEG	POS
Alexandria University_2020-08	CC1153-MRSA-[V+fus] (PVL+)	NEG	NEG	NEG	POS	POS	POS	POS	NEG	NEG	NEG	NEG	NEG	NEG	POS
Alexandria University_2020-23	CC1153-MRSA-[V+fus] (PVL+)	NEG	NEG	NEG	POS	POS	POS	POS	NEG	NEG	NEG	NEG	NEG	NEG	POS
**Simulated hybridisations for sequences from Montelongo et al., 2022**															
**CC1**															
JAEOUR Staphylococcus aureus strain AA1	CC1-MRSA-[V+fus+tir+ccrAB1]	NEG	NEG	NEG	NEG	NEG	NEG	NEG	POS	POS	AMB	POS	NEG	NEG	POS
JAEOWK Staphylococcus aureus strain AA78	CC1-MRSA-[V+fus+tir+ccrAB1]	NEG	NEG	NEG	NEG	NEG	NEG	NEG	POS	POS	AMB	POS	NEG	NEG	POS
JAEOVR Staphylococcus aureus strain AA51	CC1-MRSA-[V+fus+tir+ccrAB1] (PVL+)	NEG	NEG	NEG	NEG	NEG	NEG	NEG	POS	POS	AMB	POS	NEG	NEG	POS
JAEOWE Staphylococcus aureus strain AA67	CC1-MRSA-[V+fus+tir+ccrAB1] (PVL+)	NEG	NEG	NEG	NEG	NEG	NEG	NEG	POS	POS	AMB	POS	NEG	NEG	POS
JAEOWG Staphylococcus aureus strain AA69	CC1-MRSA-[V+fus+tir+ccrAB1] (PVL+)	NEG	NEG	NEG	NEG	NEG	NEG	NEG	POS	POS	AMB	POS	NEG	NEG	POS
JAEOWJ Staphylococcus aureus strain AA77	CC1-MRSA-[V+fus+tir+ccrAB1] (PVL+)	NEG	NEG	NEG	NEG	NEG	NEG	NEG	POS	POS	AMB	POS	NEG	NEG	POS
**CC5**															
JAEOWI Staphylococcus aureus strain AA76	CC5-MRSA-[V+cas], WA MRSA-123	NEG	NEG	NEG	POS	POS	POS	POS	NEG	NEG	NEG	NEG	NEG	NEG	POS
JAEOWN Staphylococcus aureus strain AA80	CC5-MRSA-[V+cas], WA MRSA-123	NEG	NEG	NEG	POS	POS	POS	POS	NEG	NEG	NEG	NEG	NEG	NEG	POS
JAEOWH Staphylococcus aureus strain AA70	CC5-MRSA-[VI+fus+tir+, dcs-]	NEG	NEG	NEG	POS	POS	POS	POS	NEG	NEG	NEG	NEG	NEG	NEG	POS
**CC6**															
JAEOVF Staphylococcus aureus strain AA30	CC6-MRSA-IVa, WA MRSA-51	POS	POS	AMB	NEG	NEG	NEG	NEG	NEG	NEG	NEG	NEG	NEG	NEG	POS
**CC80**															
JAEOVM Staphylococcus aureus strain AA4	CC80-MRSA-IVc (PVL-) [aphA3/sat+]:[far1+], contaminated	AMB	POS	AMB	NEG	NEG	NEG	NEG	POS	POS	POS	POS	NEG	NEG	POS
JAEOUZ Staphylococcus aureus strain AA2	CC80-MRSA-IVc (PVL+) [aphA3/sat+]:[far1+]	NEG	NEG	NEG	NEG	NEG	NEG	NEG	POS	POS	POS	POS	NEG	NEG	POS
JAEOVE Staphylococcus aureus strain AA3	CC80-MRSA-IVc (PVL+) [aphA3/sat+]:[far1+]	NEG	NEG	NEG	NEG	NEG	NEG	NEG	POS	POS	POS	POS	NEG	NEG	POS
JAEOVO Staphylococcus aureus strain AA45	CC80-MRSA-IVc (PVL+) [aphA3/sat+]:[far1+]	NEG	NEG	NEG	NEG	NEG	NEG	NEG	POS	POS	POS	POS	NEG	NEG	POS
**CC88**															
JAEOVT Staphylococcus aureus strain AA53	CC88-MRSA-IV, contaminated	AMB	POS	AMB	NEG	NEG	NEG	NEG	POS	POS	POS	POS	NEG	NEG	POS
**CC97**															
JAEOVL Staphylococcus aureus strain AA39	CC97-MRSA-IVc, WA MRSA-54/63	POS	POS	AMB	NEG	NEG	NEG	NEG	NEG	NEG	NEG	NEG	NEG	NEG	POS
JAEOVX Staphylococcus aureus strain AA6	CC97-MRSA-IVc, WA MRSA-54/63	POS	POS	AMB	NEG	NEG	NEG	NEG	NEG	NEG	NEG	NEG	NEG	NEG	POS
JAEOWM Staphylococcus aureus strain AA8	CC97-MRSA-IVc, WA MRSA-54/63	POS	POS	AMB	NEG	NEG	NEG	NEG	NEG	NEG	NEG	NEG	NEG	NEG	POS
JAEOUU Staphylococcus aureus strain AA104	CC97-MRSA-V	POS	POS	AMB	NEG	NEG	NEG	NEG	NEG	NEG	NEG	NEG	NEG	NEG	POS
JAEOVJ Staphylococcus aureus strain AA35	CC97-MRSA-[V+fus]	POS	POS	AMB	NEG	NEG	NEG	NEG	NEG	NEG	NEG	NEG	NEG	NEG	POS
JAEOVK Staphylococcus aureus strain AA36	CC97-MRSA-[V+fus]	POS	POS	AMB	NEG	NEG	NEG	NEG	NEG	NEG	NEG	NEG	NEG	NEG	POS
**CC22**															
JAEOUY Staphylococcus aureus strain AA18	CC22-MRSA-IVa (dcs negatives) (tst1+), "Gaza Epidemic Strain"	POS	POS	AMB	NEG	NEG	NEG	NEG	NEG	NEG	NEG	NEG	NEG	NEG	POS
JAEOVH Staphylococcus aureus strain AA32	CC22-MRSA-IVa (dcs negatives) (tst1+), "Gaza Epidemic Strain"	POS	POS	AMB	NEG	NEG	NEG	NEG	NEG	NEG	NEG	NEG	NEG	NEG	POS
JAEOVQ Staphylococcus aureus strain AA5	CC22-MRSA-IVa (dcs negatives) (tst1+), "Gaza Epidemic Strain"	POS	POS	AMB	NEG	NEG	NEG	NEG	NEG	NEG	NEG	NEG	NEG	NEG	POS
**CC152**															
JAEOUX Staphylococcus aureus strain AA17	CC152-MRSA-[V+fus]	AMB	POS	AMB	NEG	NEG	NEG	NEG	NEG	NEG	NEG	NEG	NEG	NEG	POS
**CC239**															
JAEOVI Staphylococcus aureus strain AA33	CC239-MRSA-[III+Cd/Hg+ccrC] (sasX-positive), "Southeast Asian Clade"	POS	POS	AMB	NEG	NEG	NEG	NEG	NEG	NEG	NEG	NEG	NEG	NEG	POS
JAEOUS Staphylococcus aureus strain AA101	CC239-MRSA-[III+Cd+ccrC] (sasX-negat.), "Middle Eastern Cluster"	POS	POS	AMB	NEG	NEG	NEG	NEG	NEG	NEG	NEG	NEG	NEG	NEG	POS
JAEOUV Staphylococcus aureus strain AA13	CC239-MRSA-[III+Cd+ccrC] (sasX-negat.), "Middle Eastern Cluster"	POS	POS	AMB	NEG	NEG	NEG	NEG	NEG	NEG	NEG	NEG	NEG	NEG	POS
JAEOUW Staphylococcus aureus strain AA14	CC239-MRSA-[III+Cd+ccrC] (sasX-negat.), "Middle Eastern Cluster"	POS	POS	AMB	NEG	NEG	NEG	NEG	NEG	NEG	NEG	NEG	NEG	NEG	POS
JAEOVA Staphylococcus aureus strain AA22	CC239-MRSA-[III+Cd+ccrC] (sasX-negat.), "Middle Eastern Cluster"	POS	POS	AMB	NEG	NEG	NEG	NEG	NEG	NEG	NEG	NEG	NEG	NEG	POS
JAEOVB Staphylococcus aureus strain AA23	CC239-MRSA-[III+Cd+ccrC] (sasX-negat.), "Middle Eastern Cluster"	POS	POS	AMB	NEG	NEG	NEG	NEG	NEG	NEG	NEG	NEG	NEG	NEG	POS
JAEOVC Staphylococcus aureus strain AA27	CC239-MRSA-[III+Cd+ccrC] (sasX-negat.), "Middle Eastern Cluster"	POS	POS	AMB	NEG	NEG	NEG	NEG	NEG	NEG	NEG	NEG	NEG	NEG	POS
JAEOVD Staphylococcus aureus strain AA29	CC239-MRSA-[III+Cd+ccrC] (sasX-negat.), "Middle Eastern Cluster"	POS	POS	AMB	NEG	NEG	NEG	NEG	NEG	NEG	NEG	NEG	NEG	NEG	POS
JAEOVG Staphylococcus aureus strain AA31	CC239-MRSA-[III+Cd+ccrC] (sasX-negat.), "Middle Eastern Cluster"	POS	POS	AMB	NEG	NEG	NEG	NEG	NEG	NEG	NEG	NEG	NEG	NEG	POS
JAEOVP Staphylococcus aureus strain AA46	CC239-MRSA-[III+Cd+ccrC] (sasX-negat.), "Middle Eastern Cluster"	POS	POS	AMB	NEG	NEG	NEG	NEG	NEG	NEG	NEG	NEG	NEG	NEG	POS
JAEOVS Staphylococcus aureus strain AA52	CC239-MRSA-[III+Cd+ccrC] (sasX-negat.), "Middle Eastern Cluster"	POS	POS	AMB	NEG	NEG	NEG	NEG	NEG	NEG	NEG	NEG	NEG	NEG	POS
JAEOVU Staphylococcus aureus strain AA55	CC239-MRSA-[III+Cd+ccrC] (sasX-negat.), "Middle Eastern Cluster"	POS	POS	AMB	NEG	NEG	NEG	NEG	NEG	NEG	NEG	NEG	NEG	NEG	POS
JAEOVV Staphylococcus aureus strain AA57	CC239-MRSA-[III+Cd+ccrC] (sasX-negat.), "Middle Eastern Cluster"	POS	POS	AMB	NEG	NEG	NEG	NEG	NEG	NEG	NEG	NEG	NEG	NEG	POS
JAEOVY Staphylococcus aureus strain AA60	CC239-MRSA-[III+Cd+ccrC] (sasX-negat.), "Middle Eastern Cluster"	POS	POS	AMB	NEG	NEG	NEG	NEG	NEG	NEG	NEG	NEG	NEG	NEG	POS
JAEOVZ Staphylococcus aureus strain AA61	CC239-MRSA-[III+Cd+ccrC] (sasX-negat.), "Middle Eastern Cluster"	POS	POS	AMB	NEG	NEG	NEG	NEG	NEG	NEG	NEG	NEG	NEG	NEG	POS
JAEOWA Staphylococcus aureus strain AA62	CC239-MRSA-[III+Cd+ccrC] (sasX-negat.), "Middle Eastern Cluster"	POS	POS	AMB	NEG	NEG	NEG	NEG	NEG	NEG	NEG	NEG	NEG	NEG	POS
JAEOWB Staphylococcus aureus strain AA63	CC239-MRSA-[III+Cd+ccrC] (sasX-negat.), "Middle Eastern Cluster"	POS	POS	AMB	NEG	NEG	NEG	NEG	NEG	NEG	NEG	NEG	NEG	NEG	POS
JAEOWL Staphylococcus aureus strain AA79	CC239-MRSA-[III+Cd+ccrC] (sasX-negat.), "Middle Eastern Cluster"	POS	POS	AMB	NEG	NEG	NEG	NEG	NEG	NEG	NEG	NEG	NEG	NEG	POS
JAEOWQ Staphylococcus aureus strain AA92	CC239-MRSA-[III+Cd+ccrC] (sasX-negat.), "Middle Eastern Cluster"	POS	POS	AMB	NEG	NEG	NEG	NEG	NEG	NEG	NEG	NEG	NEG	NEG	POS
JAEOWC Staphylococcus aureus strain AA64	CC239-MRSA-[mec III+Cd], sasX-negative	POS	POS	AMB	NEG	NEG	NEG	NEG	NEG	NEG	NEG	NEG	NEG	NEG	POS
JAEOWP Staphylococcus aureus strain AA91	CC239-MRSA-[mec III+Cd], sasX-negative	POS	POS	AMB	NEG	NEG	NEG	NEG	NEG	NEG	NEG	NEG	NEG	NEG	POS

Isolate	Strain assignment	METHICILLIN RESISTANCE AND SCCmec TYPING													
		ugpQ	mecA	Delta mecR1	mecR	mecI	xylR/mecR2	mecC	blaZ (SCCmec XI)	pls-SCC (COL)	cstB-SCC2 (Q2G1R6)	kdpA-SCC	kdpB-SCC	kdpD-SCC	D1GU38
		Glycerophosphoryl diester phosphodiesterase	Modified penicillin binding protein (PBP2a)	Truncated methicillin resistance operon repressor 1.	Methicillin resistance operon repressor 1.	Methicillin-resistance regulatory protein	Methicillin resistance operon repressor 2	Alternate gene encoding a modified penicillin binding protein	Beta-lactamase from SCCmec XI	Plasmin-sensitive surface protein	CsoR-like sulfur transferase-regulated gene	Potassium-translocating ATPase A, chain 2	Potassium-transporting ATPase B, chain 1	Sensor kinase protein	Putative protein.
**CC1**															
Alexandria University_2020-17	CC1-MRSA-[V+fus+tir]	POS	POS	NEG	NEG	NEG	NEG	NEG	NEG	NEG	NEG	NEG	NEG	NEG	NEG
Alexandria University_2020-03	CC1-MRSA-[V+fus+tir+ccrAB1]	POS	POS	NEG	NEG	NEG	NEG	NEG	NEG	NEG	NEG	NEG	NEG	NEG	NEG
Alexandria University_2020-19	CC1-MRSA-[V+fus+tir+ccrAB1]	POS	POS	NEG	NEG	NEG	NEG	NEG	NEG	NEG	NEG	NEG	NEG	NEG	NEG
Alexandria University_2020-21	CC1-MRSA-[V+fus+tir+ccrAB1]	POS	POS	NEG	NEG	NEG	NEG	NEG	NEG	NEG	NEG	NEG	NEG	NEG	NEG
Alexandria University_2020-09	CC1-MRSA-[V+fus+tir+ccrAB1] (PVL+)	POS	POS	NEG	NEG	NEG	NEG	NEG	NEG	NEG	NEG	NEG	NEG	NEG	NEG
Alexandria University_2020-14	CC1-MRSA-[V+fus+tir+ccrAB1] (PVL+)	POS	POS	NEG	NEG	NEG	NEG	NEG	NEG	NEG	NEG	NEG	NEG	NEG	NEG
Alexandria University_2020-24	CC1-MRSA-[V+fus+tir+ccrAB1] (PVL+)	POS	POS	NEG	NEG	NEG	NEG	NEG	NEG	NEG	NEG	NEG	NEG	NEG	NEG
Alexandria University_2020-27	CC1-MRSA-[V+fus+tir+ccrAB1] (PVL+)	POS	POS	NEG	NEG	NEG	NEG	NEG	NEG	NEG	NEG	NEG	NEG	NEG	NEG
Alexandria University_2020-28	CC1-MRSA-[V+fus+tir+ccrAB1] (PVL+)	POS	POS	NEG	NEG	NEG	NEG	NEG	NEG	NEG	NEG	NEG	NEG	NEG	NEG
**CC5**															
Alexandria University_2020-12	CC5-MRSA-[VI+fus+tir]	POS	POS	POS	NEG	NEG	NEG	NEG	NEG	NEG	NEG	NEG	NEG	NEG	NEG
Alexandria University_2020-20	CC5-MRSA-[VI+fus+tir]	POS	POS	POS	NEG	NEG	NEG	NEG	NEG	NEG	NEG	NEG	NEG	NEG	NEG
**CC6**															
Alexandria University_2020-29	CC6-MRSA-[V+fus]	POS	POS	NEG	NEG	NEG	NEG	NEG	NEG	NEG	NEG	NEG	NEG	NEG	NEG
**CC15**															
Alexandria University_2020-01	CC15-MRSA-[V+fus]	POS	POS	NEG	NEG	NEG	NEG	NEG	NEG	NEG	NEG	NEG	NEG	NEG	NEG
Alexandria University_2020-02	CC15-MRSA-[V+fus]	POS	POS	NEG	NEG	NEG	NEG	NEG	NEG	NEG	NEG	NEG	NEG	NEG	NEG
Alexandria University_2020-05	CC15-MRSA-[V+fus]	POS	POS	NEG	NEG	NEG	NEG	NEG	NEG	NEG	NEG	NEG	NEG	NEG	NEG
Alexandria University_2020-15	CC15-MRSA-[V+fus]	POS	POS	NEG	NEG	NEG	NEG	NEG	NEG	NEG	NEG	NEG	NEG	NEG	NEG
Alexandria University_2020-22	CC15-MRSA-[V+fus]	POS	POS	NEG	NEG	NEG	NEG	NEG	NEG	NEG	NEG	NEG	NEG	NEG	NEG
Alexandria University_2020-32	CC15-MRSA-[V+fus]	POS	POS	NEG	NEG	NEG	NEG	NEG	NEG	NEG	NEG	NEG	NEG	NEG	NEG
**CC22**															
Alexandria University_2020-04	CC22-MRSA-IVa (dcs negatives) (tst1+), "Gaza Epidemic Strain"	POS	POS	POS	NEG	NEG	NEG	NEG	NEG	NEG	POS	NEG	NEG	NEG	NEG
Alexandria University_2020-11	CC22-MRSA-IVa (dcs negatives) (tst1+), "Gaza Epidemic Strain"	POS	POS	POS	NEG	NEG	NEG	NEG	NEG	NEG	POS	NEG	NEG	NEG	NEG
**CC30**															
Alexandria University_2020-26	CC30-MRSA-IVa (PVL+), "WSPP/Southwest Pacific Clone"	POS	POS	POS	NEG	NEG	NEG	NEG	NEG	NEG	POS	NEG	NEG	NEG	NEG
**CC97**															
Alexandria University_2020-30	CC97-MRSA-[V+fus]	POS	POS	NEG	NEG	NEG	NEG	NEG	NEG	NEG	NEG	NEG	NEG	NEG	NEG
**CC121**															
Alexandria University_2020-06	CC121-MRSA-[V+fus] (PVL+)	POS	POS	NEG	NEG	NEG	NEG	NEG	NEG	NEG	NEG	NEG	NEG	NEG	NEG
**CC152**															
Alexandria University_2020-18	CC152-MRSA-[V+fus]	POS	POS	NEG	NEG	NEG	NEG	NEG	NEG	NEG	NEG	NEG	NEG	NEG	NEG
Alexandria University_2020-31	CC152-MRSA-[V+fus]	POS	POS	NEG	NEG	NEG	NEG	NEG	NEG	NEG	NEG	NEG	NEG	NEG	NEG
**CC239**															
Alexandria University_2020-13	CC239-MRSA-[III+Cd+ccrC] (sasX-negat.), "Middle Eastern Cluster"	POS	POS	POS	POS	POS	POS	NEG	NEG	NEG	NEG	NEG	NEG	NEG	NEG
Alexandria University_2020-16	CC239-MRSA-[III+Cd], sasX-negative	POS	POS	POS	POS	POS	POS	NEG	NEG	NEG	NEG	NEG	NEG	NEG	NEG
**CC1153**															
Alexandria University_2020-07	CC1153-MRSA-[V+fus] (PVL+)	POS	POS	NEG	NEG	NEG	NEG	NEG	NEG	NEG	NEG	NEG	NEG	NEG	NEG
Alexandria University_2020-08	CC1153-MRSA-[V+fus] (PVL+)	POS	POS	NEG	NEG	NEG	NEG	NEG	NEG	NEG	NEG	NEG	NEG	NEG	NEG
Alexandria University_2020-23	CC1153-MRSA-[V+fus] (PVL+)	POS	POS	NEG	NEG	NEG	NEG	NEG	NEG	NEG	NEG	NEG	NEG	NEG	NEG
**Simulated hybridisations for sequences from Montelongo et al., 2022**															
**CC1**															
JAEOUR Staphylococcus aureus strain AA1	CC1-MRSA-[V+fus+tir+ccrAB1]	POS	POS	NEG	NEG	NEG	NEG	NEG	NEG	NEG	NEG	NEG	NEG	NEG	NEG
JAEOWK Staphylococcus aureus strain AA78	CC1-MRSA-[V+fus+tir+ccrAB1]	POS	POS	NEG	NEG	NEG	NEG	NEG	NEG	NEG	NEG	NEG	NEG	NEG	NEG
JAEOVR Staphylococcus aureus strain AA51	CC1-MRSA-[V+fus+tir+ccrAB1] (PVL+)	POS	POS	NEG	NEG	NEG	NEG	NEG	NEG	NEG	NEG	NEG	NEG	NEG	NEG
JAEOWE Staphylococcus aureus strain AA67	CC1-MRSA-[V+fus+tir+ccrAB1] (PVL+)	POS	POS	NEG	NEG	NEG	NEG	NEG	NEG	NEG	NEG	NEG	NEG	NEG	NEG
JAEOWG Staphylococcus aureus strain AA69	CC1-MRSA-[V+fus+tir+ccrAB1] (PVL+)	POS	POS	NEG	NEG	NEG	NEG	NEG	NEG	NEG	NEG	NEG	NEG	NEG	NEG
JAEOWJ Staphylococcus aureus strain AA77	CC1-MRSA-[V+fus+tir+ccrAB1] (PVL+)	POS	POS	NEG	NEG	NEG	NEG	NEG	NEG	NEG	NEG	NEG	NEG	NEG	NEG
**CC5**															
JAEOWI Staphylococcus aureus strain AA76	CC5-MRSA-[V+cas], WA MRSA-123	POS	POS	NEG	NEG	NEG	NEG	NEG	NEG	NEG	NEG	NEG	NEG	NEG	NEG
JAEOWN Staphylococcus aureus strain AA80	CC5-MRSA-[V+cas], WA MRSA-123	POS	POS	NEG	NEG	NEG	NEG	NEG	NEG	NEG	NEG	NEG	NEG	NEG	NEG
JAEOWH Staphylococcus aureus strain AA70	CC5-MRSA-[VI+fus+tir+, dcs-]	POS	POS	POS	NEG	NEG	NEG	NEG	NEG	NEG	NEG	NEG	NEG	NEG	NEG
**CC6**															
JAEOVF Staphylococcus aureus strain AA30	CC6-MRSA-IVa, WA MRSA-51	POS	POS	POS	NEG	NEG	NEG	NEG	NEG	NEG	POS	NEG	NEG	NEG	NEG
**CC80**															
JAEOVM Staphylococcus aureus strain AA4	CC80-MRSA-IVc (PVL-) [aphA3/sat+]:[far1+], contaminated	POS	POS	POS	NEG	NEG	NEG	NEG	NEG	NEG	NEG	NEG	NEG	NEG	NEG
JAEOUZ Staphylococcus aureus strain AA2	CC80-MRSA-IVc (PVL+) [aphA3/sat+]:[far1+]	POS	POS	POS	NEG	NEG	NEG	NEG	NEG	NEG	NEG	NEG	NEG	NEG	NEG
JAEOVE Staphylococcus aureus strain AA3	CC80-MRSA-IVc (PVL+) [aphA3/sat+]:[far1+]	POS	POS	POS	NEG	NEG	NEG	NEG	NEG	NEG	NEG	NEG	NEG	NEG	NEG
JAEOVO Staphylococcus aureus strain AA45	CC80-MRSA-IVc (PVL+) [aphA3/sat+]:[far1+]	POS	POS	POS	NEG	NEG	NEG	NEG	NEG	NEG	NEG	NEG	NEG	NEG	NEG
**CC88**															
JAEOVT Staphylococcus aureus strain AA53	CC88-MRSA-IV, contaminated	POS	POS	POS	NEG	NEG	NEG	NEG	NEG	NEG	POS	NEG	NEG	NEG	NEG
**CC97**															
JAEOVL Staphylococcus aureus strain AA39	CC97-MRSA-IVc, WA MRSA-54/63	POS	POS	POS	NEG	NEG	NEG	NEG	NEG	NEG	NEG	NEG	NEG	NEG	NEG
JAEOVX Staphylococcus aureus strain AA6	CC97-MRSA-IVc, WA MRSA-54/63	POS	POS	POS	NEG	NEG	NEG	NEG	NEG	NEG	NEG	NEG	NEG	NEG	NEG
JAEOWM Staphylococcus aureus strain AA8	CC97-MRSA-IVc, WA MRSA-54/63	POS	POS	POS	NEG	NEG	NEG	NEG	NEG	NEG	NEG	NEG	NEG	NEG	NEG
JAEOUU Staphylococcus aureus strain AA104	CC97-MRSA-V	POS	POS	NEG	NEG	NEG	NEG	NEG	NEG	NEG	NEG	NEG	NEG	NEG	NEG
JAEOVJ Staphylococcus aureus strain AA35	CC97-MRSA-[V+fus]	POS	POS	NEG	NEG	NEG	NEG	NEG	NEG	NEG	NEG	NEG	NEG	NEG	NEG
JAEOVK Staphylococcus aureus strain AA36	CC97-MRSA-[V+fus]	POS	POS	NEG	NEG	NEG	NEG	NEG	NEG	NEG	NEG	NEG	NEG	NEG	NEG
**CC22**															
JAEOUY Staphylococcus aureus strain AA18	CC22-MRSA-IVa (dcs negatives) (tst1+), "Gaza Epidemic Strain"	POS	POS	POS	NEG	NEG	NEG	NEG	NEG	NEG	POS	NEG	NEG	NEG	NEG
JAEOVH Staphylococcus aureus strain AA32	CC22-MRSA-IVa (dcs negatives) (tst1+), "Gaza Epidemic Strain"	POS	POS	POS	NEG	NEG	NEG	NEG	NEG	NEG	POS	NEG	NEG	NEG	NEG
JAEOVQ Staphylococcus aureus strain AA5	CC22-MRSA-IVa (dcs negatives) (tst1+), "Gaza Epidemic Strain"	POS	POS	POS	NEG	NEG	NEG	NEG	NEG	NEG	POS	NEG	NEG	NEG	NEG
**CC152**															
JAEOUX Staphylococcus aureus strain AA17	CC152-MRSA-[V+fus]	POS	POS	NEG	NEG	NEG	NEG	NEG	NEG	NEG	NEG	NEG	NEG	NEG	NEG
**CC239**															
JAEOVI Staphylococcus aureus strain AA33	CC239-MRSA-[III+Cd/Hg+ccrC] (sasX-positive), "Southeast Asian Clade"	POS	POS	POS	POS	POS	POS	NEG	NEG	NEG	NEG	NEG	NEG	NEG	POS
JAEOUS Staphylococcus aureus strain AA101	CC239-MRSA-[III+Cd+ccrC] (sasX-negat.), "Middle Eastern Cluster"	POS	POS	POS	POS	POS	POS	NEG	NEG	NEG	NEG	NEG	NEG	NEG	NEG
JAEOUV Staphylococcus aureus strain AA13	CC239-MRSA-[III+Cd+ccrC] (sasX-negat.), "Middle Eastern Cluster"	POS	POS	POS	POS	POS	POS	NEG	NEG	NEG	NEG	NEG	NEG	NEG	NEG
JAEOUW Staphylococcus aureus strain AA14	CC239-MRSA-[III+Cd+ccrC] (sasX-negat.), "Middle Eastern Cluster"	POS	POS	POS	POS	POS	POS	NEG	NEG	NEG	NEG	NEG	NEG	NEG	NEG
JAEOVA Staphylococcus aureus strain AA22	CC239-MRSA-[III+Cd+ccrC] (sasX-negat.), "Middle Eastern Cluster"	POS	POS	POS	POS	POS	POS	NEG	NEG	NEG	NEG	NEG	NEG	NEG	NEG
JAEOVB Staphylococcus aureus strain AA23	CC239-MRSA-[III+Cd+ccrC] (sasX-negat.), "Middle Eastern Cluster"	POS	POS	POS	POS	POS	POS	NEG	NEG	NEG	NEG	NEG	NEG	NEG	NEG
JAEOVC Staphylococcus aureus strain AA27	CC239-MRSA-[III+Cd+ccrC] (sasX-negat.), "Middle Eastern Cluster"	POS	POS	POS	POS	POS	POS	NEG	NEG	NEG	NEG	NEG	NEG	NEG	NEG
JAEOVD Staphylococcus aureus strain AA29	CC239-MRSA-[III+Cd+ccrC] (sasX-negat.), "Middle Eastern Cluster"	POS	POS	POS	POS	POS	POS	NEG	NEG	NEG	NEG	NEG	NEG	NEG	NEG
JAEOVG Staphylococcus aureus strain AA31	CC239-MRSA-[III+Cd+ccrC] (sasX-negat.), "Middle Eastern Cluster"	POS	POS	POS	POS	POS	POS	NEG	NEG	NEG	NEG	NEG	NEG	NEG	NEG
JAEOVP Staphylococcus aureus strain AA46	CC239-MRSA-[III+Cd+ccrC] (sasX-negat.), "Middle Eastern Cluster"	POS	POS	POS	POS	POS	POS	NEG	NEG	NEG	NEG	NEG	NEG	NEG	NEG
JAEOVS Staphylococcus aureus strain AA52	CC239-MRSA-[III+Cd+ccrC] (sasX-negat.), "Middle Eastern Cluster"	POS	POS	POS	POS	POS	POS	NEG	NEG	NEG	NEG	NEG	NEG	NEG	NEG
JAEOVU Staphylococcus aureus strain AA55	CC239-MRSA-[III+Cd+ccrC] (sasX-negat.), "Middle Eastern Cluster"	POS	POS	POS	POS	POS	POS	NEG	NEG	NEG	NEG	NEG	NEG	NEG	NEG
JAEOVV Staphylococcus aureus strain AA57	CC239-MRSA-[III+Cd+ccrC] (sasX-negat.), "Middle Eastern Cluster"	POS	POS	POS	POS	POS	POS	NEG	NEG	NEG	NEG	NEG	NEG	NEG	NEG
JAEOVY Staphylococcus aureus strain AA60	CC239-MRSA-[III+Cd+ccrC] (sasX-negat.), "Middle Eastern Cluster"	POS	POS	POS	POS	POS	POS	NEG	NEG	NEG	NEG	NEG	NEG	NEG	NEG
JAEOVZ Staphylococcus aureus strain AA61	CC239-MRSA-[III+Cd+ccrC] (sasX-negat.), "Middle Eastern Cluster"	POS	POS	POS	POS	POS	POS	NEG	NEG	NEG	NEG	NEG	NEG	NEG	NEG
JAEOWA Staphylococcus aureus strain AA62	CC239-MRSA-[III+Cd+ccrC] (sasX-negat.), "Middle Eastern Cluster"	POS	POS	POS	POS	POS	POS	NEG	NEG	NEG	NEG	NEG	NEG	NEG	NEG
JAEOWB Staphylococcus aureus strain AA63	CC239-MRSA-[III+Cd+ccrC] (sasX-negat.), "Middle Eastern Cluster"	POS	POS	POS	POS	POS	POS	NEG	NEG	NEG	NEG	NEG	NEG	NEG	NEG
JAEOWL Staphylococcus aureus strain AA79	CC239-MRSA-[III+Cd+ccrC] (sasX-negat.), "Middle Eastern Cluster"	POS	POS	POS	POS	POS	POS	NEG	NEG	NEG	NEG	NEG	NEG	NEG	NEG
JAEOWQ Staphylococcus aureus strain AA92	CC239-MRSA-[III+Cd+ccrC] (sasX-negat.), "Middle Eastern Cluster"	POS	POS	POS	POS	POS	POS	NEG	NEG	NEG	NEG	NEG	NEG	NEG	NEG
JAEOWC Staphylococcus aureus strain AA64	CC239-MRSA-[mec III+Cd], sasX-negative	POS	POS	POS	POS	POS	POS	NEG	NEG	NEG	NEG	NEG	NEG	NEG	NEG
JAEOWP Staphylococcus aureus strain AA91	CC239-MRSA-[mec III+Cd], sasX-negative	POS	POS	POS	POS	POS	POS	NEG	NEG	NEG	NEG	NEG	NEG	NEG	NEG

Isolate	Strain assignment	METHICILLIN RESISTANCE AND SCCmec TYPING										
		B2Y834	B6VQU0	Q3YK51	fusC (Q6GD50)	tirS	ACME total	arcA-SCC	arcB-SCC	arcC-SCC	opp3B	speG
		Abortive phage resistance protein.	Putative protein	Putative protein.	SCC-associated fusidic acid resistance gene	Staphylococcal TIR protein binding protein	ACME total	Arginine deiminase	Ornithine carbamoyltransferase	Carbamate kinase	Oligopeptide permease, channel-forming protein	Spermidine N-acetyltransferase from ACME
**CC1**												
Alexandria University_2020-17	CC1-MRSA-[V+fus+tir]	NEG	NEG	NEG	POS	POS	NEG	NEG	NEG	NEG	NEG	NEG
Alexandria University_2020-03	CC1-MRSA-[V+fus+tir+ccrAB1]	NEG	NEG	NEG	POS	POS	NEG	NEG	NEG	NEG	NEG	NEG
Alexandria University_2020-19	CC1-MRSA-[V+fus+tir+ccrAB1]	NEG	NEG	NEG	POS	POS	NEG	NEG	NEG	NEG	NEG	NEG
Alexandria University_2020-21	CC1-MRSA-[V+fus+tir+ccrAB1]	NEG	NEG	NEG	POS	POS	NEG	NEG	NEG	NEG	NEG	NEG
Alexandria University_2020-09	CC1-MRSA-[V+fus+tir+ccrAB1] (PVL+)	NEG	NEG	NEG	POS	POS	NEG	NEG	NEG	NEG	NEG	NEG
Alexandria University_2020-14	CC1-MRSA-[V+fus+tir+ccrAB1] (PVL+)	NEG	NEG	NEG	POS	POS	NEG	NEG	NEG	NEG	NEG	NEG
Alexandria University_2020-24	CC1-MRSA-[V+fus+tir+ccrAB1] (PVL+)	NEG	NEG	NEG	POS	POS	NEG	NEG	NEG	NEG	NEG	NEG
Alexandria University_2020-27	CC1-MRSA-[V+fus+tir+ccrAB1] (PVL+)	NEG	NEG	NEG	POS	POS	NEG	NEG	NEG	NEG	NEG	NEG
Alexandria University_2020-28	CC1-MRSA-[V+fus+tir+ccrAB1] (PVL+)	NEG	NEG	NEG	POS	POS	NEG	NEG	NEG	NEG	NEG	NEG
**CC5**												
Alexandria University_2020-12	CC5-MRSA-[VI+fus+tir]	NEG	NEG	NEG	POS	POS	NEG	NEG	NEG	NEG	NEG	NEG
Alexandria University_2020-20	CC5-MRSA-[VI+fus+tir]	NEG	NEG	NEG	POS	POS	NEG	NEG	NEG	NEG	NEG	NEG
**CC6**												
Alexandria University_2020-29	CC6-MRSA-[V+fus]	NEG	NEG	NEG	POS	NEG	NEG	NEG	NEG	NEG	NEG	NEG
**CC15**												
Alexandria University_2020-01	CC15-MRSA-[V+fus]	NEG	NEG	NEG	POS	NEG	NEG	NEG	NEG	NEG	NEG	NEG
Alexandria University_2020-02	CC15-MRSA-[V+fus]	NEG	NEG	NEG	POS	NEG	NEG	NEG	NEG	NEG	NEG	NEG
Alexandria University_2020-05	CC15-MRSA-[V+fus]	NEG	NEG	NEG	POS	NEG	NEG	NEG	NEG	NEG	NEG	NEG
Alexandria University_2020-15	CC15-MRSA-[V+fus]	NEG	NEG	NEG	POS	NEG	NEG	NEG	NEG	NEG	NEG	NEG
Alexandria University_2020-22	CC15-MRSA-[V+fus]	NEG	NEG	NEG	POS	NEG	NEG	NEG	NEG	NEG	NEG	NEG
Alexandria University_2020-32	CC15-MRSA-[V+fus]	NEG	NEG	NEG	POS	NEG	NEG	NEG	NEG	NEG	NEG	NEG
**CC22**												
Alexandria University_2020-04	CC22-MRSA-IVa (dcs negatives) (tst1+), "Gaza Epidemic Strain"	NEG	NEG	NEG	NEG	NEG	NEG	NEG	NEG	NEG	NEG	NEG
Alexandria University_2020-11	CC22-MRSA-IVa (dcs negatives) (tst1+), "Gaza Epidemic Strain"	NEG	NEG	NEG	NEG	NEG	NEG	NEG	NEG	NEG	NEG	NEG
**CC30**												
Alexandria University_2020-26	CC30-MRSA-IVa (PVL+), "WSPP/Southwest Pacific Clone"	NEG	NEG	NEG	NEG	NEG	NEG	NEG	NEG	NEG	NEG	NEG
**CC97**												
Alexandria University_2020-30	CC97-MRSA-[V+fus]	NEG	NEG	NEG	POS	NEG	NEG	NEG	NEG	NEG	NEG	NEG
**CC121**												
Alexandria University_2020-06	CC121-MRSA-[V+fus] (PVL+)	NEG	NEG	NEG	POS	NEG	NEG	NEG	NEG	NEG	NEG	NEG
**CC152**												
Alexandria University_2020-18	CC152-MRSA-[V+fus]	NEG	NEG	NEG	POS	NEG	NEG	NEG	NEG	NEG	NEG	NEG
Alexandria University_2020-31	CC152-MRSA-[V+fus]	NEG	NEG	NEG	POS	NEG	NEG	NEG	NEG	NEG	NEG	NEG
**CC239**												
Alexandria University_2020-13	CC239-MRSA-[III+Cd+ccrC] (sasX-negat.), "Middle Eastern Cluster"	NEG	NEG	NEG	NEG	NEG	NEG	NEG	NEG	NEG	NEG	NEG
Alexandria University_2020-16	CC239-MRSA-[III+Cd], sasX-negative	NEG	NEG	NEG	NEG	NEG	NEG	NEG	NEG	NEG	NEG	NEG
**CC1153**												
Alexandria University_2020-07	CC1153-MRSA-[V+fus] (PVL+)	NEG	NEG	NEG	POS	NEG	NEG	NEG	NEG	NEG	NEG	NEG
Alexandria University_2020-08	CC1153-MRSA-[V+fus] (PVL+)	NEG	NEG	NEG	POS	NEG	NEG	NEG	NEG	NEG	NEG	NEG
Alexandria University_2020-23	CC1153-MRSA-[V+fus] (PVL+)	NEG	NEG	NEG	POS	NEG	NEG	NEG	NEG	NEG	NEG	NEG
**Simulated hybridisations for sequences from Montelongo et al., 2022**												
**CC1**												
JAEOUR Staphylococcus aureus strain AA1	CC1-MRSA-[V+fus+tir+ccrAB1]	NEG	NEG	NEG	POS	POS	NEG	NEG	NEG	NEG	NEG	NEG
JAEOWK Staphylococcus aureus strain AA78	CC1-MRSA-[V+fus+tir+ccrAB1]	NEG	NEG	NEG	POS	POS	NEG	NEG	NEG	NEG	NEG	NEG
JAEOVR Staphylococcus aureus strain AA51	CC1-MRSA-[V+fus+tir+ccrAB1] (PVL+)	NEG	NEG	NEG	POS	POS	NEG	NEG	NEG	NEG	NEG	NEG
JAEOWE Staphylococcus aureus strain AA67	CC1-MRSA-[V+fus+tir+ccrAB1] (PVL+)	NEG	NEG	NEG	POS	POS	NEG	NEG	NEG	NEG	NEG	NEG
JAEOWG Staphylococcus aureus strain AA69	CC1-MRSA-[V+fus+tir+ccrAB1] (PVL+)	NEG	NEG	NEG	POS	POS	NEG	NEG	NEG	NEG	NEG	NEG
JAEOWJ Staphylococcus aureus strain AA77	CC1-MRSA-[V+fus+tir+ccrAB1] (PVL+)	NEG	NEG	NEG	POS	POS	NEG	NEG	NEG	NEG	NEG	NEG
**CC5**												
JAEOWI Staphylococcus aureus strain AA76	CC5-MRSA-[V+cas], WA MRSA-123	NEG	NEG	NEG	NEG	NEG	NEG	NEG	NEG	NEG	NEG	NEG
JAEOWN Staphylococcus aureus strain AA80	CC5-MRSA-[V+cas], WA MRSA-123	NEG	NEG	NEG	NEG	NEG	NEG	NEG	NEG	NEG	NEG	NEG
JAEOWH Staphylococcus aureus strain AA70	CC5-MRSA-[VI+fus+tir+, dcs-]	NEG	NEG	NEG	POS	POS	NEG	NEG	NEG	NEG	NEG	NEG
**CC6**												
JAEOVF Staphylococcus aureus strain AA30	CC6-MRSA-IVa, WA MRSA-51	NEG	NEG	NEG	NEG	NEG	NEG	NEG	NEG	NEG	NEG	NEG
**CC80**												
JAEOVM Staphylococcus aureus strain AA4	CC80-MRSA-IVc (PVL-) [aphA3/sat+]:[far1+], contaminated	POS	NEG	NEG	NEG	NEG	NEG	NEG	NEG	NEG	NEG	NEG
JAEOUZ Staphylococcus aureus strain AA2	CC80-MRSA-IVc (PVL+) [aphA3/sat+]:[far1+]	POS	NEG	NEG	NEG	NEG	NEG	NEG	NEG	NEG	NEG	NEG
JAEOVE Staphylococcus aureus strain AA3	CC80-MRSA-IVc (PVL+) [aphA3/sat+]:[far1+]	POS	NEG	NEG	NEG	NEG	NEG	NEG	NEG	NEG	NEG	NEG
JAEOVO Staphylococcus aureus strain AA45	CC80-MRSA-IVc (PVL+) [aphA3/sat+]:[far1+]	POS	NEG	NEG	NEG	NEG	NEG	NEG	NEG	NEG	NEG	NEG
**CC88**												
JAEOVT Staphylococcus aureus strain AA53	CC88-MRSA-IV, contaminated	NEG	NEG	NEG	POS	NEG	NEG	NEG	NEG	NEG	NEG	NEG
**CC97**												
JAEOVL Staphylococcus aureus strain AA39	CC97-MRSA-IVc, WA MRSA-54/63	POS	NEG	NEG	NEG	NEG	NEG	NEG	NEG	NEG	NEG	NEG
JAEOVX Staphylococcus aureus strain AA6	CC97-MRSA-IVc, WA MRSA-54/63	POS	NEG	NEG	NEG	NEG	NEG	NEG	NEG	NEG	NEG	NEG
JAEOWM Staphylococcus aureus strain AA8	CC97-MRSA-IVc, WA MRSA-54/63	POS	NEG	NEG	NEG	NEG	NEG	NEG	NEG	NEG	NEG	NEG
JAEOUU Staphylococcus aureus strain AA104	CC97-MRSA-V	NEG	NEG	NEG	NEG	NEG	NEG	NEG	NEG	NEG	NEG	NEG
JAEOVJ Staphylococcus aureus strain AA35	CC97-MRSA-[V+fus]	NEG	NEG	NEG	POS	NEG	NEG	NEG	NEG	NEG	NEG	NEG
JAEOVK Staphylococcus aureus strain AA36	CC97-MRSA-[V+fus]	NEG	NEG	NEG	POS	NEG	NEG	NEG	NEG	NEG	NEG	NEG
**CC22**												
JAEOUY Staphylococcus aureus strain AA18	CC22-MRSA-IVa (dcs negatives) (tst1+), "Gaza Epidemic Strain"	NEG	NEG	NEG	NEG	NEG	NEG	NEG	NEG	NEG	NEG	NEG
JAEOVH Staphylococcus aureus strain AA32	CC22-MRSA-IVa (dcs negatives) (tst1+), "Gaza Epidemic Strain"	NEG	NEG	NEG	NEG	NEG	NEG	NEG	NEG	NEG	NEG	NEG
JAEOVQ Staphylococcus aureus strain AA5	CC22-MRSA-IVa (dcs negatives) (tst1+), "Gaza Epidemic Strain"	NEG	NEG	NEG	NEG	NEG	NEG	NEG	NEG	NEG	NEG	NEG
**CC152**												
JAEOUX Staphylococcus aureus strain AA17	CC152-MRSA-[V+fus]	NEG	NEG	NEG	POS	NEG	NEG	NEG	NEG	NEG	NEG	NEG
**CC239**												
JAEOVI Staphylococcus aureus strain AA33	CC239-MRSA-[III+Cd/Hg+ccrC] (sasX-positive), "Southeast Asian Clade"	NEG	NEG	NEG	NEG	NEG	NEG	NEG	NEG	NEG	NEG	NEG
JAEOUS Staphylococcus aureus strain AA101	CC239-MRSA-[III+Cd+ccrC] (sasX-negat.), "Middle Eastern Cluster"	NEG	NEG	NEG	NEG	NEG	NEG	NEG	NEG	NEG	NEG	NEG
JAEOUV Staphylococcus aureus strain AA13	CC239-MRSA-[III+Cd+ccrC] (sasX-negat.), "Middle Eastern Cluster"	NEG	NEG	NEG	NEG	NEG	NEG	NEG	NEG	NEG	NEG	NEG
JAEOUW Staphylococcus aureus strain AA14	CC239-MRSA-[III+Cd+ccrC] (sasX-negat.), "Middle Eastern Cluster"	NEG	NEG	NEG	NEG	NEG	NEG	NEG	NEG	NEG	NEG	NEG
JAEOVA Staphylococcus aureus strain AA22	CC239-MRSA-[III+Cd+ccrC] (sasX-negat.), "Middle Eastern Cluster"	NEG	NEG	NEG	NEG	NEG	NEG	NEG	NEG	NEG	NEG	NEG
JAEOVB Staphylococcus aureus strain AA23	CC239-MRSA-[III+Cd+ccrC] (sasX-negat.), "Middle Eastern Cluster"	NEG	NEG	NEG	NEG	NEG	NEG	NEG	NEG	NEG	NEG	NEG
JAEOVC Staphylococcus aureus strain AA27	CC239-MRSA-[III+Cd+ccrC] (sasX-negat.), "Middle Eastern Cluster"	NEG	NEG	NEG	NEG	NEG	NEG	NEG	NEG	NEG	NEG	NEG
JAEOVD Staphylococcus aureus strain AA29	CC239-MRSA-[III+Cd+ccrC] (sasX-negat.), "Middle Eastern Cluster"	NEG	NEG	NEG	NEG	NEG	NEG	NEG	NEG	NEG	NEG	NEG
JAEOVG Staphylococcus aureus strain AA31	CC239-MRSA-[III+Cd+ccrC] (sasX-negat.), "Middle Eastern Cluster"	NEG	NEG	NEG	NEG	NEG	NEG	NEG	NEG	NEG	NEG	NEG
JAEOVP Staphylococcus aureus strain AA46	CC239-MRSA-[III+Cd+ccrC] (sasX-negat.), "Middle Eastern Cluster"	NEG	NEG	NEG	NEG	NEG	NEG	NEG	NEG	NEG	NEG	NEG
JAEOVS Staphylococcus aureus strain AA52	CC239-MRSA-[III+Cd+ccrC] (sasX-negat.), "Middle Eastern Cluster"	NEG	NEG	NEG	NEG	NEG	NEG	NEG	NEG	NEG	NEG	NEG
JAEOVU Staphylococcus aureus strain AA55	CC239-MRSA-[III+Cd+ccrC] (sasX-negat.), "Middle Eastern Cluster"	NEG	NEG	NEG	NEG	NEG	NEG	NEG	NEG	NEG	NEG	NEG
JAEOVV Staphylococcus aureus strain AA57	CC239-MRSA-[III+Cd+ccrC] (sasX-negat.), "Middle Eastern Cluster"	NEG	NEG	NEG	NEG	NEG	NEG	NEG	NEG	NEG	NEG	NEG
JAEOVY Staphylococcus aureus strain AA60	CC239-MRSA-[III+Cd+ccrC] (sasX-negat.), "Middle Eastern Cluster"	NEG	NEG	NEG	NEG	NEG	NEG	NEG	NEG	NEG	NEG	NEG
JAEOVZ Staphylococcus aureus strain AA61	CC239-MRSA-[III+Cd+ccrC] (sasX-negat.), "Middle Eastern Cluster"	NEG	NEG	NEG	NEG	NEG	NEG	NEG	NEG	NEG	NEG	NEG
JAEOWA Staphylococcus aureus strain AA62	CC239-MRSA-[III+Cd+ccrC] (sasX-negat.), "Middle Eastern Cluster"	NEG	NEG	NEG	NEG	NEG	NEG	NEG	NEG	NEG	NEG	NEG
JAEOWB Staphylococcus aureus strain AA63	CC239-MRSA-[III+Cd+ccrC] (sasX-negat.), "Middle Eastern Cluster"	NEG	NEG	NEG	NEG	NEG	NEG	NEG	NEG	NEG	NEG	NEG
JAEOWL Staphylococcus aureus strain AA79	CC239-MRSA-[III+Cd+ccrC] (sasX-negat.), "Middle Eastern Cluster"	NEG	NEG	NEG	NEG	NEG	NEG	NEG	NEG	NEG	NEG	NEG
JAEOWQ Staphylococcus aureus strain AA92	CC239-MRSA-[III+Cd+ccrC] (sasX-negat.), "Middle Eastern Cluster"	NEG	NEG	NEG	NEG	NEG	NEG	NEG	NEG	NEG	NEG	NEG
JAEOWC Staphylococcus aureus strain AA64	CC239-MRSA-[mec III+Cd], sasX-negative	NEG	NEG	NEG	NEG	NEG	NEG	NEG	NEG	NEG	NEG	NEG
JAEOWP Staphylococcus aureus strain AA91	CC239-MRSA-[mec III+Cd], sasX-negative	NEG	NEG	NEG	NEG	NEG	NEG	NEG	NEG	NEG	NEG	NEG

Isolate	Strain assignment	METHICILLIN RESISTANCE AND SCCmec TYPING												
		ccrA-1	ccrB-01	ccrA-02	ccrB-02	ccrA-03	ccrB-03	ccrAA			ccrC	ccrA-04	ccrB-04	Q9XB68-dcs
		ccrA-1	ccrB-1	ccrA-2	ccrB-2	ccrA-3	ccrB-3	ccrAA (85-2082)	ccrAA (MRSAZH47) _probe 1	ccrAA (MRSAZH47) _probe 2	ccrC (85-2082)	ccrA-4	ccrB-4	
		Cassette chromosome recombinase A, type 1	Cassette chromosome recombinase B, type 1	Cassette chromosome recombinase A, type 2	Cassette chromosome recombinase B, type 2	Cassette chromosome recombinase A, type 3	Cassette chromosome recombinase B, type 3	"Cassette chromosome recombinase AA"			Cassette chromosome recombinase C		Cassette chromosome recombinase B, type 4	Downstream constant segment
**CC1**														
**Alexandria University_2020-17**	CC1-MRSA-[V+fus+tir]	NEG	NEG	NEG	NEG	NEG	NEG	NEG	NEG	POS	POS	NEG	NEG	NEG
**Alexandria University_2020-03**	CC1-MRSA-[V+fus+tir+ccrAB1]	POS	POS	NEG	NEG	NEG	NEG	NEG	NEG	POS	POS	NEG	NEG	NEG
**Alexandria University_2020-19**	CC1-MRSA-[V+fus+tir+ccrAB1]	POS	POS	NEG	NEG	NEG	NEG	NEG	NEG	POS	POS	NEG	NEG	NEG
**Alexandria University_2020-21**	CC1-MRSA-[V+fus+tir+ccrAB1]	POS	POS	NEG	NEG	NEG	NEG	NEG	POS	POS	POS	NEG	NEG	NEG
**Alexandria University_2020-09**	CC1-MRSA-[V+fus+tir+ccrAB1] (PVL+)	POS	POS	NEG	NEG	NEG	NEG	NEG	NEG	POS	POS	NEG	NEG	NEG
**Alexandria University_2020-14**	CC1-MRSA-[V+fus+tir+ccrAB1] (PVL+)	POS	POS	NEG	NEG	NEG	NEG	NEG	POS	POS	POS	NEG	NEG	NEG
**Alexandria University_2020-24**	CC1-MRSA-[V+fus+tir+ccrAB1] (PVL+)	POS	POS	NEG	NEG	NEG	NEG	NEG	NEG	POS	POS	NEG	NEG	NEG
**Alexandria University_2020-27**	CC1-MRSA-[V+fus+tir+ccrAB1] (PVL+)	POS	POS	NEG	NEG	NEG	NEG	NEG	NEG	POS	POS	NEG	NEG	NEG
**Alexandria University_2020-28**	CC1-MRSA-[V+fus+tir+ccrAB1] (PVL+)	POS	POS	NEG	NEG	NEG	NEG	NEG	NEG	POS	POS	NEG	NEG	NEG
**CC5**														
**Alexandria University_2020-12**	CC5-MRSA-[VI+fus+tir]	NEG	NEG	NEG	NEG	NEG	NEG	NEG	NEG	NEG	NEG	POS	POS	NEG
**Alexandria University_2020-20**	CC5-MRSA-[VI+fus+tir]	NEG	NEG	NEG	NEG	NEG	NEG	NEG	NEG	NEG	NEG	POS	POS	NEG
**CC6**														
**Alexandria University_2020-29**	CC6-MRSA-[V+fus]	NEG	NEG	NEG	NEG	NEG	NEG	NEG	NEG	POS	POS	NEG	NEG	NEG
**CC15**														
**Alexandria University_2020-01**	CC15-MRSA-[V+fus]	NEG	NEG	NEG	NEG	NEG	NEG	NEG	NEG	POS	POS	NEG	NEG	NEG
**Alexandria University_2020-02**	CC15-MRSA-[V+fus]	NEG	NEG	NEG	NEG	NEG	NEG	NEG	NEG	POS	POS	NEG	NEG	NEG
**Alexandria University_2020-05**	CC15-MRSA-[V+fus]	NEG	NEG	NEG	NEG	NEG	NEG	NEG	NEG	POS	POS	NEG	NEG	NEG
**Alexandria University_2020-15**	CC15-MRSA-[V+fus]	NEG	NEG	NEG	NEG	NEG	NEG	NEG	NEG	POS	POS	NEG	NEG	NEG
**Alexandria University_2020-22**	CC15-MRSA-[V+fus]	NEG	NEG	NEG	NEG	NEG	NEG	NEG	NEG	POS	POS	NEG	NEG	NEG
**Alexandria University_2020-32**	CC15-MRSA-[V+fus]	NEG	NEG	NEG	NEG	NEG	NEG	NEG	NEG	POS	POS	NEG	NEG	NEG
**CC22**														
**Alexandria University_2020-04**	CC22-MRSA-IVa (dcs negatives) (tst1+), "Gaza Epidemic Strain"	NEG	NEG	POS	POS	NEG	NEG	NEG	NEG	NEG	NEG	NEG	NEG	NEG
**Alexandria University_2020-11**	CC22-MRSA-IVa (dcs negatives) (tst1+), "Gaza Epidemic Strain"	NEG	NEG	POS	POS	NEG	NEG	NEG	NEG	NEG	NEG	NEG	NEG	NEG
**CC30**														
**Alexandria University_2020-26**	CC30-MRSA-IVa (PVL+), "WSPP/Southwest Pacific Clone"	NEG	NEG	POS	POS	NEG	NEG	NEG	NEG	NEG	NEG	NEG	NEG	NEG
**CC97**														
**Alexandria University_2020-30**	CC97-MRSA-[V+fus]	NEG	NEG	NEG	NEG	NEG	NEG	NEG	NEG	POS	POS	NEG	NEG	NEG
**CC121**														
**Alexandria University_2020-06**	CC121-MRSA-[V+fus] (PVL+)	NEG	NEG	NEG	NEG	NEG	NEG	NEG	NEG	POS	POS	NEG	NEG	NEG
**CC152**														
**Alexandria University_2020-18**	CC152-MRSA-[V+fus]	NEG	NEG	NEG	NEG	NEG	NEG	NEG	NEG	POS	POS	NEG	NEG	NEG
**Alexandria University_2020-31**	CC152-MRSA-[V+fus]	NEG	NEG	NEG	NEG	NEG	NEG	POS	NEG	POS	POS	NEG	NEG	NEG
**CC239**														
**Alexandria University_2020-13**	CC239-MRSA-[III+Cd+ccrC] (sasX-negat.), "Middle Eastern Cluster"	NEG	NEG	NEG	NEG	POS	POS	POS	NEG	POS	POS	NEG	NEG	NEG
**Alexandria University_2020-16**	CC239-MRSA-[III+Cd], sasX-negative	NEG	NEG	NEG	NEG	POS	POS	NEG	NEG	NEG	NEG	NEG	NEG	NEG
**CC1153**														
**Alexandria University_2020-07**	CC1153-MRSA-[V+fus] (PVL+)	NEG	NEG	NEG	NEG	NEG	NEG	NEG	NEG	POS	POS	NEG	NEG	NEG
**Alexandria University_2020-08**	CC1153-MRSA-[V+fus] (PVL+)	NEG	NEG	NEG	NEG	NEG	NEG	NEG	NEG	POS	POS	NEG	NEG	NEG
**Alexandria University_2020-23**	CC1153-MRSA-[V+fus] (PVL+)	NEG	NEG	NEG	NEG	NEG	NEG	NEG	NEG	POS	POS	NEG	NEG	NEG
**Simulated hybridisations for sequences from Montelongo et al., 2022**														
**CC1**														
**JAEOUR Staphylococcus aureus strain AA1**	CC1-MRSA-[V+fus+tir+ccrAB1]	POS	POS	NEG	NEG	NEG	AMB		POS	POS	POS	NEG	NEG	NEG
**JAEOWK Staphylococcus aureus strain AA78**	CC1-MRSA-[V+fus+tir+ccrAB1]	POS	POS	NEG	NEG	NEG	AMB		NEG	AMB	POS	NEG	NEG	NEG
**JAEOVR Staphylococcus aureus strain AA51**	CC1-MRSA-[V+fus+tir+ccrAB1] (PVL+)	POS	POS	NEG	NEG	NEG	AMB		NEG	NEG	POS	NEG	NEG	NEG
**JAEOWE Staphylococcus aureus strain AA67**	CC1-MRSA-[V+fus+tir+ccrAB1] (PVL+)	POS	POS	NEG	NEG	NEG	AMB		NEG	NEG	POS	NEG	NEG	NEG
**JAEOWG Staphylococcus aureus strain AA69**	CC1-MRSA-[V+fus+tir+ccrAB1] (PVL+)	POS	POS	NEG	NEG	NEG	AMB		NEG	AMB	POS	NEG	NEG	NEG
**JAEOWJ Staphylococcus aureus strain AA77**	CC1-MRSA-[V+fus+tir+ccrAB1] (PVL+)	POS	POS	NEG	NEG	NEG	AMB		NEG	AMB	POS	NEG	NEG	NEG
**CC5**														
**JAEOWI Staphylococcus aureus strain AA76**	CC5-MRSA-[V+cas], WA MRSA-123	NEG	NEG	NEG	NEG	NEG	NEG		POS	POS	POS	NEG	NEG	NEG
**JAEOWN Staphylococcus aureus strain AA80**	CC5-MRSA-[V+cas], WA MRSA-123	NEG	NEG	NEG	NEG	NEG	NEG		POS	POS	POS	NEG	NEG	NEG
**JAEOWH Staphylococcus aureus strain AA70**	CC5-MRSA-[VI+fus+tir+, dcs-]	NEG	NEG	NEG	NEG	NEG	NEG		NEG	NEG	NEG	POS	POS	NEG
**CC6**														
**JAEOVF Staphylococcus aureus strain AA30**	CC6-MRSA-IVa, WA MRSA-51	NEG	NEG	POS	POS	NEG	NEG		NEG	NEG	NEG	NEG	NEG	POS
**CC80**														
**JAEOVM Staphylococcus aureus strain AA4**	CC80-MRSA-IVc (PVL-) [aphA3/sat+]:[far1+], contaminated	NEG	NEG	POS	POS	NEG	POS		NEG	NEG	NEG	NEG	NEG	POS
**JAEOUZ Staphylococcus aureus strain AA2**	CC80-MRSA-IVc (PVL+) [aphA3/sat+]:[far1+]	NEG	NEG	POS	POS	NEG	NEG		NEG	NEG	NEG	NEG	NEG	POS
**JAEOVE Staphylococcus aureus strain AA3**	CC80-MRSA-IVc (PVL+) [aphA3/sat+]:[far1+]	NEG	NEG	POS	POS	POS	NEG		NEG	NEG	NEG	NEG	NEG	POS
**JAEOVO Staphylococcus aureus strain AA45**	CC80-MRSA-IVc (PVL+) [aphA3/sat+]:[far1+]	NEG	NEG	POS	POS	NEG	NEG		NEG	NEG	NEG	NEG	NEG	POS
**CC88**														
**JAEOVT Staphylococcus aureus strain AA53**	CC88-MRSA-IV, contaminated	NEG	NEG	POS	POS	NEG	NEG		NEG	AMB	POS	NEG	NEG	NEG
**CC97**														
**JAEOVL Staphylococcus aureus strain AA39**	CC97-MRSA-IVc, WA MRSA-54/63	NEG	NEG	POS	POS	NEG	NEG		NEG	NEG	NEG	NEG	NEG	POS
**JAEOVX Staphylococcus aureus strain AA6**	CC97-MRSA-IVc, WA MRSA-54/63	NEG	NEG	POS	POS	NEG	NEG		NEG	NEG	NEG	NEG	NEG	POS
**JAEOWM Staphylococcus aureus strain AA8**	CC97-MRSA-IVc, WA MRSA-54/63	NEG	NEG	POS	POS	NEG	NEG		NEG	NEG	NEG	NEG	NEG	POS
**JAEOUU Staphylococcus aureus strain AA104**	CC97-MRSA-V	NEG	NEG	NEG	NEG	NEG	NEG		NEG	AMB	POS	NEG	NEG	NEG
**JAEOVJ Staphylococcus aureus strain AA35**	CC97-MRSA-[V+fus]	NEG	NEG	NEG	NEG	NEG	NEG		NEG	AMB	POS	NEG	NEG	NEG
**JAEOVK Staphylococcus aureus strain AA36**	CC97-MRSA-[V+fus]	NEG	NEG	NEG	NEG	NEG	NEG		NEG	AMB	POS	NEG	NEG	NEG
**CC22**														
**JAEOUY Staphylococcus aureus strain AA18**	CC22-MRSA-IVa (dcs negatives) (tst1+), "Gaza Epidemic Strain"	NEG	NEG	POS	POS	NEG	NEG		NEG	NEG	NEG	NEG	NEG	NEG
**JAEOVH Staphylococcus aureus strain AA32**	CC22-MRSA-IVa (dcs negatives) (tst1+), "Gaza Epidemic Strain"	NEG	NEG	POS	POS	NEG	NEG		NEG	NEG	NEG	NEG	NEG	NEG
**JAEOVQ Staphylococcus aureus strain AA5**	CC22-MRSA-IVa (dcs negatives) (tst1+), "Gaza Epidemic Strain"	NEG	NEG	POS	POS	NEG	NEG		NEG	NEG	NEG	NEG	NEG	NEG
**CC152**														
**JAEOUX Staphylococcus aureus strain AA17**	CC152-MRSA-[V+fus]	NEG	NEG	NEG	NEG	NEG	NEG		NEG	AMB	POS	NEG	NEG	NEG
**CC239**														
**JAEOVI Staphylococcus aureus strain AA33**	CC239-MRSA-[III+Cd/Hg+ccrC] (sasX-positive), "Southeast Asian Clade"	NEG	NEG	NEG	NEG	POS	POS		NEG	AMB	POS	NEG	NEG	NEG
**JAEOUS Staphylococcus aureus strain AA101**	CC239-MRSA-[III+Cd+ccrC] (sasX-negat.), "Middle Eastern Cluster"	NEG	NEG	NEG	NEG	POS	POS		NEG	AMB	AMB	NEG	NEG	NEG
**JAEOUV Staphylococcus aureus strain AA13**	CC239-MRSA-[III+Cd+ccrC] (sasX-negat.), "Middle Eastern Cluster"	NEG	NEG	NEG	NEG	POS	POS		NEG	AMB	AMB	NEG	NEG	NEG
**JAEOUW Staphylococcus aureus strain AA14**	CC239-MRSA-[III+Cd+ccrC] (sasX-negat.), "Middle Eastern Cluster"	NEG	NEG	NEG	NEG	POS	POS		NEG	AMB	AMB	NEG	NEG	NEG
**JAEOVA Staphylococcus aureus strain AA22**	CC239-MRSA-[III+Cd+ccrC] (sasX-negat.), "Middle Eastern Cluster"	NEG	NEG	NEG	NEG	POS	POS		NEG	AMB	AMB	NEG	NEG	NEG
**JAEOVB Staphylococcus aureus strain AA23**	CC239-MRSA-[III+Cd+ccrC] (sasX-negat.), "Middle Eastern Cluster"	NEG	NEG	NEG	NEG	POS	POS		NEG	AMB	AMB	NEG	NEG	NEG
**JAEOVC Staphylococcus aureus strain AA27**	CC239-MRSA-[III+Cd+ccrC] (sasX-negat.), "Middle Eastern Cluster"	NEG	NEG	NEG	NEG	POS	POS		NEG	AMB	AMB	NEG	NEG	NEG
**JAEOVD Staphylococcus aureus strain AA29**	CC239-MRSA-[III+Cd+ccrC] (sasX-negat.), "Middle Eastern Cluster"	NEG	NEG	NEG	NEG	POS	POS		NEG	AMB	AMB	NEG	NEG	NEG
**JAEOVG Staphylococcus aureus strain AA31**	CC239-MRSA-[III+Cd+ccrC] (sasX-negat.), "Middle Eastern Cluster"	NEG	NEG	NEG	NEG	POS	POS		NEG	AMB	AMB	NEG	NEG	NEG
**JAEOVP Staphylococcus aureus strain AA46**	CC239-MRSA-[III+Cd+ccrC] (sasX-negat.), "Middle Eastern Cluster"	NEG	NEG	NEG	NEG	POS	POS		NEG	AMB	AMB	NEG	NEG	NEG
**JAEOVS Staphylococcus aureus strain AA52**	CC239-MRSA-[III+Cd+ccrC] (sasX-negat.), "Middle Eastern Cluster"	NEG	NEG	NEG	NEG	POS	POS		NEG	AMB	AMB	NEG	NEG	NEG
**JAEOVU Staphylococcus aureus strain AA55**	CC239-MRSA-[III+Cd+ccrC] (sasX-negat.), "Middle Eastern Cluster"	NEG	NEG	NEG	NEG	POS	POS		NEG	AMB	AMB	NEG	NEG	NEG
**JAEOVV Staphylococcus aureus strain AA57**	CC239-MRSA-[III+Cd+ccrC] (sasX-negat.), "Middle Eastern Cluster"	NEG	NEG	NEG	NEG	POS	POS		NEG	AMB	AMB	NEG	NEG	NEG
**JAEOVY Staphylococcus aureus strain AA60**	CC239-MRSA-[III+Cd+ccrC] (sasX-negat.), "Middle Eastern Cluster"	NEG	NEG	NEG	NEG	POS	POS		NEG	AMB	AMB	NEG	NEG	NEG
**JAEOVZ Staphylococcus aureus strain AA61**	CC239-MRSA-[III+Cd+ccrC] (sasX-negat.), "Middle Eastern Cluster"	NEG	NEG	NEG	NEG	POS	POS		NEG	AMB	AMB	NEG	NEG	NEG
**JAEOWA Staphylococcus aureus strain AA62**	CC239-MRSA-[III+Cd+ccrC] (sasX-negat.), "Middle Eastern Cluster"	NEG	NEG	NEG	NEG	POS	POS		NEG	AMB	AMB	NEG	NEG	NEG
**JAEOWB Staphylococcus aureus strain AA63**	CC239-MRSA-[III+Cd+ccrC] (sasX-negat.), "Middle Eastern Cluster"	NEG	NEG	NEG	NEG	POS	POS		NEG	AMB	AMB	NEG	NEG	NEG
**JAEOWL Staphylococcus aureus strain AA79**	CC239-MRSA-[III+Cd+ccrC] (sasX-negat.), "Middle Eastern Cluster"	NEG	NEG	NEG	NEG	POS	POS		NEG	AMB	AMB	NEG	NEG	NEG
**JAEOWQ Staphylococcus aureus strain AA92**	CC239-MRSA-[III+Cd+ccrC] (sasX-negat.), "Middle Eastern Cluster"	NEG	NEG	NEG	NEG	POS	POS		NEG	AMB	AMB	NEG	NEG	NEG
**JAEOWC Staphylococcus aureus strain AA64**	CC239-MRSA-[mec III+Cd], sasX-negative	NEG	NEG	NEG	NEG	POS	POS		NEG	NEG	NEG	NEG	NEG	NEG
**JAEOWP Staphylococcus aureus strain AA91**	CC239-MRSA-[mec III+Cd], sasX-negative	NEG	NEG	NEG	NEG	POS	POS		NEG	NEG	NEG	NEG	NEG	NEG

Isolate	Strain assignment	HEAVY METAL RESISTANCES				RESISTANCE : PENICILLINASE			RESISTANCE : MLS-ANTIBIOTICS					
		merA	merB	czrC	cadD (R35)	blaZ	blaI	blaR	ermA	ermB	ermC	linA/lnuA	lsa-E	msrA
		mercury resistance operon		cadmium and zinc resistance gene C, heavy metal translocating P-type ATPase		beta-lactamase	beta lactamase repressor (inhibitor)	beta-lactamase regulatory protein	erythro-mycin/clindamycin resistance			Lincosamid-Nucleotidyltransferase	lincosamide ABC transporter	energy-dependent efflux of erythro-mycin
**CC1**														
Alexandria University_2020-17	CC1-MRSA-[V+fus+tir]	NEG	NEG	NEG	NEG	POS	POS	POS	NEG	NEG	NEG	NEG	NEG	NEG
Alexandria University_2020-03	CC1-MRSA-[V+fus+tir+ccrAB1]	NEG	NEG	NEG	NEG	POS	POS	POS	NEG	NEG	NEG	NEG	NEG	NEG
Alexandria University_2020-19	CC1-MRSA-[V+fus+tir+ccrAB1]	NEG	NEG	NEG	NEG	POS	POS	POS	NEG	NEG	NEG	NEG	NEG	NEG
Alexandria University_2020-21	CC1-MRSA-[V+fus+tir+ccrAB1]	NEG	NEG	NEG	NEG	POS	POS	POS	NEG	NEG	POS	NEG	NEG	NEG
Alexandria University_2020-09	CC1-MRSA-[V+fus+tir+ccrAB1] (PVL+)	NEG	NEG	NEG	NEG	POS	POS	POS	NEG	NEG	NEG	NEG	NEG	NEG
Alexandria University_2020-14	CC1-MRSA-[V+fus+tir+ccrAB1] (PVL+)	NEG	NEG	NEG	NEG	POS	POS	POS	NEG	NEG	NEG	NEG	NEG	NEG
Alexandria University_2020-24	CC1-MRSA-[V+fus+tir+ccrAB1] (PVL+)	NEG	NEG	NEG	NEG	POS	POS	POS	NEG	NEG	NEG	NEG	NEG	NEG
Alexandria University_2020-27	CC1-MRSA-[V+fus+tir+ccrAB1] (PVL+)	NEG	NEG	NEG	NEG	POS	POS	POS	NEG	NEG	NEG	NEG	NEG	NEG
Alexandria University_2020-28	CC1-MRSA-[V+fus+tir+ccrAB1] (PVL+)	NEG	NEG	NEG	NEG	POS	POS	POS	NEG	NEG	NEG	NEG	NEG	NEG
**CC5**														
Alexandria University_2020-12	CC5-MRSA-[VI+fus+tir]	NEG	NEG	NEG	NEG	POS	POS	POS	NEG	NEG	POS	NEG	NEG	NEG
Alexandria University_2020-20	CC5-MRSA-[VI+fus+tir]	NEG	NEG	NEG	NEG	POS	POS	POS	NEG	POS	NEG	NEG	NEG	NEG
**CC6**														
Alexandria University_2020-29	CC6-MRSA-[V+fus]	NEG	NEG	NEG	NEG	POS	POS	POS	NEG	NEG	NEG	NEG	NEG	NEG
**CC15**														
Alexandria University_2020-01	CC15-MRSA-[V+fus]	NEG	NEG	NEG	NEG	POS	POS	POS	NEG	NEG	NEG	AMB	NEG	AMB
Alexandria University_2020-02	CC15-MRSA-[V+fus]	NEG	NEG	NEG	NEG	POS	POS	POS	NEG	NEG	NEG	AMB	NEG	AMB
Alexandria University_2020-05	CC15-MRSA-[V+fus]	NEG	NEG	NEG	NEG	POS	POS	POS	NEG	NEG	NEG	POS	NEG	AMB
Alexandria University_2020-15	CC15-MRSA-[V+fus]	NEG	NEG	NEG	NEG	NEG	NEG	NEG	NEG	NEG	NEG	NEG	NEG	NEG
Alexandria University_2020-22	CC15-MRSA-[V+fus]	NEG	NEG	NEG	NEG	POS	POS	POS	NEG	NEG	NEG	POS	NEG	AMB
Alexandria University_2020-32	CC15-MRSA-[V+fus]	NEG	NEG	NEG	NEG	POS	POS	POS	NEG	NEG	POS	AMB	NEG	AMB
**CC22**														
Alexandria University_2020-04	CC22-MRSA-IVa (dcs negatives) (tst1+), "Gaza Epidemic Strain"	NEG	NEG	NEG	NEG	POS	POS	AMB	NEG	NEG	NEG	NEG	NEG	NEG
Alexandria University_2020-11	CC22-MRSA-IVa (dcs negatives) (tst1+), "Gaza Epidemic Strain"	NEG	NEG	NEG	NEG	POS	POS	AMB	NEG	NEG	NEG	NEG	NEG	NEG
**CC30**														
Alexandria University_2020-26	CC30-MRSA-IVa (PVL+), "WSPP/Southwest Pacific Clone"	NEG	NEG	NEG	NEG	POS	POS	POS	POS	NEG	NEG	NEG	NEG	NEG
**CC97**														
Alexandria University_2020-30	CC97-MRSA-[V+fus]	NEG	NEG	NEG	NEG	POS	POS	POS	NEG	NEG	NEG	NEG	NEG	NEG
**CC121**														
Alexandria University_2020-06	CC121-MRSA-[V+fus] (PVL+)	NEG	NEG	NEG	NEG	POS	POS	POS	NEG	NEG	NEG	NEG	NEG	NEG
**CC152**														
Alexandria University_2020-18	CC152-MRSA-[V+fus]	NEG	NEG	NEG	NEG	POS	POS	POS	NEG	NEG	NEG	NEG	NEG	NEG
Alexandria University_2020-31	CC152-MRSA-[V+fus]	NEG	NEG	NEG	NEG	POS	POS	POS	NEG	NEG	NEG	NEG	NEG	NEG
**CC239**														
Alexandria University_2020-13	CC239-MRSA-[III+Cd+ccrC] (sasX-negat.), "Middle Eastern Cluster"	NEG	NEG	NEG	POS	POS	POS	POS	NEG	NEG	NEG	NEG	NEG	NEG
Alexandria University_2020-16	CC239-MRSA-[III+Cd], sasX-negative	NEG	NEG	NEG	POS	NEG	NEG	NEG	NEG	NEG	POS	NEG	NEG	NEG
**CC1153**														
Alexandria University_2020-07	CC1153-MRSA-[V+fus] (PVL+)	NEG	NEG	NEG	NEG	POS	POS	POS	NEG	NEG	NEG	NEG	NEG	NEG
Alexandria University_2020-08	CC1153-MRSA-[V+fus] (PVL+)	NEG	NEG	NEG	NEG	POS	POS	POS	NEG	NEG	NEG	NEG	NEG	NEG
Alexandria University_2020-23	CC1153-MRSA-[V+fus] (PVL+)	NEG	NEG	NEG	NEG	POS	POS	POS	NEG	NEG	NEG	NEG	NEG	NEG
**Simulated hybridisations for sequences from Montelongo et al., 2022**														
**CC1**														
JAEOUR Staphylococcus aureus strain AA1	CC1-MRSA-[V+fus+tir+ccrAB1]	NEG	NEG	NEG	NEG	POS	POS	POS	NEG	NEG	POS	NEG	NEG	NEG
JAEOWK Staphylococcus aureus strain AA78	CC1-MRSA-[V+fus+tir+ccrAB1]	NEG	NEG	NEG	NEG	POS	POS	POS	NEG	NEG	NEG	NEG	NEG	NEG
JAEOVR Staphylococcus aureus strain AA51	CC1-MRSA-[V+fus+tir+ccrAB1] (PVL+)	NEG	NEG	NEG	NEG	POS	POS	POS	NEG	NEG	AMB	NEG	NEG	NEG
JAEOWE Staphylococcus aureus strain AA67	CC1-MRSA-[V+fus+tir+ccrAB1] (PVL+)	NEG	NEG	NEG	NEG	POS	POS	POS	NEG	NEG	NEG	NEG	NEG	NEG
JAEOWG Staphylococcus aureus strain AA69	CC1-MRSA-[V+fus+tir+ccrAB1] (PVL+)	NEG	NEG	NEG	NEG	POS	POS	POS	NEG	NEG	NEG	NEG	NEG	NEG
JAEOWJ Staphylococcus aureus strain AA77	CC1-MRSA-[V+fus+tir+ccrAB1] (PVL+)	NEG	NEG	NEG	NEG	POS	POS	POS	NEG	NEG	NEG	NEG	NEG	NEG
**CC5**														
JAEOWI Staphylococcus aureus strain AA76	CC5-MRSA-[V+cas], WA MRSA-123	NEG	NEG	NEG	NEG	NEG	NEG	NEG	NEG	NEG	NEG	NEG	NEG	NEG
JAEOWN Staphylococcus aureus strain AA80	CC5-MRSA-[V+cas], WA MRSA-123	NEG	NEG	NEG	NEG	NEG	NEG	NEG	NEG	NEG	POS	NEG	NEG	NEG
JAEOWH Staphylococcus aureus strain AA70	CC5-MRSA-[VI+fus+tir+, dcs-]	NEG	NEG	NEG	NEG	POS	POS	POS	NEG	NEG	NEG	NEG	NEG	NEG
**CC6**														
JAEOVF Staphylococcus aureus strain AA30	CC6-MRSA-IVa, WA MRSA-51	NEG	NEG	NEG	NEG	POS	POS	POS	NEG	NEG	NEG	NEG	NEG	NEG
**CC80**														
JAEOVM Staphylococcus aureus strain AA4	CC80-MRSA-IVc (PVL-) [aphA3/sat+]:[far1+], contaminated	NEG	NEG	NEG	POS	POS	POS	POS	NEG	NEG	NEG	NEG	NEG	NEG
JAEOUZ Staphylococcus aureus strain AA2	CC80-MRSA-IVc (PVL+) [aphA3/sat+]:[far1+]	NEG	NEG	NEG	NEG	POS	POS	POS	NEG	NEG	NEG	NEG	NEG	NEG
JAEOVE Staphylococcus aureus strain AA3	CC80-MRSA-IVc (PVL+) [aphA3/sat+]:[far1+]	NEG	NEG	NEG	NEG	POS	POS	POS	NEG	NEG	NEG	NEG	NEG	NEG
JAEOVO Staphylococcus aureus strain AA45	CC80-MRSA-IVc (PVL+) [aphA3/sat+]:[far1+]	NEG	NEG	NEG	NEG	POS	POS	POS	NEG	NEG	NEG	NEG	NEG	NEG
**CC88**														
JAEOVT Staphylococcus aureus strain AA53	CC88-MRSA-IV, contaminated	NEG	NEG	NEG	NEG	POS	POS	POS	NEG	NEG	POS	NEG	NEG	NEG
**CC97**														
JAEOVL Staphylococcus aureus strain AA39	CC97-MRSA-IVc, WA MRSA-54/63	NEG	NEG	NEG	NEG	POS	POS	POS	NEG	NEG	NEG	NEG	NEG	NEG
JAEOVX Staphylococcus aureus strain AA6	CC97-MRSA-IVc, WA MRSA-54/63	NEG	NEG	NEG	NEG	POS	POS	POS	NEG	NEG	NEG	NEG	NEG	NEG
JAEOWM Staphylococcus aureus strain AA8	CC97-MRSA-IVc, WA MRSA-54/63	NEG	NEG	NEG	NEG	POS	POS	POS	NEG	NEG	NEG	NEG	NEG	NEG
JAEOUU Staphylococcus aureus strain AA104	CC97-MRSA-V	NEG	NEG	NEG	NEG	POS	POS	POS	NEG	NEG	NEG	NEG	NEG	NEG
JAEOVJ Staphylococcus aureus strain AA35	CC97-MRSA-[V+fus]	NEG	NEG	NEG	NEG	POS	POS	POS	NEG	NEG	POS	NEG	NEG	NEG
JAEOVK Staphylococcus aureus strain AA36	CC97-MRSA-[V+fus]	NEG	NEG	NEG	NEG	POS	POS	POS	NEG	NEG	NEG	NEG	NEG	NEG
**CC22**														
JAEOUY Staphylococcus aureus strain AA18	CC22-MRSA-IVa (dcs negatives) (tst1+), "Gaza Epidemic Strain"	NEG	NEG	NEG	NEG	POS	AMB	AMB	NEG	NEG	NEG	NEG	NEG	NEG
JAEOVH Staphylococcus aureus strain AA32	CC22-MRSA-IVa (dcs negatives) (tst1+), "Gaza Epidemic Strain"	NEG	NEG	NEG	NEG	POS	AMB	AMB	NEG	NEG	NEG	NEG	NEG	NEG
JAEOVQ Staphylococcus aureus strain AA5	CC22-MRSA-IVa (dcs negatives) (tst1+), "Gaza Epidemic Strain"	NEG	NEG	NEG	NEG	POS	AMB	AMB	NEG	NEG	NEG	NEG	NEG	NEG
**CC152**														
JAEOUX Staphylococcus aureus strain AA17	CC152-MRSA-[V+fus]	NEG	NEG	NEG	NEG	POS	POS	POS	NEG	NEG	POS	NEG	NEG	NEG
**CC239**														
JAEOVI Staphylococcus aureus strain AA33	CC239-MRSA-[III+Cd/Hg+ccrC] (sasX-positive), "Southeast Asian Clade"	POS	POS	NEG	POS	POS	POS	POS	POS	NEG	POS	NEG	NEG	NEG
JAEOUS Staphylococcus aureus strain AA101	CC239-MRSA-[III+Cd+ccrC] (sasX-negat.), "Middle Eastern Cluster"	NEG	NEG	NEG	POS	POS	POS	POS	NEG	NEG	AMB	NEG	NEG	NEG
JAEOUV Staphylococcus aureus strain AA13	CC239-MRSA-[III+Cd+ccrC] (sasX-negat.), "Middle Eastern Cluster"	NEG	NEG	NEG	POS	POS	POS	POS	NEG	NEG	POS	NEG	NEG	NEG
JAEOUW Staphylococcus aureus strain AA14	CC239-MRSA-[III+Cd+ccrC] (sasX-negat.), "Middle Eastern Cluster"	NEG	NEG	NEG	POS	POS	POS	POS	NEG	NEG	NEG	NEG	NEG	NEG
JAEOVA Staphylococcus aureus strain AA22	CC239-MRSA-[III+Cd+ccrC] (sasX-negat.), "Middle Eastern Cluster"	NEG	NEG	NEG	POS	POS	POS	POS	NEG	NEG	NEG	NEG	NEG	NEG
JAEOVB Staphylococcus aureus strain AA23	CC239-MRSA-[III+Cd+ccrC] (sasX-negat.), "Middle Eastern Cluster"	NEG	NEG	NEG	POS	POS	POS	POS	NEG	NEG	NEG	NEG	NEG	NEG
JAEOVC Staphylococcus aureus strain AA27	CC239-MRSA-[III+Cd+ccrC] (sasX-negat.), "Middle Eastern Cluster"	NEG	NEG	NEG	POS	POS	POS	POS	NEG	NEG	NEG	NEG	NEG	NEG
JAEOVD Staphylococcus aureus strain AA29	CC239-MRSA-[III+Cd+ccrC] (sasX-negat.), "Middle Eastern Cluster"	NEG	NEG	NEG	POS	POS	POS	POS	NEG	NEG	POS	NEG	NEG	NEG
JAEOVG Staphylococcus aureus strain AA31	CC239-MRSA-[III+Cd+ccrC] (sasX-negat.), "Middle Eastern Cluster"	NEG	NEG	NEG	POS	POS	POS	POS	NEG	NEG	POS	NEG	NEG	NEG
JAEOVP Staphylococcus aureus strain AA46	CC239-MRSA-[III+Cd+ccrC] (sasX-negat.), "Middle Eastern Cluster"	NEG	NEG	NEG	POS	POS	POS	POS	NEG	NEG	NEG	NEG	NEG	NEG
JAEOVS Staphylococcus aureus strain AA52	CC239-MRSA-[III+Cd+ccrC] (sasX-negat.), "Middle Eastern Cluster"	NEG	NEG	NEG	POS	POS	POS	POS	NEG	NEG	NEG	NEG	NEG	NEG
JAEOVU Staphylococcus aureus strain AA55	CC239-MRSA-[III+Cd+ccrC] (sasX-negat.), "Middle Eastern Cluster"	NEG	NEG	NEG	POS	POS	POS	POS	NEG	NEG	NEG	NEG	NEG	NEG
JAEOVV Staphylococcus aureus strain AA57	CC239-MRSA-[III+Cd+ccrC] (sasX-negat.), "Middle Eastern Cluster"	NEG	NEG	NEG	POS	POS	POS	POS	NEG	NEG	NEG	NEG	NEG	NEG
JAEOVY Staphylococcus aureus strain AA60	CC239-MRSA-[III+Cd+ccrC] (sasX-negat.), "Middle Eastern Cluster"	NEG	NEG	NEG	POS	POS	POS	POS	NEG	NEG	POS	NEG	NEG	NEG
JAEOVZ Staphylococcus aureus strain AA61	CC239-MRSA-[III+Cd+ccrC] (sasX-negat.), "Middle Eastern Cluster"	NEG	NEG	NEG	POS	POS	POS	POS	NEG	NEG	POS	NEG	NEG	NEG
JAEOWA Staphylococcus aureus strain AA62	CC239-MRSA-[III+Cd+ccrC] (sasX-negat.), "Middle Eastern Cluster"	NEG	NEG	NEG	POS	POS	POS	POS	NEG	NEG	POS	NEG	NEG	NEG
JAEOWB Staphylococcus aureus strain AA63	CC239-MRSA-[III+Cd+ccrC] (sasX-negat.), "Middle Eastern Cluster"	NEG	NEG	NEG	POS	POS	POS	POS	NEG	NEG	POS	NEG	NEG	NEG
JAEOWL Staphylococcus aureus strain AA79	CC239-MRSA-[III+Cd+ccrC] (sasX-negat.), "Middle Eastern Cluster"	NEG	NEG	NEG	POS	POS	POS	POS	NEG	NEG	POS	NEG	NEG	NEG
JAEOWQ Staphylococcus aureus strain AA92	CC239-MRSA-[III+Cd+ccrC] (sasX-negat.), "Middle Eastern Cluster"	NEG	NEG	NEG	POS	POS	POS	POS	NEG	NEG	POS	NEG	NEG	NEG
JAEOWC Staphylococcus aureus strain AA64	CC239-MRSA-[mec III+Cd], sasX-negative	NEG	NEG	NEG	POS	POS	POS	POS	NEG	NEG	POS	NEG	NEG	NEG
JAEOWP Staphylococcus aureus strain AA91	CC239-MRSA-[mec III+Cd], sasX-negative	NEG	NEG	NEG	POS	POS	POS	POS	NEG	NEG	POS	NEG	NEG	NEG

Isolate	Strain assignment	RESISTANCE : MLS-ANTIBIOTICS							RESISTANCE : AMINOGLYSOSIDES			RESISTANCE : MISCELLANEOUS GENES			
		mefA	mph(C)	vat(A)	vat(B)	vga(A)	vga(A) (BM 3327)	vgb	aacA-aphD	aadD	aphA3	sat	dfrA	far1	mupA
		macrolide efflux protein A	probable lysylphos-phatidyl-glycerol synthetase	virginiamycin A acetyltransferase	acetyl-transferase inactivating streptogramin A	ATP binding protein, streptogramin-A-resistance		virginiamycin B hydrolase	bifunctional enzyme Aac/Aph, gentamicin resistance	amino-glycoside adenyl-transferase,tobra mycin resistance	3'5'-aminog-lycoside phospho-transferase, neo-/ kanamycin resistance	strepto-thricine-acetyl-transferase	dihydro-folate reductase type 1	fusidic acid resistence	mupirocin resistence protein
**CC1**															
Alexandria University_2020-17	CC1-MRSA-[V+fus+tir]	NEG	NEG	NEG	NEG	NEG	NEG	NEG	NEG	NEG	POS	POS	NEG	NEG	NEG
Alexandria University_2020-03	CC1-MRSA-[V+fus+tir+ccrAB1]	NEG	NEG	NEG	NEG	NEG	NEG	NEG	POS	NEG	POS	NEG	NEG	NEG	NEG
Alexandria University_2020-19	CC1-MRSA-[V+fus+tir+ccrAB1]	NEG	NEG	NEG	NEG	NEG	NEG	NEG	POS	NEG	POS	POS	NEG	NEG	NEG
Alexandria University_2020-21	CC1-MRSA-[V+fus+tir+ccrAB1]	NEG	NEG	NEG	NEG	NEG	NEG	NEG	POS	NEG	POS	POS	NEG	NEG	NEG
Alexandria University_2020-09	CC1-MRSA-[V+fus+tir+ccrAB1] (PVL+)	NEG	NEG	NEG	NEG	NEG	NEG	NEG	POS	NEG	POS	NEG	NEG	NEG	NEG
Alexandria University_2020-14	CC1-MRSA-[V+fus+tir+ccrAB1] (PVL+)	NEG	NEG	NEG	NEG	NEG	NEG	NEG	POS	NEG	POS	POS	NEG	NEG	NEG
Alexandria University_2020-24	CC1-MRSA-[V+fus+tir+ccrAB1] (PVL+)	NEG	NEG	NEG	NEG	NEG	NEG	NEG	POS	NEG	POS	POS	NEG	NEG	NEG
Alexandria University_2020-27	CC1-MRSA-[V+fus+tir+ccrAB1] (PVL+)	NEG	NEG	NEG	NEG	NEG	NEG	NEG	POS	POS	POS	POS	NEG	NEG	POS
Alexandria University_2020-28	CC1-MRSA-[V+fus+tir+ccrAB1] (PVL+)	NEG	NEG	NEG	NEG	NEG	NEG	NEG	POS	NEG	POS	NEG	NEG	NEG	NEG
**CC5**															
Alexandria University_2020-12	CC5-MRSA-[VI+fus+tir]	NEG	NEG	NEG	NEG	NEG	NEG	NEG	NEG	NEG	NEG	NEG	POS	NEG	NEG
Alexandria University_2020-20	CC5-MRSA-[VI+fus+tir]	NEG	NEG	NEG	NEG	NEG	NEG	NEG	POS	NEG	POS	POS	AMB	NEG	NEG
**CC6**															
Alexandria University_2020-29	CC6-MRSA-[V+fus]	NEG	NEG	NEG	NEG	NEG	NEG	NEG	POS	NEG	NEG	NEG	NEG	NEG	NEG
**CC15**															
Alexandria University_2020-01	CC15-MRSA-[V+fus]	NEG	NEG	NEG	NEG	NEG	NEG	NEG	POS	POS	NEG	NEG	NEG	NEG	NEG
Alexandria University_2020-02	CC15-MRSA-[V+fus]	NEG	NEG	NEG	NEG	NEG	NEG	NEG	POS	POS	NEG	NEG	NEG	NEG	NEG
Alexandria University_2020-05	CC15-MRSA-[V+fus]	NEG	NEG	NEG	NEG	NEG	NEG	NEG	POS	POS	NEG	NEG	NEG	NEG	NEG
Alexandria University_2020-15	CC15-MRSA-[V+fus]	NEG	NEG	NEG	NEG	NEG	NEG	NEG	POS	NEG	NEG	NEG	NEG	NEG	NEG
Alexandria University_2020-22	CC15-MRSA-[V+fus]	NEG	NEG	NEG	NEG	NEG	NEG	NEG	POS	POS	NEG	NEG	NEG	NEG	NEG
Alexandria University_2020-32	CC15-MRSA-[V+fus]	NEG	NEG	NEG	NEG	NEG	NEG	NEG	POS	POS	NEG	NEG	NEG	NEG	NEG
**CC22**															
Alexandria University_2020-04	CC22-MRSA-IVa (dcs negatives) (tst1+), "Gaza Epidemic Strain"	NEG	NEG	NEG	NEG	NEG	NEG	NEG	NEG	NEG	NEG	NEG	AMB	NEG	NEG
Alexandria University_2020-11	CC22-MRSA-IVa (dcs negatives) (tst1+), "Gaza Epidemic Strain"	NEG	NEG	NEG	NEG	NEG	NEG	NEG	NEG	NEG	NEG	NEG	NEG	NEG	NEG
**CC30**															
Alexandria University_2020-26	CC30-MRSA-IVa (PVL+), "WSPP/Southwest Pacific Clone"	NEG	NEG	NEG	NEG	NEG	NEG	NEG	NEG	NEG	NEG	NEG	AMB	NEG	NEG
**CC97**															
Alexandria University_2020-30	CC97-MRSA-[V+fus]	NEG	NEG	NEG	NEG	NEG	NEG	NEG	POS	NEG	NEG	NEG	NEG	NEG	NEG
**CC121**															
Alexandria University_2020-06	CC121-MRSA-[V+fus] (PVL+)	NEG	NEG	NEG	NEG	NEG	NEG	NEG	POS	NEG	NEG	NEG	NEG	NEG	NEG
**CC152**															
Alexandria University_2020-18	CC152-MRSA-[V+fus]	NEG	NEG	NEG	NEG	NEG	NEG	NEG	POS	NEG	NEG	NEG	NEG	NEG	NEG
Alexandria University_2020-31	CC152-MRSA-[V+fus]	NEG	NEG	NEG	NEG	NEG	NEG	NEG	POS	NEG	NEG	NEG	NEG	NEG	NEG
**CC239**															
Alexandria University_2020-13	CC239-MRSA-[III+Cd+ccrC] (sasX-negat.), "Middle Eastern Cluster"	NEG	NEG	NEG	NEG	NEG	NEG	NEG	POS	NEG	POS	POS	NEG	NEG	NEG
Alexandria University_2020-16	CC239-MRSA-[III+Cd], sasX-negative	NEG	NEG	NEG	NEG	NEG	NEG	NEG	POS	NEG	POS	POS	NEG	NEG	NEG
**CC1153**															
Alexandria University_2020-07	CC1153-MRSA-[V+fus] (PVL+)	NEG	NEG	NEG	NEG	NEG	NEG	NEG	AMB	NEG	NEG	NEG	NEG	NEG	NEG
Alexandria University_2020-08	CC1153-MRSA-[V+fus] (PVL+)	NEG	NEG	NEG	NEG	NEG	NEG	NEG	POS	NEG	NEG	NEG	NEG	NEG	NEG
Alexandria University_2020-23	CC1153-MRSA-[V+fus] (PVL+)	NEG	NEG	NEG	NEG	NEG	NEG	NEG	POS	NEG	NEG	NEG	NEG	NEG	NEG
**Simulated hybridisations for sequences from Montelongo et al., 2022**															
**CC1**															
JAEOUR Staphylococcus aureus strain AA1	CC1-MRSA-[V+fus+tir+ccrAB1]	NEG	NEG	NEG	NEG	NEG	NEG	NEG	POS	NEG	POS	POS	NEG	NEG	NEG
JAEOWK Staphylococcus aureus strain AA78	CC1-MRSA-[V+fus+tir+ccrAB1]	NEG	NEG	NEG	NEG	NEG	NEG	NEG	POS	NEG	POS	NEG	NEG	NEG	NEG
JAEOVR Staphylococcus aureus strain AA51	CC1-MRSA-[V+fus+tir+ccrAB1] (PVL+)	NEG	NEG	NEG	NEG	NEG	NEG	NEG	POS	NEG	POS	POS	NEG	NEG	NEG
JAEOWE Staphylococcus aureus strain AA67	CC1-MRSA-[V+fus+tir+ccrAB1] (PVL+)	NEG	NEG	NEG	NEG	NEG	NEG	NEG	POS	NEG	POS	POS	NEG	NEG	NEG
JAEOWG Staphylococcus aureus strain AA69	CC1-MRSA-[V+fus+tir+ccrAB1] (PVL+)	NEG	NEG	NEG	NEG	NEG	NEG	NEG	POS	NEG	POS	NEG	NEG	NEG	NEG
JAEOWJ Staphylococcus aureus strain AA77	CC1-MRSA-[V+fus+tir+ccrAB1] (PVL+)	NEG	NEG	NEG	NEG	NEG	NEG	NEG	POS	NEG	POS	POS	NEG	NEG	NEG
**CC5**															
JAEOWI Staphylococcus aureus strain AA76	CC5-MRSA-[V+cas], WA MRSA-123	NEG	NEG	NEG	NEG	NEG	NEG	NEG	NEG	NEG	NEG	NEG	NEG	NEG	NEG
JAEOWN Staphylococcus aureus strain AA80	CC5-MRSA-[V+cas], WA MRSA-123	NEG	NEG	NEG	NEG	NEG	NEG	NEG	NEG	POS	NEG	NEG	NEG	NEG	NEG
JAEOWH Staphylococcus aureus strain AA70	CC5-MRSA-[VI+fus+tir+, dcs-]	NEG	NEG	NEG	NEG	NEG	NEG	NEG	NEG	NEG	NEG	NEG	POS	NEG	NEG
**CC6**															
JAEOVF Staphylococcus aureus strain AA30	CC6-MRSA-IVa, WA MRSA-51	NEG	NEG	NEG	NEG	NEG	NEG	NEG	NEG	NEG	NEG	NEG	NEG	NEG	NEG
**CC80**															
JAEOVM Staphylococcus aureus strain AA4	CC80-MRSA-IVc (PVL-) [aphA3/sat+]:[far1+], contaminated	NEG	NEG	NEG	NEG	NEG	NEG	NEG	POS	NEG	POS	POS	NEG	POS	NEG
JAEOUZ Staphylococcus aureus strain AA2	CC80-MRSA-IVc (PVL+) [aphA3/sat+]:[far1+]	NEG	NEG	NEG	NEG	NEG	NEG	NEG	NEG	NEG	POS	POS	NEG	POS	NEG
JAEOVE Staphylococcus aureus strain AA3	CC80-MRSA-IVc (PVL+) [aphA3/sat+]:[far1+]	NEG	NEG	NEG	NEG	NEG	NEG	NEG	NEG	NEG	POS	POS	NEG	POS	NEG
JAEOVO Staphylococcus aureus strain AA45	CC80-MRSA-IVc (PVL+) [aphA3/sat+]:[far1+]	NEG	NEG	NEG	NEG	NEG	NEG	NEG	NEG	NEG	POS	POS	NEG	POS	NEG
**CC88**															
JAEOVT Staphylococcus aureus strain AA53	CC88-MRSA-IV, contaminated	NEG	NEG	NEG	NEG	NEG	NEG	NEG	POS	NEG	NEG	NEG	POS	NEG	NEG
**CC97**															
JAEOVL Staphylococcus aureus strain AA39	CC97-MRSA-IVc, WA MRSA-54/63	NEG	NEG	NEG	NEG	NEG	NEG	NEG	NEG	NEG	NEG	NEG	NEG	NEG	NEG
JAEOVX Staphylococcus aureus strain AA6	CC97-MRSA-IVc, WA MRSA-54/63	NEG	NEG	NEG	NEG	NEG	NEG	NEG	NEG	NEG	NEG	NEG	NEG	NEG	NEG
JAEOWM Staphylococcus aureus strain AA8	CC97-MRSA-IVc, WA MRSA-54/63	NEG	NEG	NEG	NEG	NEG	NEG	NEG	NEG	NEG	NEG	NEG	NEG	NEG	NEG
JAEOUU Staphylococcus aureus strain AA104	CC97-MRSA-V	NEG	NEG	NEG	NEG	NEG	NEG	NEG	POS	NEG	NEG	NEG	NEG	NEG	NEG
JAEOVJ Staphylococcus aureus strain AA35	CC97-MRSA-[V+fus]	NEG	NEG	NEG	NEG	NEG	NEG	NEG	POS	NEG	NEG	NEG	NEG	NEG	NEG
JAEOVK Staphylococcus aureus strain AA36	CC97-MRSA-[V+fus]	NEG	NEG	NEG	NEG	NEG	NEG	NEG	POS	NEG	NEG	NEG	NEG	NEG	NEG
**CC22**															
JAEOUY Staphylococcus aureus strain AA18	CC22-MRSA-IVa (dcs negatives) (tst1+), "Gaza Epidemic Strain"	NEG	NEG	NEG	NEG	NEG	NEG	NEG	NEG	NEG	NEG	NEG	POS	NEG	NEG
JAEOVH Staphylococcus aureus strain AA32	CC22-MRSA-IVa (dcs negatives) (tst1+), "Gaza Epidemic Strain"	NEG	NEG	NEG	NEG	NEG	NEG	NEG	NEG	NEG	NEG	NEG	POS	NEG	NEG
JAEOVQ Staphylococcus aureus strain AA5	CC22-MRSA-IVa (dcs negatives) (tst1+), "Gaza Epidemic Strain"	NEG	NEG	NEG	NEG	NEG	NEG	NEG	NEG	NEG	NEG	NEG	POS	NEG	NEG
**CC152**															
JAEOUX Staphylococcus aureus strain AA17	CC152-MRSA-[V+fus]	NEG	NEG	NEG	NEG	NEG	NEG	NEG	POS	NEG	NEG	NEG	NEG	NEG	NEG
**CC239**															
JAEOVI Staphylococcus aureus strain AA33	CC239-MRSA-[III+Cd/Hg+ccrC] (sasX-positive), "Southeast Asian Clade"	NEG	NEG	NEG	NEG	NEG	NEG	NEG	POS	NEG	POS	POS	NEG	NEG	NEG
JAEOUS Staphylococcus aureus strain AA101	CC239-MRSA-[III+Cd+ccrC] (sasX-negat.), "Middle Eastern Cluster"	NEG	NEG	NEG	NEG	NEG	NEG	NEG	POS	NEG	POS	POS	NEG	NEG	NEG
JAEOUV Staphylococcus aureus strain AA13	CC239-MRSA-[III+Cd+ccrC] (sasX-negat.), "Middle Eastern Cluster"	NEG	NEG	NEG	NEG	NEG	NEG	NEG	POS	NEG	POS	POS	NEG	NEG	NEG
JAEOUW Staphylococcus aureus strain AA14	CC239-MRSA-[III+Cd+ccrC] (sasX-negat.), "Middle Eastern Cluster"	NEG	NEG	NEG	NEG	NEG	NEG	NEG	POS	NEG	POS	POS	NEG	NEG	NEG
JAEOVA Staphylococcus aureus strain AA22	CC239-MRSA-[III+Cd+ccrC] (sasX-negat.), "Middle Eastern Cluster"	NEG	NEG	NEG	NEG	NEG	NEG	NEG	POS	NEG	POS	POS	NEG	NEG	NEG
JAEOVB Staphylococcus aureus strain AA23	CC239-MRSA-[III+Cd+ccrC] (sasX-negat.), "Middle Eastern Cluster"	NEG	NEG	NEG	NEG	NEG	NEG	NEG	POS	NEG	POS	POS	NEG	NEG	NEG
JAEOVC Staphylococcus aureus strain AA27	CC239-MRSA-[III+Cd+ccrC] (sasX-negat.), "Middle Eastern Cluster"	NEG	NEG	NEG	NEG	NEG	NEG	NEG	POS	NEG	POS	POS	NEG	NEG	NEG
JAEOVD Staphylococcus aureus strain AA29	CC239-MRSA-[III+Cd+ccrC] (sasX-negat.), "Middle Eastern Cluster"	NEG	NEG	NEG	NEG	NEG	NEG	NEG	POS	NEG	POS	POS	NEG	NEG	NEG
JAEOVG Staphylococcus aureus strain AA31	CC239-MRSA-[III+Cd+ccrC] (sasX-negat.), "Middle Eastern Cluster"	NEG	NEG	NEG	NEG	NEG	NEG	NEG	POS	NEG	POS	POS	NEG	NEG	NEG
JAEOVP Staphylococcus aureus strain AA46	CC239-MRSA-[III+Cd+ccrC] (sasX-negat.), "Middle Eastern Cluster"	NEG	NEG	NEG	NEG	NEG	NEG	NEG	POS	NEG	POS	POS	NEG	NEG	NEG
JAEOVS Staphylococcus aureus strain AA52	CC239-MRSA-[III+Cd+ccrC] (sasX-negat.), "Middle Eastern Cluster"	NEG	NEG	NEG	NEG	NEG	NEG	NEG	POS	NEG	POS	POS	NEG	NEG	NEG
JAEOVU Staphylococcus aureus strain AA55	CC239-MRSA-[III+Cd+ccrC] (sasX-negat.), "Middle Eastern Cluster"	NEG	NEG	NEG	NEG	NEG	NEG	NEG	POS	NEG	POS	POS	NEG	NEG	NEG
JAEOVV Staphylococcus aureus strain AA57	CC239-MRSA-[III+Cd+ccrC] (sasX-negat.), "Middle Eastern Cluster"	NEG	NEG	NEG	NEG	NEG	NEG	NEG	POS	NEG	POS	POS	NEG	NEG	NEG
JAEOVY Staphylococcus aureus strain AA60	CC239-MRSA-[III+Cd+ccrC] (sasX-negat.), "Middle Eastern Cluster"	NEG	NEG	NEG	NEG	NEG	NEG	NEG	POS	NEG	POS	POS	NEG	NEG	NEG
JAEOVZ Staphylococcus aureus strain AA61	CC239-MRSA-[III+Cd+ccrC] (sasX-negat.), "Middle Eastern Cluster"	NEG	NEG	NEG	NEG	NEG	NEG	NEG	POS	NEG	POS	POS	NEG	NEG	NEG
JAEOWA Staphylococcus aureus strain AA62	CC239-MRSA-[III+Cd+ccrC] (sasX-negat.), "Middle Eastern Cluster"	NEG	NEG	NEG	NEG	NEG	NEG	NEG	POS	NEG	POS	POS	NEG	NEG	NEG
JAEOWB Staphylococcus aureus strain AA63	CC239-MRSA-[III+Cd+ccrC] (sasX-negat.), "Middle Eastern Cluster"	NEG	NEG	NEG	NEG	NEG	NEG	NEG	POS	NEG	POS	POS	NEG	NEG	NEG
JAEOWL Staphylococcus aureus strain AA79	CC239-MRSA-[III+Cd+ccrC] (sasX-negat.), "Middle Eastern Cluster"	NEG	NEG	NEG	NEG	NEG	NEG	NEG	POS	NEG	POS	POS	NEG	NEG	NEG
JAEOWQ Staphylococcus aureus strain AA92	CC239-MRSA-[III+Cd+ccrC] (sasX-negat.), "Middle Eastern Cluster"	NEG	NEG	NEG	NEG	NEG	NEG	NEG	POS	NEG	POS	POS	NEG	NEG	NEG
JAEOWC Staphylococcus aureus strain AA64	CC239-MRSA-[mec III+Cd], sasX-negative	NEG	NEG	NEG	NEG	NEG	NEG	NEG	POS	NEG	NEG	NEG	NEG	NEG	NEG
JAEOWP Staphylococcus aureus strain AA91	CC239-MRSA-[mec III+Cd], sasX-negative	NEG	NEG	NEG	NEG	NEG	NEG	NEG	POS	NEG	NEG	NEG	NEG	NEG	NEG

Isolate	Strain assignment	RESISTANCE : MISCELLANEOUS GENES												
		tetK	tetL	tetM	cat	cfr	fexA	fosB		qacA	qacC	vanA	vanB	vanZ
								fosB	fosB (plasmid)					
		tetracycline resistance	Tetracycline resista	tetracycline resistance	chloramphenicol acetyltransferase	23S rRNA methyltransferase	chloramphenicol/florfenicol exporter	metallothiol transferase		quaternary ammonium compound resistance protein A	quaternary ammonium compound resistance protein C	vancomycin resistance gene	vancomycin resistance gene from enterococci and Clostridium	teicoplanin resistance gene from enterococci
CC1														
Alexandria University_2020-17	CC1-MRSA-[V+fus+tir]	NEG	POS	NEG	NEG	NEG	NEG	NEG	NEG	NEG	NEG	NEG	NEG	NEG
Alexandria University_2020-03	CC1-MRSA-[V+fus+tir+ccrAB1]	NEG	POS	NEG	NEG	NEG	NEG	NEG	NEG	NEG	NEG	NEG	NEG	NEG
Alexandria University_2020-19	CC1-MRSA-[V+fus+tir+ccrAB1]	NEG	POS	NEG	NEG	NEG	NEG	NEG	NEG	NEG	NEG	NEG	NEG	NEG
Alexandria University_2020-21	CC1-MRSA-[V+fus+tir+ccrAB1]	NEG	POS	NEG	NEG	NEG	NEG	NEG	NEG	NEG	NEG	NEG	NEG	NEG
Alexandria University_2020-09	CC1-MRSA-[V+fus+tir+ccrAB1] (PVL+)	NEG	POS	NEG	NEG	NEG	NEG	NEG	NEG	NEG	NEG	NEG	NEG	NEG
Alexandria University_2020-14	CC1-MRSA-[V+fus+tir+ccrAB1] (PVL+)	NEG	POS	NEG	NEG	NEG	NEG	NEG	NEG	NEG	NEG	NEG	NEG	NEG
Alexandria University_2020-24	CC1-MRSA-[V+fus+tir+ccrAB1] (PVL+)	NEG	POS	NEG	NEG	NEG	NEG	NEG	NEG	NEG	NEG	NEG	NEG	NEG
Alexandria University_2020-27	CC1-MRSA-[V+fus+tir+ccrAB1] (PVL+)	NEG	POS	NEG	POS	POS	NEG	NEG	NEG	NEG	NEG	NEG	NEG	NEG
Alexandria University_2020-28	CC1-MRSA-[V+fus+tir+ccrAB1] (PVL+)	NEG	POS	NEG	NEG	NEG	NEG	NEG	NEG	NEG	NEG	NEG	NEG	NEG
CC5														
Alexandria University_2020-12	CC5-MRSA-[VI+fus+tir]	NEG	NEG	POS	NEG	NEG	POS	POS	NEG	NEG	NEG	NEG	NEG	NEG
Alexandria University_2020-20	CC5-MRSA-[VI+fus+tir]	NEG	NEG	POS	NEG	NEG	POS	POS	NEG	NEG	NEG	NEG	NEG	NEG
CC6														
Alexandria University_2020-29	CC6-MRSA-[V+fus]	NEG	NEG	NEG	NEG	NEG	NEG	POS	NEG	NEG	NEG	NEG	NEG	NEG
CC15														
Alexandria University_2020-01	CC15-MRSA-[V+fus]	POS	NEG	NEG	NEG	NEG	NEG	POS	NEG	NEG	NEG	NEG	NEG	NEG
Alexandria University_2020-02	CC15-MRSA-[V+fus]	POS	NEG	NEG	NEG	NEG	NEG	POS	NEG	NEG	NEG	NEG	NEG	NEG
Alexandria University_2020-05	CC15-MRSA-[V+fus]	POS	NEG	NEG	NEG	NEG	NEG	POS	NEG	NEG	NEG	NEG	NEG	NEG
Alexandria University_2020-15	CC15-MRSA-[V+fus]	NEG	NEG	NEG	NEG	NEG	NEG	POS	NEG	NEG	NEG	NEG	NEG	NEG
Alexandria University_2020-22	CC15-MRSA-[V+fus]	POS	NEG	NEG	NEG	NEG	NEG	POS	NEG	NEG	NEG	NEG	NEG	NEG
Alexandria University_2020-32	CC15-MRSA-[V+fus]	POS	NEG	NEG	NEG	NEG	NEG	POS	NEG	NEG	NEG	NEG	NEG	NEG
CC22														
Alexandria University_2020-04	CC22-MRSA-IVa (dcs negatives) (tst1+), "Gaza Epidemic Strain"	NEG	NEG	NEG	NEG	NEG	NEG	NEG	NEG	NEG	NEG	NEG	NEG	NEG
Alexandria University_2020-11	CC22-MRSA-IVa (dcs negatives) (tst1+), "Gaza Epidemic Strain"	NEG	NEG	NEG	NEG	NEG	NEG	NEG	NEG	NEG	NEG	NEG	NEG	NEG
CC30														
Alexandria University_2020-26	CC30-MRSA-IVa (PVL+), "WSPP/Southwest Pacific Clone"	NEG	NEG	NEG	NEG	NEG	NEG	POS	NEG	NEG	NEG	NEG	NEG	NEG
CC97														
Alexandria University_2020-30	CC97-MRSA-[V+fus]	NEG	NEG	NEG	NEG	NEG	NEG	NEG	NEG	NEG	NEG	NEG	NEG	NEG
CC121														
Alexandria University_2020-06	CC121-MRSA-[V+fus] (PVL+)	NEG	NEG	NEG	NEG	NEG	NEG	POS	NEG	NEG	NEG	NEG	NEG	NEG
CC152														
Alexandria University_2020-18	CC152-MRSA-[V+fus]	NEG	NEG	NEG	NEG	NEG	NEG	NEG	NEG	NEG	NEG	NEG	NEG	NEG
Alexandria University_2020-31	CC152-MRSA-[V+fus]	NEG	NEG	NEG	NEG	NEG	NEG	NEG	NEG	NEG	NEG	NEG	NEG	NEG
CC239														
Alexandria University_2020-13	CC239-MRSA-[III+Cd+ccrC] (sasX-negat.), "Middle Eastern Cluster"	POS	NEG	POS	NEG	NEG	NEG	POS	NEG	NEG	NEG	NEG	NEG	NEG
Alexandria University_2020-16	CC239-MRSA-[III+Cd], sasX-negative	POS	NEG	POS	NEG	NEG	NEG	POS	NEG	NEG	NEG	NEG	NEG	NEG
CC1153														
Alexandria University_2020-07	CC1153-MRSA-[V+fus] (PVL+)	POS	NEG	NEG	NEG	NEG	NEG	NEG	NEG	NEG	NEG	NEG	NEG	NEG
Alexandria University_2020-08	CC1153-MRSA-[V+fus] (PVL+)	NEG	NEG	NEG	NEG	NEG	NEG	NEG	NEG	NEG	NEG	NEG	NEG	NEG
Alexandria University_2020-23	CC1153-MRSA-[V+fus] (PVL+)	POS	NEG	NEG	NEG	NEG	NEG	NEG	NEG	NEG	NEG	NEG	NEG	NEG
Simulated hybridisations for sequences from Montelongo et al., 2022														
CC1														
JAEOUR Staphylococcus aureus strain AA1	CC1-MRSA-[V+fus+tir+ccrAB1]	NEG	POS	NEG	NEG	NEG	NEG	NEG	NEG	NEG	NEG	NEG	NEG	NEG
JAEOWK Staphylococcus aureus strain AA78	CC1-MRSA-[V+fus+tir+ccrAB1]	NEG	POS	NEG	NEG	NEG	NEG	NEG	NEG	NEG	NEG	NEG	NEG	NEG
JAEOVR Staphylococcus aureus strain AA51	CC1-MRSA-[V+fus+tir+ccrAB1] (PVL+)	NEG	POS	NEG	NEG	NEG	NEG	NEG	NEG	NEG	NEG	NEG	NEG	NEG
JAEOWE Staphylococcus aureus strain AA67	CC1-MRSA-[V+fus+tir+ccrAB1] (PVL+)	NEG	POS	NEG	NEG	NEG	NEG	NEG	NEG	NEG	NEG	NEG	NEG	NEG
JAEOWG Staphylococcus aureus strain AA69	CC1-MRSA-[V+fus+tir+ccrAB1] (PVL+)	NEG	POS	NEG	NEG	NEG	NEG	NEG	NEG	NEG	NEG	NEG	NEG	NEG
JAEOWJ Staphylococcus aureus strain AA77	CC1-MRSA-[V+fus+tir+ccrAB1] (PVL+)	NEG	POS	NEG	NEG	NEG	NEG	NEG	NEG	NEG	NEG	NEG	NEG	NEG
CC5														
JAEOWI Staphylococcus aureus strain AA76	CC5-MRSA-[V+cas], WA MRSA-123	POS	NEG	POS	NEG	NEG	POS	POS	NEG	NEG	POS	NEG	NEG	NEG
JAEOWN Staphylococcus aureus strain AA80	CC5-MRSA-[V+cas], WA MRSA-123	POS	NEG	POS	NEG	NEG	POS	POS	NEG	NEG	NEG	NEG	NEG	NEG
JAEOWH Staphylococcus aureus strain AA70	CC5-MRSA-[VI+fus+tir+, dcs-]	NEG	NEG	POS	NEG	NEG	POS	POS	NEG	NEG	NEG	NEG	NEG	NEG
CC6														
JAEOVF Staphylococcus aureus strain AA30	CC6-MRSA-IVa, WA MRSA-51	NEG	NEG	NEG	NEG	NEG	NEG	POS	NEG	NEG	NEG	NEG	NEG	NEG
CC80														
JAEOVM Staphylococcus aureus strain AA4	CC80-MRSA-IVc (PVL-) [aphA3/sat+]:[far1+], contaminated	POS	NEG	AMB	POS	NEG	NEG	NEG	NEG	NEG	NEG	NEG	NEG	NEG
JAEOUZ Staphylococcus aureus strain AA2	CC80-MRSA-IVc (PVL+) [aphA3/sat+]:[far1+]	POS	NEG	NEG	NEG	NEG	NEG	NEG	NEG	NEG	NEG	NEG	NEG	NEG
JAEOVE Staphylococcus aureus strain AA3	CC80-MRSA-IVc (PVL+) [aphA3/sat+]:[far1+]	POS	NEG	NEG	NEG	NEG	NEG	NEG	NEG	NEG	NEG	NEG	NEG	NEG
JAEOVO Staphylococcus aureus strain AA45	CC80-MRSA-IVc (PVL+) [aphA3/sat+]:[far1+]	POS	NEG	NEG	NEG	NEG	NEG	NEG	NEG	NEG	NEG	NEG	NEG	NEG
CC88														
JAEOVT Staphylococcus aureus strain AA53	CC88-MRSA-IV, contaminated	NEG	NEG	NEG	POS	NEG	NEG	NEG	NEG	NEG	NEG	NEG	NEG	NEG
CC97														
JAEOVL Staphylococcus aureus strain AA39	CC97-MRSA-IVc, WA MRSA-54/63	NEG	NEG	NEG	NEG	NEG	NEG	NEG	NEG	NEG	NEG	NEG	NEG	NEG
JAEOVX Staphylococcus aureus strain AA6	CC97-MRSA-IVc, WA MRSA-54/63	NEG	NEG	NEG	NEG	NEG	NEG	NEG	NEG	NEG	NEG	NEG	NEG	NEG
JAEOWM Staphylococcus aureus strain AA8	CC97-MRSA-IVc, WA MRSA-54/63	NEG	NEG	NEG	NEG	NEG	NEG	NEG	NEG	NEG	NEG	NEG	NEG	NEG
JAEOUU Staphylococcus aureus strain AA104	CC97-MRSA-V	NEG	NEG	NEG	NEG	NEG	NEG	NEG	NEG	NEG	NEG	NEG	NEG	NEG
JAEOVJ Staphylococcus aureus strain AA35	CC97-MRSA-[V+fus]	NEG	NEG	NEG	POS	NEG	NEG	NEG	NEG	NEG	NEG	NEG	NEG	NEG
JAEOVK Staphylococcus aureus strain AA36	CC97-MRSA-[V+fus]	POS	NEG	NEG	NEG	NEG	NEG	NEG	NEG	NEG	NEG	NEG	NEG	NEG
CC22														
JAEOUY Staphylococcus aureus strain AA18	CC22-MRSA-IVa (dcs negatives) (tst1+), "Gaza Epidemic Strain"	NEG	NEG	NEG	NEG	NEG	NEG	NEG	NEG	NEG	NEG	NEG	NEG	NEG
JAEOVH Staphylococcus aureus strain AA32	CC22-MRSA-IVa (dcs negatives) (tst1+), "Gaza Epidemic Strain"	NEG	NEG	NEG	NEG	NEG	NEG	NEG	NEG	NEG	NEG	NEG	NEG	NEG
JAEOVQ Staphylococcus aureus strain AA5	CC22-MRSA-IVa (dcs negatives) (tst1+), "Gaza Epidemic Strain"	NEG	NEG	NEG	NEG	NEG	NEG	NEG	NEG	NEG	NEG	NEG	NEG	NEG
CC152														
JAEOUX Staphylococcus aureus strain AA17	CC152-MRSA-[V+fus]	POS	NEG	NEG	NEG	NEG	NEG	NEG	NEG	NEG	NEG	NEG	NEG	NEG
CC239														
JAEOVI Staphylococcus aureus strain AA33	CC239-MRSA-[III+Cd/Hg+ccrC] (sasX-positive), "Southeast Asian Clade"	POS	NEG	POS	POS	NEG	NEG	POS	NEG	POS	NEG	NEG	NEG	NEG
JAEOUS Staphylococcus aureus strain AA101	CC239-MRSA-[III+Cd+ccrC] (sasX-negat.), "Middle Eastern Cluster"	POS	NEG	POS	NEG	NEG	NEG	POS	NEG	NEG	POS	NEG	NEG	NEG
JAEOUV Staphylococcus aureus strain AA13	CC239-MRSA-[III+Cd+ccrC] (sasX-negat.), "Middle Eastern Cluster"	POS	NEG	POS	NEG	NEG	NEG	POS	NEG	NEG	NEG	NEG	NEG	NEG
JAEOUW Staphylococcus aureus strain AA14	CC239-MRSA-[III+Cd+ccrC] (sasX-negat.), "Middle Eastern Cluster"	POS	NEG	POS	POS	NEG	NEG	POS	NEG	NEG	NEG	NEG	NEG	NEG
JAEOVA Staphylococcus aureus strain AA22	CC239-MRSA-[III+Cd+ccrC] (sasX-negat.), "Middle Eastern Cluster"	POS	NEG	POS	POS	NEG	NEG	POS	NEG	NEG	NEG	NEG	NEG	NEG
JAEOVB Staphylococcus aureus strain AA23	CC239-MRSA-[III+Cd+ccrC] (sasX-negat.), "Middle Eastern Cluster"	POS	NEG	POS	POS	NEG	NEG	POS	NEG	NEG	NEG	NEG	NEG	NEG
JAEOVC Staphylococcus aureus strain AA27	CC239-MRSA-[III+Cd+ccrC] (sasX-negat.), "Middle Eastern Cluster"	POS	NEG	POS	POS	NEG	NEG	POS	NEG	NEG	NEG	NEG	NEG	NEG
JAEOVD Staphylococcus aureus strain AA29	CC239-MRSA-[III+Cd+ccrC] (sasX-negat.), "Middle Eastern Cluster"	POS	NEG	POS	POS	NEG	NEG	POS	NEG	NEG	NEG	NEG	NEG	NEG
JAEOVG Staphylococcus aureus strain AA31	CC239-MRSA-[III+Cd+ccrC] (sasX-negat.), "Middle Eastern Cluster"	POS	NEG	POS	POS	NEG	NEG	POS	NEG	NEG	NEG	NEG	NEG	NEG
JAEOVP Staphylococcus aureus strain AA46	CC239-MRSA-[III+Cd+ccrC] (sasX-negat.), "Middle Eastern Cluster"	POS	NEG	POS	NEG	NEG	NEG	POS	NEG	NEG	NEG	NEG	NEG	NEG
JAEOVS Staphylococcus aureus strain AA52	CC239-MRSA-[III+Cd+ccrC] (sasX-negat.), "Middle Eastern Cluster"	POS	NEG	POS	NEG	NEG	NEG	POS	NEG	NEG	NEG	NEG	NEG	NEG
JAEOVU Staphylococcus aureus strain AA55	CC239-MRSA-[III+Cd+ccrC] (sasX-negat.), "Middle Eastern Cluster"	POS	NEG	POS	POS	NEG	NEG	POS	NEG	NEG	NEG	NEG	NEG	NEG
JAEOVV Staphylococcus aureus strain AA57	CC239-MRSA-[III+Cd+ccrC] (sasX-negat.), "Middle Eastern Cluster"	POS	NEG	POS	POS	NEG	NEG	POS	NEG	NEG	NEG	NEG	NEG	NEG
JAEOVY Staphylococcus aureus strain AA60	CC239-MRSA-[III+Cd+ccrC] (sasX-negat.), "Middle Eastern Cluster"	POS	NEG	POS	NEG	NEG	NEG	POS	NEG	NEG	NEG	NEG	NEG	NEG
JAEOVZ Staphylococcus aureus strain AA61	CC239-MRSA-[III+Cd+ccrC] (sasX-negat.), "Middle Eastern Cluster"	POS	POS	POS	POS	NEG	NEG	POS	NEG	NEG	NEG	NEG	NEG	NEG
JAEOWA Staphylococcus aureus strain AA62	CC239-MRSA-[III+Cd+ccrC] (sasX-negat.), "Middle Eastern Cluster"	POS	NEG	POS	POS	NEG	NEG	POS	NEG	NEG	NEG	NEG	NEG	NEG
JAEOWB Staphylococcus aureus strain AA63	CC239-MRSA-[III+Cd+ccrC] (sasX-negat.), "Middle Eastern Cluster"	POS	NEG	POS	POS	NEG	NEG	POS	NEG	NEG	NEG	NEG	NEG	NEG
JAEOWL Staphylococcus aureus strain AA79	CC239-MRSA-[III+Cd+ccrC] (sasX-negat.), "Middle Eastern Cluster"	POS	NEG	POS	POS	NEG	NEG	POS	NEG	NEG	NEG	NEG	NEG	NEG
JAEOWQ Staphylococcus aureus strain AA92	CC239-MRSA-[III+Cd+ccrC] (sasX-negat.), "Middle Eastern Cluster"	POS	NEG	POS	POS	NEG	NEG	POS	NEG	NEG	NEG	NEG	NEG	NEG
JAEOWC Staphylococcus aureus strain AA64	CC239-MRSA-[mec III+Cd], sasX-negative	POS	NEG	POS	POS	NEG	NEG	POS	NEG	NEG	NEG	NEG	NEG	NEG
JAEOWP Staphylococcus aureus strain AA91	CC239-MRSA-[mec III+Cd], sasX-negative	POS	POS	POS	POS	NEG	NEG	POS	NEG	NEG	POS	NEG	NEG	NEG

Isolate	Strain assignment	VIRULENCE : SUPERANTIGENS									
			seA								
		tst1	entA	entA (320E)	entA (N315) / entP	seB	seC	seD	seE	seH	seJ
		toxic shock syndrome toxin 1	Enterotoxin A	Enterotoxin A, allele from strain 320E	Enterotoxin A, allele from strain N315 = Enterotoxin P	Enterotoxin B	Enterotoxin C	Enterotoxin D	Enterotoxin E	Enterotoxin H	Enterotoxin J
**CC1**											
**Alexandria University_2020-17**	CC1-MRSA-[V+fus+tir]	NEG	POS	NEG	NEG	NEG	NEG	NEG	NEG	POS	NEG
**Alexandria University_2020-03**	CC1-MRSA-[V+fus+tir+ccrAB1]	NEG	POS	NEG	NEG	NEG	NEG	NEG	NEG	POS	NEG
**Alexandria University_2020-19**	CC1-MRSA-[V+fus+tir+ccrAB1]	NEG	POS	NEG	NEG	NEG	NEG	NEG	NEG	POS	NEG
**Alexandria University_2020-21**	CC1-MRSA-[V+fus+tir+ccrAB1]	NEG	POS	NEG	NEG	NEG	NEG	NEG	NEG	POS	NEG
**Alexandria University_2020-09**	CC1-MRSA-[V+fus+tir+ccrAB1] (PVL+)	NEG	POS	NEG	NEG	NEG	NEG	NEG	NEG	POS	NEG
**Alexandria University_2020-14**	CC1-MRSA-[V+fus+tir+ccrAB1] (PVL+)	NEG	POS	NEG	NEG	NEG	NEG	NEG	NEG	POS	NEG
**Alexandria University_2020-24**	CC1-MRSA-[V+fus+tir+ccrAB1] (PVL+)	NEG	POS	NEG	NEG	NEG	NEG	NEG	NEG	POS	NEG
**Alexandria University_2020-27**	CC1-MRSA-[V+fus+tir+ccrAB1] (PVL+)	NEG	POS	NEG	NEG	NEG	NEG	NEG	NEG	POS	NEG
**Alexandria University_2020-28**	CC1-MRSA-[V+fus+tir+ccrAB1] (PVL+)	NEG	POS	NEG	NEG	NEG	NEG	NEG	NEG	POS	NEG
**CC5**											
**Alexandria University_2020-12**	CC5-MRSA-[VI+fus+tir]	NEG	NEG	NEG	NEG	NEG	NEG	POS	NEG	NEG	POS
**Alexandria University_2020-20**	CC5-MRSA-[VI+fus+tir]	NEG	NEG	NEG	NEG	NEG	NEG	NEG	NEG	NEG	NEG
**CC6**											
**Alexandria University_2020-29**	CC6-MRSA-[V+fus]	NEG	POS	NEG	NEG	NEG	NEG	NEG	NEG	NEG	NEG
**CC15**											
**Alexandria University_2020-01**	CC15-MRSA-[V+fus]	NEG	NEG	NEG	NEG	NEG	NEG	NEG	NEG	NEG	NEG
**Alexandria University_2020-02**	CC15-MRSA-[V+fus]	NEG	NEG	NEG	NEG	NEG	NEG	NEG	NEG	NEG	NEG
**Alexandria University_2020-05**	CC15-MRSA-[V+fus]	NEG	NEG	NEG	NEG	AMB	NEG	NEG	NEG	NEG	NEG
**Alexandria University_2020-15**	CC15-MRSA-[V+fus]	NEG	NEG	NEG	NEG	NEG	NEG	NEG	NEG	NEG	NEG
**Alexandria University_2020-22**	CC15-MRSA-[V+fus]	NEG	NEG	NEG	NEG	AMB	NEG	NEG	NEG	NEG	NEG
**Alexandria University_2020-32**	CC15-MRSA-[V+fus]	NEG	NEG	NEG	NEG	NEG	NEG	NEG	NEG	NEG	NEG
**CC22**											
**Alexandria University_2020-04**	CC22-MRSA-IVa (dcs negatives) (tst1+), "Gaza Epidemic Strain"	POS	POS	NEG	NEG	NEG	NEG	NEG	NEG	NEG	NEG
**Alexandria University_2020-11**	CC22-MRSA-IVa (dcs negatives) (tst1+), "Gaza Epidemic Strain"	POS	NEG	NEG	NEG	NEG	NEG	NEG	NEG	NEG	NEG
**CC30**											
**Alexandria University_2020-26**	CC30-MRSA-IVa (PVL+), "WSPP/Southwest Pacific Clone"	NEG	NEG	NEG	NEG	NEG	NEG	NEG	NEG	NEG	NEG
**CC97**											
**Alexandria University_2020-30**	CC97-MRSA-[V+fus]	NEG	NEG	NEG	NEG	NEG	NEG	NEG	NEG	NEG	NEG
**CC121**											
**Alexandria University_2020-06**	CC121-MRSA-[V+fus] (PVL+)	NEG	NEG	NEG	NEG	POS	NEG	NEG	NEG	NEG	NEG
**CC152**											
**Alexandria University_2020-18**	CC152-MRSA-[V+fus]	NEG	NEG	NEG	NEG	NEG	NEG	NEG	NEG	NEG	NEG
**Alexandria University_2020-31**	CC152-MRSA-[V+fus]	NEG	NEG	NEG	NEG	NEG	NEG	NEG	NEG	NEG	NEG
**CC239**											
**Alexandria University_2020-13**	CC239-MRSA-[III+Cd+ccrC] (sasX-negat.), "Middle Eastern Cluster"	NEG	POS	NEG	NEG	NEG	NEG	NEG	NEG	NEG	NEG
**Alexandria University_2020-16**	CC239-MRSA-[III+Cd], sasX-negative	NEG	POS	NEG	NEG	NEG	NEG	NEG	NEG	NEG	NEG
**CC1153**											
**Alexandria University_2020-07**	CC1153-MRSA-[V+fus] (PVL+)	NEG	NEG	NEG	NEG	NEG	NEG	NEG	NEG	NEG	NEG
**Alexandria University_2020-08**	CC1153-MRSA-[V+fus] (PVL+)	NEG	NEG	NEG	NEG	NEG	NEG	NEG	NEG	NEG	NEG
**Alexandria University_2020-23**	CC1153-MRSA-[V+fus] (PVL+)	NEG	NEG	NEG	NEG	NEG	NEG	NEG	NEG	NEG	NEG
**Simulated hybridisations for sequences from Montelongo et al., 2022**											
**CC1**											
**JAEOUR Staphylococcus aureus strain AA1**	CC1-MRSA-[V+fus+tir+ccrAB1]	NEG	POS	NEG	NEG	NEG	NEG	NEG	NEG	POS	NEG
**JAEOWK Staphylococcus aureus strain AA78**	CC1-MRSA-[V+fus+tir+ccrAB1]	NEG	POS	NEG	NEG	NEG	NEG	NEG	NEG	POS	NEG
**JAEOVR Staphylococcus aureus strain AA51**	CC1-MRSA-[V+fus+tir+ccrAB1] (PVL+)	NEG	POS	NEG	NEG	NEG	NEG	NEG	NEG	POS	NEG
**JAEOWE Staphylococcus aureus strain AA67**	CC1-MRSA-[V+fus+tir+ccrAB1] (PVL+)	NEG	POS	NEG	NEG	NEG	NEG	NEG	NEG	POS	NEG
**JAEOWG Staphylococcus aureus strain AA69**	CC1-MRSA-[V+fus+tir+ccrAB1] (PVL+)	NEG	POS	NEG	NEG	NEG	NEG	NEG	NEG	POS	NEG
**JAEOWJ Staphylococcus aureus strain AA77**	CC1-MRSA-[V+fus+tir+ccrAB1] (PVL+)	NEG	POS	NEG	NEG	NEG	NEG	NEG	NEG	POS	NEG
**CC5**											
**JAEOWI Staphylococcus aureus strain AA76**	CC5-MRSA-[V+cas], WA MRSA-123	NEG	NEG	NEG	NEG	NEG	NEG	NEG	NEG	NEG	NEG
**JAEOWN Staphylococcus aureus strain AA80**	CC5-MRSA-[V+cas], WA MRSA-123	NEG	NEG	NEG	POS	NEG	NEG	NEG	NEG	NEG	NEG
**JAEOWH Staphylococcus aureus strain AA70**	CC5-MRSA-[VI+fus+tir+, dcs-]	NEG	NEG	NEG	POS	NEG	NEG	POS	NEG	NEG	POS
**CC6**											
**JAEOVF Staphylococcus aureus strain AA30**	CC6-MRSA-IVa, WA MRSA-51	NEG	POS	NEG	NEG	NEG	NEG	NEG	NEG	NEG	NEG
**CC80**											
**JAEOVM Staphylococcus aureus strain AA4**	CC80-MRSA-IVc (PVL-) [aphA3/sat+]:[far1+], contaminated	NEG	POS	NEG	NEG	NEG	NEG	NEG	NEG	NEG	NEG
**JAEOUZ Staphylococcus aureus strain AA2**	CC80-MRSA-IVc (PVL+) [aphA3/sat+]:[far1+]	NEG	NEG	NEG	NEG	NEG	NEG	NEG	NEG	NEG	NEG
**JAEOVE Staphylococcus aureus strain AA3**	CC80-MRSA-IVc (PVL+) [aphA3/sat+]:[far1+]	NEG	NEG	NEG	NEG	NEG	NEG	NEG	NEG	NEG	NEG
**JAEOVO Staphylococcus aureus strain AA45**	CC80-MRSA-IVc (PVL+) [aphA3/sat+]:[far1+]	NEG	NEG	NEG	NEG	NEG	NEG	NEG	NEG	NEG	NEG
**CC88**											
**JAEOVT Staphylococcus aureus strain AA53**	CC88-MRSA-IV, contaminated	NEG	NEG	NEG	NEG	NEG	NEG	NEG	NEG	NEG	NEG
**CC97**											
**JAEOVL Staphylococcus aureus strain AA39**	CC97-MRSA-IVc, WA MRSA-54/63	NEG	NEG	NEG	NEG	NEG	NEG	NEG	NEG	NEG	NEG
**JAEOVX Staphylococcus aureus strain AA6**	CC97-MRSA-IVc, WA MRSA-54/63	NEG	NEG	NEG	NEG	NEG	NEG	NEG	NEG	NEG	NEG
**JAEOWM Staphylococcus aureus strain AA8**	CC97-MRSA-IVc, WA MRSA-54/63	NEG	NEG	NEG	NEG	NEG	NEG	NEG	NEG	NEG	NEG
**JAEOUU Staphylococcus aureus strain AA104**	CC97-MRSA-V	NEG	NEG	NEG	NEG	NEG	NEG	NEG	NEG	NEG	NEG
**JAEOVJ Staphylococcus aureus strain AA35**	CC97-MRSA-[V+fus]	NEG	NEG	NEG	NEG	NEG	NEG	NEG	NEG	NEG	NEG
**JAEOVK Staphylococcus aureus strain AA36**	CC97-MRSA-[V+fus]	NEG	NEG	NEG	NEG	NEG	NEG	NEG	NEG	NEG	NEG
**CC22**											
**JAEOUY Staphylococcus aureus strain AA18**	CC22-MRSA-IVa (dcs negatives) (tst1+), "Gaza Epidemic Strain"	POS	NEG	NEG	NEG	NEG	NEG	NEG	NEG	NEG	NEG
**JAEOVH Staphylococcus aureus strain AA32**	CC22-MRSA-IVa (dcs negatives) (tst1+), "Gaza Epidemic Strain"	POS	NEG	NEG	NEG	NEG	NEG	NEG	NEG	NEG	NEG
**JAEOVQ Staphylococcus aureus strain AA5**	CC22-MRSA-IVa (dcs negatives) (tst1+), "Gaza Epidemic Strain"	POS	NEG	NEG	NEG	NEG	NEG	NEG	NEG	NEG	NEG
**CC152**											
**JAEOUX Staphylococcus aureus strain AA17**	CC152-MRSA-[V+fus]	NEG	NEG	NEG	NEG	NEG	NEG	NEG	NEG	NEG	NEG
**CC239**											
**JAEOVI Staphylococcus aureus strain AA33**	CC239-MRSA-[III+Cd/Hg+ccrC] (sasX-positive), "Southeast Asian Clade"	NEG	POS	NEG	NEG	NEG	NEG	NEG	NEG	NEG	NEG
**JAEOUS Staphylococcus aureus strain AA101**	CC239-MRSA-[III+Cd+ccrC] (sasX-negat.), "Middle Eastern Cluster"	NEG	POS	NEG	NEG	NEG	NEG	NEG	NEG	NEG	NEG
**JAEOUV Staphylococcus aureus strain AA13**	CC239-MRSA-[III+Cd+ccrC] (sasX-negat.), "Middle Eastern Cluster"	NEG	POS	NEG	NEG	NEG	NEG	NEG	NEG	NEG	NEG
**JAEOUW Staphylococcus aureus strain AA14**	CC239-MRSA-[III+Cd+ccrC] (sasX-negat.), "Middle Eastern Cluster"	NEG	POS	NEG	NEG	NEG	NEG	NEG	NEG	NEG	NEG
**JAEOVA Staphylococcus aureus strain AA22**	CC239-MRSA-[III+Cd+ccrC] (sasX-negat.), "Middle Eastern Cluster"	NEG	POS	NEG	NEG	NEG	NEG	NEG	NEG	NEG	NEG
**JAEOVB Staphylococcus aureus strain AA23**	CC239-MRSA-[III+Cd+ccrC] (sasX-negat.), "Middle Eastern Cluster"	NEG	POS	NEG	NEG	NEG	NEG	NEG	NEG	NEG	NEG
**JAEOVC Staphylococcus aureus strain AA27**	CC239-MRSA-[III+Cd+ccrC] (sasX-negat.), "Middle Eastern Cluster"	NEG	POS	NEG	NEG	NEG	NEG	NEG	NEG	NEG	NEG
**JAEOVD Staphylococcus aureus strain AA29**	CC239-MRSA-[III+Cd+ccrC] (sasX-negat.), "Middle Eastern Cluster"	NEG	POS	NEG	NEG	NEG	NEG	NEG	NEG	NEG	NEG
**JAEOVG Staphylococcus aureus strain AA31**	CC239-MRSA-[III+Cd+ccrC] (sasX-negat.), "Middle Eastern Cluster"	NEG	POS	NEG	NEG	NEG	NEG	NEG	NEG	NEG	NEG
**JAEOVP Staphylococcus aureus strain AA46**	CC239-MRSA-[III+Cd+ccrC] (sasX-negat.), "Middle Eastern Cluster"	NEG	POS	NEG	NEG	NEG	NEG	NEG	NEG	NEG	NEG
**JAEOVS Staphylococcus aureus strain AA52**	CC239-MRSA-[III+Cd+ccrC] (sasX-negat.), "Middle Eastern Cluster"	NEG	POS	NEG	NEG	NEG	NEG	NEG	NEG	NEG	NEG
**JAEOVU Staphylococcus aureus strain AA55**	CC239-MRSA-[III+Cd+ccrC] (sasX-negat.), "Middle Eastern Cluster"	NEG	POS	NEG	NEG	NEG	NEG	NEG	NEG	NEG	NEG
**JAEOVV Staphylococcus aureus strain AA57**	CC239-MRSA-[III+Cd+ccrC] (sasX-negat.), "Middle Eastern Cluster"	NEG	POS	NEG	NEG	NEG	NEG	NEG	NEG	NEG	NEG
**JAEOVY Staphylococcus aureus strain AA60**	CC239-MRSA-[III+Cd+ccrC] (sasX-negat.), "Middle Eastern Cluster"	NEG	POS	NEG	NEG	NEG	NEG	NEG	NEG	NEG	NEG
**JAEOVZ Staphylococcus aureus strain AA61**	CC239-MRSA-[III+Cd+ccrC] (sasX-negat.), "Middle Eastern Cluster"	NEG	POS	NEG	NEG	NEG	NEG	NEG	NEG	NEG	NEG
**JAEOWA Staphylococcus aureus strain AA62**	CC239-MRSA-[III+Cd+ccrC] (sasX-negat.), "Middle Eastern Cluster"	NEG	POS	NEG	NEG	NEG	NEG	NEG	NEG	NEG	NEG
**JAEOWB Staphylococcus aureus strain AA63**	CC239-MRSA-[III+Cd+ccrC] (sasX-negat.), "Middle Eastern Cluster"	NEG	POS	NEG	NEG	NEG	NEG	NEG	NEG	NEG	NEG
**JAEOWL Staphylococcus aureus strain AA79**	CC239-MRSA-[III+Cd+ccrC] (sasX-negat.), "Middle Eastern Cluster"	NEG	POS	NEG	NEG	NEG	NEG	NEG	NEG	NEG	NEG
**JAEOWQ Staphylococcus aureus strain AA92**	CC239-MRSA-[III+Cd+ccrC] (sasX-negat.), "Middle Eastern Cluster"	NEG	POS	NEG	NEG	NEG	NEG	NEG	NEG	NEG	NEG
**JAEOWC Staphylococcus aureus strain AA64**	CC239-MRSA-[mec III+Cd], sasX-negative	NEG	POS	NEG	NEG	NEG	NEG	NEG	NEG	NEG	NEG
**JAEOWP Staphylococcus aureus strain AA91**	CC239-MRSA-[mec III+Cd], sasX-negative	NEG	POS	NEG	NEG	NEG	NEG	NEG	NEG	NEG	NEG

Isolate	Strain assignment	VIRULENCE : SUPERANTIGENS											
		seK	seL	seQ	seR	egc (total)	seg	sei	selm	sen	selo	selu	ORF CM14
		Enterotoxin K	Enterotoxin L	Enterotoxin Q	Enterotoxin R	egc cluster	Enterotoxin G	Enterotoxin I	Enterotoxin M	Enterotoxin N	Enterotoxin O	Enterotoxin U and/or Y	Enterotoxin-like protein ORF CM14
**CC1**													
**Alexandria University_2020-17**	CC1-MRSA-[V+fus+tir]	POS	NEG	POS	NEG	NEG	NEG	NEG	NEG	NEG	NEG	NEG	NEG
**Alexandria University_2020-03**	CC1-MRSA-[V+fus+tir+ccrAB1]	POS	NEG	POS	NEG	NEG	NEG	NEG	NEG	NEG	NEG	NEG	NEG
**Alexandria University_2020-19**	CC1-MRSA-[V+fus+tir+ccrAB1]	POS	NEG	POS	NEG	NEG	NEG	NEG	NEG	NEG	NEG	NEG	NEG
**Alexandria University_2020-21**	CC1-MRSA-[V+fus+tir+ccrAB1]	POS	NEG	POS	NEG	NEG	NEG	NEG	NEG	NEG	NEG	NEG	NEG
**Alexandria University_2020-09**	CC1-MRSA-[V+fus+tir+ccrAB1] (PVL+)	POS	NEG	POS	NEG	NEG	NEG	NEG	NEG	NEG	NEG	NEG	NEG
**Alexandria University_2020-14**	CC1-MRSA-[V+fus+tir+ccrAB1] (PVL+)	POS	NEG	POS	NEG	NEG	NEG	NEG	NEG	NEG	NEG	NEG	NEG
**Alexandria University_2020-24**	CC1-MRSA-[V+fus+tir+ccrAB1] (PVL+)	POS	NEG	POS	NEG	NEG	NEG	NEG	NEG	NEG	NEG	NEG	NEG
**Alexandria University_2020-27**	CC1-MRSA-[V+fus+tir+ccrAB1] (PVL+)	POS	NEG	POS	NEG	NEG	NEG	NEG	NEG	NEG	NEG	NEG	NEG
**Alexandria University_2020-28**	CC1-MRSA-[V+fus+tir+ccrAB1] (PVL+)	POS	NEG	POS	NEG	NEG	NEG	NEG	NEG	NEG	NEG	NEG	NEG
**CC5**													
**Alexandria University_2020-12**	CC5-MRSA-[VI+fus+tir]	NEG	NEG	NEG	POS	POS	POS	POS	POS	POS	POS	POS	NEG
**Alexandria University_2020-20**	CC5-MRSA-[VI+fus+tir]	NEG	NEG	NEG	NEG	POS	POS	POS	POS	POS	POS	POS	NEG
**CC6**													
**Alexandria University_2020-29**	CC6-MRSA-[V+fus]	POS	NEG	POS	NEG	NEG	NEG	NEG	NEG	NEG	NEG	NEG	NEG
**CC15**													
**Alexandria University_2020-01**	CC15-MRSA-[V+fus]	NEG	NEG	NEG	NEG	NEG	NEG	NEG	NEG	NEG	NEG	NEG	NEG
**Alexandria University_2020-02**	CC15-MRSA-[V+fus]	NEG	NEG	NEG	NEG	NEG	NEG	NEG	NEG	NEG	NEG	NEG	NEG
**Alexandria University_2020-05**	CC15-MRSA-[V+fus]	NEG	NEG	NEG	NEG	NEG	NEG	NEG	NEG	NEG	NEG	NEG	NEG
**Alexandria University_2020-15**	CC15-MRSA-[V+fus]	NEG	NEG	NEG	NEG	NEG	NEG	NEG	NEG	NEG	NEG	NEG	NEG
**Alexandria University_2020-22**	CC15-MRSA-[V+fus]	NEG	NEG	NEG	NEG	NEG	NEG	NEG	NEG	NEG	NEG	NEG	NEG
**Alexandria University_2020-32**	CC15-MRSA-[V+fus]	NEG	NEG	NEG	NEG	NEG	NEG	NEG	NEG	NEG	NEG	NEG	NEG
**CC22**													
**Alexandria University_2020-04**	CC22-MRSA-IVa (dcs negatives) (tst1+), "Gaza Epidemic Strain"	NEG	NEG	NEG	NEG	POS	POS	POS	POS	POS	POS	POS	NEG
**Alexandria University_2020-11**	CC22-MRSA-IVa (dcs negatives) (tst1+), "Gaza Epidemic Strain"	NEG	NEG	NEG	NEG	POS	POS	POS	POS	POS	POS	POS	NEG
**CC30**													
**Alexandria University_2020-26**	CC30-MRSA-IVa (PVL+), "WSPP/Southwest Pacific Clone"	NEG	NEG	NEG	NEG	POS	POS	POS	POS	POS	POS	POS	NEG
**CC97**													
**Alexandria University_2020-30**	CC97-MRSA-[V+fus]	NEG	NEG	NEG	NEG	NEG	NEG	NEG	NEG	NEG	NEG	NEG	NEG
**CC121**													
**Alexandria University_2020-06**	CC121-MRSA-[V+fus] (PVL+)	NEG	NEG	NEG	NEG	POS	POS	AMB	POS	POS	POS	POS	POS
**CC152**													
**Alexandria University_2020-18**	CC152-MRSA-[V+fus]	NEG	NEG	NEG	NEG	NEG	NEG	NEG	NEG	NEG	NEG	NEG	NEG
**Alexandria University_2020-31**	CC152-MRSA-[V+fus]	NEG	NEG	NEG	NEG	NEG	NEG	NEG	NEG	NEG	NEG	NEG	NEG
**CC239**													
**Alexandria University_2020-13**	CC239-MRSA-[III+Cd+ccrC] (sasX-negat.), "Middle Eastern Cluster"	POS	NEG	POS	NEG	NEG	NEG	NEG	NEG	NEG	NEG	NEG	NEG
**Alexandria University_2020-16**	CC239-MRSA-[III+Cd], sasX-negative	POS	NEG	POS	NEG	NEG	NEG	NEG	NEG	NEG	NEG	NEG	NEG
**CC1153**													
**Alexandria University_2020-07**	CC1153-MRSA-[V+fus] (PVL+)	NEG	NEG	NEG	NEG	NEG	NEG	NEG	NEG	NEG	NEG	NEG	NEG
**Alexandria University_2020-08**	CC1153-MRSA-[V+fus] (PVL+)	NEG	NEG	NEG	NEG	NEG	NEG	NEG	NEG	NEG	NEG	NEG	NEG
**Alexandria University_2020-23**	CC1153-MRSA-[V+fus] (PVL+)	NEG	NEG	NEG	NEG	NEG	NEG	NEG	NEG	NEG	NEG	NEG	NEG
**Simulated hybridisations for sequences from Montelongo et al., 2022**													
**CC1**													
**JAEOUR Staphylococcus aureus strain AA1**	CC1-MRSA-[V+fus+tir+ccrAB1]	POS	NEG	POS	NEG	NEG	NEG	NEG	NEG	NEG	NEG	NEG	NEG
**JAEOWK Staphylococcus aureus strain AA78**	CC1-MRSA-[V+fus+tir+ccrAB1]	POS	NEG	POS	NEG	NEG	NEG	NEG	NEG	NEG	NEG	NEG	NEG
**JAEOVR Staphylococcus aureus strain AA51**	CC1-MRSA-[V+fus+tir+ccrAB1] (PVL+)	POS	NEG	POS	NEG	NEG	NEG	NEG	NEG	NEG	NEG	NEG	NEG
**JAEOWE Staphylococcus aureus strain AA67**	CC1-MRSA-[V+fus+tir+ccrAB1] (PVL+)	POS	NEG	POS	NEG	NEG	NEG	NEG	NEG	NEG	NEG	NEG	NEG
**JAEOWG Staphylococcus aureus strain AA69**	CC1-MRSA-[V+fus+tir+ccrAB1] (PVL+)	POS	NEG	POS	NEG	NEG	NEG	NEG	NEG	NEG	NEG	NEG	NEG
**JAEOWJ Staphylococcus aureus strain AA77**	CC1-MRSA-[V+fus+tir+ccrAB1] (PVL+)	POS	NEG	POS	NEG	NEG	NEG	NEG	NEG	NEG	NEG	NEG	NEG
**CC5**													
**JAEOWI Staphylococcus aureus strain AA76**	CC5-MRSA-[V+cas], WA MRSA-123	NEG	NEG	NEG	NEG	POS	POS	POS	POS	POS	POS	POS	NEG
**JAEOWN Staphylococcus aureus strain AA80**	CC5-MRSA-[V+cas], WA MRSA-123	NEG	NEG	NEG	NEG	POS	POS	POS	POS	POS	POS	POS	NEG
**JAEOWH Staphylococcus aureus strain AA70**	CC5-MRSA-[VI+fus+tir+, dcs-]	NEG	NEG	NEG	POS	POS	POS	POS	POS	POS	POS	POS	NEG
**CC6**													
**JAEOVF Staphylococcus aureus strain AA30**	CC6-MRSA-IVa, WA MRSA-51	NEG	NEG	NEG	NEG	NEG	NEG	NEG	NEG	NEG	NEG	NEG	NEG
**CC80**													
**JAEOVM Staphylococcus aureus strain AA4**	CC80-MRSA-IVc (PVL-) [aphA3/sat+]:[far1+], contaminated	POS	NEG	NEG	NEG	NEG	NEG	NEG	NEG	NEG	NEG	NEG	NEG
**JAEOUZ Staphylococcus aureus strain AA2**	CC80-MRSA-IVc (PVL+) [aphA3/sat+]:[far1+]	NEG	NEG	NEG	NEG	NEG	NEG	NEG	NEG	NEG	NEG	NEG	NEG
**JAEOVE Staphylococcus aureus strain AA3**	CC80-MRSA-IVc (PVL+) [aphA3/sat+]:[far1+]	NEG	NEG	NEG	NEG	NEG	NEG	NEG	NEG	NEG	NEG	NEG	NEG
**JAEOVO Staphylococcus aureus strain AA45**	CC80-MRSA-IVc (PVL+) [aphA3/sat+]:[far1+]	NEG	NEG	NEG	NEG	NEG	NEG	NEG	NEG	NEG	NEG	NEG	NEG
**CC88**													
**JAEOVT Staphylococcus aureus strain AA53**	CC88-MRSA-IV, contaminated	NEG	NEG	NEG	NEG	NEG	NEG	NEG	NEG	NEG	NEG	NEG	NEG
**CC97**													
**JAEOVL Staphylococcus aureus strain AA39**	CC97-MRSA-IVc, WA MRSA-54/63	NEG	NEG	NEG	NEG	NEG	NEG	NEG	NEG	NEG	NEG	NEG	NEG
**JAEOVX Staphylococcus aureus strain AA6**	CC97-MRSA-IVc, WA MRSA-54/63	NEG	NEG	NEG	NEG	NEG	NEG	NEG	NEG	NEG	NEG	NEG	NEG
**JAEOWM Staphylococcus aureus strain AA8**	CC97-MRSA-IVc, WA MRSA-54/63	NEG	NEG	NEG	NEG	NEG	NEG	NEG	NEG	NEG	NEG	NEG	NEG
**JAEOUU Staphylococcus aureus strain AA104**	CC97-MRSA-V	NEG	NEG	NEG	NEG	NEG	NEG	NEG	NEG	NEG	NEG	NEG	NEG
**JAEOVJ Staphylococcus aureus strain AA35**	CC97-MRSA-[V+fus]	NEG	NEG	NEG	NEG	NEG	NEG	NEG	NEG	NEG	NEG	NEG	NEG
**JAEOVK Staphylococcus aureus strain AA36**	CC97-MRSA-[V+fus]	NEG	NEG	NEG	NEG	NEG	NEG	NEG	NEG	NEG	NEG	NEG	NEG
**CC22**													
**JAEOUY Staphylococcus aureus strain AA18**	CC22-MRSA-IVa (dcs negatives) (tst1+), "Gaza Epidemic Strain"	NEG	NEG	NEG	NEG	POS	POS	POS	POS	POS	POS	POS	NEG
**JAEOVH Staphylococcus aureus strain AA32**	CC22-MRSA-IVa (dcs negatives) (tst1+), "Gaza Epidemic Strain"	NEG	NEG	NEG	NEG	POS	POS	POS	POS	POS	POS	POS	NEG
**JAEOVQ Staphylococcus aureus strain AA5**	CC22-MRSA-IVa (dcs negatives) (tst1+), "Gaza Epidemic Strain"	NEG	NEG	NEG	NEG	POS	POS	POS	POS	POS	POS	POS	NEG
**CC152**													
**JAEOUX Staphylococcus aureus strain AA17**	CC152-MRSA-[V+fus]	NEG	NEG	NEG	NEG	NEG	NEG	NEG	NEG	NEG	NEG	NEG	NEG
**CC239**													
**JAEOVI Staphylococcus aureus strain AA33**	CC239-MRSA-[III+Cd/Hg+ccrC] (sasX-positive), "Southeast Asian Clade"	POS	NEG	POS	NEG	NEG	NEG	NEG	NEG	NEG	NEG	NEG	NEG
**JAEOUS Staphylococcus aureus strain AA101**	CC239-MRSA-[III+Cd+ccrC] (sasX-negat.), "Middle Eastern Cluster"	POS	NEG	POS	NEG	NEG	NEG	NEG	NEG	NEG	NEG	NEG	NEG
**JAEOUV Staphylococcus aureus strain AA13**	CC239-MRSA-[III+Cd+ccrC] (sasX-negat.), "Middle Eastern Cluster"	POS	NEG	POS	NEG	NEG	NEG	NEG	NEG	NEG	NEG	NEG	NEG
**JAEOUW Staphylococcus aureus strain AA14**	CC239-MRSA-[III+Cd+ccrC] (sasX-negat.), "Middle Eastern Cluster"	POS	NEG	POS	NEG	NEG	NEG	NEG	NEG	NEG	NEG	NEG	NEG
**JAEOVA Staphylococcus aureus strain AA22**	CC239-MRSA-[III+Cd+ccrC] (sasX-negat.), "Middle Eastern Cluster"	POS	NEG	POS	NEG	NEG	NEG	NEG	NEG	NEG	NEG	NEG	NEG
**JAEOVB Staphylococcus aureus strain AA23**	CC239-MRSA-[III+Cd+ccrC] (sasX-negat.), "Middle Eastern Cluster"	POS	NEG	POS	NEG	NEG	NEG	NEG	NEG	NEG	NEG	NEG	NEG
**JAEOVC Staphylococcus aureus strain AA27**	CC239-MRSA-[III+Cd+ccrC] (sasX-negat.), "Middle Eastern Cluster"	POS	NEG	POS	NEG	NEG	NEG	NEG	NEG	NEG	NEG	NEG	NEG
**JAEOVD Staphylococcus aureus strain AA29**	CC239-MRSA-[III+Cd+ccrC] (sasX-negat.), "Middle Eastern Cluster"	POS	NEG	POS	NEG	NEG	NEG	NEG	NEG	NEG	NEG	NEG	NEG
**JAEOVG Staphylococcus aureus strain AA31**	CC239-MRSA-[III+Cd+ccrC] (sasX-negat.), "Middle Eastern Cluster"	POS	NEG	POS	NEG	NEG	NEG	NEG	NEG	NEG	NEG	NEG	NEG
**JAEOVP Staphylococcus aureus strain AA46**	CC239-MRSA-[III+Cd+ccrC] (sasX-negat.), "Middle Eastern Cluster"	POS	NEG	POS	NEG	NEG	NEG	NEG	NEG	NEG	NEG	NEG	NEG
**JAEOVS Staphylococcus aureus strain AA52**	CC239-MRSA-[III+Cd+ccrC] (sasX-negat.), "Middle Eastern Cluster"	POS	NEG	POS	NEG	NEG	NEG	NEG	NEG	NEG	NEG	NEG	NEG
**JAEOVU Staphylococcus aureus strain AA55**	CC239-MRSA-[III+Cd+ccrC] (sasX-negat.), "Middle Eastern Cluster"	POS	NEG	POS	NEG	NEG	NEG	NEG	NEG	NEG	NEG	NEG	NEG
**JAEOVV Staphylococcus aureus strain AA57**	CC239-MRSA-[III+Cd+ccrC] (sasX-negat.), "Middle Eastern Cluster"	POS	NEG	POS	NEG	NEG	NEG	NEG	NEG	NEG	NEG	NEG	NEG
**JAEOVY Staphylococcus aureus strain AA60**	CC239-MRSA-[III+Cd+ccrC] (sasX-negat.), "Middle Eastern Cluster"	POS	NEG	POS	NEG	NEG	NEG	NEG	NEG	NEG	NEG	NEG	NEG
**JAEOVZ Staphylococcus aureus strain AA61**	CC239-MRSA-[III+Cd+ccrC] (sasX-negat.), "Middle Eastern Cluster"	POS	NEG	POS	NEG	NEG	NEG	NEG	NEG	NEG	NEG	NEG	NEG
**JAEOWA Staphylococcus aureus strain AA62**	CC239-MRSA-[III+Cd+ccrC] (sasX-negat.), "Middle Eastern Cluster"	POS	NEG	POS	NEG	NEG	NEG	NEG	NEG	NEG	NEG	NEG	NEG
**JAEOWB Staphylococcus aureus strain AA63**	CC239-MRSA-[III+Cd+ccrC] (sasX-negat.), "Middle Eastern Cluster"	POS	NEG	POS	NEG	NEG	NEG	NEG	NEG	NEG	NEG	NEG	NEG
**JAEOWL Staphylococcus aureus strain AA79**	CC239-MRSA-[III+Cd+ccrC] (sasX-negat.), "Middle Eastern Cluster"	POS	NEG	POS	NEG	NEG	NEG	NEG	NEG	NEG	NEG	NEG	NEG
**JAEOWQ Staphylococcus aureus strain AA92**	CC239-MRSA-[III+Cd+ccrC] (sasX-negat.), "Middle Eastern Cluster"	POS	NEG	POS	NEG	NEG	NEG	NEG	NEG	NEG	NEG	NEG	NEG
**JAEOWC Staphylococcus aureus strain AA64**	CC239-MRSA-[mec III+Cd], sasX-negative	POS	NEG	POS	NEG	NEG	NEG	NEG	NEG	NEG	NEG	NEG	NEG
**JAEOWP Staphylococcus aureus strain AA91**	CC239-MRSA-[mec III+Cd], sasX-negative	POS	NEG	POS	NEG	NEG	NEG	NEG	NEG	NEG	NEG	NEG	NEG

Isolate	Strain assignment	VIRULENCE : HLG AND LEUKOCIDINS												
		lukF	lukS		hlgA	lukF-PV	lukS-PV	lukF-PV (P83)	lukM	lukD	lukE	lukX	lukY	
			lukS	lukS (ST22+ST45)									lukY	lukY (ST30+ST45)
		Haemolysin gamma / leukocidin, component B	Haemolysin gamma / leukocidin, component C		Haemolysin gamma	Panton Valentine leukocidin F component	Panton Valentine leukocidin S component	F component from ruminant leukocidin	S component from ruminant leukocidin	leukocidin D component	leukocidin E component	leukocidin/ haemolysin toxin family protein	leukocidin/haemolysin toxin family protein	
**CC1**														
Alexandria University_2020-17	CC1-MRSA-[V+fus+tir]	POS	POS	AMB	POS	NEG	NEG	NEG	NEG	POS	POS	POS	POS	NEG
Alexandria University_2020-03	CC1-MRSA-[V+fus+tir+ccrAB1]	POS	POS	AMB	POS	NEG	NEG	NEG	NEG	POS	POS	POS	POS	NEG
Alexandria University_2020-19	CC1-MRSA-[V+fus+tir+ccrAB1]	POS	POS	NEG	POS	NEG	NEG	NEG	NEG	POS	POS	AMB	POS	NEG
Alexandria University_2020-21	CC1-MRSA-[V+fus+tir+ccrAB1]	POS	POS	AMB	POS	NEG	NEG	NEG	NEG	POS	POS	POS	POS	NEG
Alexandria University_2020-09	CC1-MRSA-[V+fus+tir+ccrAB1] (PVL+)	POS	POS	AMB	POS	AMB	POS	NEG	NEG	POS	POS	POS	POS	NEG
Alexandria University_2020-14	CC1-MRSA-[V+fus+tir+ccrAB1] (PVL+)	POS	POS	POS	POS	POS	POS	NEG	NEG	POS	POS	POS	POS	NEG
Alexandria University_2020-24	CC1-MRSA-[V+fus+tir+ccrAB1] (PVL+)	POS	POS	POS	POS	POS	POS	NEG	NEG	POS	POS	POS	POS	NEG
Alexandria University_2020-27	CC1-MRSA-[V+fus+tir+ccrAB1] (PVL+)	POS	POS	AMB	POS	POS	POS	NEG	NEG	POS	POS	POS	POS	NEG
Alexandria University_2020-28	CC1-MRSA-[V+fus+tir+ccrAB1] (PVL+)	POS	POS	NEG	AMB	NEG	POS	NEG	NEG	POS	AMB	NEG	POS	NEG
**CC5**														
Alexandria University_2020-12	CC5-MRSA-[VI+fus+tir]	POS	POS	POS	POS	NEG	NEG	NEG	NEG	POS	POS	POS	POS	NEG
Alexandria University_2020-20	CC5-MRSA-[VI+fus+tir]	POS	POS	NEG	POS	NEG	NEG	NEG	NEG	POS	POS	POS	POS	NEG
**CC6**														
Alexandria University_2020-29	CC6-MRSA-[V+fus]	POS	POS	AMB	POS	NEG	NEG	NEG	NEG	POS	NEG	POS	POS	NEG
**CC15**														
Alexandria University_2020-01	CC15-MRSA-[V+fus]	POS	POS	NEG	POS	NEG	NEG	NEG	NEG	POS	NEG	AMB	POS	NEG
Alexandria University_2020-02	CC15-MRSA-[V+fus]	POS	POS	NEG	POS	NEG	NEG	NEG	NEG	POS	AMB	POS	POS	NEG
Alexandria University_2020-05	CC15-MRSA-[V+fus]	POS	POS	NEG	POS	NEG	NEG	NEG	NEG	POS	NEG	NEG	POS	NEG
Alexandria University_2020-15	CC15-MRSA-[V+fus]	POS	POS	NEG	POS	NEG	NEG	NEG	NEG	POS	AMB	POS	POS	NEG
Alexandria University_2020-22	CC15-MRSA-[V+fus]	POS	POS	NEG	AMB	NEG	NEG	NEG	NEG	POS	NEG	NEG	POS	NEG
Alexandria University_2020-32	CC15-MRSA-[V+fus]	POS	POS	NEG	POS	NEG	NEG	NEG	NEG	POS	AMB	POS	POS	NEG
**CC22**														
Alexandria University_2020-04	CC22-MRSA-IVa (dcs negatives) (tst1+), "Gaza Epidemic Strain"	AMB	NEG	POS	AMB	NEG	NEG	NEG	NEG	NEG	NEG	AMB	POS	NEG
Alexandria University_2020-11	CC22-MRSA-IVa (dcs negatives) (tst1+), "Gaza Epidemic Strain"	POS	NEG	POS	POS	NEG	NEG	NEG	NEG	NEG	NEG	POS	POS	NEG
**CC30**														
Alexandria University_2020-26	CC30-MRSA-IVa (PVL+), "WSPP/Southwest Pacific Clone"	POS	POS	NEG	POS	AMB	POS	NEG	NEG	NEG	NEG	POS	NEG	POS
**CC97**														
Alexandria University_2020-30	CC97-MRSA-[V+fus]	POS	POS	NEG	POS	NEG	NEG	NEG	NEG	NEG	NEG	POS	POS	NEG
**CC121**														
Alexandria University_2020-06	CC121-MRSA-[V+fus] (PVL+)	POS	POS	POS	POS	POS	POS	NEG	NEG	POS	POS	POS	POS	NEG
**CC152**														
Alexandria University_2020-18	CC152-MRSA-[V+fus]	POS	NEG	NEG	POS	POS	POS	NEG	NEG	NEG	NEG	NEG	POS	NEG
Alexandria University_2020-31	CC152-MRSA-[V+fus]	POS	NEG	NEG	POS	POS	POS	NEG	NEG	NEG	NEG	NEG	POS	NEG
**CC239**														
Alexandria University_2020-13	CC239-MRSA-[III+Cd+ccrC] (sasX-negat.), "Middle Eastern Cluster"	POS	POS	NEG	POS	NEG	NEG	NEG	NEG	POS	POS	POS	POS	NEG
Alexandria University_2020-16	CC239-MRSA-[III+Cd], sasX-negative	POS	POS	NEG	POS	NEG	NEG	NEG	NEG	POS	POS	NEG	POS	NEG
**CC1153**														
Alexandria University_2020-07	CC1153-MRSA-[V+fus] (PVL+)	POS	POS	NEG	AMB	AMB	AMB	NEG	NEG	NEG	NEG	AMB	POS	NEG
Alexandria University_2020-08	CC1153-MRSA-[V+fus] (PVL+)	POS	POS	NEG	POS	AMB	POS	NEG	NEG	NEG	NEG	AMB	POS	NEG
Alexandria University_2020-23	CC1153-MRSA-[V+fus] (PVL+)	POS	POS	AMB	POS	POS	POS	NEG	NEG	NEG	NEG	POS	POS	NEG
**Simulated hybridisations for sequences from Montelongo et al., 2022**														
**CC1**														
JAEOUR Staphylococcus aureus strain AA1	CC1-MRSA-[V+fus+tir+ccrAB1]	POS	POS	AMB	POS	NEG	NEG	NEG	NEG	POS	POS	POS	POS	NEG
JAEOWK Staphylococcus aureus strain AA78	CC1-MRSA-[V+fus+tir+ccrAB1]	POS	POS	AMB	POS	NEG	NEG	NEG	NEG	POS	POS	POS	POS	NEG
JAEOVR Staphylococcus aureus strain AA51	CC1-MRSA-[V+fus+tir+ccrAB1] (PVL+)	POS	POS	AMB	POS	POS	POS	NEG	NEG	POS	POS	POS	POS	NEG
JAEOWE Staphylococcus aureus strain AA67	CC1-MRSA-[V+fus+tir+ccrAB1] (PVL+)	POS	POS	AMB	POS	POS	POS	NEG	NEG	POS	POS	POS	POS	NEG
JAEOWG Staphylococcus aureus strain AA69	CC1-MRSA-[V+fus+tir+ccrAB1] (PVL+)	POS	POS	AMB	POS	POS	POS	NEG	NEG	POS	POS	POS	POS	NEG
JAEOWJ Staphylococcus aureus strain AA77	CC1-MRSA-[V+fus+tir+ccrAB1] (PVL+)	POS	POS	AMB	POS	POS	POS	NEG	NEG	POS	POS	POS	POS	NEG
**CC5**														
JAEOWI Staphylococcus aureus strain AA76	CC5-MRSA-[V+cas], WA MRSA-123	POS	POS	AMB	POS	NEG	NEG	NEG	NEG	POS	POS	POS	AMB	NEG
JAEOWN Staphylococcus aureus strain AA80	CC5-MRSA-[V+cas], WA MRSA-123	POS	POS	AMB	POS	NEG	NEG	NEG	NEG	POS	POS	POS	AMB	NEG
JAEOWH Staphylococcus aureus strain AA70	CC5-MRSA-[VI+fus+tir+, dcs-]	POS	POS	AMB	POS	NEG	NEG	NEG	NEG	POS	POS	POS	AMB	NEG
**CC6**														
JAEOVF Staphylococcus aureus strain AA30	CC6-MRSA-IVa, WA MRSA-51	POS	POS	AMB	POS	NEG	NEG	NEG	NEG	POS	POS	POS	AMB	NEG
**CC80**														
JAEOVM Staphylococcus aureus strain AA4	CC80-MRSA-IVc (PVL-) [aphA3/sat+]:[far1+], contaminated	POS	POS	AMB	POS	POS	POS	NEG	NEG	POS	POS	POS	AMB	NEG
JAEOUZ Staphylococcus aureus strain AA2	CC80-MRSA-IVc (PVL+) [aphA3/sat+]:[far1+]	POS	POS	AMB	POS	POS	POS	NEG	NEG	POS	POS	POS	AMB	NEG
JAEOVE Staphylococcus aureus strain AA3	CC80-MRSA-IVc (PVL+) [aphA3/sat+]:[far1+]	POS	POS	AMB	POS	POS	POS	NEG	NEG	POS	POS	POS	AMB	NEG
JAEOVO Staphylococcus aureus strain AA45	CC80-MRSA-IVc (PVL+) [aphA3/sat+]:[far1+]	POS	POS	AMB	POS	NEG	NEG	NEG	NEG	POS	POS	POS	AMB	NEG
**CC88**														
JAEOVT Staphylococcus aureus strain AA53	CC88-MRSA-IV, contaminated	POS	POS	AMB	POS	NEG	NEG	NEG	NEG	AMB	POS	POS	AMB	NEG
**CC97**														
JAEOVL Staphylococcus aureus strain AA39	CC97-MRSA-IVc, WA MRSA-54/63	POS	POS	AMB	POS	NEG	NEG	NEG	NEG	AMB	POS	POS	AMB	NEG
JAEOVX Staphylococcus aureus strain AA6	CC97-MRSA-IVc, WA MRSA-54/63	POS	POS	AMB	POS	NEG	NEG	NEG	NEG	AMB	POS	POS	AMB	NEG
JAEOWM Staphylococcus aureus strain AA8	CC97-MRSA-IVc, WA MRSA-54/63	POS	POS	AMB	POS	NEG	NEG	NEG	NEG	AMB	POS	POS	AMB	NEG
JAEOUU Staphylococcus aureus strain AA104	CC97-MRSA-V	POS	POS	AMB	POS	NEG	NEG	NEG	NEG	AMB	POS	POS	AMB	NEG
JAEOVJ Staphylococcus aureus strain AA35	CC97-MRSA-[V+fus]	POS	POS	AMB	POS	NEG	NEG	NEG	NEG	NEG	NEG	POS	AMB	NEG
JAEOVK Staphylococcus aureus strain AA36	CC97-MRSA-[V+fus]	POS	POS	AMB	POS	NEG	NEG	NEG	NEG	AMB	POS	POS	AMB	NEG
**CC22**														
JAEOUY Staphylococcus aureus strain AA18	CC22-MRSA-IVa (dcs negatives) (tst1+), "Gaza Epidemic Strain"	AMB	NEG	POS	POS	NEG	NEG	NEG	NEG	NEG	NEG	POS	AMB	NEG
JAEOVH Staphylococcus aureus strain AA32	CC22-MRSA-IVa (dcs negatives) (tst1+), "Gaza Epidemic Strain"	AMB	NEG	POS	POS	NEG	NEG	NEG	NEG	NEG	NEG	POS	AMB	NEG
JAEOVQ Staphylococcus aureus strain AA5	CC22-MRSA-IVa (dcs negatives) (tst1+), "Gaza Epidemic Strain"	AMB	NEG	POS	POS	NEG	NEG	NEG	NEG	NEG	NEG	POS	AMB	NEG
**CC152**														
JAEOUX Staphylococcus aureus strain AA17	CC152-MRSA-[V+fus]	AMB	NEG	NEG	NEG	POS	POS	NEG	NEG	AMB	NEG	AMB	AMB	NEG
**CC239**														
JAEOVI Staphylococcus aureus strain AA33	CC239-MRSA-[III+Cd/Hg+ccrC] (sasX-positive), "Southeast Asian Clade"	POS	POS	AMB	POS	NEG	NEG	NEG	NEG	POS	POS	POS	AMB	NEG
JAEOUS Staphylococcus aureus strain AA101	CC239-MRSA-[III+Cd+ccrC] (sasX-negat.), "Middle Eastern Cluster"	POS	POS	AMB	POS	NEG	NEG	NEG	NEG	POS	POS	POS	AMB	NEG
JAEOUV Staphylococcus aureus strain AA13	CC239-MRSA-[III+Cd+ccrC] (sasX-negat.), "Middle Eastern Cluster"	POS	POS	AMB	POS	NEG	NEG	NEG	NEG	POS	POS	POS	AMB	NEG
JAEOUW Staphylococcus aureus strain AA14	CC239-MRSA-[III+Cd+ccrC] (sasX-negat.), "Middle Eastern Cluster"	POS	POS	AMB	POS	NEG	NEG	NEG	NEG	POS	POS	POS	AMB	NEG
JAEOVA Staphylococcus aureus strain AA22	CC239-MRSA-[III+Cd+ccrC] (sasX-negat.), "Middle Eastern Cluster"	POS	POS	AMB	POS	NEG	NEG	NEG	NEG	POS	POS	POS	AMB	NEG
JAEOVB Staphylococcus aureus strain AA23	CC239-MRSA-[III+Cd+ccrC] (sasX-negat.), "Middle Eastern Cluster"	POS	POS	AMB	POS	NEG	NEG	NEG	NEG	POS	POS	POS	AMB	NEG
JAEOVC Staphylococcus aureus strain AA27	CC239-MRSA-[III+Cd+ccrC] (sasX-negat.), "Middle Eastern Cluster"	POS	POS	AMB	POS	NEG	NEG	NEG	NEG	POS	POS	POS	AMB	NEG
JAEOVD Staphylococcus aureus strain AA29	CC239-MRSA-[III+Cd+ccrC] (sasX-negat.), "Middle Eastern Cluster"	POS	POS	AMB	POS	NEG	NEG	NEG	NEG	POS	POS	POS	AMB	NEG
JAEOVG Staphylococcus aureus strain AA31	CC239-MRSA-[III+Cd+ccrC] (sasX-negat.), "Middle Eastern Cluster"	POS	POS	AMB	AMB	NEG	NEG	NEG	NEG	POS	POS	POS	AMB	NEG
JAEOVP Staphylococcus aureus strain AA46	CC239-MRSA-[III+Cd+ccrC] (sasX-negat.), "Middle Eastern Cluster"	POS	POS	AMB	POS	NEG	NEG	NEG	NEG	POS	POS	POS	AMB	NEG
JAEOVS Staphylococcus aureus strain AA52	CC239-MRSA-[III+Cd+ccrC] (sasX-negat.), "Middle Eastern Cluster"	POS	POS	AMB	POS	NEG	NEG	NEG	NEG	POS	POS	POS	AMB	NEG
JAEOVU Staphylococcus aureus strain AA55	CC239-MRSA-[III+Cd+ccrC] (sasX-negat.), "Middle Eastern Cluster"	POS	POS	AMB	POS	NEG	NEG	NEG	NEG	POS	POS	POS	AMB	NEG
JAEOVV Staphylococcus aureus strain AA57	CC239-MRSA-[III+Cd+ccrC] (sasX-negat.), "Middle Eastern Cluster"	POS	POS	AMB	POS	NEG	NEG	NEG	NEG	POS	POS	POS	AMB	NEG
JAEOVY Staphylococcus aureus strain AA60	CC239-MRSA-[III+Cd+ccrC] (sasX-negat.), "Middle Eastern Cluster"	POS	POS	AMB	POS	NEG	NEG	NEG	NEG	POS	POS	POS	AMB	NEG
JAEOVZ Staphylococcus aureus strain AA61	CC239-MRSA-[III+Cd+ccrC] (sasX-negat.), "Middle Eastern Cluster"	POS	POS	AMB	POS	NEG	NEG	NEG	NEG	POS	POS	POS	AMB	NEG
JAEOWA Staphylococcus aureus strain AA62	CC239-MRSA-[III+Cd+ccrC] (sasX-negat.), "Middle Eastern Cluster"	POS	POS	AMB	POS	NEG	NEG	NEG	NEG	POS	POS	POS	AMB	NEG
JAEOWB Staphylococcus aureus strain AA63	CC239-MRSA-[III+Cd+ccrC] (sasX-negat.), "Middle Eastern Cluster"	POS	POS	AMB	POS	NEG	NEG	NEG	NEG	POS	POS	POS	AMB	NEG
JAEOWL Staphylococcus aureus strain AA79	CC239-MRSA-[III+Cd+ccrC] (sasX-negat.), "Middle Eastern Cluster"	POS	POS	AMB	POS	NEG	NEG	NEG	NEG	POS	POS	POS	AMB	NEG
JAEOWQ Staphylococcus aureus strain AA92	CC239-MRSA-[III+Cd+ccrC] (sasX-negat.), "Middle Eastern Cluster"	POS	POS	AMB	POS	NEG	NEG	NEG	NEG	POS	POS	POS	AMB	NEG
JAEOWC Staphylococcus aureus strain AA64	CC239-MRSA-[mec III+Cd], sasX-negative	POS	POS	AMB	POS	NEG	NEG	NEG	NEG	POS	POS	POS	AMB	NEG
JAEOWP Staphylococcus aureus strain AA91	CC239-MRSA-[mec III+Cd], sasX-negative	POS	POS	AMB	POS	NEG	NEG	NEG	NEG	POS	POS	POS	AMB	NEG

Isolate	Strain assignment	VIRULENCE : HAEMOLYSINS								VIRULENCE : HLB-CONV PHAGES		
				hlIII		hlb						
		corB (=hl)	hla	hlIII (cons)	hlIII (other than RF122)	hlb-probe 1	hlb-probe 2	hlb-probe 3	un-truncated hlb	sak	chp	scn
		Putative membrane protein	Haemolysin alpha	Putative membrane protein		haemolysin beta				staphylo-kinase	chemotaxis-inhibiting protein (CHIPS)	Staphyl. Complement inhibitor
**CC1**												
Alexandria University_2020-17	CC1-MRSA-[V+fus+tir]	POS	POS	POS	POS	POS	POS	POS	NEG	POS	NEG	POS
Alexandria University_2020-03	CC1-MRSA-[V+fus+tir+ccrAB1]	POS	POS	POS	POS	POS	POS	POS	NEG	POS	NEG	POS
Alexandria University_2020-19	CC1-MRSA-[V+fus+tir+ccrAB1]	POS	POS	POS	POS	POS	POS	POS	NEG	POS	NEG	AMB
Alexandria University_2020-21	CC1-MRSA-[V+fus+tir+ccrAB1]	POS	POS	POS	POS	POS	POS	POS	NEG	POS	NEG	POS
Alexandria University_2020-09	CC1-MRSA-[V+fus+tir+ccrAB1] (PVL+)	POS	POS	POS	POS	POS	POS	POS	NEG	POS	NEG	POS
Alexandria University_2020-14	CC1-MRSA-[V+fus+tir+ccrAB1] (PVL+)	POS	POS	POS	POS	POS	POS	POS	NEG	POS	NEG	POS
Alexandria University_2020-24	CC1-MRSA-[V+fus+tir+ccrAB1] (PVL+)	POS	POS	POS	POS	POS	POS	POS	NEG	POS	NEG	POS
Alexandria University_2020-27	CC1-MRSA-[V+fus+tir+ccrAB1] (PVL+)	POS	POS	POS	POS	POS	POS	POS	NEG	POS	NEG	POS
Alexandria University_2020-28	CC1-MRSA-[V+fus+tir+ccrAB1] (PVL+)	POS	AMB	POS	POS	POS	POS	AMB	NEG	POS	NEG	AMB
**CC5**												
Alexandria University_2020-12	CC5-MRSA-[VI+fus+tir]	POS	POS	POS	POS	POS	POS	POS	NEG	POS	POS	POS
Alexandria University_2020-20	CC5-MRSA-[VI+fus+tir]	POS	POS	POS	POS	POS	POS	AMB	NEG	POS	NEG	POS
**CC6**												
Alexandria University_2020-29	CC6-MRSA-[V+fus]	POS	POS	POS	POS	POS	POS	AMB	NEG	POS	NEG	POS
**CC15**												
Alexandria University_2020-01	CC15-MRSA-[V+fus]	POS	POS	POS	POS	NEG	NEG	NEG	NEG	NEG	POS	AMB
Alexandria University_2020-02	CC15-MRSA-[V+fus]	POS	POS	POS	POS	NEG	NEG	NEG	NEG	NEG	POS	POS
Alexandria University_2020-05	CC15-MRSA-[V+fus]	POS	AMB	POS	POS	NEG	NEG	NEG	NEG	NEG	POS	AMB
Alexandria University_2020-15	CC15-MRSA-[V+fus]	POS	POS	POS	POS	NEG	NEG	NEG	NEG	NEG	POS	POS
Alexandria University_2020-22	CC15-MRSA-[V+fus]	POS	NEG	POS	POS	NEG	NEG	NEG	NEG	NEG	POS	NEG
Alexandria University_2020-32	CC15-MRSA-[V+fus]	POS	POS	POS	POS	NEG	NEG	NEG	NEG	NEG	POS	POS
**CC22**												
Alexandria University_2020-04	CC22-MRSA-IVa (dcs negatives) (tst1+), "Gaza Epidemic Strain"	POS	NEG	NEG	NEG	POS	POS	POS	NEG	POS	POS	AMB
Alexandria University_2020-11	CC22-MRSA-IVa (dcs negatives) (tst1+), "Gaza Epidemic Strain"	POS	AMB	NEG	NEG	POS	POS	POS	NEG	POS	POS	POS
**CC30**												
Alexandria University_2020-26	CC30-MRSA-IVa (PVL+), "WSPP/Southwest Pacific Clone"	POS	POS	POS	POS	POS	POS	POS	NEG	POS	NEG	POS
**CC97**												
Alexandria University_2020-30	CC97-MRSA-[V+fus]	POS	POS	POS	POS	POS	POS	POS	NEG	POS	NEG	POS
**CC121**												
Alexandria University_2020-06	CC121-MRSA-[V+fus] (PVL+)	POS	POS	POS	NEG	POS	POS	POS	NEG	POS	NEG	POS
**CC152**												
Alexandria University_2020-18	CC152-MRSA-[V+fus]	NEG	POS	POS	NEG	NEG	NEG	POS	POS	POS	NEG	POS
Alexandria University_2020-31	CC152-MRSA-[V+fus]	NEG	POS	POS	NEG	NEG	NEG	POS	POS	POS	NEG	POS
**CC239**												
Alexandria University_2020-13	CC239-MRSA-[III+Cd+ccrC] (sasX-negat.), "Middle Eastern Cluster"	POS	NEG	POS	POS	POS	POS	POS	NEG	POS	NEG	POS
Alexandria University_2020-16	CC239-MRSA-[III+Cd], sasX-negative	POS	NEG	POS	POS	POS	POS	POS	NEG	POS	NEG	AMB
**CC1153**												
Alexandria University_2020-07	CC1153-MRSA-[V+fus] (PVL+)	AMB	AMB	POS	AMB	NEG	NEG	NEG	NEG	POS	NEG	AMB
Alexandria University_2020-08	CC1153-MRSA-[V+fus] (PVL+)	POS	AMB	POS	POS	NEG	NEG	NEG	NEG	POS	NEG	AMB
Alexandria University_2020-23	CC1153-MRSA-[V+fus] (PVL+)	POS	POS	POS	POS	NEG	NEG	NEG	NEG	POS	NEG	POS
**Simulated hybridisations for sequences from Montelongo et al., 2022**												
**CC1**												
JAEOUR Staphylococcus aureus strain AA1	CC1-MRSA-[V+fus+tir+ccrAB1]	POS	POS	POS	POS	POS	AMB	AMB	POS	POS	NEG	POS
JAEOWK Staphylococcus aureus strain AA78	CC1-MRSA-[V+fus+tir+ccrAB1]	POS	POS	POS	POS	POS	AMB	AMB	POS	POS	NEG	POS
JAEOVR Staphylococcus aureus strain AA51	CC1-MRSA-[V+fus+tir+ccrAB1] (PVL+)	POS	POS	POS	POS	POS	AMB	AMB	POS	POS	NEG	POS
JAEOWE Staphylococcus aureus strain AA67	CC1-MRSA-[V+fus+tir+ccrAB1] (PVL+)	POS	AMB	POS	POS	POS	AMB	AMB	POS	POS	NEG	POS
JAEOWG Staphylococcus aureus strain AA69	CC1-MRSA-[V+fus+tir+ccrAB1] (PVL+)	POS	POS	POS	POS	POS	AMB	AMB	POS	POS	NEG	POS
JAEOWJ Staphylococcus aureus strain AA77	CC1-MRSA-[V+fus+tir+ccrAB1] (PVL+)	POS	AMB	POS	POS	POS	AMB	AMB	POS	POS	NEG	POS
**CC5**												
JAEOWI Staphylococcus aureus strain AA76	CC5-MRSA-[V+cas], WA MRSA-123	POS	POS	POS	POS	AMB	POS	AMB	POS	NEG	NEG	NEG
JAEOWN Staphylococcus aureus strain AA80	CC5-MRSA-[V+cas], WA MRSA-123	POS	POS	POS	POS	AMB	POS	AMB	POS	POS	NEG	POS
JAEOWH Staphylococcus aureus strain AA70	CC5-MRSA-[VI+fus+tir+, dcs-]	POS	POS	POS	POS	AMB	POS	AMB	POS	POS	NEG	POS
**CC6**												
JAEOVF Staphylococcus aureus strain AA30	CC6-MRSA-IVa, WA MRSA-51	POS	POS	POS	POS	AMB	POS	AMB	POS	POS	NEG	POS
**CC80**												
JAEOVM Staphylococcus aureus strain AA4	CC80-MRSA-IVc (PVL-) [aphA3/sat+]:[far1+], contaminated	POS	POS	POS	POS	AMB	POS	AMB	POS	POS	NEG	POS
JAEOUZ Staphylococcus aureus strain AA2	CC80-MRSA-IVc (PVL+) [aphA3/sat+]:[far1+]	POS	POS	POS	POS	AMB	POS	AMB	POS	POS	NEG	POS
JAEOVE Staphylococcus aureus strain AA3	CC80-MRSA-IVc (PVL+) [aphA3/sat+]:[far1+]	POS	POS	POS	POS	AMB	POS	AMB	POS	POS	NEG	POS
JAEOVO Staphylococcus aureus strain AA45	CC80-MRSA-IVc (PVL+) [aphA3/sat+]:[far1+]	POS	POS	POS	POS	AMB	POS	AMB	POS	POS	NEG	POS
**CC88**												
JAEOVT Staphylococcus aureus strain AA53	CC88-MRSA-IV, contaminated	POS	POS	POS	POS	POS	AMB	AMB	POS	POS	POS	POS
**CC97**												
JAEOVL Staphylococcus aureus strain AA39	CC97-MRSA-IVc, WA MRSA-54/63	AMB	POS	POS	POS	POS	AMB	AMB	POS	POS	NEG	POS
JAEOVX Staphylococcus aureus strain AA6	CC97-MRSA-IVc, WA MRSA-54/63	AMB	POS	POS	POS	POS	AMB	AMB	POS	POS	NEG	POS
JAEOWM Staphylococcus aureus strain AA8	CC97-MRSA-IVc, WA MRSA-54/63	AMB	POS	POS	POS	POS	AMB	AMB	POS	POS	NEG	POS
JAEOUU Staphylococcus aureus strain AA104	CC97-MRSA-V	AMB	POS	POS	POS	POS	AMB	AMB	POS	POS	NEG	POS
JAEOVJ Staphylococcus aureus strain AA35	CC97-MRSA-[V+fus]	AMB	POS	POS	POS	POS	AMB	AMB	POS	POS	NEG	POS
JAEOVK Staphylococcus aureus strain AA36	CC97-MRSA-[V+fus]	AMB	POS	POS	POS	POS	AMB	AMB	POS	POS	NEG	POS
**CC22**												
JAEOUY Staphylococcus aureus strain AA18	CC22-MRSA-IVa (dcs negatives) (tst1+), "Gaza Epidemic Strain"	POS	AMB	NEG	NEG	POS	AMB	AMB	POS	POS	POS	POS
JAEOVH Staphylococcus aureus strain AA32	CC22-MRSA-IVa (dcs negatives) (tst1+), "Gaza Epidemic Strain"	POS	AMB	NEG	NEG	POS	AMB	AMB	POS	POS	POS	POS
JAEOVQ Staphylococcus aureus strain AA5	CC22-MRSA-IVa (dcs negatives) (tst1+), "Gaza Epidemic Strain"	POS	AMB	NEG	NEG	POS	AMB	AMB	POS	POS	POS	POS
**CC152**												
JAEOUX Staphylococcus aureus strain AA17	CC152-MRSA-[V+fus]	NEG	POS	POS	NEG	AMB	NEG	AMB	AMB	POS	NEG	POS
**CC239**												
JAEOVI Staphylococcus aureus strain AA33	CC239-MRSA-[III+Cd/Hg+ccrC] (sasX-positive), "Southeast Asian Clade"	POS	NEG	POS	POS	POS	AMB	AMB	POS	POS	NEG	POS
JAEOUS Staphylococcus aureus strain AA101	CC239-MRSA-[III+Cd+ccrC] (sasX-negat.), "Middle Eastern Cluster"	POS	NEG	POS	POS	POS	AMB	AMB	POS	POS	NEG	POS
JAEOUV Staphylococcus aureus strain AA13	CC239-MRSA-[III+Cd+ccrC] (sasX-negat.), "Middle Eastern Cluster"	POS	NEG	POS	POS	POS	AMB	AMB	POS	POS	NEG	POS
JAEOUW Staphylococcus aureus strain AA14	CC239-MRSA-[III+Cd+ccrC] (sasX-negat.), "Middle Eastern Cluster"	POS	NEG	POS	POS	POS	AMB	AMB	POS	POS	NEG	POS
JAEOVA Staphylococcus aureus strain AA22	CC239-MRSA-[III+Cd+ccrC] (sasX-negat.), "Middle Eastern Cluster"	POS	NEG	POS	POS	POS	AMB	AMB	POS	POS	NEG	POS
JAEOVB Staphylococcus aureus strain AA23	CC239-MRSA-[III+Cd+ccrC] (sasX-negat.), "Middle Eastern Cluster"	POS	NEG	POS	POS	POS	AMB	AMB	POS	POS	NEG	POS
JAEOVC Staphylococcus aureus strain AA27	CC239-MRSA-[III+Cd+ccrC] (sasX-negat.), "Middle Eastern Cluster"	POS	NEG	POS	POS	POS	AMB	AMB	POS	POS	NEG	POS
JAEOVD Staphylococcus aureus strain AA29	CC239-MRSA-[III+Cd+ccrC] (sasX-negat.), "Middle Eastern Cluster"	POS	NEG	POS	POS	NEG	NEG	NEG	POS	POS	NEG	POS
JAEOVG Staphylococcus aureus strain AA31	CC239-MRSA-[III+Cd+ccrC] (sasX-negat.), "Middle Eastern Cluster"	POS	NEG	POS	POS	POS	AMB	AMB	POS	POS	NEG	POS
JAEOVP Staphylococcus aureus strain AA46	CC239-MRSA-[III+Cd+ccrC] (sasX-negat.), "Middle Eastern Cluster"	POS	NEG	POS	POS	POS	AMB	AMB	POS	POS	NEG	POS
JAEOVS Staphylococcus aureus strain AA52	CC239-MRSA-[III+Cd+ccrC] (sasX-negat.), "Middle Eastern Cluster"	POS	NEG	POS	POS	POS	AMB	AMB	POS	POS	NEG	POS
JAEOVU Staphylococcus aureus strain AA55	CC239-MRSA-[III+Cd+ccrC] (sasX-negat.), "Middle Eastern Cluster"	POS	NEG	POS	POS	POS	AMB	AMB	POS	POS	NEG	POS
JAEOVV Staphylococcus aureus strain AA57	CC239-MRSA-[III+Cd+ccrC] (sasX-negat.), "Middle Eastern Cluster"	POS	NEG	POS	POS	POS	AMB	AMB	POS	POS	NEG	POS
JAEOVY Staphylococcus aureus strain AA60	CC239-MRSA-[III+Cd+ccrC] (sasX-negat.), "Middle Eastern Cluster"	POS	NEG	POS	POS	POS	AMB	AMB	POS	POS	NEG	POS
JAEOVZ Staphylococcus aureus strain AA61	CC239-MRSA-[III+Cd+ccrC] (sasX-negat.), "Middle Eastern Cluster"	POS	NEG	POS	POS	POS	AMB	AMB	POS	POS	NEG	POS
JAEOWA Staphylococcus aureus strain AA62	CC239-MRSA-[III+Cd+ccrC] (sasX-negat.), "Middle Eastern Cluster"	POS	NEG	POS	POS	POS	AMB	AMB	POS	POS	NEG	POS
JAEOWB Staphylococcus aureus strain AA63	CC239-MRSA-[III+Cd+ccrC] (sasX-negat.), "Middle Eastern Cluster"	POS	NEG	POS	POS	POS	AMB	AMB	POS	POS	NEG	POS
JAEOWL Staphylococcus aureus strain AA79	CC239-MRSA-[III+Cd+ccrC] (sasX-negat.), "Middle Eastern Cluster"	POS	NEG	POS	POS	POS	AMB	AMB	POS	POS	NEG	POS
JAEOWQ Staphylococcus aureus strain AA92	CC239-MRSA-[III+Cd+ccrC] (sasX-negat.), "Middle Eastern Cluster"	POS	NEG	POS	POS	POS	AMB	AMB	POS	POS	NEG	POS
JAEOWC Staphylococcus aureus strain AA64	CC239-MRSA-[mec III+Cd], sasX-negative	POS	NEG	POS	POS	POS	AMB	AMB	POS	POS	NEG	POS
JAEOWP Staphylococcus aureus strain AA91	CC239-MRSA-[mec III+Cd], sasX-negative	POS	NEG	POS	POS	POS	AMB	AMB	POS	POS	NEG	POS

Isolate	Strain assignment	VIRULENCE : OTHER FACTORS							VIRULENCE : PROTEASES					
									aur					
		etA	etB	etD	etD2	edinA	edinB	edinC	aur (cons)	aur (Other than MRSA252)	aur (MRSA252)	splA	splB	splE
		exfoliative toxin serotype A	exfoliative toxin serotype B	exfoliative toxin D	exfoliative toxin D2	epidermal cell differen-tiation inhibitor	epidermal cell differen-tiation inhibitor B	epidermal cell differentiation inhibitor C	aureolysin			serin- protease A	serin- protease B	serin- protease E
**CC1**														
Alexandria University_2020-17	CC1-MRSA-[V+fus+tir]	NEG	NEG	NEG	NEG	NEG	NEG	NEG	POS	POS	NEG	POS	POS	POS
Alexandria University_2020-03	CC1-MRSA-[V+fus+tir+ccrAB1]	NEG	NEG	NEG	NEG	NEG	NEG	NEG	POS	POS	NEG	POS	POS	POS
Alexandria University_2020-19	CC1-MRSA-[V+fus+tir+ccrAB1]	NEG	NEG	NEG	NEG	NEG	NEG	NEG	POS	POS	NEG	POS	POS	POS
Alexandria University_2020-21	CC1-MRSA-[V+fus+tir+ccrAB1]	NEG	NEG	NEG	NEG	NEG	NEG	NEG	NEG	NEG	NEG	POS	POS	POS
Alexandria University_2020-09	CC1-MRSA-[V+fus+tir+ccrAB1] (PVL+)	NEG	NEG	NEG	NEG	NEG	NEG	NEG	POS	POS	NEG	POS	POS	POS
Alexandria University_2020-14	CC1-MRSA-[V+fus+tir+ccrAB1] (PVL+)	NEG	NEG	NEG	NEG	NEG	NEG	NEG	POS	POS	NEG	POS	POS	POS
Alexandria University_2020-24	CC1-MRSA-[V+fus+tir+ccrAB1] (PVL+)	NEG	NEG	NEG	NEG	NEG	NEG	NEG	POS	POS	NEG	POS	POS	POS
Alexandria University_2020-27	CC1-MRSA-[V+fus+tir+ccrAB1] (PVL+)	NEG	NEG	NEG	NEG	NEG	NEG	NEG	POS	POS	NEG	POS	POS	POS
Alexandria University_2020-28	CC1-MRSA-[V+fus+tir+ccrAB1] (PVL+)	NEG	NEG	NEG	NEG	NEG	NEG	NEG	POS	POS	NEG	POS	POS	POS
**CC5**														
Alexandria University_2020-12	CC5-MRSA-[VI+fus+tir]	NEG	NEG	NEG	NEG	NEG	NEG	NEG	POS	POS	NEG	POS	POS	NEG
Alexandria University_2020-20	CC5-MRSA-[VI+fus+tir]	NEG	NEG	NEG	NEG	POS	NEG	NEG	POS	POS	NEG	POS	POS	NEG
**CC6**														
Alexandria University_2020-29	CC6-MRSA-[V+fus]	NEG	NEG	NEG	NEG	NEG	NEG	NEG	POS	POS	NEG	POS	POS	POS
**CC15**														
Alexandria University_2020-01	CC15-MRSA-[V+fus]	NEG	NEG	NEG	NEG	NEG	NEG	NEG	POS	POS	NEG	POS	POS	POS
Alexandria University_2020-02	CC15-MRSA-[V+fus]	NEG	NEG	NEG	NEG	NEG	NEG	NEG	POS	POS	NEG	POS	POS	POS
Alexandria University_2020-05	CC15-MRSA-[V+fus]	NEG	NEG	NEG	NEG	NEG	NEG	NEG	POS	POS	NEG	POS	POS	POS
Alexandria University_2020-15	CC15-MRSA-[V+fus]	NEG	NEG	NEG	NEG	NEG	NEG	NEG	POS	POS	NEG	POS	POS	POS
Alexandria University_2020-22	CC15-MRSA-[V+fus]	NEG	NEG	NEG	NEG	NEG	NEG	NEG	POS	POS	NEG	POS	POS	AMB
Alexandria University_2020-32	CC15-MRSA-[V+fus]	NEG	NEG	NEG	NEG	NEG	NEG	NEG	POS	POS	NEG	POS	POS	POS
**CC22**														
Alexandria University_2020-04	CC22-MRSA-IVa (dcs negatives) (tst1+), "Gaza Epidemic Strain"	NEG	NEG	NEG	NEG	NEG	NEG	NEG	POS	NEG	POS	NEG	NEG	NEG
Alexandria University_2020-11	CC22-MRSA-IVa (dcs negatives) (tst1+), "Gaza Epidemic Strain"	NEG	NEG	NEG	NEG	NEG	NEG	NEG	POS	NEG	POS	NEG	NEG	NEG
**CC30**														
Alexandria University_2020-26	CC30-MRSA-IVa (PVL+), "WSPP/Southwest Pacific Clone"	NEG	NEG	NEG	NEG	NEG	NEG	NEG	POS	NEG	POS	NEG	NEG	POS
**CC97**														
Alexandria University_2020-30	CC97-MRSA-[V+fus]	NEG	NEG	NEG	NEG	NEG	NEG	NEG	POS	POS	NEG	NEG	NEG	NEG
**CC121**														
Alexandria University_2020-06	CC121-MRSA-[V+fus] (PVL+)	NEG	NEG	NEG	NEG	NEG	NEG	NEG	POS	POS	NEG	NEG	POS	NEG
**CC152**														
Alexandria University_2020-18	CC152-MRSA-[V+fus]	NEG	NEG	NEG	POS	NEG	POS	NEG	POS	POS	NEG	NEG	NEG	NEG
Alexandria University_2020-31	CC152-MRSA-[V+fus]	NEG	NEG	NEG	AMB	NEG	POS	NEG	POS	POS	NEG	NEG	NEG	NEG
**CC239**														
Alexandria University_2020-13	CC239-MRSA-[III+Cd+ccrC] (sasX-negat.), "Middle Eastern Cluster"	NEG	NEG	NEG	NEG	NEG	NEG	NEG	POS	NEG	POS	POS	POS	POS
Alexandria University_2020-16	CC239-MRSA-[III+Cd], sasX-negative	NEG	NEG	NEG	NEG	NEG	NEG	NEG	POS	NEG	POS	POS	POS	POS
**CC1153**														
Alexandria University_2020-07	CC1153-MRSA-[V+fus] (PVL+)	NEG	NEG	NEG	NEG	NEG	NEG	NEG	POS	AMB	NEG	NEG	NEG	NEG
Alexandria University_2020-08	CC1153-MRSA-[V+fus] (PVL+)	NEG	NEG	NEG	NEG	NEG	NEG	NEG	POS	POS	NEG	NEG	NEG	NEG
Alexandria University_2020-23	CC1153-MRSA-[V+fus] (PVL+)	NEG	NEG	NEG	NEG	NEG	NEG	NEG	POS	POS	NEG	NEG	NEG	NEG
**Simulated hybridisations for sequences from Montelongo et al., 2022**														
**CC1**														
JAEOUR Staphylococcus aureus strain AA1	CC1-MRSA-[V+fus+tir+ccrAB1]	NEG	NEG	NEG	NEG	NEG	NEG	NEG	POS	POS	NEG	POS	POS	POS
JAEOWK Staphylococcus aureus strain AA78	CC1-MRSA-[V+fus+tir+ccrAB1]	NEG	NEG	NEG	NEG	NEG	NEG	NEG	POS	POS	NEG	POS	POS	POS
JAEOVR Staphylococcus aureus strain AA51	CC1-MRSA-[V+fus+tir+ccrAB1] (PVL+)	NEG	NEG	NEG	NEG	NEG	NEG	NEG	POS	POS	NEG	POS	POS	POS
JAEOWE Staphylococcus aureus strain AA67	CC1-MRSA-[V+fus+tir+ccrAB1] (PVL+)	NEG	NEG	NEG	NEG	NEG	NEG	NEG	POS	POS	NEG	POS	POS	POS
JAEOWG Staphylococcus aureus strain AA69	CC1-MRSA-[V+fus+tir+ccrAB1] (PVL+)	NEG	NEG	NEG	NEG	NEG	NEG	NEG	POS	POS	NEG	POS	POS	POS
JAEOWJ Staphylococcus aureus strain AA77	CC1-MRSA-[V+fus+tir+ccrAB1] (PVL+)	NEG	NEG	NEG	NEG	NEG	NEG	NEG	POS	POS	NEG	POS	POS	POS
**CC5**														
JAEOWI Staphylococcus aureus strain AA76	CC5-MRSA-[V+cas], WA MRSA-123	NEG	NEG	NEG	NEG	NEG	NEG	NEG	POS	POS	NEG	POS	POS	NEG
JAEOWN Staphylococcus aureus strain AA80	CC5-MRSA-[V+cas], WA MRSA-123	NEG	NEG	NEG	NEG	NEG	NEG	NEG	POS	POS	NEG	POS	POS	NEG
JAEOWH Staphylococcus aureus strain AA70	CC5-MRSA-[VI+fus+tir+, dcs-]	NEG	NEG	NEG	NEG	NEG	NEG	NEG	POS	POS	NEG	POS	POS	NEG
**CC6**														
JAEOVF Staphylococcus aureus strain AA30	CC6-MRSA-IVa, WA MRSA-51	NEG	NEG	NEG	NEG	NEG	NEG	NEG	POS	POS	NEG	POS	POS	POS
**CC80**														
JAEOVM Staphylococcus aureus strain AA4	CC80-MRSA-IVc (PVL-) [aphA3/sat+]:[far1+], contaminated	NEG	NEG	POS	NEG	NEG	POS	NEG	POS	POS	POS	POS	POS	NEG
JAEOUZ Staphylococcus aureus strain AA2	CC80-MRSA-IVc (PVL+) [aphA3/sat+]:[far1+]	NEG	NEG	POS	NEG	NEG	POS	NEG	POS	POS	NEG	POS	POS	NEG
JAEOVE Staphylococcus aureus strain AA3	CC80-MRSA-IVc (PVL+) [aphA3/sat+]:[far1+]	NEG	NEG	POS	NEG	NEG	POS	NEG	POS	POS	NEG	POS	POS	NEG
JAEOVO Staphylococcus aureus strain AA45	CC80-MRSA-IVc (PVL+) [aphA3/sat+]:[far1+]	NEG	NEG	POS	NEG	NEG	POS	NEG	POS	POS	NEG	POS	POS	NEG
**CC88**														
JAEOVT Staphylococcus aureus strain AA53	CC88-MRSA-IV, contaminated	POS	NEG	NEG	NEG	NEG	NEG	NEG	POS	POS	NEG	POS	POS	NEG
**CC97**														
JAEOVL Staphylococcus aureus strain AA39	CC97-MRSA-IVc, WA MRSA-54/63	NEG	NEG	NEG	NEG	NEG	NEG	NEG	POS	POS	NEG	POS	POS	POS
JAEOVX Staphylococcus aureus strain AA6	CC97-MRSA-IVc, WA MRSA-54/63	NEG	NEG	NEG	NEG	NEG	NEG	NEG	POS	POS	NEG	POS	POS	POS
JAEOWM Staphylococcus aureus strain AA8	CC97-MRSA-IVc, WA MRSA-54/63	NEG	NEG	NEG	NEG	NEG	NEG	NEG	POS	POS	NEG	POS	POS	POS
JAEOUU Staphylococcus aureus strain AA104	CC97-MRSA-V	NEG	NEG	NEG	NEG	NEG	NEG	NEG	POS	POS	NEG	POS	POS	POS
JAEOVJ Staphylococcus aureus strain AA35	CC97-MRSA-[V+fus]	NEG	NEG	NEG	NEG	NEG	NEG	NEG	POS	POS	NEG	NEG	NEG	NEG
JAEOVK Staphylococcus aureus strain AA36	CC97-MRSA-[V+fus]	NEG	NEG	NEG	NEG	NEG	NEG	NEG	POS	POS	NEG	POS	POS	POS
**CC22**														
JAEOUY Staphylococcus aureus strain AA18	CC22-MRSA-IVa (dcs negatives) (tst1+), "Gaza Epidemic Strain"	NEG	NEG	NEG	NEG	NEG	NEG	NEG	POS	NEG	POS	NEG	NEG	NEG
JAEOVH Staphylococcus aureus strain AA32	CC22-MRSA-IVa (dcs negatives) (tst1+), "Gaza Epidemic Strain"	NEG	NEG	NEG	NEG	NEG	NEG	NEG	POS	NEG	POS	NEG	NEG	NEG
JAEOVQ Staphylococcus aureus strain AA5	CC22-MRSA-IVa (dcs negatives) (tst1+), "Gaza Epidemic Strain"	NEG	NEG	NEG	NEG	NEG	NEG	NEG	POS	NEG	POS	NEG	NEG	NEG
**CC152**														
JAEOUX Staphylococcus aureus strain AA17	CC152-MRSA-[V+fus]	NEG	NEG	NEG	NEG	NEG	POS	NEG	AMB	POS	NEG	NEG	NEG	NEG
**CC239**														
JAEOVI Staphylococcus aureus strain AA33	CC239-MRSA-[III+Cd/Hg+ccrC] (sasX-positive), "Southeast Asian Clade"	NEG	NEG	NEG	NEG	NEG	NEG	NEG	POS	NEG	POS	POS	POS	POS
JAEOUS Staphylococcus aureus strain AA101	CC239-MRSA-[III+Cd+ccrC] (sasX-negat.), "Middle Eastern Cluster"	NEG	NEG	NEG	NEG	NEG	NEG	NEG	POS	NEG	POS	POS	POS	POS
JAEOUV Staphylococcus aureus strain AA13	CC239-MRSA-[III+Cd+ccrC] (sasX-negat.), "Middle Eastern Cluster"	NEG	NEG	NEG	NEG	NEG	NEG	NEG	POS	NEG	POS	POS	POS	POS
JAEOUW Staphylococcus aureus strain AA14	CC239-MRSA-[III+Cd+ccrC] (sasX-negat.), "Middle Eastern Cluster"	NEG	NEG	NEG	NEG	NEG	NEG	NEG	POS	NEG	POS	POS	POS	POS
JAEOVA Staphylococcus aureus strain AA22	CC239-MRSA-[III+Cd+ccrC] (sasX-negat.), "Middle Eastern Cluster"	NEG	NEG	NEG	NEG	NEG	NEG	NEG	POS	NEG	POS	POS	POS	POS
JAEOVB Staphylococcus aureus strain AA23	CC239-MRSA-[III+Cd+ccrC] (sasX-negat.), "Middle Eastern Cluster"	NEG	NEG	NEG	NEG	NEG	NEG	NEG	POS	NEG	POS	POS	POS	POS
JAEOVC Staphylococcus aureus strain AA27	CC239-MRSA-[III+Cd+ccrC] (sasX-negat.), "Middle Eastern Cluster"	NEG	NEG	NEG	NEG	NEG	NEG	NEG	POS	NEG	POS	POS	POS	POS
JAEOVD Staphylococcus aureus strain AA29	CC239-MRSA-[III+Cd+ccrC] (sasX-negat.), "Middle Eastern Cluster"	NEG	NEG	NEG	NEG	NEG	NEG	NEG	POS	NEG	POS	POS	POS	NEG
JAEOVG Staphylococcus aureus strain AA31	CC239-MRSA-[III+Cd+ccrC] (sasX-negat.), "Middle Eastern Cluster"	NEG	NEG	NEG	NEG	NEG	NEG	NEG	POS	NEG	POS	POS	POS	POS
JAEOVP Staphylococcus aureus strain AA46	CC239-MRSA-[III+Cd+ccrC] (sasX-negat.), "Middle Eastern Cluster"	NEG	NEG	NEG	NEG	NEG	NEG	NEG	POS	NEG	POS	POS	POS	POS
JAEOVS Staphylococcus aureus strain AA52	CC239-MRSA-[III+Cd+ccrC] (sasX-negat.), "Middle Eastern Cluster"	NEG	NEG	NEG	NEG	NEG	NEG	NEG	POS	NEG	POS	POS	POS	POS
JAEOVU Staphylococcus aureus strain AA55	CC239-MRSA-[III+Cd+ccrC] (sasX-negat.), "Middle Eastern Cluster"	NEG	NEG	NEG	NEG	NEG	NEG	NEG	POS	NEG	POS	POS	POS	POS
JAEOVV Staphylococcus aureus strain AA57	CC239-MRSA-[III+Cd+ccrC] (sasX-negat.), "Middle Eastern Cluster"	NEG	NEG	NEG	NEG	NEG	NEG	NEG	POS	NEG	POS	POS	POS	POS
JAEOVY Staphylococcus aureus strain AA60	CC239-MRSA-[III+Cd+ccrC] (sasX-negat.), "Middle Eastern Cluster"	NEG	NEG	NEG	NEG	NEG	NEG	NEG	POS	NEG	POS	POS	POS	POS
JAEOVZ Staphylococcus aureus strain AA61	CC239-MRSA-[III+Cd+ccrC] (sasX-negat.), "Middle Eastern Cluster"	NEG	NEG	NEG	NEG	NEG	NEG	NEG	POS	NEG	POS	POS	POS	POS
JAEOWA Staphylococcus aureus strain AA62	CC239-MRSA-[III+Cd+ccrC] (sasX-negat.), "Middle Eastern Cluster"	NEG	NEG	NEG	NEG	NEG	NEG	NEG	POS	NEG	POS	POS	POS	POS
JAEOWB Staphylococcus aureus strain AA63	CC239-MRSA-[III+Cd+ccrC] (sasX-negat.), "Middle Eastern Cluster"	NEG	NEG	NEG	NEG	NEG	NEG	NEG	POS	NEG	POS	POS	POS	POS
JAEOWL Staphylococcus aureus strain AA79	CC239-MRSA-[III+Cd+ccrC] (sasX-negat.), "Middle Eastern Cluster"	NEG	NEG	NEG	NEG	NEG	NEG	NEG	POS	NEG	POS	POS	POS	POS
JAEOWQ Staphylococcus aureus strain AA92	CC239-MRSA-[III+Cd+ccrC] (sasX-negat.), "Middle Eastern Cluster"	NEG	NEG	NEG	NEG	NEG	NEG	NEG	POS	NEG	POS	POS	POS	POS
JAEOWC Staphylococcus aureus strain AA64	CC239-MRSA-[mec III+Cd], sasX-negative	NEG	NEG	NEG	NEG	NEG	NEG	NEG	POS	NEG	POS	POS	POS	POS
JAEOWP Staphylococcus aureus strain AA91	CC239-MRSA-[mec III+Cd], sasX-negative	NEG	NEG	NEG	NEG	NEG	NEG	NEG	POS	NEG	POS	POS	POS	POS

Isolate	Strain assignment	VIRULENCE : PROTEASES				VIRULENCE : STAPHYLOCOCCAL SUPERANTIGEN/ENTEROTOXIN-LIKE GENES (SET/SSL)								
				sspP									ssl02	
		sspA	sspB	sspP (cons)	sspP (other than ST93)	setC / selX	ssl01/set6 (COL)	ssl01/set6 (Mu50+ N315)	ssl01/set6 (MW2+ MSSA476)	ssl01/set6 (MRSA252)	ssl01/set6 (RF122)	ssl01/set6 (other alleles)	ssl02/set7	ssl02/set7 (MRSA252)
		glutamylendopeptidase	Staphopain B, protease	Staphopain A (Staphylopain A), protease		Staphyl. exotoxin-like protein							Staphylococcal superantigen-like protein 2	
**CC1**														
Alexandria University_2020-17	CC1-MRSA-[V+fus+tir]	POS	POS	POS	POS	POS	NEG	NEG	POS	NEG	NEG	NEG	POS	NEG
Alexandria University_2020-03	CC1-MRSA-[V+fus+tir+ccrAB1]	POS	POS	POS	POS	POS	NEG	NEG	POS	NEG	NEG	NEG	POS	NEG
Alexandria University_2020-19	CC1-MRSA-[V+fus+tir+ccrAB1]	POS	POS	POS	POS	POS	NEG	NEG	POS	NEG	NEG	NEG	POS	NEG
Alexandria University_2020-21	CC1-MRSA-[V+fus+tir+ccrAB1]	POS	POS	POS	POS	POS	NEG	NEG	POS	NEG	NEG	NEG	POS	NEG
Alexandria University_2020-09	CC1-MRSA-[V+fus+tir+ccrAB1] (PVL+)	POS	POS	POS	POS	POS	NEG	NEG	POS	NEG	NEG	NEG	POS	NEG
Alexandria University_2020-14	CC1-MRSA-[V+fus+tir+ccrAB1] (PVL+)	POS	POS	POS	POS	POS	NEG	NEG	POS	NEG	NEG	NEG	POS	NEG
Alexandria University_2020-24	CC1-MRSA-[V+fus+tir+ccrAB1] (PVL+)	POS	POS	POS	POS	POS	NEG	NEG	POS	NEG	NEG	NEG	POS	NEG
Alexandria University_2020-27	CC1-MRSA-[V+fus+tir+ccrAB1] (PVL+)	POS	POS	POS	POS	POS	NEG	NEG	POS	NEG	NEG	NEG	POS	NEG
Alexandria University_2020-28	CC1-MRSA-[V+fus+tir+ccrAB1] (PVL+)	POS	POS	POS	POS	POS	NEG	NEG	POS	NEG	NEG	NEG	POS	NEG
**CC5**														
Alexandria University_2020-12	CC5-MRSA-[VI+fus+tir]	POS	POS	POS	POS	POS	NEG	POS	NEG	NEG	NEG	NEG	POS	AMB
Alexandria University_2020-20	CC5-MRSA-[VI+fus+tir]	POS	POS	POS	POS	POS	NEG	POS	NEG	NEG	NEG	NEG	POS	NEG
**CC6**														
Alexandria University_2020-29	CC6-MRSA-[V+fus]	POS	POS	POS	POS	POS	NEG	NEG	NEG	NEG	NEG	POS	POS	NEG
**CC15**														
Alexandria University_2020-01	CC15-MRSA-[V+fus]	POS	POS	POS	POS	POS	NEG	NEG	NEG	NEG	NEG	POS	POS	NEG
Alexandria University_2020-02	CC15-MRSA-[V+fus]	POS	POS	POS	POS	POS	NEG	NEG	NEG	NEG	NEG	POS	POS	NEG
Alexandria University_2020-05	CC15-MRSA-[V+fus]	POS	POS	POS	POS	POS	NEG	NEG	NEG	NEG	NEG	POS	POS	NEG
Alexandria University_2020-15	CC15-MRSA-[V+fus]	POS	POS	POS	POS	POS	NEG	NEG	NEG	NEG	NEG	POS	POS	NEG
Alexandria University_2020-22	CC15-MRSA-[V+fus]	POS	POS	POS	POS	POS	NEG	NEG	NEG	NEG	NEG	POS	POS	NEG
Alexandria University_2020-32	CC15-MRSA-[V+fus]	POS	POS	POS	POS	POS	NEG	NEG	NEG	NEG	NEG	POS	POS	NEG
**CC22**														
Alexandria University_2020-04	CC22-MRSA-IVa (dcs negatives) (tst1+), "Gaza Epidemic Strain"	POS	POS	POS	POS	POS	NEG	NEG	NEG	POS	NEG	NEG	AMB	POS
Alexandria University_2020-11	CC22-MRSA-IVa (dcs negatives) (tst1+), "Gaza Epidemic Strain"	POS	POS	POS	POS	POS	NEG	NEG	NEG	POS	NEG	NEG	AMB	POS
**CC30**														
Alexandria University_2020-26	CC30-MRSA-IVa (PVL+), "WSPP/Southwest Pacific Clone"	POS	POS	POS	POS	NEG	NEG	NEG	NEG	POS	NEG	NEG	NEG	POS
**CC97**														
Alexandria University_2020-30	CC97-MRSA-[V+fus]	POS	POS	POS	POS	POS	NEG	NEG	NEG	NEG	POS	NEG	POS	NEG
**CC121**														
Alexandria University_2020-06	CC121-MRSA-[V+fus] (PVL+)	POS	POS	POS	POS	POS	NEG	POS	NEG	NEG	NEG	NEG	POS	NEG
**CC152**														
Alexandria University_2020-18	CC152-MRSA-[V+fus]	POS	POS	POS	POS	NEG	NEG	NEG	NEG	NEG	POS	NEG	POS	AMB
Alexandria University_2020-31	CC152-MRSA-[V+fus]	POS	POS	POS	POS	NEG	NEG	NEG	NEG	NEG	POS	NEG	AMB	POS
**CC239**														
Alexandria University_2020-13	CC239-MRSA-[III+Cd+ccrC] (sasX-negat.), "Middle Eastern Cluster"	POS	POS	POS	POS	POS	POS	AMB	NEG	NEG	NEG	NEG	POS	NEG
Alexandria University_2020-16	CC239-MRSA-[III+Cd], sasX-negative	POS	POS	POS	POS	POS	POS	AMB	NEG	NEG	NEG	NEG	POS	NEG
**CC1153**														
Alexandria University_2020-07	CC1153-MRSA-[V+fus] (PVL+)	POS	POS	POS	POS	POS	NEG	NEG	NEG	NEG	NEG	POS	POS	NEG
Alexandria University_2020-08	CC1153-MRSA-[V+fus] (PVL+)	POS	POS	POS	POS	POS	NEG	NEG	NEG	NEG	NEG	POS	POS	NEG
Alexandria University_2020-23	CC1153-MRSA-[V+fus] (PVL+)	POS	POS	POS	POS	POS	NEG	NEG	NEG	NEG	NEG	POS	POS	NEG
**Simulated hybridisations for sequences from Montelongo et al., 2022**														
**CC1**														
JAEOUR Staphylococcus aureus strain AA1	CC1-MRSA-[V+fus+tir+ccrAB1]	POS	POS	POS	POS	POS	NEG	NEG	POS	NEG	NEG	NEG	POS	NEG
JAEOWK Staphylococcus aureus strain AA78	CC1-MRSA-[V+fus+tir+ccrAB1]	POS	POS	POS	POS	POS	NEG	NEG	POS	NEG	NEG	NEG	POS	NEG
JAEOVR Staphylococcus aureus strain AA51	CC1-MRSA-[V+fus+tir+ccrAB1] (PVL+)	POS	POS	POS	POS	POS	NEG	NEG	POS	NEG	NEG	NEG	POS	NEG
JAEOWE Staphylococcus aureus strain AA67	CC1-MRSA-[V+fus+tir+ccrAB1] (PVL+)	POS	POS	POS	POS	POS	NEG	NEG	POS	NEG	NEG	NEG	POS	NEG
JAEOWG Staphylococcus aureus strain AA69	CC1-MRSA-[V+fus+tir+ccrAB1] (PVL+)	POS	POS	POS	POS	POS	NEG	NEG	POS	NEG	NEG	NEG	POS	NEG
JAEOWJ Staphylococcus aureus strain AA77	CC1-MRSA-[V+fus+tir+ccrAB1] (PVL+)	POS	POS	POS	POS	POS	NEG	NEG	POS	NEG	NEG	NEG	POS	NEG
**CC5**														
JAEOWI Staphylococcus aureus strain AA76	CC5-MRSA-[V+cas], WA MRSA-123	POS	POS	POS	POS	POS	NEG	POS	NEG	NEG	NEG	NEG	POS	NEG
JAEOWN Staphylococcus aureus strain AA80	CC5-MRSA-[V+cas], WA MRSA-123	POS	POS	POS	POS	POS	NEG	POS	NEG	NEG	NEG	NEG	POS	NEG
JAEOWH Staphylococcus aureus strain AA70	CC5-MRSA-[VI+fus+tir+, dcs-]	POS	POS	POS	POS	POS	NEG	POS	NEG	NEG	NEG	NEG	POS	NEG
**CC6**														
JAEOVF Staphylococcus aureus strain AA30	CC6-MRSA-IVa, WA MRSA-51	POS	POS	POS	POS	POS	NEG	NEG	NEG	NEG	NEG	POS	POS	NEG
**CC80**														
JAEOVM Staphylococcus aureus strain AA4	CC80-MRSA-IVc (PVL-) [aphA3/sat+]:[far1+], contaminated	AMB	POS	POS	POS	AMB	NEG	NEG	NEG	NEG	NEG	POS	POS	NEG
JAEOUZ Staphylococcus aureus strain AA2	CC80-MRSA-IVc (PVL+) [aphA3/sat+]:[far1+]	AMB	POS	POS	POS	AMB	NEG	NEG	NEG	NEG	NEG	POS	POS	NEG
JAEOVE Staphylococcus aureus strain AA3	CC80-MRSA-IVc (PVL+) [aphA3/sat+]:[far1+]	AMB	POS	POS	POS	AMB	NEG	NEG	NEG	NEG	NEG	POS	POS	NEG
JAEOVO Staphylococcus aureus strain AA45	CC80-MRSA-IVc (PVL+) [aphA3/sat+]:[far1+]	AMB	POS	POS	POS	AMB	NEG	NEG	NEG	NEG	NEG	POS	POS	NEG
**CC88**														
JAEOVT Staphylococcus aureus strain AA53	CC88-MRSA-IV, contaminated	POS	POS	POS	POS	POS	NEG	NEG	NEG	NEG	NEG	POS	POS	NEG
**CC97**														
JAEOVL Staphylococcus aureus strain AA39	CC97-MRSA-IVc, WA MRSA-54/63	POS	POS	POS	POS	POS	NEG	NEG	NEG	NEG	POS	NEG	POS	NEG
JAEOVX Staphylococcus aureus strain AA6	CC97-MRSA-IVc, WA MRSA-54/63	POS	POS	POS	POS	POS	NEG	NEG	NEG	NEG	POS	NEG	POS	NEG
JAEOWM Staphylococcus aureus strain AA8	CC97-MRSA-IVc, WA MRSA-54/63	POS	POS	POS	POS	POS	NEG	NEG	NEG	NEG	POS	NEG	POS	NEG
JAEOUU Staphylococcus aureus strain AA104	CC97-MRSA-V	POS	POS	POS	POS	POS	NEG	NEG	NEG	NEG	POS	NEG	POS	NEG
JAEOVJ Staphylococcus aureus strain AA35	CC97-MRSA-[V+fus]	POS	POS	POS	POS	POS	NEG	NEG	NEG	NEG	POS	NEG	POS	NEG
JAEOVK Staphylococcus aureus strain AA36	CC97-MRSA-[V+fus]	POS	POS	POS	POS	POS	NEG	NEG	NEG	NEG	POS	NEG	POS	NEG
**CC22**														
JAEOUY Staphylococcus aureus strain AA18	CC22-MRSA-IVa (dcs negatives) (tst1+), "Gaza Epidemic Strain"	POS	POS	POS	POS	POS	NEG	NEG	NEG	NEG	NEG	POS	POS	NEG
JAEOVH Staphylococcus aureus strain AA32	CC22-MRSA-IVa (dcs negatives) (tst1+), "Gaza Epidemic Strain"	POS	POS	POS	POS	POS	NEG	NEG	NEG	NEG	NEG	POS	POS	NEG
JAEOVQ Staphylococcus aureus strain AA5	CC22-MRSA-IVa (dcs negatives) (tst1+), "Gaza Epidemic Strain"	POS	POS	POS	POS	POS	NEG	NEG	NEG	NEG	NEG	POS	POS	NEG
**CC152**														
JAEOUX Staphylococcus aureus strain AA17	CC152-MRSA-[V+fus]	NEG	POS	POS	POS	NEG	NEG	NEG	NEG	NEG	POS	NEG	POS	NEG
**CC239**														
JAEOVI Staphylococcus aureus strain AA33	CC239-MRSA-[III+Cd/Hg+ccrC] (sasX-positive), "Southeast Asian Clade"	POS	POS	POS	POS	POS	NEG	NEG	NEG	NEG	NEG	POS	POS	NEG
JAEOUS Staphylococcus aureus strain AA101	CC239-MRSA-[III+Cd+ccrC] (sasX-negat.), "Middle Eastern Cluster"	POS	POS	POS	POS	POS	NEG	NEG	NEG	NEG	NEG	POS	POS	NEG
JAEOUV Staphylococcus aureus strain AA13	CC239-MRSA-[III+Cd+ccrC] (sasX-negat.), "Middle Eastern Cluster"	POS	POS	POS	POS	POS	NEG	NEG	NEG	NEG	NEG	POS	POS	NEG
JAEOUW Staphylococcus aureus strain AA14	CC239-MRSA-[III+Cd+ccrC] (sasX-negat.), "Middle Eastern Cluster"	POS	POS	POS	POS	POS	NEG	NEG	NEG	NEG	NEG	POS	POS	NEG
JAEOVA Staphylococcus aureus strain AA22	CC239-MRSA-[III+Cd+ccrC] (sasX-negat.), "Middle Eastern Cluster"	POS	POS	POS	POS	POS	NEG	NEG	NEG	NEG	NEG	POS	POS	NEG
JAEOVB Staphylococcus aureus strain AA23	CC239-MRSA-[III+Cd+ccrC] (sasX-negat.), "Middle Eastern Cluster"	POS	POS	POS	POS	POS	NEG	NEG	NEG	NEG	NEG	POS	POS	NEG
JAEOVC Staphylococcus aureus strain AA27	CC239-MRSA-[III+Cd+ccrC] (sasX-negat.), "Middle Eastern Cluster"	POS	POS	POS	POS	POS	NEG	NEG	NEG	NEG	NEG	POS	POS	NEG
JAEOVD Staphylococcus aureus strain AA29	CC239-MRSA-[III+Cd+ccrC] (sasX-negat.), "Middle Eastern Cluster"	POS	POS	POS	POS	POS	NEG	NEG	NEG	NEG	NEG	POS	POS	NEG
JAEOVG Staphylococcus aureus strain AA31	CC239-MRSA-[III+Cd+ccrC] (sasX-negat.), "Middle Eastern Cluster"	POS	POS	POS	POS	POS	NEG	NEG	NEG	NEG	NEG	POS	POS	NEG
JAEOVP Staphylococcus aureus strain AA46	CC239-MRSA-[III+Cd+ccrC] (sasX-negat.), "Middle Eastern Cluster"	POS	POS	POS	POS	POS	NEG	NEG	NEG	NEG	NEG	POS	POS	NEG
JAEOVS Staphylococcus aureus strain AA52	CC239-MRSA-[III+Cd+ccrC] (sasX-negat.), "Middle Eastern Cluster"	POS	POS	POS	POS	POS	NEG	NEG	NEG	NEG	NEG	POS	POS	NEG
JAEOVU Staphylococcus aureus strain AA55	CC239-MRSA-[III+Cd+ccrC] (sasX-negat.), "Middle Eastern Cluster"	POS	POS	POS	POS	POS	NEG	NEG	NEG	NEG	NEG	POS	POS	NEG
JAEOVV Staphylococcus aureus strain AA57	CC239-MRSA-[III+Cd+ccrC] (sasX-negat.), "Middle Eastern Cluster"	POS	POS	POS	POS	POS	NEG	NEG	NEG	NEG	NEG	POS	POS	NEG
JAEOVY Staphylococcus aureus strain AA60	CC239-MRSA-[III+Cd+ccrC] (sasX-negat.), "Middle Eastern Cluster"	POS	POS	POS	POS	POS	NEG	NEG	NEG	NEG	NEG	POS	POS	NEG
JAEOVZ Staphylococcus aureus strain AA61	CC239-MRSA-[III+Cd+ccrC] (sasX-negat.), "Middle Eastern Cluster"	POS	POS	POS	POS	POS	NEG	NEG	NEG	NEG	NEG	POS	POS	NEG
JAEOWA Staphylococcus aureus strain AA62	CC239-MRSA-[III+Cd+ccrC] (sasX-negat.), "Middle Eastern Cluster"	POS	POS	POS	POS	POS	NEG	NEG	NEG	NEG	NEG	POS	POS	NEG
JAEOWB Staphylococcus aureus strain AA63	CC239-MRSA-[III+Cd+ccrC] (sasX-negat.), "Middle Eastern Cluster"	POS	POS	POS	POS	POS	NEG	NEG	NEG	NEG	NEG	POS	POS	NEG
JAEOWL Staphylococcus aureus strain AA79	CC239-MRSA-[III+Cd+ccrC] (sasX-negat.), "Middle Eastern Cluster"	POS	POS	POS	POS	POS	NEG	NEG	NEG	NEG	NEG	POS	POS	NEG
JAEOWQ Staphylococcus aureus strain AA92	CC239-MRSA-[III+Cd+ccrC] (sasX-negat.), "Middle Eastern Cluster"	POS	POS	POS	POS	POS	NEG	NEG	NEG	NEG	NEG	POS	POS	NEG
JAEOWC Staphylococcus aureus strain AA64	CC239-MRSA-[mec III+Cd], sasX-negative	POS	POS	POS	POS	POS	NEG	NEG	NEG	NEG	NEG	POS	POS	NEG
JAEOWP Staphylococcus aureus strain AA91	CC239-MRSA-[mec III+Cd], sasX-negative	POS	POS	POS	POS	POS	NEG	NEG	NEG	NEG	NEG	POS	POS	NEG

Isolate	Strain assignment	VIRULENCE : STAPHYLOCOCCAL SUPERANTIGEN/ENTEROTOXIN-LIKE GENES (SET/SSL)													
		ssl03			ssl04		ssl05				ssl06		ssl07		
		ssl03/set8_probe 1	ssl03/set8_probe 2	ssl03/set8 (MRSA252, SAR0424)	ssl04/set9	ssl04/set9 (MRSA252, SAR0425)	ssl05/set3_probe 1	ssl05/set3 (RF122, probe-611)	ssl05/set3_probe 2 (612)	ssl05/set3 (MRSA252)	ssl06/set21	ssl06 (NCTC8325+MW2)	ssl07/set1	ssl07/set1 (MRSA252)	ssl07/set1 (AF188836)
		Staphylococcal superantigen-like protein 3			Staphylococcal superantigen-like protein 4		Staphylococcal superantigen-like protein 5				Staphylococcal superantigen-like protein 6		Staphylococcal superantigen-like protein 7		
**CC1**															
Alexandria University_2020-17	CC1-MRSA-[V+fus+tir]	POS	POS	NEG	POS	NEG	POS	NEG	POS	NEG	POS	POS	POS	AMB	NEG
Alexandria University_2020-03	CC1-MRSA-[V+fus+tir+ccrAB1]	POS	POS	NEG	POS	NEG	POS	AMB	POS	NEG	POS	POS	POS	AMB	NEG
Alexandria University_2020-19	CC1-MRSA-[V+fus+tir+ccrAB1]	POS	POS	NEG	POS	NEG	POS	NEG	POS	NEG	POS	POS	POS	AMB	NEG
Alexandria University_2020-21	CC1-MRSA-[V+fus+tir+ccrAB1]	POS	POS	NEG	POS	NEG	POS	NEG	POS	NEG	POS	POS	POS	AMB	NEG
Alexandria University_2020-09	CC1-MRSA-[V+fus+tir+ccrAB1] (PVL+)	POS	POS	NEG	POS	NEG	POS	AMB	POS	NEG	POS	POS	POS	AMB	NEG
Alexandria University_2020-14	CC1-MRSA-[V+fus+tir+ccrAB1] (PVL+)	POS	POS	NEG	POS	NEG	POS	AMB	POS	NEG	POS	POS	POS	AMB	NEG
Alexandria University_2020-24	CC1-MRSA-[V+fus+tir+ccrAB1] (PVL+)	POS	POS	NEG	POS	NEG	POS	AMB	POS	NEG	POS	POS	POS	AMB	NEG
Alexandria University_2020-27	CC1-MRSA-[V+fus+tir+ccrAB1] (PVL+)	POS	POS	NEG	POS	NEG	POS	NEG	POS	NEG	POS	POS	POS	AMB	NEG
Alexandria University_2020-28	CC1-MRSA-[V+fus+tir+ccrAB1] (PVL+)	POS	POS	NEG	POS	NEG	POS	NEG	POS	NEG	POS	POS	POS	NEG	NEG
**CC5**															
Alexandria University_2020-12	CC5-MRSA-[VI+fus+tir]	POS	POS	NEG	POS	NEG	POS	AMB	POS	NEG	NEG	NEG	POS	AMB	NEG
Alexandria University_2020-20	CC5-MRSA-[VI+fus+tir]	POS	POS	NEG	POS	NEG	POS	NEG	POS	NEG	NEG	NEG	POS	AMB	NEG
**CC6**															
Alexandria University_2020-29	CC6-MRSA-[V+fus]	POS	POS	NEG	POS	NEG	POS	POS	AMB	NEG	NEG	NEG	POS	NEG	NEG
**CC15**															
Alexandria University_2020-01	CC15-MRSA-[V+fus]	POS	POS	NEG	POS	NEG	POS	POS	AMB	NEG	POS	POS	POS	AMB	NEG
Alexandria University_2020-02	CC15-MRSA-[V+fus]	POS	POS	NEG	POS	NEG	POS	POS	AMB	NEG	POS	POS	POS	AMB	NEG
Alexandria University_2020-05	CC15-MRSA-[V+fus]	POS	POS	NEG	POS	NEG	POS	POS	AMB	NEG	POS	POS	POS	NEG	NEG
Alexandria University_2020-15	CC15-MRSA-[V+fus]	POS	POS	NEG	POS	NEG	POS	POS	AMB	NEG	POS	POS	POS	AMB	NEG
Alexandria University_2020-22	CC15-MRSA-[V+fus]	POS	POS	NEG	POS	NEG	POS	POS	AMB	NEG	POS	POS	POS	NEG	NEG
Alexandria University_2020-32	CC15-MRSA-[V+fus]	POS	POS	NEG	POS	NEG	POS	POS	AMB	NEG	POS	POS	POS	AMB	NEG
**CC22**															
Alexandria University_2020-04	CC22-MRSA-IVa (dcs negatives) (tst1+), "Gaza Epidemic Strain"	NEG	NEG	NEG	NEG	POS	POS	NEG	NEG	POS	NEG	NEG	NEG	AMB	POS
Alexandria University_2020-11	CC22-MRSA-IVa (dcs negatives) (tst1+), "Gaza Epidemic Strain"	NEG	NEG	AMB	NEG	POS	POS	NEG	NEG	POS	NEG	NEG	NEG	AMB	POS
**CC30**															
Alexandria University_2020-26	CC30-MRSA-IVa (PVL+), "WSPP/Southwest Pacific Clone"	NEG	NEG	POS	NEG	POS	NEG	NEG	NEG	POS	NEG	NEG	NEG	POS	NEG
**CC97**															
Alexandria University_2020-30	CC97-MRSA-[V+fus]	POS	AMB	NEG	POS	NEG	POS	POS	AMB	NEG	NEG	NEG	POS	AMB	NEG
**CC121**															
Alexandria University_2020-06	CC121-MRSA-[V+fus] (PVL+)	POS	POS	NEG	POS	NEG	NEG	POS	AMB	NEG	POS	POS	POS	NEG	NEG
**CC152**															
Alexandria University_2020-18	CC152-MRSA-[V+fus]	NEG	NEG	NEG	NEG	NEG	NEG	NEG	NEG	NEG	NEG	NEG	NEG	NEG	NEG
Alexandria University_2020-31	CC152-MRSA-[V+fus]	NEG	NEG	NEG	NEG	NEG	NEG	NEG	NEG	NEG	NEG	NEG	NEG	NEG	NEG
**CC239**															
Alexandria University_2020-13	CC239-MRSA-[III+Cd+ccrC] (sasX-negat.), "Middle Eastern Cluster"	POS	POS	NEG	POS	NEG	POS	AMB	POS	NEG	POS	POS	POS	AMB	NEG
Alexandria University_2020-16	CC239-MRSA-[III+Cd], sasX-negative	POS	POS	NEG	POS	NEG	POS	NEG	POS	NEG	POS	POS	POS	NEG	NEG
**CC1153**															
Alexandria University_2020-07	CC1153-MRSA-[V+fus] (PVL+)	POS	POS	NEG	POS	NEG	POS	POS	AMB	NEG	NEG	NEG	POS	AMB	NEG
Alexandria University_2020-08	CC1153-MRSA-[V+fus] (PVL+)	POS	POS	NEG	POS	NEG	POS	POS	AMB	NEG	NEG	NEG	POS	AMB	NEG
Alexandria University_2020-23	CC1153-MRSA-[V+fus] (PVL+)	POS	POS	NEG	POS	NEG	POS	POS	AMB	NEG	NEG	NEG	POS	AMB	NEG
**Simulated hybridisations for sequences from Montelongo et al., 2022**															
**CC1**															
JAEOUR Staphylococcus aureus strain AA1	CC1-MRSA-[V+fus+tir+ccrAB1]	POS	AMB	NEG	POS	NEG	POS	NEG	POS	NEG	POS	POS	POS	NEG	NEG
JAEOWK Staphylococcus aureus strain AA78	CC1-MRSA-[V+fus+tir+ccrAB1]	POS	AMB	NEG	POS	NEG	POS	NEG	POS	NEG	POS	POS	POS	NEG	NEG
JAEOVR Staphylococcus aureus strain AA51	CC1-MRSA-[V+fus+tir+ccrAB1] (PVL+)	POS	AMB	NEG	POS	NEG	POS	NEG	POS	NEG	POS	POS	POS	NEG	NEG
JAEOWE Staphylococcus aureus strain AA67	CC1-MRSA-[V+fus+tir+ccrAB1] (PVL+)	POS	AMB	NEG	POS	NEG	POS	NEG	POS	NEG	POS	POS	POS	NEG	NEG
JAEOWG Staphylococcus aureus strain AA69	CC1-MRSA-[V+fus+tir+ccrAB1] (PVL+)	POS	AMB	NEG	POS	NEG	POS	NEG	POS	NEG	POS	POS	POS	NEG	NEG
JAEOWJ Staphylococcus aureus strain AA77	CC1-MRSA-[V+fus+tir+ccrAB1] (PVL+)	POS	AMB	NEG	POS	NEG	POS	NEG	POS	NEG	POS	POS	POS	NEG	NEG
**CC5**															
JAEOWI Staphylococcus aureus strain AA76	CC5-MRSA-[V+cas], WA MRSA-123	POS	AMB	NEG	POS	NEG	POS	NEG	POS	NEG	NEG	NEG	POS	NEG	NEG
JAEOWN Staphylococcus aureus strain AA80	CC5-MRSA-[V+cas], WA MRSA-123	POS	AMB	NEG	POS	NEG	POS	NEG	POS	NEG	NEG	NEG	POS	NEG	NEG
JAEOWH Staphylococcus aureus strain AA70	CC5-MRSA-[VI+fus+tir+, dcs-]	POS	AMB	NEG	POS	NEG	POS	NEG	POS	NEG	NEG	NEG	POS	NEG	NEG
**CC6**															
JAEOVF Staphylococcus aureus strain AA30	CC6-MRSA-IVa, WA MRSA-51	AMB	POS	NEG	POS	NEG	POS	POS	NEG	NEG	NEG	NEG	POS	NEG	NEG
**CC80**															
JAEOVM Staphylococcus aureus strain AA4	CC80-MRSA-IVc (PVL-) [aphA3/sat+]:[far1+], contaminated	AMB	POS	NEG	POS	NEG	POS	POS	NEG	NEG	NEG	NEG	POS	NEG	NEG
JAEOUZ Staphylococcus aureus strain AA2	CC80-MRSA-IVc (PVL+) [aphA3/sat+]:[far1+]	AMB	POS	NEG	POS	NEG	POS	POS	NEG	NEG	NEG	NEG	POS	NEG	NEG
JAEOVE Staphylococcus aureus strain AA3	CC80-MRSA-IVc (PVL+) [aphA3/sat+]:[far1+]	AMB	POS	NEG	POS	NEG	POS	POS	NEG	NEG	NEG	NEG	POS	NEG	NEG
JAEOVO Staphylococcus aureus strain AA45	CC80-MRSA-IVc (PVL+) [aphA3/sat+]:[far1+]	AMB	POS	NEG	POS	NEG	POS	POS	NEG	NEG	NEG	NEG	POS	NEG	NEG
**CC88**															
JAEOVT Staphylococcus aureus strain AA53	CC88-MRSA-IV, contaminated	AMB	POS	NEG	POS	NEG	NEG	NEG	POS	NEG	POS	AMB	POS	NEG	NEG
**CC97**															
JAEOVL Staphylococcus aureus strain AA39	CC97-MRSA-IVc, WA MRSA-54/63	POS	AMB	NEG	POS	NEG	POS	POS	NEG	NEG	NEG	NEG	POS	NEG	NEG
JAEOVX Staphylococcus aureus strain AA6	CC97-MRSA-IVc, WA MRSA-54/63	POS	AMB	NEG	POS	NEG	POS	POS	NEG	NEG	NEG	NEG	POS	NEG	NEG
JAEOWM Staphylococcus aureus strain AA8	CC97-MRSA-IVc, WA MRSA-54/63	POS	AMB	NEG	POS	NEG	POS	POS	NEG	NEG	NEG	NEG	POS	NEG	NEG
JAEOUU Staphylococcus aureus strain AA104	CC97-MRSA-V	POS	AMB	NEG	POS	NEG	POS	POS	NEG	NEG	NEG	NEG	POS	NEG	NEG
JAEOVJ Staphylococcus aureus strain AA35	CC97-MRSA-[V+fus]	POS	AMB	NEG	POS	NEG	POS	POS	NEG	NEG	NEG	NEG	POS	NEG	NEG
JAEOVK Staphylococcus aureus strain AA36	CC97-MRSA-[V+fus]	POS	AMB	NEG	POS	NEG	POS	POS	NEG	NEG	NEG	NEG	POS	NEG	NEG
**CC22**															
JAEOUY Staphylococcus aureus strain AA18	CC22-MRSA-IVa (dcs negatives) (tst1+), "Gaza Epidemic Strain"	NEG	NEG	AMB	NEG	AMB	AMB	NEG	NEG	POS	NEG	NEG	NEG	NEG	POS
JAEOVH Staphylococcus aureus strain AA32	CC22-MRSA-IVa (dcs negatives) (tst1+), "Gaza Epidemic Strain"	NEG	NEG	AMB	NEG	AMB	AMB	NEG	NEG	POS	NEG	NEG	NEG	NEG	POS
JAEOVQ Staphylococcus aureus strain AA5	CC22-MRSA-IVa (dcs negatives) (tst1+), "Gaza Epidemic Strain"	NEG	NEG	AMB	NEG	AMB	AMB	NEG	NEG	POS	NEG	NEG	NEG	NEG	POS
**CC152**															
JAEOUX Staphylococcus aureus strain AA17	CC152-MRSA-[V+fus]	NEG	NEG	NEG	NEG	NEG	NEG	NEG	NEG	NEG	NEG	NEG	NEG	NEG	POS
**CC239**															
JAEOVI Staphylococcus aureus strain AA33	CC239-MRSA-[III+Cd/Hg+ccrC] (sasX-positive), "Southeast Asian Clade"	POS	AMB	NEG	POS	NEG	POS	NEG	POS	NEG	POS	POS	POS	NEG	NEG
JAEOUS Staphylococcus aureus strain AA101	CC239-MRSA-[III+Cd+ccrC] (sasX-negat.), "Middle Eastern Cluster"	POS	AMB	NEG	POS	NEG	POS	NEG	POS	NEG	POS	POS	POS	NEG	NEG
JAEOUV Staphylococcus aureus strain AA13	CC239-MRSA-[III+Cd+ccrC] (sasX-negat.), "Middle Eastern Cluster"	POS	AMB	NEG	POS	NEG	POS	NEG	POS	NEG	POS	POS	POS	NEG	NEG
JAEOUW Staphylococcus aureus strain AA14	CC239-MRSA-[III+Cd+ccrC] (sasX-negat.), "Middle Eastern Cluster"	POS	AMB	NEG	POS	NEG	POS	NEG	POS	NEG	POS	POS	POS	NEG	NEG
JAEOVA Staphylococcus aureus strain AA22	CC239-MRSA-[III+Cd+ccrC] (sasX-negat.), "Middle Eastern Cluster"	POS	AMB	NEG	POS	NEG	POS	NEG	POS	NEG	POS	POS	POS	NEG	NEG
JAEOVB Staphylococcus aureus strain AA23	CC239-MRSA-[III+Cd+ccrC] (sasX-negat.), "Middle Eastern Cluster"	POS	AMB	NEG	POS	NEG	POS	NEG	POS	NEG	POS	POS	POS	NEG	NEG
JAEOVC Staphylococcus aureus strain AA27	CC239-MRSA-[III+Cd+ccrC] (sasX-negat.), "Middle Eastern Cluster"	POS	AMB	NEG	POS	NEG	POS	NEG	POS	NEG	POS	POS	POS	NEG	NEG
JAEOVD Staphylococcus aureus strain AA29	CC239-MRSA-[III+Cd+ccrC] (sasX-negat.), "Middle Eastern Cluster"	POS	AMB	NEG	POS	NEG	POS	NEG	POS	NEG	POS	POS	POS	NEG	NEG
JAEOVG Staphylococcus aureus strain AA31	CC239-MRSA-[III+Cd+ccrC] (sasX-negat.), "Middle Eastern Cluster"	POS	AMB	NEG	POS	NEG	POS	NEG	POS	NEG	POS	POS	POS	NEG	NEG
JAEOVP Staphylococcus aureus strain AA46	CC239-MRSA-[III+Cd+ccrC] (sasX-negat.), "Middle Eastern Cluster"	POS	AMB	NEG	POS	NEG	POS	NEG	POS	NEG	POS	POS	POS	NEG	NEG
JAEOVS Staphylococcus aureus strain AA52	CC239-MRSA-[III+Cd+ccrC] (sasX-negat.), "Middle Eastern Cluster"	POS	AMB	NEG	POS	NEG	POS	NEG	POS	NEG	POS	POS	POS	NEG	NEG
JAEOVU Staphylococcus aureus strain AA55	CC239-MRSA-[III+Cd+ccrC] (sasX-negat.), "Middle Eastern Cluster"	POS	AMB	NEG	POS	NEG	POS	NEG	POS	NEG	POS	POS	POS	NEG	NEG
JAEOVV Staphylococcus aureus strain AA57	CC239-MRSA-[III+Cd+ccrC] (sasX-negat.), "Middle Eastern Cluster"	POS	AMB	NEG	POS	NEG	POS	NEG	POS	NEG	POS	POS	POS	NEG	NEG
JAEOVY Staphylococcus aureus strain AA60	CC239-MRSA-[III+Cd+ccrC] (sasX-negat.), "Middle Eastern Cluster"	POS	AMB	NEG	POS	NEG	POS	NEG	POS	NEG	POS	POS	POS	NEG	NEG
JAEOVZ Staphylococcus aureus strain AA61	CC239-MRSA-[III+Cd+ccrC] (sasX-negat.), "Middle Eastern Cluster"	POS	AMB	NEG	POS	NEG	POS	NEG	POS	NEG	POS	POS	POS	NEG	NEG
JAEOWA Staphylococcus aureus strain AA62	CC239-MRSA-[III+Cd+ccrC] (sasX-negat.), "Middle Eastern Cluster"	POS	AMB	NEG	POS	NEG	POS	NEG	POS	NEG	POS	POS	POS	NEG	NEG
JAEOWB Staphylococcus aureus strain AA63	CC239-MRSA-[III+Cd+ccrC] (sasX-negat.), "Middle Eastern Cluster"	POS	AMB	NEG	POS	NEG	POS	NEG	POS	NEG	POS	POS	POS	NEG	NEG
JAEOWL Staphylococcus aureus strain AA79	CC239-MRSA-[III+Cd+ccrC] (sasX-negat.), "Middle Eastern Cluster"	POS	AMB	NEG	POS	NEG	POS	NEG	POS	NEG	POS	POS	POS	NEG	NEG
JAEOWQ Staphylococcus aureus strain AA92	CC239-MRSA-[III+Cd+ccrC] (sasX-negat.), "Middle Eastern Cluster"	POS	AMB	NEG	POS	NEG	POS	NEG	POS	NEG	POS	POS	POS	NEG	NEG
JAEOWC Staphylococcus aureus strain AA64	CC239-MRSA-[mec III+Cd], sasX-negative	POS	AMB	NEG	POS	NEG	POS	NEG	POS	NEG	POS	POS	POS	NEG	NEG
JAEOWP Staphylococcus aureus strain AA91	CC239-MRSA-[mec III+Cd], sasX-negative	POS	AMB	NEG	POS	NEG	POS	NEG	POS	NEG	POS	POS	POS	NEG	NEG

Isolate	Strain assignment	VIRULENCE : STAPHYLOCOCCAL SUPERANTIGEN/ENTEROTOXIN-LIKE GENES (SET/SSL)											
		ssl08		ssl09			ssl10			ssl11			
		ssl08/set12_probe 1	ssl08/set12_probe 2	ssl09/set5_probe 1	ssl09/set5_probe 2	ssl09/set5 (MRSA252)	ssl10/set4	ssl10 (RF122)	ssl10/set4 (MRSA252)	ssl11/set2 (COL)	ssl11/set2 (Mu50+N315)	ssl11/set2 (MW2+MSSA476)	ssl11/set2 (MRSA252)
		Staphylococcal superantigen-like protein 8		Staphylococcal superantigen-like protein 9			Staphylococcal superantigen-like protein 10			Staphylococcal superantigene-like protein 11			
**CC1**													
Alexandria University_2020-17	CC1-MRSA-[V+fus+tir]	POS	POS	POS	POS	NEG	POS	NEG	AMB	NEG	NEG	POS	NEG
Alexandria University_2020-03	CC1-MRSA-[V+fus+tir+ccrAB1]	POS	POS	POS	POS	NEG	POS	NEG	AMB	NEG	NEG	POS	NEG
Alexandria University_2020-19	CC1-MRSA-[V+fus+tir+ccrAB1]	POS	POS	POS	POS	NEG	POS	NEG	AMB	NEG	NEG	POS	NEG
Alexandria University_2020-21	CC1-MRSA-[V+fus+tir+ccrAB1]	POS	POS	POS	POS	NEG	POS	NEG	AMB	NEG	NEG	POS	NEG
Alexandria University_2020-09	CC1-MRSA-[V+fus+tir+ccrAB1] (PVL+)	POS	POS	POS	POS	NEG	POS	NEG	AMB	NEG	NEG	POS	NEG
Alexandria University_2020-14	CC1-MRSA-[V+fus+tir+ccrAB1] (PVL+)	POS	POS	POS	POS	NEG	POS	NEG	AMB	NEG	NEG	POS	NEG
Alexandria University_2020-24	CC1-MRSA-[V+fus+tir+ccrAB1] (PVL+)	POS	POS	POS	POS	NEG	POS	NEG	AMB	NEG	NEG	POS	NEG
Alexandria University_2020-27	CC1-MRSA-[V+fus+tir+ccrAB1] (PVL+)	POS	POS	POS	POS	NEG	POS	NEG	AMB	NEG	NEG	POS	NEG
Alexandria University_2020-28	CC1-MRSA-[V+fus+tir+ccrAB1] (PVL+)	POS	POS	POS	POS	NEG	POS	NEG	NEG	NEG	NEG	POS	NEG
**CC5**													
Alexandria University_2020-12	CC5-MRSA-[VI+fus+tir]	POS	POS	POS	POS	NEG	POS	NEG	AMB	NEG	POS	NEG	NEG
Alexandria University_2020-20	CC5-MRSA-[VI+fus+tir]	POS	POS	POS	POS	NEG	POS	NEG	AMB	NEG	POS	NEG	NEG
**CC6**													
Alexandria University_2020-29	CC6-MRSA-[V+fus]	POS	POS	POS	POS	NEG	AMB	POS	NEG	NEG	POS	NEG	NEG
**CC15**													
Alexandria University_2020-01	CC15-MRSA-[V+fus]	NEG	POS	POS	POS	NEG	AMB	POS	NEG	NEG	NEG	NEG	POS
Alexandria University_2020-02	CC15-MRSA-[V+fus]	NEG	POS	POS	POS	NEG	AMB	POS	NEG	NEG	NEG	NEG	POS
Alexandria University_2020-05	CC15-MRSA-[V+fus]	NEG	POS	POS	POS	NEG	AMB	POS	NEG	NEG	NEG	NEG	POS
Alexandria University_2020-15	CC15-MRSA-[V+fus]	NEG	POS	POS	POS	NEG	AMB	POS	NEG	NEG	NEG	NEG	POS
Alexandria University_2020-22	CC15-MRSA-[V+fus]	NEG	POS	POS	POS	NEG	AMB	POS	NEG	NEG	NEG	NEG	POS
Alexandria University_2020-32	CC15-MRSA-[V+fus]	NEG	POS	POS	POS	NEG	AMB	POS	NEG	NEG	NEG	NEG	POS
**CC22**													
Alexandria University_2020-04	CC22-MRSA-IVa (dcs negatives) (tst1+), "Gaza Epidemic Strain"	NEG	NEG	POS	POS	NEG	NEG	NEG	NEG	NEG	NEG	NEG	NEG
Alexandria University_2020-11	CC22-MRSA-IVa (dcs negatives) (tst1+), "Gaza Epidemic Strain"	NEG	NEG	POS	POS	NEG	NEG	NEG	NEG	NEG	NEG	NEG	NEG
**CC30**													
Alexandria University_2020-26	CC30-MRSA-IVa (PVL+), "WSPP/Southwest Pacific Clone"	NEG	NEG	NEG	NEG	POS	AMB	NEG	POS	NEG	NEG	NEG	POS
**CC97**													
Alexandria University_2020-30	CC97-MRSA-[V+fus]	POS	POS	POS	POS	NEG	POS	NEG	AMB	NEG	NEG	NEG	NEG
**CC121**													
Alexandria University_2020-06	CC121-MRSA-[V+fus] (PVL+)	POS	POS	POS	POS	NEG	AMB	POS	NEG	NEG	NEG	NEG	NEG
**CC152**													
Alexandria University_2020-18	CC152-MRSA-[V+fus]	NEG	NEG	NEG	NEG	NEG	POS	NEG	NEG	NEG	NEG	NEG	NEG
Alexandria University_2020-31	CC152-MRSA-[V+fus]	NEG	NEG	NEG	NEG	NEG	POS	NEG	NEG	NEG	NEG	NEG	NEG
**CC239**													
Alexandria University_2020-13	CC239-MRSA-[III+Cd+ccrC] (sasX-negat.), "Middle Eastern Cluster"	POS	POS	POS	POS	NEG	POS	NEG	AMB	POS	NEG	NEG	NEG
Alexandria University_2020-16	CC239-MRSA-[III+Cd], sasX-negative	POS	POS	POS	POS	NEG	POS	NEG	NEG	POS	NEG	NEG	NEG
**CC1153**													
Alexandria University_2020-07	CC1153-MRSA-[V+fus] (PVL+)	POS	POS	AMB	POS	NEG	POS	NEG	NEG	NEG	NEG	NEG	NEG
Alexandria University_2020-08	CC1153-MRSA-[V+fus] (PVL+)	POS	POS	POS	POS	NEG	POS	NEG	AMB	NEG	NEG	NEG	NEG
Alexandria University_2020-23	CC1153-MRSA-[V+fus] (PVL+)	POS	POS	POS	POS	NEG	POS	NEG	AMB	NEG	NEG	NEG	NEG
**Simulated hybridisations for sequences from Montelongo et al., 2022**													
**CC1**													
JAEOUR Staphylococcus aureus strain AA1	CC1-MRSA-[V+fus+tir+ccrAB1]	POS	AMB	POS	POS	NEG	POS	NEG	NEG	NEG	NEG	POS	NEG
JAEOWK Staphylococcus aureus strain AA78	CC1-MRSA-[V+fus+tir+ccrAB1]	POS	AMB	POS	POS	NEG	POS	NEG	NEG	NEG	NEG	POS	NEG
JAEOVR Staphylococcus aureus strain AA51	CC1-MRSA-[V+fus+tir+ccrAB1] (PVL+)	POS	AMB	POS	POS	NEG	POS	NEG	NEG	NEG	NEG	POS	NEG
JAEOWE Staphylococcus aureus strain AA67	CC1-MRSA-[V+fus+tir+ccrAB1] (PVL+)	POS	AMB	POS	POS	NEG	POS	NEG	NEG	NEG	NEG	POS	NEG
JAEOWG Staphylococcus aureus strain AA69	CC1-MRSA-[V+fus+tir+ccrAB1] (PVL+)	POS	AMB	POS	POS	NEG	POS	NEG	NEG	NEG	NEG	POS	NEG
JAEOWJ Staphylococcus aureus strain AA77	CC1-MRSA-[V+fus+tir+ccrAB1] (PVL+)	POS	AMB	POS	POS	NEG	POS	NEG	NEG	NEG	NEG	POS	NEG
**CC5**													
JAEOWI Staphylococcus aureus strain AA76	CC5-MRSA-[V+cas], WA MRSA-123	POS	AMB	POS	POS	NEG	POS	NEG	NEG	NEG	POS	NEG	NEG
JAEOWN Staphylococcus aureus strain AA80	CC5-MRSA-[V+cas], WA MRSA-123	POS	AMB	POS	POS	NEG	POS	NEG	NEG	NEG	POS	NEG	NEG
JAEOWH Staphylococcus aureus strain AA70	CC5-MRSA-[VI+fus+tir+, dcs-]	POS	AMB	POS	POS	NEG	POS	NEG	NEG	NEG	POS	NEG	NEG
**CC6**													
JAEOVF Staphylococcus aureus strain AA30	CC6-MRSA-IVa, WA MRSA-51	POS	AMB	POS	POS	NEG	NEG	POS	NEG	NEG	POS	NEG	NEG
**CC80**													
JAEOVM Staphylococcus aureus strain AA4	CC80-MRSA-IVc (PVL-) [aphA3/sat+]:[far1+], contaminated	POS	AMB	POS	POS	NEG	POS	NEG	NEG	POS	NEG	NEG	NEG
JAEOUZ Staphylococcus aureus strain AA2	CC80-MRSA-IVc (PVL+) [aphA3/sat+]:[far1+]	POS	AMB	POS	POS	NEG	POS	NEG	NEG	NEG	NEG	NEG	NEG
JAEOVE Staphylococcus aureus strain AA3	CC80-MRSA-IVc (PVL+) [aphA3/sat+]:[far1+]	POS	AMB	POS	POS	NEG	POS	NEG	NEG	NEG	NEG	NEG	NEG
JAEOVO Staphylococcus aureus strain AA45	CC80-MRSA-IVc (PVL+) [aphA3/sat+]:[far1+]	POS	AMB	POS	POS	NEG	POS	NEG	NEG	NEG	NEG	NEG	NEG
**CC88**													
JAEOVT Staphylococcus aureus strain AA53	CC88-MRSA-IV, contaminated	POS	AMB	POS	POS	NEG	POS	NEG	NEG	NEG	NEG	NEG	AMB
**CC97**													
JAEOVL Staphylococcus aureus strain AA39	CC97-MRSA-IVc, WA MRSA-54/63	POS	AMB	POS	POS	NEG	POS	NEG	NEG	NEG	NEG	NEG	NEG
JAEOVX Staphylococcus aureus strain AA6	CC97-MRSA-IVc, WA MRSA-54/63	POS	AMB	POS	POS	NEG	POS	NEG	NEG	NEG	NEG	NEG	NEG
JAEOWM Staphylococcus aureus strain AA8	CC97-MRSA-IVc, WA MRSA-54/63	POS	AMB	POS	POS	NEG	POS	NEG	NEG	NEG	NEG	NEG	NEG
JAEOUU Staphylococcus aureus strain AA104	CC97-MRSA-V	POS	AMB	POS	POS	NEG	POS	NEG	NEG	NEG	NEG	NEG	NEG
JAEOVJ Staphylococcus aureus strain AA35	CC97-MRSA-[V+fus]	POS	AMB	POS	POS	NEG	POS	NEG	NEG	NEG	NEG	NEG	NEG
JAEOVK Staphylococcus aureus strain AA36	CC97-MRSA-[V+fus]	POS	AMB	POS	POS	NEG	POS	NEG	NEG	NEG	NEG	NEG	NEG
**CC22**													
JAEOUY Staphylococcus aureus strain AA18	CC22-MRSA-IVa (dcs negatives) (tst1+), "Gaza Epidemic Strain"	NEG	NEG	POS	POS	NEG	NEG	NEG	POS	NEG	NEG	NEG	NEG
JAEOVH Staphylococcus aureus strain AA32	CC22-MRSA-IVa (dcs negatives) (tst1+), "Gaza Epidemic Strain"	NEG	NEG	POS	POS	NEG	NEG	NEG	POS	NEG	NEG	NEG	NEG
JAEOVQ Staphylococcus aureus strain AA5	CC22-MRSA-IVa (dcs negatives) (tst1+), "Gaza Epidemic Strain"	NEG	NEG	POS	POS	NEG	NEG	NEG	POS	NEG	NEG	NEG	NEG
**CC152**													
JAEOUX Staphylococcus aureus strain AA17	CC152-MRSA-[V+fus]	NEG	NEG	NEG	NEG	NEG	NEG	NEG	NEG	NEG	NEG	NEG	NEG
**CC239**													
JAEOVI Staphylococcus aureus strain AA33	CC239-MRSA-[III+Cd/Hg+ccrC] (sasX-positive), "Southeast Asian Clade"	POS	AMB	POS	POS	NEG	POS	NEG	NEG	POS	NEG	NEG	NEG
JAEOUS Staphylococcus aureus strain AA101	CC239-MRSA-[III+Cd+ccrC] (sasX-negat.), "Middle Eastern Cluster"	POS	AMB	POS	POS	NEG	POS	NEG	NEG	POS	NEG	NEG	NEG
JAEOUV Staphylococcus aureus strain AA13	CC239-MRSA-[III+Cd+ccrC] (sasX-negat.), "Middle Eastern Cluster"	POS	AMB	POS	POS	NEG	POS	NEG	NEG	POS	NEG	NEG	NEG
JAEOUW Staphylococcus aureus strain AA14	CC239-MRSA-[III+Cd+ccrC] (sasX-negat.), "Middle Eastern Cluster"	POS	AMB	POS	POS	NEG	POS	NEG	NEG	POS	NEG	NEG	NEG
JAEOVA Staphylococcus aureus strain AA22	CC239-MRSA-[III+Cd+ccrC] (sasX-negat.), "Middle Eastern Cluster"	POS	AMB	POS	POS	NEG	POS	NEG	NEG	POS	NEG	NEG	NEG
JAEOVB Staphylococcus aureus strain AA23	CC239-MRSA-[III+Cd+ccrC] (sasX-negat.), "Middle Eastern Cluster"	POS	AMB	POS	POS	NEG	POS	NEG	NEG	POS	NEG	NEG	NEG
JAEOVC Staphylococcus aureus strain AA27	CC239-MRSA-[III+Cd+ccrC] (sasX-negat.), "Middle Eastern Cluster"	POS	AMB	POS	POS	NEG	POS	NEG	NEG	POS	NEG	NEG	NEG
JAEOVD Staphylococcus aureus strain AA29	CC239-MRSA-[III+Cd+ccrC] (sasX-negat.), "Middle Eastern Cluster"	POS	AMB	POS	POS	NEG	POS	NEG	NEG	POS	NEG	NEG	NEG
JAEOVG Staphylococcus aureus strain AA31	CC239-MRSA-[III+Cd+ccrC] (sasX-negat.), "Middle Eastern Cluster"	POS	AMB	POS	POS	NEG	POS	NEG	NEG	POS	NEG	NEG	NEG
JAEOVP Staphylococcus aureus strain AA46	CC239-MRSA-[III+Cd+ccrC] (sasX-negat.), "Middle Eastern Cluster"	POS	AMB	POS	POS	NEG	POS	NEG	NEG	POS	NEG	NEG	NEG
JAEOVS Staphylococcus aureus strain AA52	CC239-MRSA-[III+Cd+ccrC] (sasX-negat.), "Middle Eastern Cluster"	POS	AMB	POS	POS	NEG	POS	NEG	NEG	POS	NEG	NEG	NEG
JAEOVU Staphylococcus aureus strain AA55	CC239-MRSA-[III+Cd+ccrC] (sasX-negat.), "Middle Eastern Cluster"	POS	AMB	POS	POS	NEG	POS	NEG	NEG	POS	NEG	NEG	NEG
JAEOVV Staphylococcus aureus strain AA57	CC239-MRSA-[III+Cd+ccrC] (sasX-negat.), "Middle Eastern Cluster"	POS	AMB	POS	POS	NEG	POS	NEG	NEG	POS	NEG	NEG	NEG
JAEOVY Staphylococcus aureus strain AA60	CC239-MRSA-[III+Cd+ccrC] (sasX-negat.), "Middle Eastern Cluster"	POS	AMB	POS	POS	NEG	POS	NEG	NEG	POS	NEG	NEG	NEG
JAEOVZ Staphylococcus aureus strain AA61	CC239-MRSA-[III+Cd+ccrC] (sasX-negat.), "Middle Eastern Cluster"	POS	AMB	POS	POS	NEG	POS	NEG	NEG	POS	NEG	NEG	NEG
JAEOWA Staphylococcus aureus strain AA62	CC239-MRSA-[III+Cd+ccrC] (sasX-negat.), "Middle Eastern Cluster"	POS	AMB	POS	POS	NEG	POS	NEG	NEG	POS	NEG	NEG	NEG
JAEOWB Staphylococcus aureus strain AA63	CC239-MRSA-[III+Cd+ccrC] (sasX-negat.), "Middle Eastern Cluster"	POS	AMB	POS	POS	NEG	POS	NEG	NEG	POS	NEG	NEG	NEG
JAEOWL Staphylococcus aureus strain AA79	CC239-MRSA-[III+Cd+ccrC] (sasX-negat.), "Middle Eastern Cluster"	POS	AMB	POS	POS	NEG	POS	NEG	NEG	POS	NEG	NEG	NEG
JAEOWQ Staphylococcus aureus strain AA92	CC239-MRSA-[III+Cd+ccrC] (sasX-negat.), "Middle Eastern Cluster"	POS	AMB	POS	POS	NEG	POS	NEG	NEG	POS	NEG	NEG	NEG
JAEOWC Staphylococcus aureus strain AA64	CC239-MRSA-[mec III+Cd], sasX-negative	POS	AMB	POS	POS	NEG	POS	NEG	NEG	POS	NEG	NEG	NEG
JAEOWP Staphylococcus aureus strain AA91	CC239-MRSA-[mec III+Cd], sasX-negative	POS	AMB	POS	POS	NEG	POS	NEG	NEG	POS	NEG	NEG	NEG

Isolate	Strain assignment	VIRULENCE : SET/SSL GENES					CAPSULE- AND BIOFILM-ASSOCIATED GENES						
		setB3		setB2		setB1	Capsule type 1			Capsule type 5			
		setB3	setB3 (MRSA252)	setB2	setB2 (MRSA252)	setB1	cap 1	capH1	capJ1	cap 5	capH5	capJ5	capK5
		Staphylococcal exotoxin-like protein, second locus					Capsule type 1	capsular poly-saccharide synthesis enzyme	O-antigen polymerase	Capsule type 5	capsular poly-saccharide synthesis enzyme	O-antigen polymerase	capsular poly-saccharide biosyn-thesis protein
**CC1**													
Alexandria University_2020-17	CC1-MRSA-[V+fus+tir]	POS	NEG	POS	NEG	POS	NEG	NEG	NEG	NEG	NEG	NEG	NEG
Alexandria University_2020-03	CC1-MRSA-[V+fus+tir+ccrAB1]	POS	NEG	POS	NEG	POS	NEG	NEG	NEG	NEG	NEG	NEG	NEG
Alexandria University_2020-19	CC1-MRSA-[V+fus+tir+ccrAB1]	POS	NEG	POS	NEG	POS	NEG	NEG	NEG	NEG	NEG	NEG	NEG
Alexandria University_2020-21	CC1-MRSA-[V+fus+tir+ccrAB1]	POS	NEG	POS	NEG	POS	NEG	NEG	NEG	NEG	NEG	NEG	NEG
Alexandria University_2020-09	CC1-MRSA-[V+fus+tir+ccrAB1] (PVL+)	POS	NEG	POS	NEG	POS	NEG	NEG	NEG	NEG	NEG	NEG	NEG
Alexandria University_2020-14	CC1-MRSA-[V+fus+tir+ccrAB1] (PVL+)	POS	NEG	POS	NEG	POS	NEG	NEG	NEG	NEG	NEG	NEG	NEG
Alexandria University_2020-24	CC1-MRSA-[V+fus+tir+ccrAB1] (PVL+)	POS	NEG	POS	NEG	POS	NEG	NEG	NEG	NEG	NEG	NEG	NEG
Alexandria University_2020-27	CC1-MRSA-[V+fus+tir+ccrAB1] (PVL+)	POS	NEG	POS	NEG	POS	NEG	NEG	NEG	NEG	NEG	NEG	NEG
Alexandria University_2020-28	CC1-MRSA-[V+fus+tir+ccrAB1] (PVL+)	POS	NEG	POS	NEG	POS	NEG	NEG	NEG	NEG	NEG	NEG	NEG
**CC5**													
Alexandria University_2020-12	CC5-MRSA-[VI+fus+tir]	POS	NEG	POS	NEG	POS	NEG	NEG	NEG	POS	POS	POS	POS
Alexandria University_2020-20	CC5-MRSA-[VI+fus+tir]	POS	NEG	POS	NEG	POS	NEG	NEG	NEG	POS	POS	POS	POS
**CC6**													
Alexandria University_2020-29	CC6-MRSA-[V+fus]	POS	NEG	POS	NEG	POS	NEG	NEG	NEG	NEG	NEG	NEG	NEG
**CC15**													
Alexandria University_2020-01	CC15-MRSA-[V+fus]	POS	NEG	POS	NEG	POS	NEG	NEG	NEG	NEG	NEG	NEG	NEG
Alexandria University_2020-02	CC15-MRSA-[V+fus]	POS	NEG	POS	NEG	POS	NEG	NEG	NEG	NEG	NEG	NEG	NEG
Alexandria University_2020-05	CC15-MRSA-[V+fus]	POS	NEG	AMB	NEG	POS	NEG	NEG	NEG	NEG	NEG	NEG	NEG
Alexandria University_2020-15	CC15-MRSA-[V+fus]	POS	NEG	POS	NEG	POS	NEG	NEG	NEG	NEG	NEG	NEG	NEG
Alexandria University_2020-22	CC15-MRSA-[V+fus]	POS	NEG	NEG	NEG	POS	NEG	NEG	NEG	NEG	NEG	NEG	NEG
Alexandria University_2020-32	CC15-MRSA-[V+fus]	POS	NEG	POS	NEG	POS	NEG	NEG	NEG	NEG	NEG	NEG	NEG
**CC22**													
Alexandria University_2020-04	CC22-MRSA-IVa (dcs negatives) (tst1+), "Gaza Epidemic Strain"	NEG	NEG	NEG	NEG	NEG	NEG	NEG	NEG	POS	POS	POS	POS
Alexandria University_2020-11	CC22-MRSA-IVa (dcs negatives) (tst1+), "Gaza Epidemic Strain"	NEG	NEG	NEG	NEG	NEG	NEG	NEG	NEG	POS	POS	POS	POS
**CC30**													
Alexandria University_2020-26	CC30-MRSA-IVa (PVL+), "WSPP/Southwest Pacific Clone"	NEG	POS	NEG	POS	POS	NEG	NEG	NEG	NEG	NEG	NEG	NEG
**CC97**													
Alexandria University_2020-30	CC97-MRSA-[V+fus]	POS	NEG	POS	NEG	POS	NEG	NEG	NEG	POS	POS	POS	POS
**CC121**													
Alexandria University_2020-06	CC121-MRSA-[V+fus] (PVL+)	POS	NEG	POS	NEG	POS	NEG	NEG	NEG	NEG	NEG	NEG	NEG
**CC152**													
Alexandria University_2020-18	CC152-MRSA-[V+fus]	POS	NEG	NEG	NEG	POS	NEG	NEG	NEG	POS	POS	POS	POS
Alexandria University_2020-31	CC152-MRSA-[V+fus]	POS	NEG	POS	NEG	POS	NEG	NEG	NEG	POS	POS	POS	POS
**CC239**													
Alexandria University_2020-13	CC239-MRSA-[III+Cd+ccrC] (sasX-negat.), "Middle Eastern Cluster"	POS	NEG	POS	NEG	POS	NEG	NEG	NEG	NEG	NEG	NEG	NEG
Alexandria University_2020-16	CC239-MRSA-[III+Cd], sasX-negative	POS	NEG	POS	NEG	POS	NEG	NEG	NEG	NEG	NEG	NEG	NEG
**CC1153**													
Alexandria University_2020-07	CC1153-MRSA-[V+fus] (PVL+)	POS	NEG	AMB	NEG	POS	NEG	NEG	NEG	POS	POS	AMB	POS
Alexandria University_2020-08	CC1153-MRSA-[V+fus] (PVL+)	POS	NEG	POS	NEG	POS	NEG	NEG	NEG	POS	POS	POS	POS
Alexandria University_2020-23	CC1153-MRSA-[V+fus] (PVL+)	POS	NEG	POS	NEG	POS	NEG	NEG	NEG	POS	POS	POS	POS
**Simulated hybridisations for sequences from Montelongo et al., 2022**													
**CC1**													
JAEOUR Staphylococcus aureus strain AA1	CC1-MRSA-[V+fus+tir+ccrAB1]	POS	NEG	POS	NEG	POS	NEG	NEG	NEG	NEG	NEG	NEG	NEG
JAEOWK Staphylococcus aureus strain AA78	CC1-MRSA-[V+fus+tir+ccrAB1]	POS	NEG	POS	NEG	POS	NEG	NEG	NEG	NEG	NEG	NEG	NEG
JAEOVR Staphylococcus aureus strain AA51	CC1-MRSA-[V+fus+tir+ccrAB1] (PVL+)	POS	NEG	POS	NEG	POS	NEG	NEG	NEG	NEG	NEG	NEG	NEG
JAEOWE Staphylococcus aureus strain AA67	CC1-MRSA-[V+fus+tir+ccrAB1] (PVL+)	POS	NEG	POS	NEG	POS	NEG	NEG	NEG	NEG	NEG	NEG	NEG
JAEOWG Staphylococcus aureus strain AA69	CC1-MRSA-[V+fus+tir+ccrAB1] (PVL+)	POS	NEG	POS	NEG	POS	NEG	NEG	NEG	NEG	NEG	NEG	NEG
JAEOWJ Staphylococcus aureus strain AA77	CC1-MRSA-[V+fus+tir+ccrAB1] (PVL+)	POS	NEG	POS	NEG	POS	NEG	NEG	NEG	NEG	NEG	NEG	NEG
**CC5**													
JAEOWI Staphylococcus aureus strain AA76	CC5-MRSA-[V+cas], WA MRSA-123	POS	NEG	POS	NEG	POS	NEG	NEG	NEG	POS	POS	POS	POS
JAEOWN Staphylococcus aureus strain AA80	CC5-MRSA-[V+cas], WA MRSA-123	POS	NEG	POS	NEG	POS	NEG	NEG	NEG	POS	POS	POS	POS
JAEOWH Staphylococcus aureus strain AA70	CC5-MRSA-[VI+fus+tir+, dcs-]	POS	NEG	POS	NEG	POS	NEG	NEG	NEG	POS	POS	POS	POS
**CC6**													
JAEOVF Staphylococcus aureus strain AA30	CC6-MRSA-IVa, WA MRSA-51	POS	NEG	POS	NEG	POS	NEG	NEG	NEG	NEG	NEG	NEG	NEG
**CC80**													
JAEOVM Staphylococcus aureus strain AA4	CC80-MRSA-IVc (PVL-) [aphA3/sat+]:[far1+], contaminated	POS	NEG	POS	NEG	POS	NEG	NEG	NEG	NEG	NEG	NEG	NEG
JAEOUZ Staphylococcus aureus strain AA2	CC80-MRSA-IVc (PVL+) [aphA3/sat+]:[far1+]	POS	NEG	POS	NEG	POS	NEG	NEG	NEG	NEG	NEG	NEG	NEG
JAEOVE Staphylococcus aureus strain AA3	CC80-MRSA-IVc (PVL+) [aphA3/sat+]:[far1+]	POS	NEG	POS	NEG	POS	NEG	NEG	NEG	NEG	NEG	NEG	NEG
JAEOVO Staphylococcus aureus strain AA45	CC80-MRSA-IVc (PVL+) [aphA3/sat+]:[far1+]	POS	NEG	POS	NEG	POS	NEG	NEG	NEG	NEG	NEG	NEG	NEG
**CC88**													
JAEOVT Staphylococcus aureus strain AA53	CC88-MRSA-IV, contaminated	POS	NEG	POS	NEG	POS	NEG	NEG	NEG	POS	POS	POS	POS
**CC97**													
JAEOVL Staphylococcus aureus strain AA39	CC97-MRSA-IVc, WA MRSA-54/63	POS	NEG	POS	NEG	POS	NEG	NEG	NEG	POS	POS	POS	POS
JAEOVX Staphylococcus aureus strain AA6	CC97-MRSA-IVc, WA MRSA-54/63	POS	NEG	POS	NEG	POS	NEG	NEG	NEG	POS	POS	POS	POS
JAEOWM Staphylococcus aureus strain AA8	CC97-MRSA-IVc, WA MRSA-54/63	POS	NEG	POS	NEG	POS	NEG	NEG	NEG	POS	POS	POS	POS
JAEOUU Staphylococcus aureus strain AA104	CC97-MRSA-V	POS	NEG	POS	NEG	POS	NEG	NEG	NEG	POS	POS	POS	POS
JAEOVJ Staphylococcus aureus strain AA35	CC97-MRSA-[V+fus]	POS	NEG	POS	NEG	POS	NEG	NEG	NEG	POS	POS	POS	POS
JAEOVK Staphylococcus aureus strain AA36	CC97-MRSA-[V+fus]	POS	NEG	POS	NEG	POS	NEG	NEG	NEG	POS	POS	POS	POS
**CC22**													
JAEOUY Staphylococcus aureus strain AA18	CC22-MRSA-IVa (dcs negatives) (tst1+), "Gaza Epidemic Strain"	NEG	NEG	AMB	NEG	AMB	NEG	NEG	NEG	POS	POS	POS	POS
JAEOVH Staphylococcus aureus strain AA32	CC22-MRSA-IVa (dcs negatives) (tst1+), "Gaza Epidemic Strain"	NEG	NEG	AMB	NEG	AMB	NEG	NEG	NEG	POS	POS	POS	POS
JAEOVQ Staphylococcus aureus strain AA5	CC22-MRSA-IVa (dcs negatives) (tst1+), "Gaza Epidemic Strain"	NEG	NEG	AMB	NEG	AMB	NEG	NEG	NEG	POS	POS	POS	POS
**CC152**													
JAEOUX Staphylococcus aureus strain AA17	CC152-MRSA-[V+fus]	POS	NEG	POS	NEG	POS	NEG	NEG	NEG	AMB	AMB	AMB	AMB
**CC239**													
JAEOVI Staphylococcus aureus strain AA33	CC239-MRSA-[III+Cd/Hg+ccrC] (sasX-positive), "Southeast Asian Clade"	POS	NEG	POS	NEG	POS	NEG	NEG	NEG	NEG	NEG	NEG	NEG
JAEOUS Staphylococcus aureus strain AA101	CC239-MRSA-[III+Cd+ccrC] (sasX-negat.), "Middle Eastern Cluster"	POS	NEG	POS	NEG	POS	NEG	NEG	NEG	NEG	NEG	NEG	NEG
JAEOUV Staphylococcus aureus strain AA13	CC239-MRSA-[III+Cd+ccrC] (sasX-negat.), "Middle Eastern Cluster"	POS	NEG	POS	NEG	POS	NEG	NEG	NEG	NEG	NEG	NEG	NEG
JAEOUW Staphylococcus aureus strain AA14	CC239-MRSA-[III+Cd+ccrC] (sasX-negat.), "Middle Eastern Cluster"	POS	NEG	POS	NEG	POS	NEG	NEG	NEG	NEG	NEG	NEG	NEG
JAEOVA Staphylococcus aureus strain AA22	CC239-MRSA-[III+Cd+ccrC] (sasX-negat.), "Middle Eastern Cluster"	POS	NEG	POS	NEG	POS	NEG	NEG	NEG	NEG	NEG	NEG	NEG
JAEOVB Staphylococcus aureus strain AA23	CC239-MRSA-[III+Cd+ccrC] (sasX-negat.), "Middle Eastern Cluster"	POS	NEG	POS	NEG	POS	NEG	NEG	NEG	NEG	NEG	NEG	NEG
JAEOVC Staphylococcus aureus strain AA27	CC239-MRSA-[III+Cd+ccrC] (sasX-negat.), "Middle Eastern Cluster"	POS	NEG	POS	NEG	POS	NEG	NEG	NEG	NEG	NEG	NEG	NEG
JAEOVD Staphylococcus aureus strain AA29	CC239-MRSA-[III+Cd+ccrC] (sasX-negat.), "Middle Eastern Cluster"	POS	NEG	POS	NEG	POS	NEG	NEG	NEG	NEG	NEG	NEG	NEG
JAEOVG Staphylococcus aureus strain AA31	CC239-MRSA-[III+Cd+ccrC] (sasX-negat.), "Middle Eastern Cluster"	POS	NEG	POS	NEG	POS	NEG	NEG	NEG	NEG	NEG	NEG	NEG
JAEOVP Staphylococcus aureus strain AA46	CC239-MRSA-[III+Cd+ccrC] (sasX-negat.), "Middle Eastern Cluster"	POS	NEG	POS	NEG	POS	NEG	NEG	NEG	NEG	NEG	NEG	NEG
JAEOVS Staphylococcus aureus strain AA52	CC239-MRSA-[III+Cd+ccrC] (sasX-negat.), "Middle Eastern Cluster"	POS	NEG	POS	NEG	POS	NEG	NEG	NEG	NEG	NEG	NEG	NEG
JAEOVU Staphylococcus aureus strain AA55	CC239-MRSA-[III+Cd+ccrC] (sasX-negat.), "Middle Eastern Cluster"	POS	NEG	POS	NEG	POS	NEG	NEG	NEG	NEG	NEG	NEG	NEG
JAEOVV Staphylococcus aureus strain AA57	CC239-MRSA-[III+Cd+ccrC] (sasX-negat.), "Middle Eastern Cluster"	POS	NEG	POS	NEG	POS	NEG	NEG	NEG	NEG	NEG	NEG	NEG
JAEOVY Staphylococcus aureus strain AA60	CC239-MRSA-[III+Cd+ccrC] (sasX-negat.), "Middle Eastern Cluster"	POS	NEG	POS	NEG	POS	NEG	NEG	NEG	NEG	NEG	NEG	NEG
JAEOVZ Staphylococcus aureus strain AA61	CC239-MRSA-[III+Cd+ccrC] (sasX-negat.), "Middle Eastern Cluster"	POS	NEG	POS	NEG	POS	NEG	NEG	NEG	NEG	NEG	NEG	NEG
JAEOWA Staphylococcus aureus strain AA62	CC239-MRSA-[III+Cd+ccrC] (sasX-negat.), "Middle Eastern Cluster"	POS	NEG	POS	NEG	POS	NEG	NEG	NEG	NEG	NEG	NEG	NEG
JAEOWB Staphylococcus aureus strain AA63	CC239-MRSA-[III+Cd+ccrC] (sasX-negat.), "Middle Eastern Cluster"	POS	NEG	POS	NEG	POS	NEG	NEG	NEG	NEG	NEG	NEG	NEG
JAEOWL Staphylococcus aureus strain AA79	CC239-MRSA-[III+Cd+ccrC] (sasX-negat.), "Middle Eastern Cluster"	POS	NEG	POS	NEG	POS	NEG	NEG	NEG	NEG	NEG	NEG	NEG
JAEOWQ Staphylococcus aureus strain AA92	CC239-MRSA-[III+Cd+ccrC] (sasX-negat.), "Middle Eastern Cluster"	POS	NEG	POS	NEG	POS	NEG	NEG	NEG	NEG	NEG	NEG	NEG
JAEOWC Staphylococcus aureus strain AA64	CC239-MRSA-[mec III+Cd], sasX-negative	POS	NEG	POS	NEG	POS	NEG	NEG	NEG	NEG	NEG	NEG	NEG
JAEOWP Staphylococcus aureus strain AA91	CC239-MRSA-[mec III+Cd], sasX-negative	POS	NEG	POS	NEG	POS	NEG	NEG	NEG	NEG	NEG	NEG	NEG

Isolate	Strain assignment	CAPSULE- AND BIOFILM-ASSOCIATED GENES								
		Capsule type 8					icaA	icaC	icaD	bap
		cap 8	capH8	capI8	capJ8	capK8				
		Capsule type 8	capsular poly-saccharide synthesis enzyme	capsular poly-saccharide biosyn-thesis protein	O-antigen poly-merase	capsular poly-saccharide biosyn-thesis protein	intercellular adhesion protein A	intercellular adhesion protein C	biofilm PIA synthesis protein D	Surface protein involved in biofilm formation
**CC1**										
Alexandria University_2020-17	CC1-MRSA-[V+fus+tir]	POS	NEG	POS	POS	POS	POS	POS	POS	NEG
Alexandria University_2020-03	CC1-MRSA-[V+fus+tir+ccrAB1]	POS	NEG	POS	POS	POS	POS	POS	POS	NEG
Alexandria University_2020-19	CC1-MRSA-[V+fus+tir+ccrAB1]	POS	NEG	POS	POS	POS	POS	POS	POS	NEG
Alexandria University_2020-21	CC1-MRSA-[V+fus+tir+ccrAB1]	POS	NEG	POS	POS	POS	POS	POS	POS	NEG
Alexandria University_2020-09	CC1-MRSA-[V+fus+tir+ccrAB1] (PVL+)	POS	NEG	POS	POS	POS	POS	POS	POS	NEG
Alexandria University_2020-14	CC1-MRSA-[V+fus+tir+ccrAB1] (PVL+)	POS	NEG	POS	POS	POS	POS	POS	POS	NEG
Alexandria University_2020-24	CC1-MRSA-[V+fus+tir+ccrAB1] (PVL+)	POS	NEG	POS	POS	POS	POS	POS	POS	NEG
Alexandria University_2020-27	CC1-MRSA-[V+fus+tir+ccrAB1] (PVL+)	POS	NEG	POS	POS	POS	POS	POS	POS	NEG
Alexandria University_2020-28	CC1-MRSA-[V+fus+tir+ccrAB1] (PVL+)	POS	NEG	POS	POS	POS	POS	POS	POS	NEG
**CC5**										
Alexandria University_2020-12	CC5-MRSA-[VI+fus+tir]	NEG	NEG	NEG	NEG	NEG	POS	POS	POS	NEG
Alexandria University_2020-20	CC5-MRSA-[VI+fus+tir]	NEG	NEG	NEG	NEG	NEG	POS	POS	POS	NEG
**CC6**										
Alexandria University_2020-29	CC6-MRSA-[V+fus]	POS	POS	POS	POS	POS	POS	POS	POS	NEG
**CC15**										
Alexandria University_2020-01	CC15-MRSA-[V+fus]	POS	POS	POS	POS	POS	POS	POS	POS	NEG
Alexandria University_2020-02	CC15-MRSA-[V+fus]	POS	POS	POS	POS	POS	POS	POS	POS	NEG
Alexandria University_2020-05	CC15-MRSA-[V+fus]	POS	POS	POS	POS	POS	POS	POS	POS	NEG
Alexandria University_2020-15	CC15-MRSA-[V+fus]	POS	POS	POS	POS	POS	POS	POS	POS	NEG
Alexandria University_2020-22	CC15-MRSA-[V+fus]	POS	POS	POS	POS	POS	POS	POS	POS	NEG
Alexandria University_2020-32	CC15-MRSA-[V+fus]	POS	POS	POS	POS	POS	POS	POS	POS	NEG
**CC22**										
Alexandria University_2020-04	CC22-MRSA-IVa (dcs negatives) (tst1+), "Gaza Epidemic Strain"	NEG	NEG	NEG	NEG	NEG	POS	POS	POS	NEG
Alexandria University_2020-11	CC22-MRSA-IVa (dcs negatives) (tst1+), "Gaza Epidemic Strain"	NEG	NEG	NEG	NEG	NEG	POS	POS	POS	NEG
**CC30**										
Alexandria University_2020-26	CC30-MRSA-IVa (PVL+), "WSPP/Southwest Pacific Clone"	POS	POS	POS	POS	POS	POS	POS	POS	NEG
**CC97**										
Alexandria University_2020-30	CC97-MRSA-[V+fus]	NEG	NEG	NEG	NEG	NEG	POS	POS	POS	NEG
**CC121**										
Alexandria University_2020-06	CC121-MRSA-[V+fus] (PVL+)	POS	POS	POS	POS	POS	POS	POS	POS	NEG
**CC152**										
Alexandria University_2020-18	CC152-MRSA-[V+fus]	NEG	NEG	NEG	NEG	NEG	POS	NEG	POS	NEG
Alexandria University_2020-31	CC152-MRSA-[V+fus]	NEG	NEG	NEG	NEG	NEG	POS	NEG	POS	NEG
**CC239**										
Alexandria University_2020-13	CC239-MRSA-[III+Cd+ccrC] (sasX-negat.), "Middle Eastern Cluster"	POS	POS	POS	POS	POS	POS	POS	POS	NEG
Alexandria University_2020-16	CC239-MRSA-[III+Cd], sasX-negative	POS	POS	POS	POS	POS	POS	POS	POS	NEG
**CC1153**										
Alexandria University_2020-07	CC1153-MRSA-[V+fus] (PVL+)	NEG	NEG	NEG	NEG	NEG	POS	POS	POS	NEG
Alexandria University_2020-08	CC1153-MRSA-[V+fus] (PVL+)	NEG	NEG	NEG	NEG	NEG	POS	POS	POS	NEG
Alexandria University_2020-23	CC1153-MRSA-[V+fus] (PVL+)	NEG	NEG	NEG	NEG	NEG	POS	POS	POS	NEG
**Simulated hybridisations for sequences from Montelongo et al., 2022**										
**CC1**										
JAEOUR Staphylococcus aureus strain AA1	CC1-MRSA-[V+fus+tir+ccrAB1]	POS	NEG	POS	POS	POS	POS	POS	POS	NEG
JAEOWK Staphylococcus aureus strain AA78	CC1-MRSA-[V+fus+tir+ccrAB1]	POS	NEG	POS	POS	POS	POS	POS	POS	NEG
JAEOVR Staphylococcus aureus strain AA51	CC1-MRSA-[V+fus+tir+ccrAB1] (PVL+)	POS	NEG	POS	POS	POS	POS	POS	POS	NEG
JAEOWE Staphylococcus aureus strain AA67	CC1-MRSA-[V+fus+tir+ccrAB1] (PVL+)	POS	NEG	POS	POS	POS	POS	POS	POS	NEG
JAEOWG Staphylococcus aureus strain AA69	CC1-MRSA-[V+fus+tir+ccrAB1] (PVL+)	POS	NEG	POS	POS	POS	POS	POS	POS	NEG
JAEOWJ Staphylococcus aureus strain AA77	CC1-MRSA-[V+fus+tir+ccrAB1] (PVL+)	POS	NEG	POS	POS	POS	POS	POS	POS	NEG
**CC5**										
JAEOWI Staphylococcus aureus strain AA76	CC5-MRSA-[V+cas], WA MRSA-123	NEG	NEG	NEG	NEG	NEG	POS	POS	POS	NEG
JAEOWN Staphylococcus aureus strain AA80	CC5-MRSA-[V+cas], WA MRSA-123	NEG	NEG	NEG	NEG	NEG	POS	POS	POS	NEG
JAEOWH Staphylococcus aureus strain AA70	CC5-MRSA-[VI+fus+tir+, dcs-]	NEG	NEG	NEG	NEG	NEG	POS	POS	POS	NEG
**CC6**										
JAEOVF Staphylococcus aureus strain AA30	CC6-MRSA-IVa, WA MRSA-51	POS	POS	POS	AMB	POS	POS	POS	POS	NEG
**CC80**										
JAEOVM Staphylococcus aureus strain AA4	CC80-MRSA-IVc (PVL-) [aphA3/sat+]:[far1+], contaminated	POS	POS	POS	POS	POS	POS	POS	POS	NEG
JAEOUZ Staphylococcus aureus strain AA2	CC80-MRSA-IVc (PVL+) [aphA3/sat+]:[far1+]	POS	POS	POS	POS	POS	POS	POS	POS	NEG
JAEOVE Staphylococcus aureus strain AA3	CC80-MRSA-IVc (PVL+) [aphA3/sat+]:[far1+]	POS	POS	POS	POS	POS	POS	POS	POS	NEG
JAEOVO Staphylococcus aureus strain AA45	CC80-MRSA-IVc (PVL+) [aphA3/sat+]:[far1+]	POS	POS	POS	POS	POS	POS	POS	POS	NEG
**CC88**										
JAEOVT Staphylococcus aureus strain AA53	CC88-MRSA-IV, contaminated	POS	POS	POS	POS	POS	POS	POS	POS	NEG
**CC97**										
JAEOVL Staphylococcus aureus strain AA39	CC97-MRSA-IVc, WA MRSA-54/63	NEG	NEG	NEG	NEG	NEG	POS	POS	POS	NEG
JAEOVX Staphylococcus aureus strain AA6	CC97-MRSA-IVc, WA MRSA-54/63	NEG	NEG	NEG	NEG	NEG	POS	POS	POS	NEG
JAEOWM Staphylococcus aureus strain AA8	CC97-MRSA-IVc, WA MRSA-54/63	NEG	NEG	NEG	NEG	NEG	POS	POS	POS	NEG
JAEOUU Staphylococcus aureus strain AA104	CC97-MRSA-V	NEG	NEG	NEG	NEG	NEG	POS	POS	POS	NEG
JAEOVJ Staphylococcus aureus strain AA35	CC97-MRSA-[V+fus]	NEG	NEG	NEG	NEG	NEG	POS	POS	POS	NEG
JAEOVK Staphylococcus aureus strain AA36	CC97-MRSA-[V+fus]	NEG	NEG	NEG	NEG	NEG	POS	POS	POS	NEG
**CC22**										
JAEOUY Staphylococcus aureus strain AA18	CC22-MRSA-IVa (dcs negatives) (tst1+), "Gaza Epidemic Strain"	NEG	NEG	NEG	NEG	NEG	POS	POS	POS	NEG
JAEOVH Staphylococcus aureus strain AA32	CC22-MRSA-IVa (dcs negatives) (tst1+), "Gaza Epidemic Strain"	NEG	NEG	NEG	NEG	NEG	POS	POS	POS	NEG
JAEOVQ Staphylococcus aureus strain AA5	CC22-MRSA-IVa (dcs negatives) (tst1+), "Gaza Epidemic Strain"	NEG	NEG	NEG	NEG	NEG	POS	POS	POS	NEG
**CC152**										
JAEOUX Staphylococcus aureus strain AA17	CC152-MRSA-[V+fus]	NEG	NEG	NEG	NEG	NEG	POS	NEG	POS	NEG
**CC239**										
JAEOVI Staphylococcus aureus strain AA33	CC239-MRSA-[III+Cd/Hg+ccrC] (sasX-positive), "Southeast Asian Clade"	POS	POS	POS	POS	POS	POS	POS	POS	NEG
JAEOUS Staphylococcus aureus strain AA101	CC239-MRSA-[III+Cd+ccrC] (sasX-negat.), "Middle Eastern Cluster"	POS	POS	POS	POS	POS	POS	POS	POS	NEG
JAEOUV Staphylococcus aureus strain AA13	CC239-MRSA-[III+Cd+ccrC] (sasX-negat.), "Middle Eastern Cluster"	POS	POS	POS	POS	POS	POS	POS	POS	NEG
JAEOUW Staphylococcus aureus strain AA14	CC239-MRSA-[III+Cd+ccrC] (sasX-negat.), "Middle Eastern Cluster"	POS	POS	POS	POS	POS	POS	POS	POS	NEG
JAEOVA Staphylococcus aureus strain AA22	CC239-MRSA-[III+Cd+ccrC] (sasX-negat.), "Middle Eastern Cluster"	POS	POS	POS	POS	POS	POS	POS	POS	NEG
JAEOVB Staphylococcus aureus strain AA23	CC239-MRSA-[III+Cd+ccrC] (sasX-negat.), "Middle Eastern Cluster"	POS	POS	POS	POS	POS	POS	POS	POS	NEG
JAEOVC Staphylococcus aureus strain AA27	CC239-MRSA-[III+Cd+ccrC] (sasX-negat.), "Middle Eastern Cluster"	POS	POS	POS	POS	POS	POS	POS	POS	NEG
JAEOVD Staphylococcus aureus strain AA29	CC239-MRSA-[III+Cd+ccrC] (sasX-negat.), "Middle Eastern Cluster"	POS	POS	POS	POS	POS	POS	POS	POS	NEG
JAEOVG Staphylococcus aureus strain AA31	CC239-MRSA-[III+Cd+ccrC] (sasX-negat.), "Middle Eastern Cluster"	POS	POS	POS	POS	POS	POS	POS	POS	NEG
JAEOVP Staphylococcus aureus strain AA46	CC239-MRSA-[III+Cd+ccrC] (sasX-negat.), "Middle Eastern Cluster"	POS	POS	POS	POS	POS	POS	POS	POS	NEG
JAEOVS Staphylococcus aureus strain AA52	CC239-MRSA-[III+Cd+ccrC] (sasX-negat.), "Middle Eastern Cluster"	POS	POS	POS	POS	POS	POS	POS	POS	NEG
JAEOVU Staphylococcus aureus strain AA55	CC239-MRSA-[III+Cd+ccrC] (sasX-negat.), "Middle Eastern Cluster"	POS	POS	POS	POS	POS	POS	POS	POS	NEG
JAEOVV Staphylococcus aureus strain AA57	CC239-MRSA-[III+Cd+ccrC] (sasX-negat.), "Middle Eastern Cluster"	POS	POS	POS	POS	POS	POS	POS	POS	NEG
JAEOVY Staphylococcus aureus strain AA60	CC239-MRSA-[III+Cd+ccrC] (sasX-negat.), "Middle Eastern Cluster"	POS	POS	POS	POS	POS	POS	POS	POS	NEG
JAEOVZ Staphylococcus aureus strain AA61	CC239-MRSA-[III+Cd+ccrC] (sasX-negat.), "Middle Eastern Cluster"	POS	POS	POS	POS	POS	POS	POS	POS	NEG
JAEOWA Staphylococcus aureus strain AA62	CC239-MRSA-[III+Cd+ccrC] (sasX-negat.), "Middle Eastern Cluster"	POS	POS	POS	POS	POS	POS	POS	POS	NEG
JAEOWB Staphylococcus aureus strain AA63	CC239-MRSA-[III+Cd+ccrC] (sasX-negat.), "Middle Eastern Cluster"	POS	POS	POS	POS	POS	POS	POS	POS	NEG
JAEOWL Staphylococcus aureus strain AA79	CC239-MRSA-[III+Cd+ccrC] (sasX-negat.), "Middle Eastern Cluster"	POS	POS	POS	POS	POS	POS	POS	POS	NEG
JAEOWQ Staphylococcus aureus strain AA92	CC239-MRSA-[III+Cd+ccrC] (sasX-negat.), "Middle Eastern Cluster"	POS	POS	POS	POS	POS	POS	POS	POS	NEG
JAEOWC Staphylococcus aureus strain AA64	CC239-MRSA-[mec III+Cd], sasX-negative	POS	POS	POS	POS	POS	POS	POS	POS	NEG
JAEOWP Staphylococcus aureus strain AA91	CC239-MRSA-[mec III+Cd], sasX-negative	POS	POS	POS	POS	POS	POS	POS	POS	NEG

Isolate	Strain assignment	ADHAESION FACTORS / MSCRAMM GENES											
		bbp							clfA				
		bbp	bbp (cons)	bbp (COL+ MW2)	bbp (MRSA 252)	bbp (Mu50)	bbp (RF122)	bbp (ST45)	clfA	clfA (cons)	clfA (COL+ RF122)	clfA (MRSA 252)	clfA (Mu50+ MW2)
		Bone sialoprotein-binding protein							Clumping factor A				
**CC1**													
Alexandria University_2020-17	CC1-MRSA-[V+fus+tir]	POS	POS	POS	NEG	NEG	NEG	NEG	POS	POS	NEG	NEG	POS
Alexandria University_2020-03	CC1-MRSA-[V+fus+tir+ccrAB1]	POS	POS	POS	NEG	NEG	NEG	NEG	POS	POS	NEG	NEG	POS
Alexandria University_2020-19	CC1-MRSA-[V+fus+tir+ccrAB1]	POS	POS	POS	NEG	NEG	NEG	NEG	POS	POS	NEG	NEG	POS
Alexandria University_2020-21	CC1-MRSA-[V+fus+tir+ccrAB1]	POS	POS	POS	NEG	NEG	NEG	NEG	POS	POS	NEG	NEG	POS
Alexandria University_2020-09	CC1-MRSA-[V+fus+tir+ccrAB1] (PVL+)	POS	POS	POS	NEG	NEG	NEG	NEG	POS	POS	NEG	NEG	POS
Alexandria University_2020-14	CC1-MRSA-[V+fus+tir+ccrAB1] (PVL+)	POS	POS	POS	NEG	NEG	NEG	NEG	POS	POS	NEG	NEG	POS
Alexandria University_2020-24	CC1-MRSA-[V+fus+tir+ccrAB1] (PVL+)	POS	POS	POS	NEG	NEG	NEG	NEG	POS	POS	NEG	NEG	POS
Alexandria University_2020-27	CC1-MRSA-[V+fus+tir+ccrAB1] (PVL+)	POS	POS	POS	NEG	NEG	NEG	NEG	POS	POS	NEG	NEG	POS
Alexandria University_2020-28	CC1-MRSA-[V+fus+tir+ccrAB1] (PVL+)	POS	POS	POS	NEG	NEG	NEG	NEG	POS	POS	NEG	NEG	POS
**CC5**													
Alexandria University_2020-12	CC5-MRSA-[VI+fus+tir]	POS	POS	NEG	NEG	POS	NEG	AMB	POS	POS	NEG	NEG	POS
Alexandria University_2020-20	CC5-MRSA-[VI+fus+tir]	POS	POS	NEG	NEG	POS	NEG	NEG	POS	POS	NEG	NEG	POS
**CC6**													
Alexandria University_2020-29	CC6-MRSA-[V+fus]	POS	POS	POS	NEG	NEG	NEG	NEG	POS	POS	NEG	NEG	POS
**CC15**													
Alexandria University_2020-01	CC15-MRSA-[V+fus]	POS	POS	NEG	NEG	POS	NEG	NEG	POS	POS	NEG	NEG	POS
Alexandria University_2020-02	CC15-MRSA-[V+fus]	POS	POS	NEG	NEG	POS	NEG	NEG	POS	POS	NEG	NEG	POS
Alexandria University_2020-05	CC15-MRSA-[V+fus]	POS	POS	NEG	NEG	POS	NEG	NEG	POS	POS	NEG	NEG	POS
Alexandria University_2020-15	CC15-MRSA-[V+fus]	POS	POS	NEG	NEG	POS	NEG	NEG	POS	POS	NEG	NEG	POS
Alexandria University_2020-22	CC15-MRSA-[V+fus]	POS	POS	NEG	NEG	POS	NEG	NEG	POS	POS	NEG	NEG	POS
Alexandria University_2020-32	CC15-MRSA-[V+fus]	POS	POS	NEG	NEG	POS	NEG	NEG	POS	POS	NEG	NEG	POS
**CC22**													
Alexandria University_2020-04	CC22-MRSA-IVa (dcs negatives) (tst1+), "Gaza Epidemic Strain"	POS	POS	NEG	NEG	NEG	NEG	NEG	POS	POS	NEG	NEG	POS
Alexandria University_2020-11	CC22-MRSA-IVa (dcs negatives) (tst1+), "Gaza Epidemic Strain"	POS	POS	NEG	NEG	NEG	NEG	NEG	POS	POS	NEG	NEG	POS
**CC30**													
Alexandria University_2020-26	CC30-MRSA-IVa (PVL+), "WSPP/Southwest Pacific Clone"	POS	POS	NEG	POS	NEG	NEG	NEG	POS	POS	AMB	POS	NEG
**CC97**													
Alexandria University_2020-30	CC97-MRSA-[V+fus]	POS	POS	NEG	NEG	POS	NEG	NEG	POS	POS	POS	AMB	AMB
**CC121**													
Alexandria University_2020-06	CC121-MRSA-[V+fus] (PVL+)	POS	POS	NEG	POS	NEG	NEG	AMB	POS	POS	NEG	NEG	POS
**CC152**													
Alexandria University_2020-18	CC152-MRSA-[V+fus]	POS	POS	POS	NEG	NEG	NEG	NEG	POS	POS	POS	AMB	AMB
Alexandria University_2020-31	CC152-MRSA-[V+fus]	POS	POS	POS	NEG	NEG	NEG	NEG	POS	POS	POS	AMB	AMB
**CC239**													
Alexandria University_2020-13	CC239-MRSA-[III+Cd+ccrC] (sasX-negat.), "Middle Eastern Cluster"	POS	POS	POS	NEG	NEG	NEG	NEG	POS	POS	POS	AMB	AMB
Alexandria University_2020-16	CC239-MRSA-[III+Cd], sasX-negative	POS	POS	POS	NEG	NEG	NEG	NEG	POS	POS	POS	NEG	AMB
**CC1153**													
Alexandria University_2020-07	CC1153-MRSA-[V+fus] (PVL+)	POS	POS	POS	NEG	NEG	NEG	AMB	POS	POS	NEG	NEG	POS
Alexandria University_2020-08	CC1153-MRSA-[V+fus] (PVL+)	POS	POS	POS	NEG	NEG	NEG	AMB	POS	POS	NEG	NEG	POS
Alexandria University_2020-23	CC1153-MRSA-[V+fus] (PVL+)	POS	POS	POS	NEG	NEG	NEG	NEG	POS	POS	NEG	NEG	POS
**Simulated hybridisations for sequences from Montelongo et al., 2022**													
**CC1**													
JAEOUR Staphylococcus aureus strain AA1	CC1-MRSA-[V+fus+tir+ccrAB1]	POS	POS	POS	NEG	NEG	NEG	NEG	POS	POS	NEG	NEG	POS
JAEOWK Staphylococcus aureus strain AA78	CC1-MRSA-[V+fus+tir+ccrAB1]	POS	POS	POS	NEG	NEG	NEG	NEG	POS	POS	NEG	NEG	POS
JAEOVR Staphylococcus aureus strain AA51	CC1-MRSA-[V+fus+tir+ccrAB1] (PVL+)	POS	POS	POS	NEG	NEG	NEG	NEG	POS	POS	NEG	NEG	POS
JAEOWE Staphylococcus aureus strain AA67	CC1-MRSA-[V+fus+tir+ccrAB1] (PVL+)	POS	POS	POS	NEG	NEG	NEG	NEG	POS	POS	NEG	NEG	POS
JAEOWG Staphylococcus aureus strain AA69	CC1-MRSA-[V+fus+tir+ccrAB1] (PVL+)	POS	POS	POS	NEG	NEG	NEG	NEG	POS	POS	NEG	NEG	POS
JAEOWJ Staphylococcus aureus strain AA77	CC1-MRSA-[V+fus+tir+ccrAB1] (PVL+)	POS	POS	POS	NEG	NEG	NEG	NEG	POS	POS	NEG	NEG	POS
**CC5**													
JAEOWI Staphylococcus aureus strain AA76	CC5-MRSA-[V+cas], WA MRSA-123	POS	POS	NEG	NEG	POS	NEG	NEG	POS	POS	NEG	NEG	POS
JAEOWN Staphylococcus aureus strain AA80	CC5-MRSA-[V+cas], WA MRSA-123	POS	POS	NEG	NEG	POS	NEG	NEG	POS	POS	NEG	NEG	POS
JAEOWH Staphylococcus aureus strain AA70	CC5-MRSA-[VI+fus+tir+, dcs-]	POS	POS	NEG	NEG	POS	NEG	NEG	POS	POS	NEG	NEG	POS
**CC6**													
JAEOVF Staphylococcus aureus strain AA30	CC6-MRSA-IVa, WA MRSA-51	NEG	NEG	NEG	NEG	NEG	NEG	NEG	POS	AMB	NEG	NEG	POS
**CC80**													
JAEOVM Staphylococcus aureus strain AA4	CC80-MRSA-IVc (PVL-) [aphA3/sat+]:[far1+], contaminated	POS	POS	NEG	NEG	POS	NEG	NEG	POS	POS	NEG	NEG	POS
JAEOUZ Staphylococcus aureus strain AA2	CC80-MRSA-IVc (PVL+) [aphA3/sat+]:[far1+]	POS	POS	NEG	NEG	POS	NEG	NEG	POS	POS	NEG	NEG	POS
JAEOVE Staphylococcus aureus strain AA3	CC80-MRSA-IVc (PVL+) [aphA3/sat+]:[far1+]	POS	POS	NEG	NEG	POS	NEG	NEG	POS	POS	NEG	NEG	POS
JAEOVO Staphylococcus aureus strain AA45	CC80-MRSA-IVc (PVL+) [aphA3/sat+]:[far1+]	POS	POS	NEG	NEG	POS	NEG	NEG	POS	POS	NEG	NEG	POS
**CC88**													
JAEOVT Staphylococcus aureus strain AA53	CC88-MRSA-IV, contaminated	POS	POS	NEG	NEG	POS	NEG	NEG	POS	POS	NEG	NEG	POS
**CC97**													
JAEOVL Staphylococcus aureus strain AA39	CC97-MRSA-IVc, WA MRSA-54/63	POS	POS	NEG	NEG	POS	NEG	NEG	POS	POS	POS	NEG	AMB
JAEOVX Staphylococcus aureus strain AA6	CC97-MRSA-IVc, WA MRSA-54/63	POS	POS	NEG	NEG	POS	NEG	NEG	POS	POS	POS	NEG	AMB
JAEOWM Staphylococcus aureus strain AA8	CC97-MRSA-IVc, WA MRSA-54/63	POS	POS	NEG	NEG	POS	NEG	NEG	POS	POS	POS	NEG	AMB
JAEOUU Staphylococcus aureus strain AA104	CC97-MRSA-V	POS	POS	NEG	NEG	POS	NEG	NEG	POS	POS	POS	NEG	NEG
JAEOVJ Staphylococcus aureus strain AA35	CC97-MRSA-[V+fus]	POS	POS	NEG	NEG	POS	NEG	NEG	POS	POS	POS	NEG	NEG
JAEOVK Staphylococcus aureus strain AA36	CC97-MRSA-[V+fus]	POS	POS	NEG	NEG	POS	NEG	NEG	POS	POS	POS	NEG	NEG
**CC22**													
JAEOUY Staphylococcus aureus strain AA18	CC22-MRSA-IVa (dcs negatives) (tst1+), "Gaza Epidemic Strain"	POS	POS	NEG	NEG	NEG	NEG	NEG	POS	POS	NEG	NEG	POS
JAEOVH Staphylococcus aureus strain AA32	CC22-MRSA-IVa (dcs negatives) (tst1+), "Gaza Epidemic Strain"	POS	POS	NEG	NEG	NEG	NEG	NEG	POS	POS	NEG	NEG	POS
JAEOVQ Staphylococcus aureus strain AA5	CC22-MRSA-IVa (dcs negatives) (tst1+), "Gaza Epidemic Strain"	POS	POS	NEG	NEG	NEG	NEG	NEG	POS	POS	NEG	NEG	POS
**CC152**													
JAEOUX Staphylococcus aureus strain AA17	CC152-MRSA-[V+fus]	POS	POS	POS	NEG	NEG	NEG	NEG	POS	AMB	POS	NEG	AMB
**CC239**													
JAEOVI Staphylococcus aureus strain AA33	CC239-MRSA-[III+Cd/Hg+ccrC] (sasX-positive), "Southeast Asian Clade"	POS	POS	POS	NEG	NEG	NEG	NEG	POS	AMB	POS	NEG	NEG
JAEOUS Staphylococcus aureus strain AA101	CC239-MRSA-[III+Cd+ccrC] (sasX-negat.), "Middle Eastern Cluster"	POS	POS	POS	NEG	NEG	NEG	NEG	POS	AMB	POS	NEG	NEG
JAEOUV Staphylococcus aureus strain AA13	CC239-MRSA-[III+Cd+ccrC] (sasX-negat.), "Middle Eastern Cluster"	POS	POS	POS	NEG	NEG	NEG	NEG	POS	AMB	POS	NEG	AMB
JAEOUW Staphylococcus aureus strain AA14	CC239-MRSA-[III+Cd+ccrC] (sasX-negat.), "Middle Eastern Cluster"	POS	POS	POS	NEG	NEG	NEG	NEG	POS	AMB	POS	NEG	NEG
JAEOVA Staphylococcus aureus strain AA22	CC239-MRSA-[III+Cd+ccrC] (sasX-negat.), "Middle Eastern Cluster"	POS	POS	POS	NEG	NEG	NEG	NEG	POS	AMB	POS	NEG	AMB
JAEOVB Staphylococcus aureus strain AA23	CC239-MRSA-[III+Cd+ccrC] (sasX-negat.), "Middle Eastern Cluster"	POS	POS	POS	NEG	NEG	NEG	NEG	POS	AMB	POS	NEG	NEG
JAEOVC Staphylococcus aureus strain AA27	CC239-MRSA-[III+Cd+ccrC] (sasX-negat.), "Middle Eastern Cluster"	POS	POS	POS	NEG	NEG	NEG	NEG	POS	AMB	POS	NEG	NEG
JAEOVD Staphylococcus aureus strain AA29	CC239-MRSA-[III+Cd+ccrC] (sasX-negat.), "Middle Eastern Cluster"	POS	POS	POS	NEG	NEG	NEG	NEG	POS	AMB	POS	NEG	NEG
JAEOVG Staphylococcus aureus strain AA31	CC239-MRSA-[III+Cd+ccrC] (sasX-negat.), "Middle Eastern Cluster"	POS	POS	POS	NEG	NEG	NEG	NEG	POS	POS	POS	NEG	NEG
JAEOVP Staphylococcus aureus strain AA46	CC239-MRSA-[III+Cd+ccrC] (sasX-negat.), "Middle Eastern Cluster"	POS	POS	POS	NEG	NEG	NEG	NEG	POS	AMB	POS	NEG	AMB
JAEOVS Staphylococcus aureus strain AA52	CC239-MRSA-[III+Cd+ccrC] (sasX-negat.), "Middle Eastern Cluster"	POS	POS	POS	NEG	NEG	NEG	NEG	POS	AMB	POS	NEG	AMB
JAEOVU Staphylococcus aureus strain AA55	CC239-MRSA-[III+Cd+ccrC] (sasX-negat.), "Middle Eastern Cluster"	POS	POS	POS	NEG	NEG	NEG	NEG	POS	AMB	POS	NEG	AMB
JAEOVV Staphylococcus aureus strain AA57	CC239-MRSA-[III+Cd+ccrC] (sasX-negat.), "Middle Eastern Cluster"	POS	POS	POS	NEG	NEG	NEG	NEG	POS	AMB	POS	NEG	NEG
JAEOVY Staphylococcus aureus strain AA60	CC239-MRSA-[III+Cd+ccrC] (sasX-negat.), "Middle Eastern Cluster"	POS	POS	POS	NEG	NEG	NEG	NEG	POS	AMB	POS	NEG	AMB
JAEOVZ Staphylococcus aureus strain AA61	CC239-MRSA-[III+Cd+ccrC] (sasX-negat.), "Middle Eastern Cluster"	POS	POS	POS	NEG	NEG	NEG	NEG	POS	AMB	POS	NEG	AMB
JAEOWA Staphylococcus aureus strain AA62	CC239-MRSA-[III+Cd+ccrC] (sasX-negat.), "Middle Eastern Cluster"	POS	POS	POS	NEG	NEG	NEG	NEG	POS	AMB	POS	NEG	NEG
JAEOWB Staphylococcus aureus strain AA63	CC239-MRSA-[III+Cd+ccrC] (sasX-negat.), "Middle Eastern Cluster"	POS	POS	POS	NEG	NEG	NEG	NEG	POS	AMB	POS	NEG	AMB
JAEOWL Staphylococcus aureus strain AA79	CC239-MRSA-[III+Cd+ccrC] (sasX-negat.), "Middle Eastern Cluster"	POS	POS	POS	NEG	NEG	NEG	NEG	POS	AMB	POS	NEG	NEG
JAEOWQ Staphylococcus aureus strain AA92	CC239-MRSA-[III+Cd+ccrC] (sasX-negat.), "Middle Eastern Cluster"	POS	POS	POS	NEG	NEG	NEG	NEG	POS	AMB	POS	NEG	AMB
JAEOWC Staphylococcus aureus strain AA64	CC239-MRSA-[mec III+Cd], sasX-negative	POS	POS	POS	NEG	NEG	NEG	NEG	POS	AMB	POS	NEG	NEG
JAEOWP Staphylococcus aureus strain AA91	CC239-MRSA-[mec III+Cd], sasX-negative	POS	POS	POS	NEG	NEG	NEG	NEG	POS	AMB	POS	NEG	AMB

Isolate	Strain assignment	ADHAESION FACTORS / MSCRAMM GENES												
		clfB					cna	ebh	ebpS					eno
		clfB	clfB (cons)	clfB (COL+Mu50)	clfB (MW2)	clfB (RF122)		ebh (cons)	ebpS	ebpS_probe 612	ebpS_probe 614	ebpS (01-1111)	ebpS (COL)	
		Clumping factor B					Collagen-binding adhesin	Cell wall associated fibronectin-binding protein	cell surface elastin binding protein					enolase
**CC1**														
Alexandria University_2020-17	CC1-MRSA-[V+fus+tir]	POS	POS	NEG	POS	AMB	POS	POS	POS	POS	POS	NEG	NEG	POS
Alexandria University_2020-03	CC1-MRSA-[V+fus+tir+ccrAB1]	POS	POS	NEG	POS	AMB	POS	POS	POS	POS	POS	NEG	NEG	POS
Alexandria University_2020-19	CC1-MRSA-[V+fus+tir+ccrAB1]	POS	POS	NEG	POS	AMB	POS	POS	POS	AMB	POS	NEG	NEG	POS
Alexandria University_2020-21	CC1-MRSA-[V+fus+tir+ccrAB1]	POS	POS	NEG	POS	AMB	POS	POS	POS	POS	POS	NEG	NEG	POS
Alexandria University_2020-09	CC1-MRSA-[V+fus+tir+ccrAB1] (PVL+)	POS	POS	NEG	POS	AMB	POS	POS	POS	POS	POS	NEG	NEG	POS
Alexandria University_2020-14	CC1-MRSA-[V+fus+tir+ccrAB1] (PVL+)	POS	POS	NEG	POS	AMB	POS	POS	POS	POS	POS	NEG	NEG	POS
Alexandria University_2020-24	CC1-MRSA-[V+fus+tir+ccrAB1] (PVL+)	POS	POS	NEG	POS	AMB	POS	POS	POS	POS	POS	NEG	NEG	POS
Alexandria University_2020-27	CC1-MRSA-[V+fus+tir+ccrAB1] (PVL+)	POS	POS	NEG	POS	AMB	POS	POS	POS	POS	POS	NEG	NEG	POS
Alexandria University_2020-28	CC1-MRSA-[V+fus+tir+ccrAB1] (PVL+)	POS	POS	NEG	POS	AMB	POS	POS	POS	NEG	POS	NEG	NEG	POS
**CC5**														
Alexandria University_2020-12	CC5-MRSA-[VI+fus+tir]	POS	POS	POS	NEG	NEG	NEG	POS	POS	POS	POS	NEG	NEG	POS
Alexandria University_2020-20	CC5-MRSA-[VI+fus+tir]	POS	POS	POS	NEG	NEG	NEG	POS	POS	POS	POS	NEG	NEG	POS
**CC6**														
Alexandria University_2020-29	CC6-MRSA-[V+fus]	POS	POS	NEG	NEG	NEG	POS	POS	POS	POS	POS	NEG	NEG	POS
**CC15**														
Alexandria University_2020-01	CC15-MRSA-[V+fus]	POS	POS	NEG	POS	AMB	NEG	POS	POS	AMB	POS	NEG	POS	POS
Alexandria University_2020-02	CC15-MRSA-[V+fus]	POS	POS	NEG	POS	AMB	NEG	POS	POS	POS	POS	NEG	POS	POS
Alexandria University_2020-05	CC15-MRSA-[V+fus]	POS	POS	NEG	POS	AMB	NEG	POS	POS	AMB	POS	NEG	POS	POS
Alexandria University_2020-15	CC15-MRSA-[V+fus]	POS	POS	NEG	POS	AMB	NEG	POS	POS	POS	POS	NEG	POS	POS
Alexandria University_2020-22	CC15-MRSA-[V+fus]	POS	POS	NEG	POS	AMB	NEG	POS	POS	NEG	POS	NEG	POS	POS
Alexandria University_2020-32	CC15-MRSA-[V+fus]	POS	POS	NEG	POS	AMB	NEG	POS	POS	POS	POS	NEG	POS	POS
**CC22**														
Alexandria University_2020-04	CC22-MRSA-IVa (dcs negatives) (tst1+), "Gaza Epidemic Strain"	POS	POS	NEG	NEG	NEG	POS	NEG	POS	POS	POS	NEG	POS	POS
Alexandria University_2020-11	CC22-MRSA-IVa (dcs negatives) (tst1+), "Gaza Epidemic Strain"	POS	POS	NEG	NEG	POS	POS	NEG	POS	POS	POS	NEG	POS	POS
**CC30**														
Alexandria University_2020-26	CC30-MRSA-IVa (PVL+), "WSPP/Southwest Pacific Clone"	POS	POS	NEG	NEG	NEG	POS	POS	POS	POS	POS	NEG	NEG	POS
**CC97**														
Alexandria University_2020-30	CC97-MRSA-[V+fus]	POS	POS	NEG	NEG	NEG	NEG	POS	POS	POS	POS	NEG	NEG	POS
**CC121**														
Alexandria University_2020-06	CC121-MRSA-[V+fus] (PVL+)	POS	POS	NEG	NEG	POS	POS	POS	POS	NEG	POS	AMB	NEG	POS
**CC152**														
Alexandria University_2020-18	CC152-MRSA-[V+fus]	POS	POS	NEG	AMB	POS	POS	POS	POS	POS	POS	NEG	NEG	POS
Alexandria University_2020-31	CC152-MRSA-[V+fus]	POS	POS	NEG	AMB	POS	POS	POS	POS	POS	POS	NEG	NEG	POS
**CC239**														
Alexandria University_2020-13	CC239-MRSA-[III+Cd+ccrC] (sasX-negat.), "Middle Eastern Cluster"	POS	POS	NEG	NEG	POS	POS	POS	POS	POS	POS	NEG	POS	POS
Alexandria University_2020-16	CC239-MRSA-[III+Cd], sasX-negative	POS	POS	NEG	NEG	NEG	POS	POS	POS	AMB	POS	NEG	POS	POS
**CC1153**														
Alexandria University_2020-07	CC1153-MRSA-[V+fus] (PVL+)	POS	POS	NEG	NEG	NEG	NEG	AMB	POS	AMB	POS	NEG	AMB	POS
Alexandria University_2020-08	CC1153-MRSA-[V+fus] (PVL+)	POS	POS	NEG	NEG	NEG	NEG	POS	POS	AMB	POS	NEG	POS	POS
Alexandria University_2020-23	CC1153-MRSA-[V+fus] (PVL+)	POS	POS	NEG	NEG	POS	NEG	POS	POS	POS	POS	NEG	POS	POS
**Simulated hybridisations for sequences from Montelongo et al., 2022**														
**CC1**														
JAEOUR Staphylococcus aureus strain AA1	CC1-MRSA-[V+fus+tir+ccrAB1]	POS	POS	NEG	POS	NEG	POS	POS	POS	POS	POS	NEG	NEG	POS
JAEOWK Staphylococcus aureus strain AA78	CC1-MRSA-[V+fus+tir+ccrAB1]	POS	POS	NEG	POS	NEG	POS	POS	POS	POS	POS	NEG	NEG	POS
JAEOVR Staphylococcus aureus strain AA51	CC1-MRSA-[V+fus+tir+ccrAB1] (PVL+)	POS	POS	NEG	POS	NEG	POS	POS	POS	POS	POS	NEG	NEG	POS
JAEOWE Staphylococcus aureus strain AA67	CC1-MRSA-[V+fus+tir+ccrAB1] (PVL+)	POS	POS	NEG	POS	NEG	POS	POS	POS	POS	POS	NEG	NEG	POS
JAEOWG Staphylococcus aureus strain AA69	CC1-MRSA-[V+fus+tir+ccrAB1] (PVL+)	POS	POS	NEG	POS	NEG	POS	POS	POS	POS	POS	NEG	NEG	POS
JAEOWJ Staphylococcus aureus strain AA77	CC1-MRSA-[V+fus+tir+ccrAB1] (PVL+)	POS	POS	NEG	POS	NEG	POS	POS	POS	POS	POS	NEG	NEG	POS
**CC5**														
JAEOWI Staphylococcus aureus strain AA76	CC5-MRSA-[V+cas], WA MRSA-123	POS	POS	POS	NEG	NEG	NEG	POS	POS	POS	POS	NEG	NEG	POS
JAEOWN Staphylococcus aureus strain AA80	CC5-MRSA-[V+cas], WA MRSA-123	POS	POS	POS	NEG	NEG	NEG	POS	POS	POS	POS	NEG	NEG	POS
JAEOWH Staphylococcus aureus strain AA70	CC5-MRSA-[VI+fus+tir+, dcs-]	POS	POS	POS	NEG	NEG	NEG	POS	POS	POS	POS	NEG	NEG	POS
**CC6**														
JAEOVF Staphylococcus aureus strain AA30	CC6-MRSA-IVa, WA MRSA-51	POS	POS	NEG	NEG	NEG	POS	POS	POS	AMB	POS	NEG	NEG	POS
**CC80**														
JAEOVM Staphylococcus aureus strain AA4	CC80-MRSA-IVc (PVL-) [aphA3/sat+]:[far1+], contaminated	POS	POS	POS	NEG	NEG	NEG	POS	POS	POS	POS	NEG	NEG	POS
JAEOUZ Staphylococcus aureus strain AA2	CC80-MRSA-IVc (PVL+) [aphA3/sat+]:[far1+]	POS	POS	POS	NEG	NEG	NEG	POS	POS	POS	POS	NEG	NEG	POS
JAEOVE Staphylococcus aureus strain AA3	CC80-MRSA-IVc (PVL+) [aphA3/sat+]:[far1+]	POS	POS	POS	NEG	NEG	NEG	POS	POS	POS	POS	NEG	NEG	POS
JAEOVO Staphylococcus aureus strain AA45	CC80-MRSA-IVc (PVL+) [aphA3/sat+]:[far1+]	POS	POS	POS	NEG	NEG	NEG	POS	POS	POS	POS	NEG	NEG	POS
**CC88**														
JAEOVT Staphylococcus aureus strain AA53	CC88-MRSA-IV, contaminated	POS	POS	NEG	NEG	NEG	NEG	POS	POS	AMB	POS	NEG	POS	POS
**CC97**														
JAEOVL Staphylococcus aureus strain AA39	CC97-MRSA-IVc, WA MRSA-54/63	POS	POS	NEG	NEG	NEG	NEG	POS	POS	POS	POS	NEG	NEG	POS
JAEOVX Staphylococcus aureus strain AA6	CC97-MRSA-IVc, WA MRSA-54/63	POS	POS	NEG	NEG	NEG	NEG	POS	POS	POS	POS	NEG	NEG	POS
JAEOWM Staphylococcus aureus strain AA8	CC97-MRSA-IVc, WA MRSA-54/63	POS	POS	NEG	NEG	NEG	NEG	POS	POS	POS	POS	NEG	NEG	POS
JAEOUU Staphylococcus aureus strain AA104	CC97-MRSA-V	POS	POS	NEG	NEG	NEG	NEG	POS	POS	POS	POS	NEG	NEG	POS
JAEOVJ Staphylococcus aureus strain AA35	CC97-MRSA-[V+fus]	POS	POS	NEG	NEG	NEG	NEG	POS	POS	POS	POS	NEG	NEG	POS
JAEOVK Staphylococcus aureus strain AA36	CC97-MRSA-[V+fus]	POS	POS	NEG	NEG	NEG	NEG	POS	POS	POS	POS	NEG	NEG	POS
**CC22**														
JAEOUY Staphylococcus aureus strain AA18	CC22-MRSA-IVa (dcs negatives) (tst1+), "Gaza Epidemic Strain"	POS	POS	NEG	NEG	NEG	POS	NEG	POS	AMB	POS	NEG	POS	POS
JAEOVH Staphylococcus aureus strain AA32	CC22-MRSA-IVa (dcs negatives) (tst1+), "Gaza Epidemic Strain"	POS	POS	NEG	NEG	NEG	POS	NEG	POS	AMB	POS	NEG	POS	POS
JAEOVQ Staphylococcus aureus strain AA5	CC22-MRSA-IVa (dcs negatives) (tst1+), "Gaza Epidemic Strain"	POS	POS	NEG	NEG	NEG	POS	NEG	POS	AMB	POS	NEG	POS	POS
**CC152**														
JAEOUX Staphylococcus aureus strain AA17	CC152-MRSA-[V+fus]	POS	POS	NEG	POS	NEG	AMB	POS	POS	POS	POS	NEG	NEG	POS
**CC239**														
JAEOVI Staphylococcus aureus strain AA33	CC239-MRSA-[III+Cd/Hg+ccrC] (sasX-positive), "Southeast Asian Clade"	POS	POS	NEG	NEG	NEG	POS	POS	POS	POS	POS	NEG	POS	POS
JAEOUS Staphylococcus aureus strain AA101	CC239-MRSA-[III+Cd+ccrC] (sasX-negat.), "Middle Eastern Cluster"	POS	POS	NEG	NEG	NEG	POS	POS	POS	POS	POS	NEG	POS	POS
JAEOUV Staphylococcus aureus strain AA13	CC239-MRSA-[III+Cd+ccrC] (sasX-negat.), "Middle Eastern Cluster"	POS	POS	NEG	NEG	NEG	POS	POS	POS	POS	POS	NEG	POS	POS
JAEOUW Staphylococcus aureus strain AA14	CC239-MRSA-[III+Cd+ccrC] (sasX-negat.), "Middle Eastern Cluster"	POS	POS	NEG	NEG	NEG	POS	POS	POS	POS	POS	NEG	POS	POS
JAEOVA Staphylococcus aureus strain AA22	CC239-MRSA-[III+Cd+ccrC] (sasX-negat.), "Middle Eastern Cluster"	POS	POS	NEG	NEG	NEG	POS	POS	POS	POS	POS	NEG	POS	POS
JAEOVB Staphylococcus aureus strain AA23	CC239-MRSA-[III+Cd+ccrC] (sasX-negat.), "Middle Eastern Cluster"	POS	POS	NEG	NEG	NEG	POS	POS	POS	POS	POS	NEG	POS	POS
JAEOVC Staphylococcus aureus strain AA27	CC239-MRSA-[III+Cd+ccrC] (sasX-negat.), "Middle Eastern Cluster"	POS	POS	NEG	NEG	NEG	POS	POS	POS	POS	POS	NEG	POS	POS
JAEOVD Staphylococcus aureus strain AA29	CC239-MRSA-[III+Cd+ccrC] (sasX-negat.), "Middle Eastern Cluster"	POS	POS	NEG	NEG	NEG	POS	POS	POS	POS	POS	NEG	POS	POS
JAEOVG Staphylococcus aureus strain AA31	CC239-MRSA-[III+Cd+ccrC] (sasX-negat.), "Middle Eastern Cluster"	POS	POS	NEG	NEG	NEG	POS	POS	POS	POS	POS	NEG	POS	POS
JAEOVP Staphylococcus aureus strain AA46	CC239-MRSA-[III+Cd+ccrC] (sasX-negat.), "Middle Eastern Cluster"	POS	POS	NEG	NEG	NEG	POS	POS	POS	POS	POS	NEG	POS	POS
JAEOVS Staphylococcus aureus strain AA52	CC239-MRSA-[III+Cd+ccrC] (sasX-negat.), "Middle Eastern Cluster"	POS	POS	NEG	NEG	NEG	POS	POS	POS	POS	POS	NEG	POS	POS
JAEOVU Staphylococcus aureus strain AA55	CC239-MRSA-[III+Cd+ccrC] (sasX-negat.), "Middle Eastern Cluster"	POS	POS	NEG	NEG	NEG	POS	POS	POS	POS	POS	NEG	POS	POS
JAEOVV Staphylococcus aureus strain AA57	CC239-MRSA-[III+Cd+ccrC] (sasX-negat.), "Middle Eastern Cluster"	POS	POS	NEG	NEG	NEG	POS	POS	POS	POS	POS	NEG	POS	POS
JAEOVY Staphylococcus aureus strain AA60	CC239-MRSA-[III+Cd+ccrC] (sasX-negat.), "Middle Eastern Cluster"	POS	POS	NEG	NEG	NEG	POS	POS	POS	POS	POS	NEG	POS	POS
JAEOVZ Staphylococcus aureus strain AA61	CC239-MRSA-[III+Cd+ccrC] (sasX-negat.), "Middle Eastern Cluster"	POS	POS	NEG	NEG	NEG	POS	POS	POS	POS	POS	NEG	POS	POS
JAEOWA Staphylococcus aureus strain AA62	CC239-MRSA-[III+Cd+ccrC] (sasX-negat.), "Middle Eastern Cluster"	POS	POS	NEG	NEG	NEG	POS	POS	POS	POS	POS	NEG	POS	POS
JAEOWB Staphylococcus aureus strain AA63	CC239-MRSA-[III+Cd+ccrC] (sasX-negat.), "Middle Eastern Cluster"	POS	POS	NEG	NEG	NEG	POS	POS	POS	POS	POS	NEG	POS	POS
JAEOWL Staphylococcus aureus strain AA79	CC239-MRSA-[III+Cd+ccrC] (sasX-negat.), "Middle Eastern Cluster"	POS	POS	NEG	NEG	NEG	POS	POS	POS	POS	POS	NEG	POS	POS
JAEOWQ Staphylococcus aureus strain AA92	CC239-MRSA-[III+Cd+ccrC] (sasX-negat.), "Middle Eastern Cluster"	POS	POS	NEG	NEG	NEG	POS	POS	POS	POS	POS	NEG	POS	POS
JAEOWC Staphylococcus aureus strain AA64	CC239-MRSA-[mec III+Cd], sasX-negative	POS	POS	NEG	NEG	NEG	POS	POS	POS	POS	POS	NEG	POS	POS
JAEOWP Staphylococcus aureus strain AA91	CC239-MRSA-[mec III+Cd], sasX-negative	POS	POS	NEG	NEG	NEG	POS	POS	POS	POS	POS	NEG	POS	POS

Isolate	Strain assignment	ADHAESION FACTORS / MSCRAMM GENES													
		efb		fnbA						fnbB					
		efb / fib	efb / fib (MRSA 252)	fnbA	fnbA (cons)	fnbA (COL)	fnbA (MRSA 252)	fnbA (Mu50+ MW2)	fnbA (RF122)	fnbB	fnbB (COL)	fnbB (COL+ Mu50+ MW2)	fnbB (Mu50)	fnbB (MW2)	fnbB (ST15)
		fibrinogen binding protein (19 kDa)		fibronectin-binding protein A						Fibronectin-binding protein B					
**CC1**															
Alexandria University_2020-17	CC1-MRSA-[V+fus+tir]	POS	NEG	POS	POS	NEG	NEG	POS	NEG	POS	NEG	POS	NEG	AMB	NEG
Alexandria University_2020-03	CC1-MRSA-[V+fus+tir+ccrAB1]	POS	NEG	POS	POS	NEG	NEG	POS	NEG	POS	NEG	POS	NEG	AMB	NEG
Alexandria University_2020-19	CC1-MRSA-[V+fus+tir+ccrAB1]	POS	NEG	POS	POS	NEG	NEG	POS	NEG	POS	NEG	POS	NEG	AMB	NEG
Alexandria University_2020-21	CC1-MRSA-[V+fus+tir+ccrAB1]	POS	NEG	POS	POS	NEG	NEG	POS	NEG	POS	NEG	POS	NEG	AMB	NEG
Alexandria University_2020-09	CC1-MRSA-[V+fus+tir+ccrAB1] (PVL+)	POS	NEG	POS	POS	NEG	NEG	POS	NEG	POS	NEG	POS	NEG	AMB	NEG
Alexandria University_2020-14	CC1-MRSA-[V+fus+tir+ccrAB1] (PVL+)	POS	NEG	POS	POS	NEG	NEG	POS	NEG	POS	NEG	POS	NEG	AMB	NEG
Alexandria University_2020-24	CC1-MRSA-[V+fus+tir+ccrAB1] (PVL+)	POS	NEG	POS	POS	NEG	NEG	POS	NEG	POS	NEG	POS	NEG	AMB	NEG
Alexandria University_2020-27	CC1-MRSA-[V+fus+tir+ccrAB1] (PVL+)	POS	NEG	POS	POS	NEG	NEG	POS	NEG	POS	NEG	POS	NEG	AMB	NEG
Alexandria University_2020-28	CC1-MRSA-[V+fus+tir+ccrAB1] (PVL+)	POS	NEG	POS	POS	NEG	NEG	AMB	NEG	POS	NEG	POS	NEG	AMB	NEG
**CC5**															
Alexandria University_2020-12	CC5-MRSA-[VI+fus+tir]	POS	NEG	POS	POS	NEG	NEG	POS	NEG	POS	NEG	POS	AMB	NEG	AMB
Alexandria University_2020-20	CC5-MRSA-[VI+fus+tir]	POS	NEG	POS	POS	NEG	NEG	POS	NEG	POS	NEG	POS	AMB	NEG	NEG
**CC6**															
Alexandria University_2020-29	CC6-MRSA-[V+fus]	POS	NEG	POS	POS	NEG	POS	NEG	NEG	POS	NEG	POS	AMB	NEG	NEG
**CC15**															
Alexandria University_2020-01	CC15-MRSA-[V+fus]	POS	NEG	POS	POS	POS	NEG	NEG	NEG	POS	NEG	POS	AMB	NEG	AMB
Alexandria University_2020-02	CC15-MRSA-[V+fus]	POS	NEG	POS	POS	POS	NEG	NEG	NEG	POS	NEG	POS	AMB	NEG	AMB
Alexandria University_2020-05	CC15-MRSA-[V+fus]	POS	NEG	POS	POS	POS	NEG	NEG	NEG	POS	NEG	POS	AMB	NEG	AMB
Alexandria University_2020-15	CC15-MRSA-[V+fus]	POS	NEG	POS	POS	POS	NEG	NEG	NEG	POS	NEG	POS	AMB	NEG	AMB
Alexandria University_2020-22	CC15-MRSA-[V+fus]	POS	NEG	POS	POS	POS	NEG	NEG	NEG	POS	NEG	POS	AMB	NEG	AMB
Alexandria University_2020-32	CC15-MRSA-[V+fus]	POS	NEG	POS	POS	POS	NEG	NEG	NEG	POS	NEG	POS	AMB	NEG	AMB
**CC22**															
Alexandria University_2020-04	CC22-MRSA-IVa (dcs negatives) (tst1+), "Gaza Epidemic Strain"	NEG	POS	POS	POS	NEG	NEG	POS	NEG	NEG	NEG	NEG	NEG	NEG	NEG
Alexandria University_2020-11	CC22-MRSA-IVa (dcs negatives) (tst1+), "Gaza Epidemic Strain"	NEG	POS	POS	POS	NEG	NEG	POS	NEG	NEG	NEG	NEG	NEG	NEG	NEG
**CC30**															
Alexandria University_2020-26	CC30-MRSA-IVa (PVL+), "WSPP/Southwest Pacific Clone"	NEG	POS	POS	POS	NEG	POS	NEG	NEG	POS	NEG	POS	AMB	NEG	NEG
**CC97**															
Alexandria University_2020-30	CC97-MRSA-[V+fus]	POS	NEG	POS	POS	NEG	NEG	NEG	NEG	POS	NEG	POS	AMB	NEG	NEG
**CC121**															
Alexandria University_2020-06	CC121-MRSA-[V+fus] (PVL+)	POS	NEG	POS	POS	NEG	NEG	NEG	NEG	POS	NEG	POS	NEG	NEG	NEG
**CC152**															
Alexandria University_2020-18	CC152-MRSA-[V+fus]	NEG	NEG	POS	POS	NEG	NEG	NEG	NEG	POS	NEG	POS	AMB	NEG	NEG
Alexandria University_2020-31	CC152-MRSA-[V+fus]	NEG	NEG	POS	POS	NEG	NEG	NEG	NEG	POS	NEG	POS	AMB	NEG	NEG
**CC239**															
Alexandria University_2020-13	CC239-MRSA-[III+Cd+ccrC] (sasX-negat.), "Middle Eastern Cluster"	POS	NEG	POS	POS	POS	NEG	NEG	NEG	POS	POS	AMB	NEG	NEG	NEG
Alexandria University_2020-16	CC239-MRSA-[III+Cd], sasX-negative	POS	NEG	POS	POS	POS	NEG	NEG	NEG	POS	AMB	POS	NEG	NEG	NEG
**CC1153**															
Alexandria University_2020-07	CC1153-MRSA-[V+fus] (PVL+)	POS	NEG	POS	POS	NEG	NEG	NEG	NEG	POS	NEG	POS	NEG	NEG	NEG
Alexandria University_2020-08	CC1153-MRSA-[V+fus] (PVL+)	POS	NEG	POS	POS	NEG	NEG	NEG	NEG	POS	NEG	POS	NEG	NEG	NEG
Alexandria University_2020-23	CC1153-MRSA-[V+fus] (PVL+)	POS	NEG	POS	POS	NEG	NEG	NEG	NEG	POS	NEG	POS	NEG	NEG	NEG
**Simulated hybridisations for sequences from Montelongo et al., 2022**															
**CC1**															
JAEOUR Staphylococcus aureus strain AA1	CC1-MRSA-[V+fus+tir+ccrAB1]	POS	NEG	POS	POS	NEG	NEG	POS	NEG	POS	NEG	AMB	NEG	AMB	NEG
JAEOWK Staphylococcus aureus strain AA78	CC1-MRSA-[V+fus+tir+ccrAB1]	POS	NEG	POS	POS	NEG	NEG	POS	NEG	POS	NEG	AMB	NEG	AMB	NEG
JAEOVR Staphylococcus aureus strain AA51	CC1-MRSA-[V+fus+tir+ccrAB1] (PVL+)	POS	NEG	POS	POS	NEG	NEG	POS	NEG	POS	NEG	AMB	NEG	AMB	NEG
JAEOWE Staphylococcus aureus strain AA67	CC1-MRSA-[V+fus+tir+ccrAB1] (PVL+)	POS	NEG	POS	POS	NEG	NEG	POS	NEG	POS	NEG	AMB	NEG	AMB	NEG
JAEOWG Staphylococcus aureus strain AA69	CC1-MRSA-[V+fus+tir+ccrAB1] (PVL+)	POS	NEG	POS	POS	NEG	NEG	POS	NEG	POS	NEG	AMB	NEG	AMB	NEG
JAEOWJ Staphylococcus aureus strain AA77	CC1-MRSA-[V+fus+tir+ccrAB1] (PVL+)	POS	NEG	POS	POS	NEG	NEG	POS	NEG	POS	NEG	AMB	NEG	AMB	NEG
**CC5**															
JAEOWI Staphylococcus aureus strain AA76	CC5-MRSA-[V+cas], WA MRSA-123	POS	NEG	POS	POS	NEG	NEG	POS	NEG	POS	NEG	AMB	AMB	NEG	NEG
JAEOWN Staphylococcus aureus strain AA80	CC5-MRSA-[V+cas], WA MRSA-123	POS	NEG	POS	POS	NEG	NEG	POS	NEG	POS	NEG	AMB	AMB	NEG	NEG
JAEOWH Staphylococcus aureus strain AA70	CC5-MRSA-[VI+fus+tir+, dcs-]	POS	NEG	POS	POS	NEG	NEG	POS	NEG	POS	NEG	AMB	AMB	NEG	NEG
**CC6**															
JAEOVF Staphylococcus aureus strain AA30	CC6-MRSA-IVa, WA MRSA-51	POS	NEG	POS	POS	NEG	POS	NEG	NEG	POS	NEG	AMB	AMB	NEG	NEG
**CC80**															
JAEOVM Staphylococcus aureus strain AA4	CC80-MRSA-IVc (PVL-) [aphA3/sat+]:[far1+], contaminated	POS	NEG	POS	POS	NEG	NEG	NEG	NEG	POS	NEG	AMB	NEG	POS	NEG
JAEOUZ Staphylococcus aureus strain AA2	CC80-MRSA-IVc (PVL+) [aphA3/sat+]:[far1+]	POS	NEG	POS	POS	NEG	NEG	NEG	NEG	POS	NEG	AMB	NEG	POS	NEG
JAEOVE Staphylococcus aureus strain AA3	CC80-MRSA-IVc (PVL+) [aphA3/sat+]:[far1+]	POS	NEG	POS	POS	NEG	NEG	NEG	NEG	POS	NEG	AMB	NEG	POS	NEG
JAEOVO Staphylococcus aureus strain AA45	CC80-MRSA-IVc (PVL+) [aphA3/sat+]:[far1+]	POS	NEG	POS	POS	NEG	NEG	NEG	NEG	POS	NEG	AMB	NEG	POS	NEG
**CC88**															
JAEOVT Staphylococcus aureus strain AA53	CC88-MRSA-IV, contaminated	POS	NEG	POS	POS	NEG	NEG	NEG	NEG	POS	NEG	POS	AMB	NEG	NEG
**CC97**															
JAEOVL Staphylococcus aureus strain AA39	CC97-MRSA-IVc, WA MRSA-54/63	POS	NEG	POS	POS	NEG	NEG	NEG	NEG	POS	NEG	POS	AMB	NEG	NEG
JAEOVX Staphylococcus aureus strain AA6	CC97-MRSA-IVc, WA MRSA-54/63	POS	NEG	POS	POS	NEG	NEG	NEG	NEG	POS	NEG	POS	AMB	NEG	NEG
JAEOWM Staphylococcus aureus strain AA8	CC97-MRSA-IVc, WA MRSA-54/63	POS	NEG	POS	POS	NEG	NEG	NEG	NEG	POS	NEG	POS	AMB	NEG	NEG
JAEOUU Staphylococcus aureus strain AA104	CC97-MRSA-V	POS	NEG	POS	POS	NEG	NEG	NEG	NEG	POS	NEG	POS	AMB	NEG	NEG
JAEOVJ Staphylococcus aureus strain AA35	CC97-MRSA-[V+fus]	POS	NEG	POS	POS	NEG	NEG	NEG	NEG	POS	NEG	POS	AMB	NEG	NEG
JAEOVK Staphylococcus aureus strain AA36	CC97-MRSA-[V+fus]	POS	NEG	POS	POS	NEG	NEG	NEG	NEG	POS	NEG	POS	AMB	NEG	NEG
**CC22**															
JAEOUY Staphylococcus aureus strain AA18	CC22-MRSA-IVa (dcs negatives) (tst1+), "Gaza Epidemic Strain"	NEG	AMB	POS	POS	NEG	NEG	POS	NEG	NEG	NEG	NEG	NEG	NEG	NEG
JAEOVH Staphylococcus aureus strain AA32	CC22-MRSA-IVa (dcs negatives) (tst1+), "Gaza Epidemic Strain"	NEG	AMB	POS	POS	NEG	NEG	POS	NEG	NEG	NEG	NEG	NEG	NEG	NEG
JAEOVQ Staphylococcus aureus strain AA5	CC22-MRSA-IVa (dcs negatives) (tst1+), "Gaza Epidemic Strain"	NEG	AMB	POS	POS	NEG	NEG	POS	NEG	NEG	NEG	NEG	NEG	NEG	NEG
**CC152**															
JAEOUX Staphylococcus aureus strain AA17	CC152-MRSA-[V+fus]	NEG	NEG	NEG	AMB	NEG	NEG	NEG	NEG	POS	NEG	AMB	AMB	NEG	NEG
**CC239**															
JAEOVI Staphylococcus aureus strain AA33	CC239-MRSA-[III+Cd/Hg+ccrC] (sasX-positive), "Southeast Asian Clade"	POS	NEG	POS	POS	POS	NEG	NEG	NEG	POS	AMB	AMB	NEG	NEG	NEG
JAEOUS Staphylococcus aureus strain AA101	CC239-MRSA-[III+Cd+ccrC] (sasX-negat.), "Middle Eastern Cluster"	POS	NEG	POS	POS	POS	NEG	NEG	NEG	POS	AMB	AMB	NEG	NEG	NEG
JAEOUV Staphylococcus aureus strain AA13	CC239-MRSA-[III+Cd+ccrC] (sasX-negat.), "Middle Eastern Cluster"	POS	NEG	POS	POS	POS	NEG	NEG	NEG	POS	AMB	AMB	NEG	NEG	NEG
JAEOUW Staphylococcus aureus strain AA14	CC239-MRSA-[III+Cd+ccrC] (sasX-negat.), "Middle Eastern Cluster"	POS	NEG	POS	POS	POS	NEG	NEG	NEG	POS	AMB	AMB	NEG	NEG	NEG
JAEOVA Staphylococcus aureus strain AA22	CC239-MRSA-[III+Cd+ccrC] (sasX-negat.), "Middle Eastern Cluster"	POS	NEG	POS	POS	POS	NEG	NEG	NEG	POS	AMB	AMB	NEG	NEG	NEG
JAEOVB Staphylococcus aureus strain AA23	CC239-MRSA-[III+Cd+ccrC] (sasX-negat.), "Middle Eastern Cluster"	POS	NEG	POS	POS	POS	NEG	NEG	NEG	POS	AMB	AMB	NEG	NEG	NEG
JAEOVC Staphylococcus aureus strain AA27	CC239-MRSA-[III+Cd+ccrC] (sasX-negat.), "Middle Eastern Cluster"	POS	NEG	POS	POS	POS	NEG	NEG	NEG	POS	AMB	AMB	NEG	NEG	NEG
JAEOVD Staphylococcus aureus strain AA29	CC239-MRSA-[III+Cd+ccrC] (sasX-negat.), "Middle Eastern Cluster"	POS	NEG	POS	POS	POS	NEG	NEG	NEG	POS	AMB	AMB	NEG	NEG	NEG
JAEOVG Staphylococcus aureus strain AA31	CC239-MRSA-[III+Cd+ccrC] (sasX-negat.), "Middle Eastern Cluster"	POS	NEG	POS	POS	POS	NEG	NEG	NEG	POS	AMB	AMB	NEG	NEG	NEG
JAEOVP Staphylococcus aureus strain AA46	CC239-MRSA-[III+Cd+ccrC] (sasX-negat.), "Middle Eastern Cluster"	POS	NEG	POS	POS	POS	NEG	NEG	NEG	POS	AMB	AMB	NEG	NEG	NEG
JAEOVS Staphylococcus aureus strain AA52	CC239-MRSA-[III+Cd+ccrC] (sasX-negat.), "Middle Eastern Cluster"	POS	NEG	POS	POS	POS	NEG	NEG	NEG	POS	AMB	AMB	NEG	NEG	NEG
JAEOVU Staphylococcus aureus strain AA55	CC239-MRSA-[III+Cd+ccrC] (sasX-negat.), "Middle Eastern Cluster"	POS	NEG	POS	POS	POS	NEG	NEG	NEG	POS	AMB	AMB	NEG	NEG	NEG
JAEOVV Staphylococcus aureus strain AA57	CC239-MRSA-[III+Cd+ccrC] (sasX-negat.), "Middle Eastern Cluster"	POS	NEG	POS	POS	POS	NEG	NEG	NEG	POS	AMB	AMB	NEG	NEG	NEG
JAEOVY Staphylococcus aureus strain AA60	CC239-MRSA-[III+Cd+ccrC] (sasX-negat.), "Middle Eastern Cluster"	POS	NEG	POS	POS	POS	NEG	NEG	NEG	POS	AMB	AMB	NEG	NEG	NEG
JAEOVZ Staphylococcus aureus strain AA61	CC239-MRSA-[III+Cd+ccrC] (sasX-negat.), "Middle Eastern Cluster"	POS	NEG	POS	POS	POS	NEG	NEG	NEG	POS	AMB	AMB	NEG	NEG	NEG
JAEOWA Staphylococcus aureus strain AA62	CC239-MRSA-[III+Cd+ccrC] (sasX-negat.), "Middle Eastern Cluster"	POS	NEG	POS	POS	POS	NEG	NEG	NEG	POS	AMB	AMB	NEG	NEG	NEG
JAEOWB Staphylococcus aureus strain AA63	CC239-MRSA-[III+Cd+ccrC] (sasX-negat.), "Middle Eastern Cluster"	POS	NEG	POS	POS	POS	NEG	NEG	NEG	POS	AMB	AMB	NEG	NEG	NEG
JAEOWL Staphylococcus aureus strain AA79	CC239-MRSA-[III+Cd+ccrC] (sasX-negat.), "Middle Eastern Cluster"	POS	NEG	POS	POS	POS	NEG	NEG	NEG	POS	AMB	AMB	NEG	NEG	NEG
JAEOWQ Staphylococcus aureus strain AA92	CC239-MRSA-[III+Cd+ccrC] (sasX-negat.), "Middle Eastern Cluster"	POS	NEG	POS	POS	POS	NEG	NEG	NEG	POS	AMB	AMB	NEG	NEG	NEG
JAEOWC Staphylococcus aureus strain AA64	CC239-MRSA-[mec III+Cd], sasX-negative	POS	NEG	POS	POS	POS	NEG	NEG	NEG	POS	AMB	AMB	NEG	NEG	NEG
JAEOWP Staphylococcus aureus strain AA91	CC239-MRSA-[mec III+Cd], sasX-negative	POS	NEG	POS	POS	POS	NEG	NEG	NEG	POS	AMB	AMB	NEG	NEG	NEG

Isolate	Strain assignment	ADHAESION FACTORS / MSCRAMM GENES														
		map				sasG				sasX	sdrC					
		map	map (COL)	map (MRSA252)	map (Mu50+MW2)	sasG	sasG (COL+Mu50)	sasG (MW2)	sasG (OtherThan 252+122	sasX / sesI	sdrC	sdrC (cons)	sdrC (COL)	sdrC (Mu50)	sdrC (MW2+MRSA252+RF122)	sdrC (OtherThan 252+RF122)
		Major histocompatibility complex class II analog protein (=Extracellular adherence protein, eap)				Staphylococcus aureus surface protein G					Ser-Asp rich fibrinogen-/bone sialoprotein-binding protein C					
**CC1**																
Alexandria University_2020-17	CC1-MRSA-[V+fus+tir]	POS	NEG	NEG	POS	POS	NEG	POS	POS	NEG	POS	POS	NEG	NEG	POS	POS
Alexandria University_2020-03	CC1-MRSA-[V+fus+tir+ccrAB1]	POS	NEG	NEG	POS	POS	NEG	POS	POS	NEG	POS	POS	NEG	NEG	POS	POS
Alexandria University_2020-19	CC1-MRSA-[V+fus+tir+ccrAB1]	POS	NEG	NEG	POS	POS	NEG	POS	POS	NEG	POS	POS	NEG	NEG	POS	POS
Alexandria University_2020-21	CC1-MRSA-[V+fus+tir+ccrAB1]	POS	NEG	NEG	POS	POS	NEG	POS	POS	NEG	POS	POS	NEG	NEG	POS	POS
Alexandria University_2020-09	CC1-MRSA-[V+fus+tir+ccrAB1] (PVL+)	POS	NEG	NEG	POS	POS	NEG	POS	POS	NEG	POS	POS	NEG	NEG	POS	POS
Alexandria University_2020-14	CC1-MRSA-[V+fus+tir+ccrAB1] (PVL+)	POS	NEG	NEG	POS	POS	NEG	POS	POS	NEG	POS	POS	NEG	NEG	POS	POS
Alexandria University_2020-24	CC1-MRSA-[V+fus+tir+ccrAB1] (PVL+)	POS	NEG	NEG	POS	POS	NEG	POS	POS	NEG	POS	POS	NEG	NEG	POS	POS
Alexandria University_2020-27	CC1-MRSA-[V+fus+tir+ccrAB1] (PVL+)	POS	NEG	NEG	POS	POS	NEG	POS	POS	NEG	POS	POS	NEG	NEG	POS	POS
Alexandria University_2020-28	CC1-MRSA-[V+fus+tir+ccrAB1] (PVL+)	POS	NEG	NEG	POS	POS	NEG	POS	POS	NEG	POS	POS	NEG	NEG	POS	POS
**CC5**																
Alexandria University_2020-12	CC5-MRSA-[VI+fus+tir]	POS	POS	NEG	POS	POS	POS	NEG	POS	NEG	POS	POS	NEG	POS	NEG	POS
Alexandria University_2020-20	CC5-MRSA-[VI+fus+tir]	POS	NEG	NEG	POS	POS	POS	NEG	POS	NEG	POS	POS	NEG	POS	NEG	POS
**CC6**																
Alexandria University_2020-29	CC6-MRSA-[V+fus]	POS	NEG	NEG	POS	POS	NEG	POS	POS	NEG	POS	POS	POS	NEG	NEG	POS
**CC15**																
Alexandria University_2020-01	CC15-MRSA-[V+fus]	POS	POS	NEG	POS	POS	NEG	POS	POS	NEG	POS	POS	POS	NEG	NEG	POS
Alexandria University_2020-02	CC15-MRSA-[V+fus]	POS	POS	NEG	POS	POS	NEG	POS	POS	NEG	POS	POS	POS	NEG	NEG	POS
Alexandria University_2020-05	CC15-MRSA-[V+fus]	POS	POS	NEG	POS	POS	NEG	POS	POS	NEG	POS	POS	POS	NEG	NEG	POS
Alexandria University_2020-15	CC15-MRSA-[V+fus]	POS	POS	NEG	POS	POS	NEG	POS	POS	NEG	POS	POS	POS	NEG	NEG	POS
Alexandria University_2020-22	CC15-MRSA-[V+fus]	POS	AMB	NEG	POS	POS	NEG	POS	POS	NEG	POS	POS	POS	NEG	NEG	POS
Alexandria University_2020-32	CC15-MRSA-[V+fus]	POS	POS	NEG	POS	POS	NEG	POS	POS	NEG	POS	POS	POS	NEG	NEG	POS
**CC22**																
Alexandria University_2020-04	CC22-MRSA-IVa (dcs negatives) (tst1+), "Gaza Epidemic Strain"	NEG	NEG	NEG	AMB	POS	NEG	POS	POS	NEG	POS	POS	NEG	POS	NEG	POS
Alexandria University_2020-11	CC22-MRSA-IVa (dcs negatives) (tst1+), "Gaza Epidemic Strain"	POS	NEG	NEG	POS	POS	NEG	POS	POS	NEG	POS	POS	NEG	POS	NEG	POS
**CC30**																
Alexandria University_2020-26	CC30-MRSA-IVa (PVL+), "WSPP/Southwest Pacific Clone"	POS	NEG	POS	NEG	NEG	NEG	NEG	NEG	NEG	POS	POS	NEG	NEG	NEG	NEG
**CC97**																
Alexandria University_2020-30	CC97-MRSA-[V+fus]	POS	POS	NEG	POS	POS	POS	NEG	POS	NEG	POS	POS	NEG	POS	POS	POS
**CC121**																
Alexandria University_2020-06	CC121-MRSA-[V+fus] (PVL+)	POS	POS	NEG	POS	NEG	NEG	NEG	NEG	NEG	POS	POS	POS	NEG	NEG	NEG
**CC152**																
Alexandria University_2020-18	CC152-MRSA-[V+fus]	NEG	NEG	NEG	NEG	NEG	NEG	NEG	NEG	NEG	NEG	NEG	NEG	NEG	NEG	NEG
Alexandria University_2020-31	CC152-MRSA-[V+fus]	NEG	NEG	NEG	NEG	NEG	NEG	NEG	NEG	NEG	NEG	NEG	NEG	NEG	NEG	NEG
**CC239**																
Alexandria University_2020-13	CC239-MRSA-[III+Cd+ccrC] (sasX-negat.), "Middle Eastern Cluster"	POS	POS	NEG	NEG	POS	POS	NEG	POS	NEG	POS	POS	POS	NEG	NEG	POS
Alexandria University_2020-16	CC239-MRSA-[III+Cd], sasX-negative	POS	POS	NEG	NEG	POS	POS	NEG	POS	NEG	POS	POS	POS	NEG	NEG	POS
**CC1153**																
Alexandria University_2020-07	CC1153-MRSA-[V+fus] (PVL+)	POS	NEG	NEG	POS	POS	NEG	POS	POS	NEG	POS	POS	AMB	NEG	NEG	POS
Alexandria University_2020-08	CC1153-MRSA-[V+fus] (PVL+)	POS	NEG	NEG	POS	POS	NEG	POS	POS	NEG	POS	POS	POS	NEG	NEG	POS
Alexandria University_2020-23	CC1153-MRSA-[V+fus] (PVL+)	POS	NEG	NEG	POS	POS	NEG	POS	POS	NEG	POS	POS	POS	NEG	NEG	POS
**Simulated hybridisations for sequences from Montelongo et al., 2022**																
**CC1**																
JAEOUR Staphylococcus aureus strain AA1	CC1-MRSA-[V+fus+tir+ccrAB1]	POS	AMB	NEG	POS	POS	NEG	POS	POS	NEG	POS	POS	NEG	NEG	POS	POS
JAEOWK Staphylococcus aureus strain AA78	CC1-MRSA-[V+fus+tir+ccrAB1]	NEG	AMB	NEG	NEG	POS	NEG	POS	POS	NEG	POS	POS	NEG	NEG	POS	POS
JAEOVR Staphylococcus aureus strain AA51	CC1-MRSA-[V+fus+tir+ccrAB1] (PVL+)	POS	AMB	NEG	POS	POS	NEG	POS	POS	NEG	POS	POS	NEG	NEG	POS	POS
JAEOWE Staphylococcus aureus strain AA67	CC1-MRSA-[V+fus+tir+ccrAB1] (PVL+)	POS	AMB	NEG	POS	POS	NEG	POS	POS	NEG	POS	POS	NEG	NEG	POS	POS
JAEOWG Staphylococcus aureus strain AA69	CC1-MRSA-[V+fus+tir+ccrAB1] (PVL+)	POS	AMB	NEG	POS	POS	NEG	POS	POS	NEG	POS	POS	NEG	NEG	POS	POS
JAEOWJ Staphylococcus aureus strain AA77	CC1-MRSA-[V+fus+tir+ccrAB1] (PVL+)	POS	AMB	NEG	POS	POS	NEG	POS	POS	NEG	POS	POS	NEG	NEG	POS	POS
**CC5**																
JAEOWI Staphylococcus aureus strain AA76	CC5-MRSA-[V+cas], WA MRSA-123	POS	AMB	NEG	POS	POS	POS	NEG	POS	NEG	POS	POS	NEG	POS	NEG	POS
JAEOWN Staphylococcus aureus strain AA80	CC5-MRSA-[V+cas], WA MRSA-123	POS	AMB	NEG	POS	POS	POS	NEG	POS	NEG	POS	POS	NEG	POS	NEG	POS
JAEOWH Staphylococcus aureus strain AA70	CC5-MRSA-[VI+fus+tir+, dcs-]	POS	AMB	NEG	POS	POS	POS	NEG	POS	NEG	POS	POS	NEG	POS	NEG	POS
**CC6**																
JAEOVF Staphylococcus aureus strain AA30	CC6-MRSA-IVa, WA MRSA-51	POS	AMB	NEG	POS	POS	NEG	POS	POS	NEG	POS	POS	POS	NEG	NEG	POS
**CC80**																
JAEOVM Staphylococcus aureus strain AA4	CC80-MRSA-IVc (PVL-) [aphA3/sat+]:[far1+], contaminated	POS	AMB	NEG	POS	POS	POS	POS	POS	NEG	POS	POS	POS	NEG	NEG	POS
JAEOUZ Staphylococcus aureus strain AA2	CC80-MRSA-IVc (PVL+) [aphA3/sat+]:[far1+]	POS	AMB	NEG	POS	POS	NEG	POS	POS	NEG	POS	POS	POS	NEG	NEG	POS
JAEOVE Staphylococcus aureus strain AA3	CC80-MRSA-IVc (PVL+) [aphA3/sat+]:[far1+]	POS	AMB	NEG	POS	POS	NEG	POS	POS	NEG	POS	POS	POS	NEG	NEG	POS
JAEOVO Staphylococcus aureus strain AA45	CC80-MRSA-IVc (PVL+) [aphA3/sat+]:[far1+]	POS	AMB	NEG	POS	POS	NEG	POS	POS	NEG	POS	POS	POS	NEG	NEG	POS
**CC88**																
JAEOVT Staphylococcus aureus strain AA53	CC88-MRSA-IV, contaminated	POS	AMB	NEG	POS	POS	POS	POS	POS	NEG	POS	AMB	NEG	AMB	NEG	POS
**CC97**																
JAEOVL Staphylococcus aureus strain AA39	CC97-MRSA-IVc, WA MRSA-54/63	POS	AMB	NEG	POS	POS	POS	NEG	POS	NEG	POS	AMB	NEG	AMB	NEG	POS
JAEOVX Staphylococcus aureus strain AA6	CC97-MRSA-IVc, WA MRSA-54/63	POS	AMB	NEG	POS	POS	POS	NEG	POS	NEG	POS	AMB	NEG	AMB	NEG	POS
JAEOWM Staphylococcus aureus strain AA8	CC97-MRSA-IVc, WA MRSA-54/63	POS	AMB	NEG	POS	POS	POS	NEG	POS	NEG	POS	AMB	NEG	AMB	NEG	POS
JAEOUU Staphylococcus aureus strain AA104	CC97-MRSA-V	POS	AMB	NEG	POS	POS	POS	NEG	POS	NEG	POS	AMB	NEG	AMB	NEG	POS
JAEOVJ Staphylococcus aureus strain AA35	CC97-MRSA-[V+fus]	POS	AMB	NEG	POS	POS	POS	NEG	POS	NEG	POS	AMB	NEG	AMB	NEG	POS
JAEOVK Staphylococcus aureus strain AA36	CC97-MRSA-[V+fus]	POS	AMB	NEG	POS	POS	POS	NEG	POS	NEG	POS	AMB	NEG	AMB	NEG	POS
**CC22**																
JAEOUY Staphylococcus aureus strain AA18	CC22-MRSA-IVa (dcs negatives) (tst1+), "Gaza Epidemic Strain"	POS	NEG	NEG	POS	POS	NEG	POS	POS	NEG	POS	POS	NEG	POS	NEG	POS
JAEOVH Staphylococcus aureus strain AA32	CC22-MRSA-IVa (dcs negatives) (tst1+), "Gaza Epidemic Strain"	POS	NEG	NEG	POS	POS	NEG	POS	POS	NEG	POS	POS	NEG	POS	NEG	POS
JAEOVQ Staphylococcus aureus strain AA5	CC22-MRSA-IVa (dcs negatives) (tst1+), "Gaza Epidemic Strain"	POS	NEG	NEG	POS	POS	NEG	POS	POS	NEG	POS	POS	NEG	POS	NEG	POS
**CC152**																
JAEOUX Staphylococcus aureus strain AA17	CC152-MRSA-[V+fus]	NEG	NEG	NEG	NEG	NEG	NEG	NEG	NEG	NEG	NEG	NEG	NEG	NEG	NEG	NEG
**CC239**																
JAEOVI Staphylococcus aureus strain AA33	CC239-MRSA-[III+Cd/Hg+ccrC] (sasX-positive), "Southeast Asian Clade"	POS	POS	NEG	NEG	POS	POS	NEG	POS	POS	POS	POS	POS	NEG	NEG	POS
JAEOUS Staphylococcus aureus strain AA101	CC239-MRSA-[III+Cd+ccrC] (sasX-negat.), "Middle Eastern Cluster"	POS	POS	NEG	NEG	POS	POS	NEG	POS	NEG	POS	POS	POS	NEG	NEG	POS
JAEOUV Staphylococcus aureus strain AA13	CC239-MRSA-[III+Cd+ccrC] (sasX-negat.), "Middle Eastern Cluster"	POS	POS	NEG	NEG	POS	POS	NEG	POS	NEG	POS	POS	POS	NEG	NEG	POS
JAEOUW Staphylococcus aureus strain AA14	CC239-MRSA-[III+Cd+ccrC] (sasX-negat.), "Middle Eastern Cluster"	POS	POS	NEG	NEG	POS	POS	NEG	POS	NEG	POS	POS	POS	NEG	NEG	POS
JAEOVA Staphylococcus aureus strain AA22	CC239-MRSA-[III+Cd+ccrC] (sasX-negat.), "Middle Eastern Cluster"	POS	POS	NEG	NEG	POS	POS	NEG	POS	NEG	POS	POS	POS	NEG	NEG	POS
JAEOVB Staphylococcus aureus strain AA23	CC239-MRSA-[III+Cd+ccrC] (sasX-negat.), "Middle Eastern Cluster"	POS	POS	NEG	NEG	POS	POS	NEG	POS	NEG	POS	POS	POS	NEG	NEG	POS
JAEOVC Staphylococcus aureus strain AA27	CC239-MRSA-[III+Cd+ccrC] (sasX-negat.), "Middle Eastern Cluster"	POS	POS	NEG	NEG	POS	POS	NEG	POS	NEG	POS	POS	POS	NEG	NEG	POS
JAEOVD Staphylococcus aureus strain AA29	CC239-MRSA-[III+Cd+ccrC] (sasX-negat.), "Middle Eastern Cluster"	POS	POS	NEG	NEG	POS	POS	NEG	POS	NEG	POS	POS	POS	NEG	NEG	POS
JAEOVG Staphylococcus aureus strain AA31	CC239-MRSA-[III+Cd+ccrC] (sasX-negat.), "Middle Eastern Cluster"	POS	POS	NEG	NEG	POS	POS	NEG	POS	NEG	POS	POS	POS	NEG	NEG	POS
JAEOVP Staphylococcus aureus strain AA46	CC239-MRSA-[III+Cd+ccrC] (sasX-negat.), "Middle Eastern Cluster"	POS	POS	NEG	NEG	POS	POS	NEG	POS	NEG	POS	POS	POS	NEG	NEG	POS
JAEOVS Staphylococcus aureus strain AA52	CC239-MRSA-[III+Cd+ccrC] (sasX-negat.), "Middle Eastern Cluster"	POS	POS	NEG	NEG	POS	POS	NEG	POS	NEG	POS	POS	POS	NEG	NEG	POS
JAEOVU Staphylococcus aureus strain AA55	CC239-MRSA-[III+Cd+ccrC] (sasX-negat.), "Middle Eastern Cluster"	POS	POS	NEG	NEG	POS	POS	NEG	POS	NEG	POS	POS	POS	NEG	NEG	POS
JAEOVV Staphylococcus aureus strain AA57	CC239-MRSA-[III+Cd+ccrC] (sasX-negat.), "Middle Eastern Cluster"	POS	POS	NEG	NEG	POS	POS	NEG	POS	NEG	POS	POS	POS	NEG	NEG	POS
JAEOVY Staphylococcus aureus strain AA60	CC239-MRSA-[III+Cd+ccrC] (sasX-negat.), "Middle Eastern Cluster"	POS	POS	NEG	NEG	POS	POS	NEG	POS	NEG	POS	POS	POS	NEG	NEG	POS
JAEOVZ Staphylococcus aureus strain AA61	CC239-MRSA-[III+Cd+ccrC] (sasX-negat.), "Middle Eastern Cluster"	POS	POS	NEG	NEG	POS	POS	NEG	POS	NEG	POS	POS	POS	NEG	NEG	POS
JAEOWA Staphylococcus aureus strain AA62	CC239-MRSA-[III+Cd+ccrC] (sasX-negat.), "Middle Eastern Cluster"	POS	POS	NEG	NEG	POS	POS	NEG	POS	NEG	POS	POS	POS	NEG	NEG	POS
JAEOWB Staphylococcus aureus strain AA63	CC239-MRSA-[III+Cd+ccrC] (sasX-negat.), "Middle Eastern Cluster"	POS	POS	NEG	NEG	POS	POS	NEG	POS	NEG	POS	POS	POS	NEG	NEG	POS
JAEOWL Staphylococcus aureus strain AA79	CC239-MRSA-[III+Cd+ccrC] (sasX-negat.), "Middle Eastern Cluster"	POS	POS	NEG	NEG	POS	POS	NEG	POS	NEG	POS	POS	POS	NEG	NEG	POS
JAEOWQ Staphylococcus aureus strain AA92	CC239-MRSA-[III+Cd+ccrC] (sasX-negat.), "Middle Eastern Cluster"	POS	POS	NEG	NEG	POS	POS	NEG	POS	NEG	POS	POS	POS	NEG	NEG	POS
JAEOWC Staphylococcus aureus strain AA64	CC239-MRSA-[mec III+Cd], sasX-negative	POS	POS	NEG	NEG	POS	POS	NEG	POS	NEG	POS	POS	POS	NEG	NEG	POS
JAEOWP Staphylococcus aureus strain AA91	CC239-MRSA-[mec III+Cd], sasX-negative	POS	POS	NEG	NEG	POS	POS	NEG	POS	NEG	POS	POS	POS	NEG	NEG	POS

Isolate	Strain assignment	ADHAESION FACTORS / MSCRAMM GENES										
		sdrD					vwb					
		sdrD	sdrD (cons)	sdrD (COL+MW2)	sdrD (Mu50)	sdrD (other)	vwb	vwb (cons)	vwb (COL+MW2)	vwb (MRSA252)	vwb (Mu50)	vwb (RF122)
		Ser-Asp rich fibrinogen-/bone sialoprotein-binding protein D					van Willebrand factor binding protein					
**CC1**												
Alexandria University_2020-17	CC1-MRSA-[V+fus+tir]	POS	POS	NEG	NEG	NEG	POS	POS	POS	NEG	NEG	NEG
Alexandria University_2020-03	CC1-MRSA-[V+fus+tir+ccrAB1]	POS	POS	NEG	NEG	NEG	POS	POS	POS	NEG	NEG	NEG
Alexandria University_2020-19	CC1-MRSA-[V+fus+tir+ccrAB1]	POS	POS	NEG	NEG	NEG	POS	POS	POS	NEG	NEG	NEG
Alexandria University_2020-21	CC1-MRSA-[V+fus+tir+ccrAB1]	POS	POS	NEG	NEG	NEG	POS	POS	POS	NEG	NEG	NEG
Alexandria University_2020-09	CC1-MRSA-[V+fus+tir+ccrAB1] (PVL+)	POS	POS	AMB	NEG	NEG	POS	POS	POS	NEG	NEG	NEG
Alexandria University_2020-14	CC1-MRSA-[V+fus+tir+ccrAB1] (PVL+)	POS	POS	POS	NEG	NEG	POS	POS	POS	NEG	NEG	NEG
Alexandria University_2020-24	CC1-MRSA-[V+fus+tir+ccrAB1] (PVL+)	POS	POS	POS	NEG	NEG	POS	POS	POS	NEG	NEG	NEG
Alexandria University_2020-27	CC1-MRSA-[V+fus+tir+ccrAB1] (PVL+)	POS	POS	AMB	NEG	NEG	POS	POS	POS	NEG	NEG	NEG
Alexandria University_2020-28	CC1-MRSA-[V+fus+tir+ccrAB1] (PVL+)	POS	POS	NEG	NEG	NEG	POS	POS	POS	NEG	NEG	NEG
**CC5**												
Alexandria University_2020-12	CC5-MRSA-[VI+fus+tir]	POS	POS	NEG	POS	NEG	POS	POS	NEG	NEG	POS	NEG
Alexandria University_2020-20	CC5-MRSA-[VI+fus+tir]	POS	POS	NEG	POS	NEG	POS	POS	NEG	NEG	POS	NEG
**CC6**												
Alexandria University_2020-29	CC6-MRSA-[V+fus]	NEG	NEG	NEG	NEG	NEG	POS	POS	NEG	POS	NEG	NEG
**CC15**												
Alexandria University_2020-01	CC15-MRSA-[V+fus]	POS	POS	NEG	POS	NEG	POS	POS	NEG	NEG	NEG	NEG
Alexandria University_2020-02	CC15-MRSA-[V+fus]	POS	POS	NEG	POS	NEG	POS	POS	NEG	NEG	NEG	NEG
Alexandria University_2020-05	CC15-MRSA-[V+fus]	POS	POS	NEG	POS	NEG	POS	POS	NEG	NEG	NEG	NEG
Alexandria University_2020-15	CC15-MRSA-[V+fus]	POS	POS	NEG	POS	NEG	POS	POS	NEG	NEG	NEG	NEG
Alexandria University_2020-22	CC15-MRSA-[V+fus]	POS	POS	NEG	POS	NEG	POS	POS	NEG	NEG	NEG	NEG
Alexandria University_2020-32	CC15-MRSA-[V+fus]	POS	POS	NEG	POS	NEG	POS	POS	NEG	NEG	NEG	NEG
**CC22**												
Alexandria University_2020-04	CC22-MRSA-IVa (dcs negatives) (tst1+), "Gaza Epidemic Strain"	POS	POS	NEG	NEG	POS	POS	POS	NEG	NEG	NEG	POS
Alexandria University_2020-11	CC22-MRSA-IVa (dcs negatives) (tst1+), "Gaza Epidemic Strain"	POS	POS	NEG	NEG	POS	POS	POS	NEG	NEG	NEG	POS
**CC30**												
Alexandria University_2020-26	CC30-MRSA-IVa (PVL+), "WSPP/Southwest Pacific Clone"	POS	POS	NEG	NEG	POS	POS	POS	NEG	POS	NEG	NEG
**CC97**												
Alexandria University_2020-30	CC97-MRSA-[V+fus]	POS	POS	NEG	POS	NEG	POS	POS	NEG	NEG	NEG	POS
**CC121**												
Alexandria University_2020-06	CC121-MRSA-[V+fus] (PVL+)	POS	POS	NEG	NEG	NEG	POS	POS	POS	NEG	NEG	NEG
**CC152**												
Alexandria University_2020-18	CC152-MRSA-[V+fus]	POS	POS	POS	NEG	NEG	POS	POS	NEG	NEG	NEG	NEG
Alexandria University_2020-31	CC152-MRSA-[V+fus]	POS	POS	POS	NEG	NEG	POS	POS	NEG	NEG	NEG	NEG
**CC239**												
Alexandria University_2020-13	CC239-MRSA-[III+Cd+ccrC] (sasX-negat.), "Middle Eastern Cluster"	POS	POS	AMB	NEG	NEG	POS	POS	POS	NEG	NEG	NEG
Alexandria University_2020-16	CC239-MRSA-[III+Cd], sasX-negative	POS	POS	NEG	NEG	NEG	POS	POS	POS	NEG	NEG	NEG
**CC1153**												
Alexandria University_2020-07	CC1153-MRSA-[V+fus] (PVL+)	POS	POS	NEG	AMB	NEG	POS	POS	NEG	NEG	NEG	NEG
Alexandria University_2020-08	CC1153-MRSA-[V+fus] (PVL+)	POS	POS	NEG	POS	NEG	POS	POS	NEG	NEG	NEG	NEG
Alexandria University_2020-23	CC1153-MRSA-[V+fus] (PVL+)	POS	POS	NEG	POS	NEG	POS	POS	NEG	NEG	NEG	NEG
**Simulated hybridisations for sequences from Montelongo et al., 2022**												
**CC1**												
JAEOUR Staphylococcus aureus strain AA1	CC1-MRSA-[V+fus+tir+ccrAB1]	POS	POS	POS	NEG	NEG	POS	POS	POS	NEG	NEG	NEG
JAEOWK Staphylococcus aureus strain AA78	CC1-MRSA-[V+fus+tir+ccrAB1]	POS	POS	POS	NEG	NEG	POS	POS	POS	NEG	NEG	NEG
JAEOVR Staphylococcus aureus strain AA51	CC1-MRSA-[V+fus+tir+ccrAB1] (PVL+)	POS	POS	POS	NEG	NEG	POS	POS	POS	NEG	NEG	NEG
JAEOWE Staphylococcus aureus strain AA67	CC1-MRSA-[V+fus+tir+ccrAB1] (PVL+)	POS	POS	POS	NEG	NEG	POS	POS	POS	NEG	NEG	NEG
JAEOWG Staphylococcus aureus strain AA69	CC1-MRSA-[V+fus+tir+ccrAB1] (PVL+)	POS	POS	POS	NEG	NEG	POS	POS	POS	NEG	NEG	NEG
JAEOWJ Staphylococcus aureus strain AA77	CC1-MRSA-[V+fus+tir+ccrAB1] (PVL+)	POS	POS	POS	NEG	NEG	POS	POS	POS	NEG	NEG	NEG
**CC5**												
JAEOWI Staphylococcus aureus strain AA76	CC5-MRSA-[V+cas], WA MRSA-123	POS	POS	NEG	POS	NEG	POS	POS	NEG	NEG	POS	NEG
JAEOWN Staphylococcus aureus strain AA80	CC5-MRSA-[V+cas], WA MRSA-123	POS	POS	NEG	POS	NEG	POS	POS	NEG	NEG	POS	NEG
JAEOWH Staphylococcus aureus strain AA70	CC5-MRSA-[VI+fus+tir+, dcs-]	POS	POS	NEG	POS	NEG	POS	POS	NEG	NEG	POS	NEG
**CC6**												
JAEOVF Staphylococcus aureus strain AA30	CC6-MRSA-IVa, WA MRSA-51	POS	POS	NEG	POS	NEG	POS	POS	NEG	AMB	NEG	NEG
**CC80**												
JAEOVM Staphylococcus aureus strain AA4	CC80-MRSA-IVc (PVL-) [aphA3/sat+]:[far1+], contaminated	POS	POS	NEG	NEG	POS	POS	POS	NEG	AMB	NEG	NEG
JAEOUZ Staphylococcus aureus strain AA2	CC80-MRSA-IVc (PVL+) [aphA3/sat+]:[far1+]	POS	POS	NEG	NEG	POS	POS	POS	NEG	AMB	NEG	NEG
JAEOVE Staphylococcus aureus strain AA3	CC80-MRSA-IVc (PVL+) [aphA3/sat+]:[far1+]	POS	POS	NEG	NEG	POS	POS	POS	NEG	AMB	NEG	NEG
JAEOVO Staphylococcus aureus strain AA45	CC80-MRSA-IVc (PVL+) [aphA3/sat+]:[far1+]	POS	POS	NEG	NEG	POS	POS	POS	NEG	AMB	NEG	NEG
**CC88**												
JAEOVT Staphylococcus aureus strain AA53	CC88-MRSA-IV, contaminated	POS	POS	NEG	POS	POS	POS	POS	NEG	NEG	POS	NEG
**CC97**												
JAEOVL Staphylococcus aureus strain AA39	CC97-MRSA-IVc, WA MRSA-54/63	POS	POS	NEG	POS	NEG	POS	POS	NEG	NEG	NEG	POS
JAEOVX Staphylococcus aureus strain AA6	CC97-MRSA-IVc, WA MRSA-54/63	POS	POS	NEG	POS	NEG	POS	POS	NEG	NEG	NEG	POS
JAEOWM Staphylococcus aureus strain AA8	CC97-MRSA-IVc, WA MRSA-54/63	POS	POS	NEG	POS	NEG	POS	POS	NEG	NEG	NEG	POS
JAEOUU Staphylococcus aureus strain AA104	CC97-MRSA-V	POS	POS	NEG	POS	NEG	POS	POS	NEG	NEG	NEG	POS
JAEOVJ Staphylococcus aureus strain AA35	CC97-MRSA-[V+fus]	POS	POS	NEG	POS	NEG	POS	POS	NEG	NEG	NEG	POS
JAEOVK Staphylococcus aureus strain AA36	CC97-MRSA-[V+fus]	POS	POS	NEG	POS	NEG	POS	POS	NEG	NEG	NEG	POS
**CC22**												
JAEOUY Staphylococcus aureus strain AA18	CC22-MRSA-IVa (dcs negatives) (tst1+), "Gaza Epidemic Strain"	POS	POS	NEG	NEG	POS	POS	POS	NEG	NEG	NEG	POS
JAEOVH Staphylococcus aureus strain AA32	CC22-MRSA-IVa (dcs negatives) (tst1+), "Gaza Epidemic Strain"	POS	POS	NEG	NEG	POS	POS	POS	NEG	NEG	NEG	POS
JAEOVQ Staphylococcus aureus strain AA5	CC22-MRSA-IVa (dcs negatives) (tst1+), "Gaza Epidemic Strain"	POS	POS	NEG	NEG	POS	POS	POS	NEG	NEG	NEG	POS
**CC152**												
JAEOUX Staphylococcus aureus strain AA17	CC152-MRSA-[V+fus]	POS	POS	POS	NEG	NEG	POS	POS	NEG	NEG	NEG	NEG
**CC239**												
JAEOVI Staphylococcus aureus strain AA33	CC239-MRSA-[III+Cd/Hg+ccrC] (sasX-positive), "Southeast Asian Clade"	POS	POS	POS	NEG	NEG	POS	POS	POS	NEG	NEG	NEG
JAEOUS Staphylococcus aureus strain AA101	CC239-MRSA-[III+Cd+ccrC] (sasX-negat.), "Middle Eastern Cluster"	POS	POS	POS	NEG	NEG	POS	POS	POS	NEG	NEG	NEG
JAEOUV Staphylococcus aureus strain AA13	CC239-MRSA-[III+Cd+ccrC] (sasX-negat.), "Middle Eastern Cluster"	POS	POS	POS	NEG	NEG	POS	POS	POS	NEG	NEG	NEG
JAEOUW Staphylococcus aureus strain AA14	CC239-MRSA-[III+Cd+ccrC] (sasX-negat.), "Middle Eastern Cluster"	POS	POS	POS	NEG	NEG	POS	POS	POS	NEG	NEG	NEG
JAEOVA Staphylococcus aureus strain AA22	CC239-MRSA-[III+Cd+ccrC] (sasX-negat.), "Middle Eastern Cluster"	POS	POS	POS	NEG	NEG	POS	POS	POS	NEG	NEG	NEG
JAEOVB Staphylococcus aureus strain AA23	CC239-MRSA-[III+Cd+ccrC] (sasX-negat.), "Middle Eastern Cluster"	POS	POS	POS	NEG	NEG	POS	POS	POS	NEG	NEG	NEG
JAEOVC Staphylococcus aureus strain AA27	CC239-MRSA-[III+Cd+ccrC] (sasX-negat.), "Middle Eastern Cluster"	POS	POS	POS	NEG	NEG	POS	POS	POS	NEG	NEG	NEG
JAEOVD Staphylococcus aureus strain AA29	CC239-MRSA-[III+Cd+ccrC] (sasX-negat.), "Middle Eastern Cluster"	POS	POS	POS	NEG	NEG	POS	POS	POS	NEG	NEG	NEG
JAEOVG Staphylococcus aureus strain AA31	CC239-MRSA-[III+Cd+ccrC] (sasX-negat.), "Middle Eastern Cluster"	POS	POS	POS	NEG	NEG	POS	POS	POS	NEG	NEG	NEG
JAEOVP Staphylococcus aureus strain AA46	CC239-MRSA-[III+Cd+ccrC] (sasX-negat.), "Middle Eastern Cluster"	POS	POS	POS	NEG	NEG	POS	POS	POS	NEG	NEG	NEG
JAEOVS Staphylococcus aureus strain AA52	CC239-MRSA-[III+Cd+ccrC] (sasX-negat.), "Middle Eastern Cluster"	POS	POS	POS	NEG	NEG	POS	POS	POS	NEG	NEG	NEG
JAEOVU Staphylococcus aureus strain AA55	CC239-MRSA-[III+Cd+ccrC] (sasX-negat.), "Middle Eastern Cluster"	POS	POS	POS	NEG	NEG	POS	POS	POS	NEG	NEG	NEG
JAEOVV Staphylococcus aureus strain AA57	CC239-MRSA-[III+Cd+ccrC] (sasX-negat.), "Middle Eastern Cluster"	POS	POS	POS	NEG	NEG	POS	POS	POS	NEG	NEG	NEG
JAEOVY Staphylococcus aureus strain AA60	CC239-MRSA-[III+Cd+ccrC] (sasX-negat.), "Middle Eastern Cluster"	POS	POS	POS	NEG	NEG	POS	POS	POS	NEG	NEG	NEG
JAEOVZ Staphylococcus aureus strain AA61	CC239-MRSA-[III+Cd+ccrC] (sasX-negat.), "Middle Eastern Cluster"	POS	POS	POS	NEG	NEG	POS	POS	POS	NEG	NEG	NEG
JAEOWA Staphylococcus aureus strain AA62	CC239-MRSA-[III+Cd+ccrC] (sasX-negat.), "Middle Eastern Cluster"	POS	POS	POS	NEG	NEG	POS	POS	POS	NEG	NEG	NEG
JAEOWB Staphylococcus aureus strain AA63	CC239-MRSA-[III+Cd+ccrC] (sasX-negat.), "Middle Eastern Cluster"	POS	POS	POS	NEG	NEG	POS	POS	POS	NEG	NEG	NEG
JAEOWL Staphylococcus aureus strain AA79	CC239-MRSA-[III+Cd+ccrC] (sasX-negat.), "Middle Eastern Cluster"	POS	POS	POS	NEG	NEG	POS	POS	POS	NEG	NEG	NEG
JAEOWQ Staphylococcus aureus strain AA92	CC239-MRSA-[III+Cd+ccrC] (sasX-negat.), "Middle Eastern Cluster"	POS	POS	POS	NEG	NEG	POS	POS	POS	NEG	NEG	NEG
JAEOWC Staphylococcus aureus strain AA64	CC239-MRSA-[mec III+Cd], sasX-negative	POS	POS	POS	NEG	NEG	POS	POS	POS	NEG	NEG	NEG
JAEOWP Staphylococcus aureus strain AA91	CC239-MRSA-[mec III+Cd], sasX-negative	POS	POS	POS	NEG	NEG	POS	POS	POS	NEG	NEG	NEG

Isolate	Strain assignment	isaB	isaB (MRSA252)	mprF (COL+MW2)	mprF (Mu50+252)	isdA (cons)	isdA (MRSA252)	isdA (Other Than MRSA252)	lmrP (OtherThanRF122)	lmrP (RF122)
		MISCELLANEOUS GENES								
		isaB		mprF		isdA			lmrP	
		immunodominant antigen B		defensin resistance protein		transferrin-binding protein			hypothetical protein, similar to integral membrane protein LmrP	
**CC1**										
Alexandria University_2020-17	CC1-MRSA-[V+fus+tir]	POS	AMB	POS	AMB	POS	NEG	POS	POS	NEG
Alexandria University_2020-03	CC1-MRSA-[V+fus+tir+ccrAB1]	POS	AMB	POS	AMB	POS	NEG	POS	POS	NEG
Alexandria University_2020-19	CC1-MRSA-[V+fus+tir+ccrAB1]	POS	AMB	POS	NEG	POS	NEG	POS	POS	NEG
Alexandria University_2020-21	CC1-MRSA-[V+fus+tir+ccrAB1]	NEG	NEG	POS	AMB	POS	NEG	POS	POS	NEG
Alexandria University_2020-09	CC1-MRSA-[V+fus+tir+ccrAB1] (PVL+)	POS	AMB	POS	AMB	POS	NEG	POS	POS	NEG
Alexandria University_2020-14	CC1-MRSA-[V+fus+tir+ccrAB1] (PVL+)	POS	AMB	POS	AMB	POS	NEG	POS	POS	NEG
Alexandria University_2020-24	CC1-MRSA-[V+fus+tir+ccrAB1] (PVL+)	POS	AMB	POS	AMB	POS	NEG	POS	POS	NEG
Alexandria University_2020-27	CC1-MRSA-[V+fus+tir+ccrAB1] (PVL+)	POS	AMB	POS	AMB	POS	NEG	POS	POS	NEG
Alexandria University_2020-28	CC1-MRSA-[V+fus+tir+ccrAB1] (PVL+)	POS	AMB	POS	NEG	POS	NEG	POS	POS	NEG
**CC5**										
Alexandria University_2020-12	CC5-MRSA-[VI+fus+tir]	POS	AMB	AMB	POS	POS	NEG	POS	POS	NEG
Alexandria University_2020-20	CC5-MRSA-[VI+fus+tir]	POS	AMB	AMB	POS	POS	NEG	POS	POS	NEG
**CC6**										
Alexandria University_2020-29	CC6-MRSA-[V+fus]	POS	AMB	POS	AMB	POS	NEG	POS	POS	NEG
**CC15**										
Alexandria University_2020-01	CC15-MRSA-[V+fus]	POS	AMB	POS	NEG	POS	POS	NEG	POS	NEG
Alexandria University_2020-02	CC15-MRSA-[V+fus]	POS	AMB	POS	NEG	POS	POS	NEG	POS	NEG
Alexandria University_2020-05	CC15-MRSA-[V+fus]	POS	AMB	POS	NEG	POS	POS	NEG	POS	NEG
Alexandria University_2020-15	CC15-MRSA-[V+fus]	POS	AMB	POS	NEG	POS	POS	NEG	POS	NEG
Alexandria University_2020-22	CC15-MRSA-[V+fus]	POS	AMB	POS	NEG	POS	POS	NEG	POS	NEG
Alexandria University_2020-32	CC15-MRSA-[V+fus]	POS	AMB	POS	NEG	POS	POS	NEG	POS	NEG
**CC22**										
Alexandria University_2020-04	CC22-MRSA-IVa (dcs negatives) (tst1+), "Gaza Epidemic Strain"	NEG	POS	NEG	POS	POS	NEG	POS	POS	NEG
Alexandria University_2020-11	CC22-MRSA-IVa (dcs negatives) (tst1+), "Gaza Epidemic Strain"	NEG	POS	AMB	POS	POS	NEG	POS	POS	NEG
**CC30**										
Alexandria University_2020-26	CC30-MRSA-IVa (PVL+), "WSPP/Southwest Pacific Clone"	NEG	POS	AMB	POS	POS	POS	NEG	POS	NEG
**CC97**										
Alexandria University_2020-30	CC97-MRSA-[V+fus]	POS	AMB	POS	NEG	POS	NEG	POS	POS	NEG
**CC121**										
Alexandria University_2020-06	CC121-MRSA-[V+fus] (PVL+)	POS	AMB	POS	AMB	POS	NEG	POS	NEG	POS
**CC152**										
Alexandria University_2020-18	CC152-MRSA-[V+fus]	POS	AMB	POS	NEG	POS	NEG	NEG	NEG	POS
Alexandria University_2020-31	CC152-MRSA-[V+fus]	POS	AMB	POS	NEG	POS	NEG	NEG	NEG	POS
**CC239**										
Alexandria University_2020-13	CC239-MRSA-[III+Cd+ccrC] (sasX-negat.), "Middle Eastern Cluster"	NEG	POS	POS	AMB	POS	NEG	POS	POS	NEG
Alexandria University_2020-16	CC239-MRSA-[III+Cd], sasX-negative	NEG	POS	POS	NEG	POS	NEG	POS	POS	NEG
**CC1153**										
Alexandria University_2020-07	CC1153-MRSA-[V+fus] (PVL+)	POS	AMB	NEG	POS	AMB	NEG	POS	AMB	NEG
Alexandria University_2020-08	CC1153-MRSA-[V+fus] (PVL+)	POS	AMB	AMB	POS	AMB	NEG	POS	POS	NEG
Alexandria University_2020-23	CC1153-MRSA-[V+fus] (PVL+)	POS	AMB	AMB	POS	POS	NEG	POS	POS	NEG
**Simulated hybridisations for sequences from Montelongo et al., 2022**										
**CC1**										
JAEOUR Staphylococcus aureus strain AA1	CC1-MRSA-[V+fus+tir+ccrAB1]	POS	NEG	POS	NEG	POS	NEG	POS	POS	NEG
JAEOWK Staphylococcus aureus strain AA78	CC1-MRSA-[V+fus+tir+ccrAB1]	POS	NEG	POS	NEG	POS	NEG	POS	POS	NEG
JAEOVR Staphylococcus aureus strain AA51	CC1-MRSA-[V+fus+tir+ccrAB1] (PVL+)	POS	NEG	POS	NEG	POS	NEG	POS	POS	NEG
JAEOWE Staphylococcus aureus strain AA67	CC1-MRSA-[V+fus+tir+ccrAB1] (PVL+)	POS	NEG	POS	NEG	POS	NEG	POS	POS	NEG
JAEOWG Staphylococcus aureus strain AA69	CC1-MRSA-[V+fus+tir+ccrAB1] (PVL+)	POS	NEG	POS	NEG	POS	NEG	POS	POS	NEG
JAEOWJ Staphylococcus aureus strain AA77	CC1-MRSA-[V+fus+tir+ccrAB1] (PVL+)	POS	NEG	POS	NEG	POS	NEG	POS	POS	NEG
**CC5**										
JAEOWI Staphylococcus aureus strain AA76	CC5-MRSA-[V+cas], WA MRSA-123	POS	NEG	NEG	POS	POS	NEG	POS	POS	NEG
JAEOWN Staphylococcus aureus strain AA80	CC5-MRSA-[V+cas], WA MRSA-123	POS	NEG	NEG	POS	POS	NEG	POS	POS	NEG
JAEOWH Staphylococcus aureus strain AA70	CC5-MRSA-[VI+fus+tir+, dcs-]	POS	NEG	NEG	POS	POS	NEG	POS	POS	NEG
**CC6**										
JAEOVF Staphylococcus aureus strain AA30	CC6-MRSA-IVa, WA MRSA-51	POS	NEG	POS	NEG	POS	NEG	POS	POS	NEG
**CC80**										
JAEOVM Staphylococcus aureus strain AA4	CC80-MRSA-IVc (PVL-) [aphA3/sat+]:[far1+], contaminated	AMB	AMB	NEG	POS	POS	NEG	POS	POS	NEG
JAEOUZ Staphylococcus aureus strain AA2	CC80-MRSA-IVc (PVL+) [aphA3/sat+]:[far1+]	POS	NEG	NEG	POS	POS	NEG	POS	POS	NEG
JAEOVE Staphylococcus aureus strain AA3	CC80-MRSA-IVc (PVL+) [aphA3/sat+]:[far1+]	POS	NEG	NEG	POS	POS	NEG	POS	POS	NEG
JAEOVO Staphylococcus aureus strain AA45	CC80-MRSA-IVc (PVL+) [aphA3/sat+]:[far1+]	POS	NEG	NEG	POS	POS	NEG	POS	POS	NEG
**CC88**										
JAEOVT Staphylococcus aureus strain AA53	CC88-MRSA-IV, contaminated	POS	NEG	POS	NEG	POS	NEG	POS	POS	NEG
**CC97**										
JAEOVL Staphylococcus aureus strain AA39	CC97-MRSA-IVc, WA MRSA-54/63	POS	NEG	POS	NEG	POS	NEG	POS	POS	NEG
JAEOVX Staphylococcus aureus strain AA6	CC97-MRSA-IVc, WA MRSA-54/63	POS	NEG	POS	NEG	POS	NEG	POS	POS	NEG
JAEOWM Staphylococcus aureus strain AA8	CC97-MRSA-IVc, WA MRSA-54/63	POS	NEG	POS	NEG	POS	NEG	POS	POS	NEG
JAEOUU Staphylococcus aureus strain AA104	CC97-MRSA-V	POS	NEG	POS	NEG	POS	NEG	POS	POS	NEG
JAEOVJ Staphylococcus aureus strain AA35	CC97-MRSA-[V+fus]	POS	NEG	POS	NEG	POS	NEG	POS	POS	NEG
JAEOVK Staphylococcus aureus strain AA36	CC97-MRSA-[V+fus]	POS	NEG	POS	NEG	POS	NEG	POS	POS	NEG
**CC22**										
JAEOUY Staphylococcus aureus strain AA18	CC22-MRSA-IVa (dcs negatives) (tst1+), "Gaza Epidemic Strain"	NEG	POS	NEG	POS	POS	NEG	POS	POS	NEG
JAEOVH Staphylococcus aureus strain AA32	CC22-MRSA-IVa (dcs negatives) (tst1+), "Gaza Epidemic Strain"	NEG	POS	NEG	POS	POS	NEG	POS	POS	NEG
JAEOVQ Staphylococcus aureus strain AA5	CC22-MRSA-IVa (dcs negatives) (tst1+), "Gaza Epidemic Strain"	NEG	POS	NEG	POS	POS	NEG	POS	POS	NEG
**CC152**										
JAEOUX Staphylococcus aureus strain AA17	CC152-MRSA-[V+fus]	POS	NEG	POS	NEG	AMB	NEG	NEG	NEG	POS
**CC239**										
JAEOVI Staphylococcus aureus strain AA33	CC239-MRSA-[III+Cd/Hg+ccrC] (sasX-positive), "Southeast Asian Clade"	NEG	POS	POS	NEG	POS	NEG	POS	POS	NEG
JAEOUS Staphylococcus aureus strain AA101	CC239-MRSA-[III+Cd+ccrC] (sasX-negat.), "Middle Eastern Cluster"	NEG	POS	POS	NEG	POS	NEG	POS	POS	NEG
JAEOUV Staphylococcus aureus strain AA13	CC239-MRSA-[III+Cd+ccrC] (sasX-negat.), "Middle Eastern Cluster"	NEG	POS	POS	NEG	POS	NEG	POS	POS	NEG
JAEOUW Staphylococcus aureus strain AA14	CC239-MRSA-[III+Cd+ccrC] (sasX-negat.), "Middle Eastern Cluster"	NEG	POS	POS	NEG	POS	NEG	POS	POS	NEG
JAEOVA Staphylococcus aureus strain AA22	CC239-MRSA-[III+Cd+ccrC] (sasX-negat.), "Middle Eastern Cluster"	NEG	POS	POS	NEG	POS	NEG	POS	POS	NEG
JAEOVB Staphylococcus aureus strain AA23	CC239-MRSA-[III+Cd+ccrC] (sasX-negat.), "Middle Eastern Cluster"	NEG	POS	POS	NEG	POS	NEG	POS	POS	NEG
JAEOVC Staphylococcus aureus strain AA27	CC239-MRSA-[III+Cd+ccrC] (sasX-negat.), "Middle Eastern Cluster"	NEG	POS	POS	NEG	POS	NEG	POS	POS	NEG
JAEOVD Staphylococcus aureus strain AA29	CC239-MRSA-[III+Cd+ccrC] (sasX-negat.), "Middle Eastern Cluster"	NEG	POS	POS	NEG	POS	NEG	POS	POS	NEG
JAEOVG Staphylococcus aureus strain AA31	CC239-MRSA-[III+Cd+ccrC] (sasX-negat.), "Middle Eastern Cluster"	NEG	POS	POS	NEG	POS	NEG	POS	POS	NEG
JAEOVP Staphylococcus aureus strain AA46	CC239-MRSA-[III+Cd+ccrC] (sasX-negat.), "Middle Eastern Cluster"	NEG	POS	POS	NEG	POS	NEG	POS	POS	NEG
JAEOVS Staphylococcus aureus strain AA52	CC239-MRSA-[III+Cd+ccrC] (sasX-negat.), "Middle Eastern Cluster"	NEG	POS	POS	NEG	POS	NEG	POS	POS	NEG
JAEOVU Staphylococcus aureus strain AA55	CC239-MRSA-[III+Cd+ccrC] (sasX-negat.), "Middle Eastern Cluster"	NEG	POS	POS	NEG	POS	NEG	POS	POS	NEG
JAEOVV Staphylococcus aureus strain AA57	CC239-MRSA-[III+Cd+ccrC] (sasX-negat.), "Middle Eastern Cluster"	NEG	POS	POS	NEG	POS	NEG	POS	POS	NEG
JAEOVY Staphylococcus aureus strain AA60	CC239-MRSA-[III+Cd+ccrC] (sasX-negat.), "Middle Eastern Cluster"	NEG	POS	POS	NEG	POS	NEG	POS	POS	NEG
JAEOVZ Staphylococcus aureus strain AA61	CC239-MRSA-[III+Cd+ccrC] (sasX-negat.), "Middle Eastern Cluster"	NEG	POS	POS	NEG	POS	NEG	POS	POS	NEG
JAEOWA Staphylococcus aureus strain AA62	CC239-MRSA-[III+Cd+ccrC] (sasX-negat.), "Middle Eastern Cluster"	NEG	POS	POS	NEG	POS	NEG	POS	POS	NEG
JAEOWB Staphylococcus aureus strain AA63	CC239-MRSA-[III+Cd+ccrC] (sasX-negat.), "Middle Eastern Cluster"	NEG	POS	POS	NEG	POS	NEG	POS	POS	NEG
JAEOWL Staphylococcus aureus strain AA79	CC239-MRSA-[III+Cd+ccrC] (sasX-negat.), "Middle Eastern Cluster"	NEG	POS	POS	NEG	POS	NEG	POS	POS	NEG
JAEOWQ Staphylococcus aureus strain AA92	CC239-MRSA-[III+Cd+ccrC] (sasX-negat.), "Middle Eastern Cluster"	NEG	POS	POS	NEG	POS	NEG	POS	POS	NEG
JAEOWC Staphylococcus aureus strain AA64	CC239-MRSA-[mec III+Cd], sasX-negative	NEG	POS	POS	NEG	POS	NEG	POS	POS	NEG
JAEOWP Staphylococcus aureus strain AA91	CC239-MRSA-[mec III+Cd], sasX-negative	NEG	POS	POS	NEG	POS	NEG	POS	POS	NEG

Isolate	Strain assignment	TYPE I RESTRICTION-MODIFICATION SYSTEM, SINGLE SEQUENCE SPECIFICITY PROTEIN											
		hsdS1	hsdS2			hsdS3					hsdSx		
		hsdS1-RF122	hsdS2-ST5+ST8	hsdS2-MW2+476	hsdS2-MRSA252	hsdS3-AllOtherThanRF122+252	hsdS3-ST8+ST1+RF122	hsdS3-Mu50+N315	hsdS3-CC51+252	hsdS3-MRSA252	hsdSx-CC25	hsdSx-CC15	hsdSx-etd
		type I site-specific deoxyribo-nuclease subunit, 1st locus	type I site-specific deoxyribonuclease subunit, 2nd locus			type I site-specific deoxyribonuclease subunit, 3rd locus					type I site-specific deoxyribonuclease subunit, unknown locus		
**CC1**													
Alexandria University_2020-17	CC1-MRSA-[V+fus+tir]	NEG	NEG	POS	NEG	POS	POS	NEG	NEG	NEG	POS	NEG	NEG
Alexandria University_2020-03	CC1-MRSA-[V+fus+tir+ccrAB1]	NEG	NEG	POS	NEG	POS	POS	NEG	NEG	NEG	POS	NEG	NEG
Alexandria University_2020-19	CC1-MRSA-[V+fus+tir+ccrAB1]	NEG	NEG	POS	NEG	POS	POS	NEG	NEG	NEG	POS	NEG	NEG
Alexandria University_2020-21	CC1-MRSA-[V+fus+tir+ccrAB1]	NEG	NEG	POS	NEG	POS	POS	NEG	NEG	NEG	POS	NEG	NEG
Alexandria University_2020-09	CC1-MRSA-[V+fus+tir+ccrAB1] (PVL+)	NEG	NEG	POS	NEG	POS	POS	NEG	NEG	NEG	POS	NEG	NEG
Alexandria University_2020-14	CC1-MRSA-[V+fus+tir+ccrAB1] (PVL+)	NEG	NEG	POS	NEG	POS	POS	NEG	NEG	NEG	POS	NEG	NEG
Alexandria University_2020-24	CC1-MRSA-[V+fus+tir+ccrAB1] (PVL+)	NEG	NEG	POS	NEG	POS	POS	NEG	NEG	NEG	POS	NEG	NEG
Alexandria University_2020-27	CC1-MRSA-[V+fus+tir+ccrAB1] (PVL+)	NEG	NEG	POS	NEG	POS	POS	NEG	NEG	NEG	POS	NEG	NEG
Alexandria University_2020-28	CC1-MRSA-[V+fus+tir+ccrAB1] (PVL+)	NEG	NEG	POS	NEG	POS	POS	NEG	NEG	NEG	POS	NEG	NEG
**CC5**													
Alexandria University_2020-12	CC5-MRSA-[VI+fus+tir]	NEG	NEG	NEG	NEG	POS	NEG	POS	NEG	NEG	POS	AMB	NEG
Alexandria University_2020-20	CC5-MRSA-[VI+fus+tir]	NEG	NEG	NEG	NEG	POS	NEG	POS	NEG	NEG	POS	NEG	NEG
**CC6**													
Alexandria University_2020-29	CC6-MRSA-[V+fus]	NEG	NEG	NEG	NEG	POS	POS	NEG	POS	NEG	POS	NEG	NEG
**CC15**													
Alexandria University_2020-01	CC15-MRSA-[V+fus]	NEG	NEG	NEG	NEG	NEG	NEG	NEG	NEG	NEG	NEG	POS	NEG
Alexandria University_2020-02	CC15-MRSA-[V+fus]	NEG	NEG	NEG	NEG	NEG	NEG	NEG	NEG	NEG	NEG	POS	NEG
Alexandria University_2020-05	CC15-MRSA-[V+fus]	NEG	NEG	NEG	NEG	NEG	NEG	NEG	NEG	NEG	NEG	POS	NEG
Alexandria University_2020-15	CC15-MRSA-[V+fus]	NEG	NEG	NEG	NEG	NEG	NEG	NEG	NEG	NEG	NEG	POS	NEG
Alexandria University_2020-22	CC15-MRSA-[V+fus]	NEG	NEG	NEG	NEG	NEG	NEG	NEG	NEG	NEG	NEG	POS	NEG
Alexandria University_2020-32	CC15-MRSA-[V+fus]	NEG	NEG	NEG	NEG	NEG	NEG	NEG	NEG	NEG	NEG	POS	NEG
**CC22**													
Alexandria University_2020-04	CC22-MRSA-IVa (dcs negatives) (tst1+), "Gaza Epidemic Strain"	NEG	POS	NEG	NEG	NEG	NEG	NEG	NEG	NEG	POS	NEG	NEG
Alexandria University_2020-11	CC22-MRSA-IVa (dcs negatives) (tst1+), "Gaza Epidemic Strain"	NEG	POS	NEG	NEG	AMB	NEG	NEG	NEG	NEG	POS	NEG	NEG
**CC30**													
Alexandria University_2020-26	CC30-MRSA-IVa (PVL+), "WSPP/Southwest Pacific Clone"	NEG	NEG	NEG	POS	NEG	NEG	NEG	POS	POS	POS	NEG	NEG
**CC97**													
Alexandria University_2020-30	CC97-MRSA-[V+fus]	NEG	NEG	NEG	NEG	NEG	NEG	NEG	NEG	NEG	POS	NEG	NEG
**CC121**													
Alexandria University_2020-06	CC121-MRSA-[V+fus] (PVL+)	NEG	POS	NEG	NEG	NEG	NEG	NEG	POS	NEG	POS	AMB	NEG
**CC152**													
Alexandria University_2020-18	CC152-MRSA-[V+fus]	NEG	POS	NEG	NEG	POS	NEG	NEG	POS	POS	POS	NEG	POS
Alexandria University_2020-31	CC152-MRSA-[V+fus]	NEG	POS	NEG	NEG	NEG	NEG	NEG	POS	POS	POS	NEG	POS
**CC239**													
Alexandria University_2020-13	CC239-MRSA-[III+Cd+ccrC] (sasX-negat.), "Middle Eastern Cluster"	NEG	NEG	NEG	NEG	POS	POS	NEG	NEG	NEG	POS	NEG	NEG
Alexandria University_2020-16	CC239-MRSA-[III+Cd], sasX-negative	NEG	NEG	NEG	NEG	POS	POS	NEG	NEG	NEG	POS	NEG	NEG
**CC1153**													
Alexandria University_2020-07	CC1153-MRSA-[V+fus] (PVL+)	NEG	POS	NEG	NEG	NEG	NEG	NEG	NEG	NEG	AMB	AMB	NEG
Alexandria University_2020-08	CC1153-MRSA-[V+fus] (PVL+)	NEG	POS	NEG	NEG	NEG	NEG	NEG	NEG	NEG	POS	AMB	NEG
Alexandria University_2020-23	CC1153-MRSA-[V+fus] (PVL+)	NEG	POS	NEG	NEG	AMB	NEG	NEG	NEG	NEG	POS	NEG	NEG
**Simulated hybridisations for sequences from Montelongo et al., 2022**													
**CC1**													
JAEOUR Staphylococcus aureus strain AA1	CC1-MRSA-[V+fus+tir+ccrAB1]	NEG	NEG	POS	NEG	AMB	POS	NEG	NEG	NEG	POS	NEG	NEG
JAEOWK Staphylococcus aureus strain AA78	CC1-MRSA-[V+fus+tir+ccrAB1]	NEG	NEG	POS	NEG	AMB	POS	NEG	NEG	NEG	POS	NEG	NEG
JAEOVR Staphylococcus aureus strain AA51	CC1-MRSA-[V+fus+tir+ccrAB1] (PVL+)	NEG	NEG	POS	NEG	AMB	POS	NEG	NEG	NEG	AMB	NEG	NEG
JAEOWE Staphylococcus aureus strain AA67	CC1-MRSA-[V+fus+tir+ccrAB1] (PVL+)	NEG	NEG	POS	NEG	AMB	POS	NEG	NEG	NEG	POS	NEG	NEG
JAEOWG Staphylococcus aureus strain AA69	CC1-MRSA-[V+fus+tir+ccrAB1] (PVL+)	NEG	NEG	POS	NEG	AMB	POS	NEG	NEG	NEG	POS	NEG	NEG
JAEOWJ Staphylococcus aureus strain AA77	CC1-MRSA-[V+fus+tir+ccrAB1] (PVL+)	NEG	NEG	POS	NEG	AMB	POS	NEG	NEG	NEG	POS	NEG	NEG
**CC5**													
JAEOWI Staphylococcus aureus strain AA76	CC5-MRSA-[V+cas], WA MRSA-123	NEG	POS	NEG	NEG	AMB	NEG	POS	NEG	NEG	POS	NEG	NEG
JAEOWN Staphylococcus aureus strain AA80	CC5-MRSA-[V+cas], WA MRSA-123	NEG	POS	NEG	NEG	AMB	NEG	POS	NEG	NEG	POS	NEG	NEG
JAEOWH Staphylococcus aureus strain AA70	CC5-MRSA-[VI+fus+tir+, dcs-]	NEG	NEG	NEG	NEG	AMB	NEG	POS	NEG	NEG	POS	NEG	NEG
**CC6**													
JAEOVF Staphylococcus aureus strain AA30	CC6-MRSA-IVa, WA MRSA-51	NEG	NEG	NEG	NEG	AMB	POS	NEG	POS	NEG	AMB	NEG	NEG
**CC80**													
JAEOVM Staphylococcus aureus strain AA4	CC80-MRSA-IVc (PVL-) [aphA3/sat+]:[far1+], contaminated	NEG	NEG	NEG	NEG	NEG	NEG	NEG	NEG	NEG	NEG	NEG	POS
JAEOUZ Staphylococcus aureus strain AA2	CC80-MRSA-IVc (PVL+) [aphA3/sat+]:[far1+]	NEG	NEG	NEG	NEG	NEG	NEG	NEG	NEG	NEG	NEG	NEG	POS
JAEOVE Staphylococcus aureus strain AA3	CC80-MRSA-IVc (PVL+) [aphA3/sat+]:[far1+]	NEG	NEG	NEG	NEG	NEG	NEG	NEG	NEG	NEG	NEG	NEG	POS
JAEOVO Staphylococcus aureus strain AA45	CC80-MRSA-IVc (PVL+) [aphA3/sat+]:[far1+]	NEG	NEG	NEG	NEG	NEG	NEG	NEG	NEG	NEG	NEG	NEG	POS
**CC88**													
JAEOVT Staphylococcus aureus strain AA53	CC88-MRSA-IV, contaminated	AMB	NEG	NEG	NEG	NEG	NEG	NEG	NEG	POS	AMB	NEG	NEG
**CC97**													
JAEOVL Staphylococcus aureus strain AA39	CC97-MRSA-IVc, WA MRSA-54/63	NEG	NEG	NEG	NEG	NEG	NEG	NEG	NEG	NEG	AMB	NEG	NEG
JAEOVX Staphylococcus aureus strain AA6	CC97-MRSA-IVc, WA MRSA-54/63	NEG	NEG	NEG	NEG	NEG	NEG	NEG	NEG	NEG	AMB	NEG	NEG
JAEOWM Staphylococcus aureus strain AA8	CC97-MRSA-IVc, WA MRSA-54/63	NEG	NEG	NEG	NEG	NEG	NEG	NEG	NEG	NEG	AMB	NEG	NEG
JAEOUU Staphylococcus aureus strain AA104	CC97-MRSA-V	NEG	NEG	NEG	NEG	NEG	NEG	NEG	NEG	NEG	AMB	NEG	NEG
JAEOVJ Staphylococcus aureus strain AA35	CC97-MRSA-[V+fus]	NEG	NEG	NEG	NEG	NEG	NEG	NEG	NEG	NEG	AMB	NEG	NEG
JAEOVK Staphylococcus aureus strain AA36	CC97-MRSA-[V+fus]	NEG	NEG	NEG	NEG	NEG	NEG	NEG	NEG	NEG	AMB	NEG	NEG
**CC22**													
JAEOUY Staphylococcus aureus strain AA18	CC22-MRSA-IVa (dcs negatives) (tst1+), "Gaza Epidemic Strain"	NEG	POS	NEG	NEG	AMB	NEG	NEG	NEG	NEG	AMB	NEG	NEG
JAEOVH Staphylococcus aureus strain AA32	CC22-MRSA-IVa (dcs negatives) (tst1+), "Gaza Epidemic Strain"	NEG	POS	NEG	NEG	AMB	NEG	NEG	NEG	NEG	AMB	NEG	NEG
JAEOVQ Staphylococcus aureus strain AA5	CC22-MRSA-IVa (dcs negatives) (tst1+), "Gaza Epidemic Strain"	NEG	POS	NEG	NEG	AMB	NEG	NEG	NEG	NEG	AMB	NEG	NEG
**CC152**													
JAEOUX Staphylococcus aureus strain AA17	CC152-MRSA-[V+fus]	NEG	POS	NEG	NEG	NEG	NEG	NEG	POS	POS	AMB	NEG	AMB
**CC239**													
JAEOVI Staphylococcus aureus strain AA33	CC239-MRSA-[III+Cd/Hg+ccrC] (sasX-positive), "Southeast Asian Clade"	NEG	POS	NEG	NEG	AMB	POS	NEG	NEG	NEG	POS	NEG	NEG
JAEOUS Staphylococcus aureus strain AA101	CC239-MRSA-[III+Cd+ccrC] (sasX-negat.), "Middle Eastern Cluster"	NEG	NEG	NEG	NEG	AMB	POS	NEG	NEG	NEG	POS	NEG	NEG
JAEOUV Staphylococcus aureus strain AA13	CC239-MRSA-[III+Cd+ccrC] (sasX-negat.), "Middle Eastern Cluster"	NEG	NEG	NEG	NEG	AMB	POS	NEG	NEG	NEG	POS	NEG	NEG
JAEOUW Staphylococcus aureus strain AA14	CC239-MRSA-[III+Cd+ccrC] (sasX-negat.), "Middle Eastern Cluster"	NEG	NEG	NEG	NEG	AMB	POS	NEG	NEG	NEG	POS	NEG	NEG
JAEOVA Staphylococcus aureus strain AA22	CC239-MRSA-[III+Cd+ccrC] (sasX-negat.), "Middle Eastern Cluster"	NEG	NEG	NEG	NEG	AMB	POS	NEG	NEG	NEG	POS	NEG	NEG
JAEOVB Staphylococcus aureus strain AA23	CC239-MRSA-[III+Cd+ccrC] (sasX-negat.), "Middle Eastern Cluster"	NEG	NEG	NEG	NEG	AMB	POS	NEG	NEG	NEG	POS	NEG	NEG
JAEOVC Staphylococcus aureus strain AA27	CC239-MRSA-[III+Cd+ccrC] (sasX-negat.), "Middle Eastern Cluster"	NEG	NEG	NEG	NEG	AMB	POS	NEG	NEG	NEG	POS	NEG	NEG
JAEOVD Staphylococcus aureus strain AA29	CC239-MRSA-[III+Cd+ccrC] (sasX-negat.), "Middle Eastern Cluster"	NEG	NEG	NEG	NEG	AMB	POS	NEG	NEG	NEG	POS	NEG	NEG
JAEOVG Staphylococcus aureus strain AA31	CC239-MRSA-[III+Cd+ccrC] (sasX-negat.), "Middle Eastern Cluster"	NEG	NEG	NEG	NEG	AMB	POS	NEG	NEG	NEG	POS	NEG	NEG
JAEOVP Staphylococcus aureus strain AA46	CC239-MRSA-[III+Cd+ccrC] (sasX-negat.), "Middle Eastern Cluster"	NEG	NEG	NEG	NEG	AMB	POS	NEG	NEG	NEG	POS	NEG	NEG
JAEOVS Staphylococcus aureus strain AA52	CC239-MRSA-[III+Cd+ccrC] (sasX-negat.), "Middle Eastern Cluster"	NEG	NEG	NEG	NEG	AMB	POS	NEG	NEG	NEG	POS	NEG	NEG
JAEOVU Staphylococcus aureus strain AA55	CC239-MRSA-[III+Cd+ccrC] (sasX-negat.), "Middle Eastern Cluster"	NEG	NEG	NEG	NEG	AMB	POS	NEG	NEG	NEG	POS	NEG	NEG
JAEOVV Staphylococcus aureus strain AA57	CC239-MRSA-[III+Cd+ccrC] (sasX-negat.), "Middle Eastern Cluster"	NEG	NEG	NEG	NEG	AMB	POS	NEG	NEG	NEG	POS	NEG	NEG
JAEOVY Staphylococcus aureus strain AA60	CC239-MRSA-[III+Cd+ccrC] (sasX-negat.), "Middle Eastern Cluster"	NEG	NEG	NEG	NEG	AMB	POS	NEG	NEG	NEG	POS	NEG	NEG
JAEOVZ Staphylococcus aureus strain AA61	CC239-MRSA-[III+Cd+ccrC] (sasX-negat.), "Middle Eastern Cluster"	NEG	NEG	NEG	NEG	AMB	POS	NEG	NEG	NEG	POS	NEG	NEG
JAEOWA Staphylococcus aureus strain AA62	CC239-MRSA-[III+Cd+ccrC] (sasX-negat.), "Middle Eastern Cluster"	NEG	NEG	NEG	NEG	AMB	POS	NEG	NEG	NEG	POS	NEG	NEG
JAEOWB Staphylococcus aureus strain AA63	CC239-MRSA-[III+Cd+ccrC] (sasX-negat.), "Middle Eastern Cluster"	NEG	NEG	NEG	NEG	AMB	POS	NEG	NEG	NEG	POS	NEG	NEG
JAEOWL Staphylococcus aureus strain AA79	CC239-MRSA-[III+Cd+ccrC] (sasX-negat.), "Middle Eastern Cluster"	NEG	NEG	NEG	NEG	AMB	POS	NEG	NEG	NEG	POS	NEG	NEG
JAEOWQ Staphylococcus aureus strain AA92	CC239-MRSA-[III+Cd+ccrC] (sasX-negat.), "Middle Eastern Cluster"	NEG	NEG	NEG	NEG	AMB	POS	NEG	NEG	NEG	POS	NEG	NEG
JAEOWC Staphylococcus aureus strain AA64	CC239-MRSA-[mec III+Cd], sasX-negative	NEG	NEG	NEG	NEG	AMB	POS	NEG	NEG	NEG	POS	NEG	NEG
JAEOWP Staphylococcus aureus strain AA91	CC239-MRSA-[mec III+Cd], sasX-negative	NEG	NEG	NEG	NEG	AMB	POS	NEG	NEG	NEG	POS	NEG	NEG

Isolate	Strain assignment	MISCELLANEOUS GENES					HYALURONATE LYASE							
					ycjY	sdrM	hysA1			hysA2				
		ear2 = Q2FXC0	Q2YUB3	Q7A4X2	ycjY = C5Q1F1 ("Argenteus/ ST1850-like", CC12, CC361, CC398)	sdrM / tetEfflux	hysA1 (MRSA252)	hysA1 (MRSA252+RF122) and/or hysA2 (cons)	hysA1 (MRSA252+RF122) and/or hysA2 (COL+USA300)	hysA2 (All Other Than MRSA252)	hysA2 (COL+USA300+NCTC8325)	hysA2 (All Other Than COL+USA300+NCTC8325)	hysA2 (All Other Than COL+USA300+NCTC8325)	hysA2 (MRSA252)
		Putative protein	Multidrug resistance transporter	Putative protein		Multidrug resistance transporter	Hyaluronate lyase, variable first / second locus			Hyaluronate lyase, second locus				
**CC1**														
Alexandria University_2020-17	CC1-MRSA-[V+fus+tir]	POS	NEG	NEG	NEG	POS	NEG	POS	NEG	POS	NEG	POS	POS	NEG
Alexandria University_2020-03	CC1-MRSA-[V+fus+tir+ccrAB1]	POS	NEG	NEG	NEG	POS	NEG	POS	NEG	POS	NEG	POS	POS	NEG
Alexandria University_2020-19	CC1-MRSA-[V+fus+tir+ccrAB1]	POS	NEG	NEG	NEG	POS	NEG	POS	NEG	POS	NEG	POS	POS	NEG
Alexandria University_2020-21	CC1-MRSA-[V+fus+tir+ccrAB1]	POS	NEG	NEG	NEG	POS	NEG	POS	NEG	POS	NEG	POS	POS	NEG
Alexandria University_2020-09	CC1-MRSA-[V+fus+tir+ccrAB1] (PVL+)	POS	NEG	NEG	NEG	POS	NEG	POS	NEG	POS	NEG	POS	POS	NEG
Alexandria University_2020-14	CC1-MRSA-[V+fus+tir+ccrAB1] (PVL+)	POS	NEG	NEG	NEG	POS	NEG	POS	NEG	POS	NEG	POS	POS	NEG
Alexandria University_2020-24	CC1-MRSA-[V+fus+tir+ccrAB1] (PVL+)	POS	NEG	NEG	NEG	POS	NEG	POS	NEG	POS	NEG	POS	POS	NEG
Alexandria University_2020-27	CC1-MRSA-[V+fus+tir+ccrAB1] (PVL+)	POS	NEG	NEG	NEG	POS	NEG	POS	NEG	POS	NEG	POS	POS	NEG
Alexandria University_2020-28	CC1-MRSA-[V+fus+tir+ccrAB1] (PVL+)	NEG	NEG	NEG	NEG	POS	NEG	POS	NEG	POS	NEG	POS	POS	NEG
**CC5**														
Alexandria University_2020-12	CC5-MRSA-[VI+fus+tir]	NEG	NEG	POS	NEG	POS	NEG	POS	NEG	POS	NEG	POS	POS	NEG
Alexandria University_2020-20	CC5-MRSA-[VI+fus+tir]	NEG	NEG	POS	NEG	POS	NEG	POS	NEG	POS	NEG	POS	POS	NEG
**CC6**														
Alexandria University_2020-29	CC6-MRSA-[V+fus]	POS	NEG	NEG	POS	POS	NEG	POS	NEG	NEG	NEG	POS	POS	NEG
**CC15**														
Alexandria University_2020-01	CC15-MRSA-[V+fus]	NEG	NEG	NEG	NEG	POS	NEG	POS	POS	POS	POS	POS	POS	NEG
Alexandria University_2020-02	CC15-MRSA-[V+fus]	NEG	NEG	NEG	NEG	POS	NEG	POS	POS	POS	POS	POS	POS	NEG
Alexandria University_2020-05	CC15-MRSA-[V+fus]	NEG	NEG	NEG	NEG	POS	NEG	POS	POS	POS	POS	POS	POS	NEG
Alexandria University_2020-15	CC15-MRSA-[V+fus]	NEG	NEG	NEG	NEG	POS	NEG	POS	POS	POS	POS	POS	POS	NEG
Alexandria University_2020-22	CC15-MRSA-[V+fus]	NEG	NEG	NEG	NEG	POS	NEG	POS	POS	NEG	POS	POS	POS	NEG
Alexandria University_2020-32	CC15-MRSA-[V+fus]	NEG	NEG	NEG	NEG	POS	NEG	POS	POS	POS	POS	POS	POS	NEG
**CC22**														
Alexandria University_2020-04	CC22-MRSA-IVa (dcs negatives) (tst1+), "Gaza Epidemic Strain"	NEG	NEG	POS	NEG	NEG	NEG	POS	NEG	NEG	NEG	POS	POS	NEG
Alexandria University_2020-11	CC22-MRSA-IVa (dcs negatives) (tst1+), "Gaza Epidemic Strain"	NEG	NEG	POS	NEG	NEG	NEG	POS	NEG	NEG	NEG	POS	POS	NEG
**CC30**														
Alexandria University_2020-26	CC30-MRSA-IVa (PVL+), "WSPP/Southwest Pacific Clone"	NEG	NEG	POS	NEG	POS	POS	POS	POS	NEG	POS	POS	POS	POS
**CC97**														
Alexandria University_2020-30	CC97-MRSA-[V+fus]	NEG	NEG	NEG	NEG	POS	NEG	POS	NEG	NEG	NEG	POS	POS	NEG
**CC121**														
Alexandria University_2020-06	CC121-MRSA-[V+fus] (PVL+)	NEG	NEG	POS	NEG	POS	NEG	POS	POS	NEG	POS	NEG	NEG	NEG
**CC152**														
Alexandria University_2020-18	CC152-MRSA-[V+fus]	NEG	NEG	NEG	NEG	POS	NEG	POS	POS	NEG	POS	NEG	NEG	AMB
Alexandria University_2020-31	CC152-MRSA-[V+fus]	NEG	NEG	NEG	NEG	POS	NEG	POS	AMB	NEG	POS	NEG	NEG	NEG
**CC239**														
Alexandria University_2020-13	CC239-MRSA-[III+Cd+ccrC] (sasX-negat.), "Middle Eastern Cluster"	POS	NEG	NEG	NEG	POS	NEG	POS	POS	POS	POS	NEG	NEG	NEG
Alexandria University_2020-16	CC239-MRSA-[III+Cd], sasX-negative	POS	NEG	NEG	NEG	POS	NEG	POS	POS	POS	POS	NEG	NEG	NEG
**CC1153**														
Alexandria University_2020-07	CC1153-MRSA-[V+fus] (PVL+)	NEG	NEG	NEG	POS	POS	NEG	POS	NEG	NEG	NEG	POS	POS	NEG
Alexandria University_2020-08	CC1153-MRSA-[V+fus] (PVL+)	NEG	NEG	NEG	POS	POS	NEG	POS	NEG	NEG	NEG	POS	POS	NEG
Alexandria University_2020-23	CC1153-MRSA-[V+fus] (PVL+)	NEG	NEG	NEG	POS	POS	NEG	POS	NEG	NEG	NEG	POS	POS	NEG
**Simulated hybridisations for sequences from Montelongo et al., 2022**														
**CC1**														
JAEOUR Staphylococcus aureus strain AA1	CC1-MRSA-[V+fus+tir+ccrAB1]	POS	NEG	NEG	NEG	NEG	NEG	POS	NEG	POS	NEG	POS	AMB	NEG
JAEOWK Staphylococcus aureus strain AA78	CC1-MRSA-[V+fus+tir+ccrAB1]	POS	NEG	NEG	NEG	NEG	NEG	POS	NEG	POS	NEG	POS	AMB	NEG
JAEOVR Staphylococcus aureus strain AA51	CC1-MRSA-[V+fus+tir+ccrAB1] (PVL+)	POS	NEG	NEG	NEG	NEG	NEG	POS	NEG	POS	NEG	POS	AMB	NEG
JAEOWE Staphylococcus aureus strain AA67	CC1-MRSA-[V+fus+tir+ccrAB1] (PVL+)	POS	NEG	NEG	NEG	NEG	NEG	POS	NEG	POS	NEG	POS	AMB	NEG
JAEOWG Staphylococcus aureus strain AA69	CC1-MRSA-[V+fus+tir+ccrAB1] (PVL+)	POS	NEG	NEG	NEG	NEG	NEG	POS	NEG	POS	NEG	POS	AMB	NEG
JAEOWJ Staphylococcus aureus strain AA77	CC1-MRSA-[V+fus+tir+ccrAB1] (PVL+)	POS	NEG	NEG	NEG	NEG	NEG	POS	NEG	POS	NEG	POS	AMB	NEG
**CC5**														
JAEOWI Staphylococcus aureus strain AA76	CC5-MRSA-[V+cas], WA MRSA-123	NEG	NEG	POS	NEG	NEG	NEG	POS	NEG	POS	NEG	AMB	POS	NEG
JAEOWN Staphylococcus aureus strain AA80	CC5-MRSA-[V+cas], WA MRSA-123	NEG	NEG	POS	NEG	NEG	NEG	POS	NEG	POS	NEG	AMB	POS	NEG
JAEOWH Staphylococcus aureus strain AA70	CC5-MRSA-[VI+fus+tir+, dcs-]	NEG	NEG	POS	NEG	NEG	NEG	POS	NEG	POS	NEG	AMB	POS	NEG
**CC6**														
JAEOVF Staphylococcus aureus strain AA30	CC6-MRSA-IVa, WA MRSA-51	POS	NEG	NEG	POS	NEG	NEG	POS	NEG	NEG	NEG	AMB	AMB	NEG
**CC80**														
JAEOVM Staphylococcus aureus strain AA4	CC80-MRSA-IVc (PVL-) [aphA3/sat+]:[far1+], contaminated	NEG	NEG	NEG	NEG	NEG	NEG	POS	NEG	AMB	NEG	AMB	AMB	NEG
JAEOUZ Staphylococcus aureus strain AA2	CC80-MRSA-IVc (PVL+) [aphA3/sat+]:[far1+]	NEG	NEG	NEG	NEG	NEG	NEG	POS	NEG	AMB	NEG	AMB	AMB	NEG
JAEOVE Staphylococcus aureus strain AA3	CC80-MRSA-IVc (PVL+) [aphA3/sat+]:[far1+]	NEG	NEG	NEG	NEG	NEG	NEG	POS	NEG	AMB	NEG	AMB	AMB	NEG
JAEOVO Staphylococcus aureus strain AA45	CC80-MRSA-IVc (PVL+) [aphA3/sat+]:[far1+]	NEG	NEG	NEG	NEG	NEG	NEG	POS	NEG	AMB	NEG	AMB	AMB	NEG
**CC88**														
JAEOVT Staphylococcus aureus strain AA53	CC88-MRSA-IV, contaminated	NEG	NEG	AMB	POS	NEG	NEG	POS	NEG	POS	NEG	AMB	POS	NEG
**CC97**														
JAEOVL Staphylococcus aureus strain AA39	CC97-MRSA-IVc, WA MRSA-54/63	NEG	NEG	POS	NEG	NEG	NEG	POS	NEG	AMB	NEG	POS	POS	NEG
JAEOVX Staphylococcus aureus strain AA6	CC97-MRSA-IVc, WA MRSA-54/63	NEG	NEG	POS	NEG	NEG	NEG	POS	NEG	AMB	NEG	POS	POS	NEG
JAEOWM Staphylococcus aureus strain AA8	CC97-MRSA-IVc, WA MRSA-54/63	NEG	NEG	POS	NEG	NEG	NEG	POS	NEG	AMB	NEG	AMB	POS	NEG
JAEOUU Staphylococcus aureus strain AA104	CC97-MRSA-V	NEG	NEG	POS	NEG	NEG	NEG	POS	NEG	AMB	NEG	POS	POS	NEG
JAEOVJ Staphylococcus aureus strain AA35	CC97-MRSA-[V+fus]	NEG	NEG	NEG	NEG	NEG	NEG	POS	NEG	AMB	NEG	POS	POS	NEG
JAEOVK Staphylococcus aureus strain AA36	CC97-MRSA-[V+fus]	NEG	NEG	POS	NEG	NEG	NEG	POS	NEG	AMB	NEG	POS	POS	NEG
**CC22**														
JAEOUY Staphylococcus aureus strain AA18	CC22-MRSA-IVa (dcs negatives) (tst1+), "Gaza Epidemic Strain"	NEG	NEG	POS	NEG	POS	NEG	POS	NEG	NEG	NEG	POS	AMB	NEG
JAEOVH Staphylococcus aureus strain AA32	CC22-MRSA-IVa (dcs negatives) (tst1+), "Gaza Epidemic Strain"	NEG	NEG	POS	NEG	POS	NEG	POS	NEG	NEG	NEG	POS	AMB	NEG
JAEOVQ Staphylococcus aureus strain AA5	CC22-MRSA-IVa (dcs negatives) (tst1+), "Gaza Epidemic Strain"	NEG	NEG	POS	NEG	POS	NEG	POS	NEG	NEG	NEG	POS	AMB	NEG
**CC152**														
JAEOUX Staphylococcus aureus strain AA17	CC152-MRSA-[V+fus]	NEG	NEG	NEG	NEG	NEG	NEG	AMB	NEG	NEG	AMB	NEG	NEG	POS
**CC239**														
JAEOVI Staphylococcus aureus strain AA33	CC239-MRSA-[III+Cd/Hg+ccrC] (sasX-positive), "Southeast Asian Clade"	POS	NEG	NEG	NEG	NEG	NEG	POS	NEG	POS	POS	NEG	NEG	NEG
JAEOUS Staphylococcus aureus strain AA101	CC239-MRSA-[III+Cd+ccrC] (sasX-negat.), "Middle Eastern Cluster"	POS	NEG	NEG	NEG	NEG	NEG	POS	NEG	POS	POS	NEG	NEG	NEG
JAEOUV Staphylococcus aureus strain AA13	CC239-MRSA-[III+Cd+ccrC] (sasX-negat.), "Middle Eastern Cluster"	POS	NEG	NEG	NEG	NEG	NEG	POS	NEG	POS	POS	NEG	NEG	NEG
JAEOUW Staphylococcus aureus strain AA14	CC239-MRSA-[III+Cd+ccrC] (sasX-negat.), "Middle Eastern Cluster"	POS	NEG	NEG	NEG	NEG	NEG	POS	NEG	POS	POS	NEG	NEG	NEG
JAEOVA Staphylococcus aureus strain AA22	CC239-MRSA-[III+Cd+ccrC] (sasX-negat.), "Middle Eastern Cluster"	POS	NEG	NEG	NEG	NEG	NEG	POS	NEG	POS	POS	NEG	NEG	NEG
JAEOVB Staphylococcus aureus strain AA23	CC239-MRSA-[III+Cd+ccrC] (sasX-negat.), "Middle Eastern Cluster"	POS	NEG	NEG	NEG	NEG	NEG	POS	NEG	POS	POS	NEG	NEG	NEG
JAEOVC Staphylococcus aureus strain AA27	CC239-MRSA-[III+Cd+ccrC] (sasX-negat.), "Middle Eastern Cluster"	POS	NEG	NEG	NEG	NEG	NEG	POS	NEG	POS	POS	NEG	NEG	NEG
JAEOVD Staphylococcus aureus strain AA29	CC239-MRSA-[III+Cd+ccrC] (sasX-negat.), "Middle Eastern Cluster"	POS	NEG	NEG	NEG	NEG	NEG	POS	NEG	POS	POS	NEG	NEG	NEG
JAEOVG Staphylococcus aureus strain AA31	CC239-MRSA-[III+Cd+ccrC] (sasX-negat.), "Middle Eastern Cluster"	POS	NEG	NEG	NEG	NEG	NEG	POS	NEG	POS	POS	NEG	NEG	NEG
JAEOVP Staphylococcus aureus strain AA46	CC239-MRSA-[III+Cd+ccrC] (sasX-negat.), "Middle Eastern Cluster"	POS	NEG	NEG	NEG	NEG	NEG	POS	NEG	POS	POS	NEG	NEG	NEG
JAEOVS Staphylococcus aureus strain AA52	CC239-MRSA-[III+Cd+ccrC] (sasX-negat.), "Middle Eastern Cluster"	POS	NEG	NEG	NEG	NEG	NEG	POS	NEG	POS	POS	NEG	NEG	NEG
JAEOVU Staphylococcus aureus strain AA55	CC239-MRSA-[III+Cd+ccrC] (sasX-negat.), "Middle Eastern Cluster"	POS	NEG	NEG	NEG	NEG	NEG	POS	NEG	POS	POS	NEG	NEG	NEG
JAEOVV Staphylococcus aureus strain AA57	CC239-MRSA-[III+Cd+ccrC] (sasX-negat.), "Middle Eastern Cluster"	POS	NEG	NEG	NEG	NEG	NEG	POS	NEG	POS	POS	NEG	NEG	NEG
JAEOVY Staphylococcus aureus strain AA60	CC239-MRSA-[III+Cd+ccrC] (sasX-negat.), "Middle Eastern Cluster"	POS	NEG	NEG	NEG	NEG	NEG	POS	NEG	POS	POS	NEG	NEG	NEG
JAEOVZ Staphylococcus aureus strain AA61	CC239-MRSA-[III+Cd+ccrC] (sasX-negat.), "Middle Eastern Cluster"	POS	NEG	NEG	NEG	NEG	NEG	POS	NEG	POS	POS	NEG	NEG	NEG
JAEOWA Staphylococcus aureus strain AA62	CC239-MRSA-[III+Cd+ccrC] (sasX-negat.), "Middle Eastern Cluster"	POS	NEG	NEG	NEG	NEG	NEG	POS	NEG	POS	POS	NEG	NEG	NEG
JAEOWB Staphylococcus aureus strain AA63	CC239-MRSA-[III+Cd+ccrC] (sasX-negat.), "Middle Eastern Cluster"	POS	NEG	NEG	NEG	NEG	NEG	POS	NEG	POS	POS	NEG	NEG	NEG
JAEOWL Staphylococcus aureus strain AA79	CC239-MRSA-[III+Cd+ccrC] (sasX-negat.), "Middle Eastern Cluster"	POS	NEG	NEG	NEG	NEG	NEG	POS	NEG	POS	POS	NEG	NEG	NEG
JAEOWQ Staphylococcus aureus strain AA92	CC239-MRSA-[III+Cd+ccrC] (sasX-negat.), "Middle Eastern Cluster"	POS	NEG	NEG	NEG	NEG	NEG	POS	NEG	POS	POS	NEG	NEG	NEG
JAEOWC Staphylococcus aureus strain AA64	CC239-MRSA-[mec III+Cd], sasX-negative	POS	NEG	NEG	NEG	NEG	NEG	POS	NEG	POS	POS	NEG	NEG	NEG
JAEOWP Staphylococcus aureus strain AA91	CC239-MRSA-[mec III+Cd], sasX-negative	POS	NEG	NEG	NEG	NEG	NEG	POS	NEG	POS	POS	NEG	NEG	NEG
